# Genomic evidence for plant-parasitic nematodes as the earliest *Wolbachia* hosts

**DOI:** 10.1038/srep34955

**Published:** 2016-10-13

**Authors:** Amanda M. V. Brown, Sulochana K. Wasala, Dana K. Howe, Amy B. Peetz, Inga A. Zasada, Dee R. Denver

**Affiliations:** 1 Department of Integrative Biology, 3029 Cordley Hall, Oregon State University, Corvallis, OR 97331 USA; 2 USDA-ARS Horticultural Crops Research Laboratory, 3420 NW Orchard Avenue, Corvallis, OR 97330, USA

## Abstract

*Wolbachia*, one of the most widespread endosymbionts, is a target for biological control of mosquito-borne diseases (malaria and dengue virus), and antibiotic elimination of infectious filarial nematodes. We sequenced and analyzed the genome of a new *Wolbachia* strain (wPpe) in the plant-parasitic nematode *Pratylenchus penetrans*. Phylogenomic analyses placed wPpe as the earliest diverging *Wolbachia*, suggesting two evolutionary invasions into nematodes. The next branches comprised strains in sap-feeding insects, suggesting *Wolbachia* may have first evolved as a nutritional mutualist. Genome size, protein content, %GC, and repetitive DNA allied wPpe with mutualistic *Wolbachia*, whereas gene repertoire analyses placed it between parasite (A, B) and mutualist (C, D, F) groups. Conservation of iron metabolism genes across *Wolbachia* suggests iron homeostasis as a potential factor in its success. This study enhances our understanding of this globally pandemic endosymbiont, highlighting genetic patterns associated with host changes. Combined with future work on this strain, these genomic data could help provide potential new targets for plant-parasitic nematode control.

*Wolbachia pipientis* (alphaproteobacteria) is the most common bacterial endosymbiont of arthropods, occurring in 61–66% of insect species^1^,^2^,^3^,^4^, yet its evolution is still not well understood. This is despite decades of research into how this endosymbiont successfully manipulates host reproduction to promote vertical transmission through the female germ line^3^,^5^,^6^. The success of *Wolbachia* arises from a wide array of transmission-enhancing phenotypes, including feminization, male killing, parthenogenesis induction, cytoplasmic incompatibility, and varying degrees of metabolic mutualism^7^,^8^,^9^. A contemporary area of interest in *Wolbachia* research is its demonstrated potential to control disease through its effect on mosquito vectors of malaria and viruses (e.g. Dengue fever, Chikungunya, yellow fever, West Nile)^10^,^11^,^12^,^13^,^14^,^15^, which together cause an estimated 520 million human infections annually (World Health Organization http://www.who.int/topics/en/).

Although *Wolbachia* is most commonly known as a reproductive parasite in well-studied arthropod systems, it also functions as an obligate mutualist in other species^16^,^17^,^18^,^19^. The filarial nematodes, which cause filariasis and onchocerciasis infections in 157 million people worldwide^20^ offer one notable example. In filarial infections, antibiotics attacking the *Wolbachia* symbiont dramatically reduce or cure disease^21^. Recent studies show additional complexity in *Wolbachia*^19^,^22^,^23^ emphasizing that strategies to manage disease through manipulating *Wolbachia* endosymbionts will depend on a better understanding of not only the phenotypic effect on the host, from antagonistic to benevolent, but also the genetic and evolutionary shifts between mutualism and parasitism.

*Wolbachia* research has recently flourished with a growing set of genomic and transcriptomic contributions^16^,^24^,^25^,^26^,^27^,^28^, permitting a view of both phylogenomic relationships and genotype-to-phenotype hypotheses^7^,^29^,^30^. These analyses show that most known *Wolbachia* strains fall into two major sister groups. One group is comprised of mostly reproductive parasites, dominated by insect hosts, short evolutionary distances, frequent host-switching, genetic exchange between strains, and common co-infections (supergroups A and B). The second group is largely comprised of obligate mutualists, dominated by filarial nematode hosts, long evolutionary distances, with more limited host-switching, genetic exchange, and co-infections (supergroups C, D, and F)^18^,^31^,^32^,^33^. Research also points to genes and pathways that may be central to the second group, supplying essential nutrients to the host (e.g. riboflavin, biotin, thiamine, iron, etc.)^9^,^16^,^24^,^30^,^34^,^35^; however, presence of these genes does not always predict phenotype. A major challenge in understanding such patterns is the absence of closely related free-living outgroups. Even the nearest *Wolbachia* outgroups amongst the Rickettsiales (e.g., *Ehrlichia* and *Anaplasma*) are highly specialized pathogens, separated from *Wolbachia* with long branches^33^,^36^,^37^, making it difficult to resolve the ancestral state of this group or polarize the model of major transitions between parasitism and mutualism. A related long-standing challenge has been a lack of taxonomic sampling at the root of this clade. A decade ago, PCR surveys suggested the absence of *Wolbachia* in non-filarial nematodes^38^. Recently, however, *Wolbachia* was found in the Tylenchid plant-parasitic nematode *Radopholus similis*^39^. Until the present study phylogenomic analyses including plant-parasitic nematode-associated *Wolbachia* were not possible, since genomic data was lacking^37^,^40^.

The present study looks at evolutionary transitions and genetic features of *Wolbachia* first using phylogenomics that incorporate genome data for a new strain of *Wolbachia* from the root lesion nematode *Pratylenchus penetrans* (order Tylenchida). Plant-parasitic nematodes collectively cost $80 billion in agricultural crop loss annually worldwide^41^, with *Pratylenchus* spp. ranked as the third most economically-important group. *P. penetrans* is difficult to control due to its broad host range. For many high-value crop systems toxic fumigants are required to obtain economically viable yield. Here, as for insects and filarial nematodes, *Wolbachia* could provide an intriguing novel target for management of this serious nematode pest.

In this study, we confirm the presence of *Wolbachia* in nematode cells in *P. penetrans* using fluorescence *in situ* hybridization (FISH), ruling out false positives associated with contaminant DNA or horizontally transferred genome fragments in nematode nuclear DNA. Then, using comparative genome analyses, we explore changes in gene content across *Wolbachia*, investigating whether plant-parasitic nematode *Wolbachia* strains are more similar to mutualists or reproductive parasites. Lastly, we examine differences in gene content and metabolic capacity across the *Wolbachia* tree as related to previously hypothesized conserved functions, in particular, host riboflavin (vitamin B2) supplementation^7^ and host iron metabolism regulation^9^. Our results reveal a strain at the root of the *Wolbachia* phylogeny, helping piece together the long-standing puzzle of the success of the most widespread ecdysozoan endosymbiont^6^,^18^,^33^,^36^,^42^,^43^.

## Results

### Localization of *Wolbachia* in *P. penetrans* by fluorescence *in situ* hybridization (FISH)

**Figure 1:**
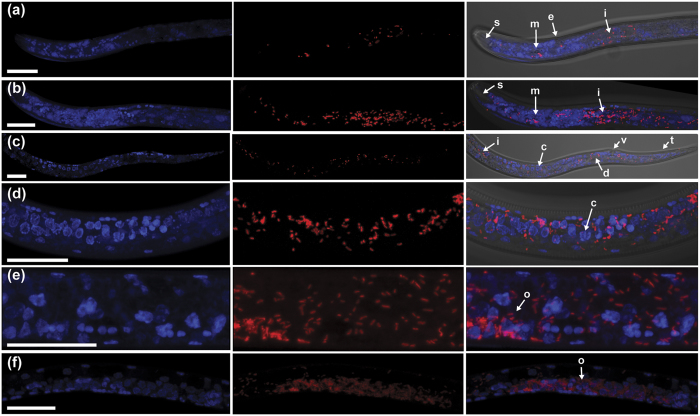
Localization of *Wolbachia* wPpe in *P. penetrans* by fluorescence *in situ* hybridization (FISH) using confocal microscopy. (**a**) Anterior region of adult female nematode showing DAPI stain alone (blue), *Wolbachia*-specific FISH probe (red), and combined light DIC, DAPI, and *Wolbachia* probe, on the left, middle, and right panels, respectively. s = stylet, m = median bulb, e = excretory pore, i = intestine (**b**) Same region as in **a** in combined z-stacks to reveal the density of *Wolbachia* (red) in this region. (**c**) Posterior region of adult female nematode. i = intestine, c = early oocyst within ovaries, d = developing egg, v = vulva, t = tail. (**d**) Ovaries containing developing oocysts. c = early oocyst. (**e**) Ovaries at higher magnification in combined z-stacks to reveal density of *Wolbachia* within and outside ovary. o = ovary. (**f**) Ovaries at lower magnification with densely packed *Wolbachia* cells. Scale bars = 20 μm.

We applied FISH confocal microscopy to confirm the presence of *Wolbachia* cells in *P. penetrans*. The *Wolbachia*-specific FISH probe localized coccoid to rod-shaped cells throughout the tissues of *P. penetrans* nematodes (Fig. 1a–f) in approximately half of the individuals examined (N = 60). Bacterial cells appeared less densely packed in the pharynx and head (Fig. 1a,b) and more densely packed from the anterior portion of the intestine to the tail (Fig. 1e–f). Bacterial cells were most dense in the ovaries where they were associated with oocytes and developing eggs adjacent to the vulva (Fig. 1d). Bacterial cells were more sparsely distributed in juveniles.

### Sequencing, assembly, and annotation of *Wolbachia* from *P. penetrans*

High-throughput sequencing was performed for *Wolbachia* from *P. penetrans*, followed by assembly along with other strains available in NCBI databases. Sequencing from *P. penetrans* produced about 19 million raw paired end reads (SRA accession SRR3097580) of 301 bp length with an average insert size of about 640 bp (Supplementary Table S1). The initial assembly produced many scaffolds with N50 of 5,531 bp and a total assembled length of about 350 Mbp. Removing non-*Wolbachia* hits and refining the assembly produced 12 scaffolds with an N50 of 95,550 bp and total length of 975,127 bp, with average coverage 16.7X (see Supplementary Table S1) with few Ns (0.13%) (Supplementary Fig. S1). Among these scaffolds were homologs to all the well-characterized marker genes for *Wolbachia*, including five MLST genes, several outer surface protein (*wsp*) homologs, and 16S rRNA gene with 96–97% sequence identity to other *Wolbachia* strains in GenBank, including those from the type host *Culex pipiens*, with the next closest 16S sequences being 85–89% similar (*Anaplasma*, *Ehrlichia*, and *Neorickettsia* spp.). This inter-strain 16S identity was similar to that between several other *Wolbachia* strains and type host *Wolbachia* (e.g. strain from *R. similis* 95–96%, strain from *B. tabaci* 96–97%, strain from *P. nigronervosa* 96–97%). Given these features and the FISH data above, this bacterium from *P. penetrans* was identified as *Wolbachia* and is hereafter denoted wPpe. Its 12 scaffolds serve as the draft genome for this strain (deposited at DDBJ/ENA/GenBank under accession number MJMG00000000 version MJMG01000000). This genome had 32.1% G + C, 962 predicted proteins, and a full set of rRNA and tRNA genes (3 and 34, respectively), with 86.6% of the genome coding and about 30% of predicted proteins having no known function. To add taxa for comparison, we obtained SRA data and assembled *Wolbachia* genomes from the banana aphid *Pentalonia nigronervosa* (denoted wPni) and from the springtail *Folsomia candida* (denoted wFol)^37^,^44^ (Supplementary Table S1). A wide array of other *Wolbachia* strains with completed or partially completed genomes are shown in Supplementary Table S2, along with details on host, supergroup, genome size, and predicted proteins.

### Phylogenomics show the earliest *Wolbachia* branches in plant-parasitic nematode hosts

**Figure 2:**
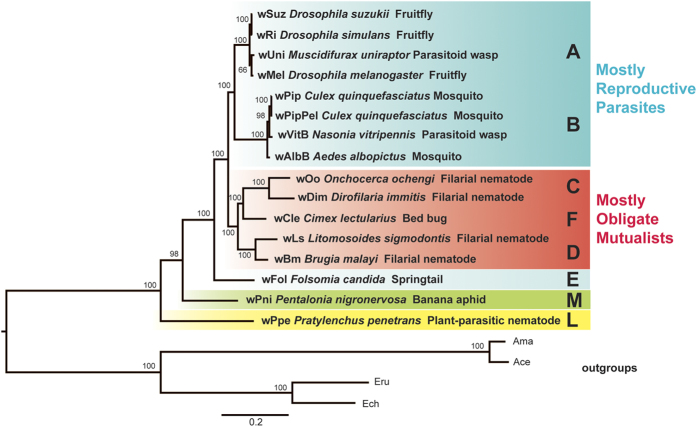
Maximum likelihood phylogeny of *Wolbachia* supergroups A, B, C, D, E, M and L, based on 79 conserved single-copy orthologous genes. The tree was generated from 61,465 nucleotide alignment positions with RAxML under the GTR model. Bootstrap values shown on branches are from 1,000 replicates. Shading indicates major groups. Outgroups are *Anaplasma centrale* str. *Israel* PRJNA42155, *Anaplasma marginale* str. *Florida* PRJNA58577, *Ehrlichia chaffeensis* str. *Arkansas* PRJNA57933, and *Ehrlichia ruminantium* str. *Gardel* PRJNA58245. Topology and majority support were consistent among numerous similar analyses that involved modifying nucleotides vs. amino acid data, alignment filtering stringency (Gblock), outgroups, model (GAMMA vs. CAT), or phylogeny inference method (ML vs. Bayesian).

Phylogenetic analyses consistently produced trees with topology and support similar to that shown in Fig. 2. This phylogeny was generated from orthologous protein-coding loci shown in previous studies to be single-copy in all *Wolbachia* strains and outgroups (*Ehrlichia* spp. and *Anaplasma* spp.) and exhibiting no recombination and no nucleotide saturation^37^,^40^ (Fig. 2). We could confidently align only 79 of 90 genes due to short contigs breaking within genes in the assembly of strain wPni. We further tested for recombination and saturation and found no evidence for recombination by the Phi test within *Wolbachia*, but evidence for recombination with outgroups included (Supplementary Table S3). NSS and Max χ^2^ tests within *Wolbachia* suggested possible mutational hotspots within *Wolbachia*^45^ (Supplementary Table S3). The Xia’s test showed no evidence of nucleotide saturation within this dataset (Supplementary Table S4). In virtually all ML phylogenetic analyses on all data sets with varying character filtering and recoding (Supplementary Figs S2–S8), *Wolbachia* wPpe formed the earliest branch (denoted supergroup “L” here as in ref. 4, equivalent to “I” in ref. 38) with *Wolbachia* wPni and *Wolbachia* wFol forming the second and third basal branches. The groups C + D + F and A + B and individual supergroups were also strongly supported. Group F formed a branch within the C + D group. This same well-supported topology was produced for both nucleotide and amino acid sequence datasets, for a variety of Gblock stringencies regardless of maximum likelihood parameters, or choice of outgroup (Supplementary Figs S2–S7). Results were similar for Bayesian inference using MrBayes (Supplementary Fig. S8). Analysis of 36 orthologs with two additional outgroups *Neorickettsia sennetsu* and *Candidatus* Xenolissoclinum pacificiensis produced a similar strongly supported tree (Supplementary Figs S9–S14) regardless of phylogenetic inference method, parameters, or outgroups.

Despite consistency in topology using multiple data sets and tree reconstruction parameters and strong bootstrap and posterior probability support, these results could be affected by artifacts arising from different evolutionary histories and long branch lengths to outgroups. For example, *Anaplasma* species had higher %GC (~49.5%) and longer branch compared with *Ehrlichia* species. Hence, we applied the CAT-Poisson and CAT + GTR models in PhyloBayes, and found support for all nodes with outgroups excluded (Supplementary Fig. S15), but with any number of outgroups included there was an overall decrease in support for major in-groups and root positions (Supplementary Table S5) regardless of choice of outgroup or dataset. In some of these, wPpe and wPni formed a sister clade at the root of the *Wolbachia* tree, however, no analyses supported a branch position for wPpe at a later node relative to the root. Notably, in two of these analyses with *Anaplasma* species removed, wPpe grouped basally along with supergroup C strains (Supplementary Table S5).

To further examine alternate root topologies, we used the Approximately Unbiased (AU) test to evaluate the best unconstrained tree, which placed wPpe as the earliest root branch of *Wolbachia*, against various alternate roots, and results showed all other topologies were rejected (p-values < 0.005) (Supplementary Table S6).

**Figure 3:**
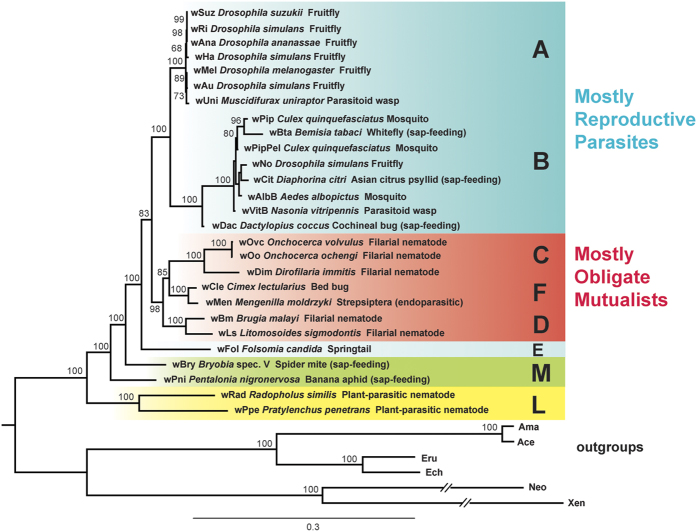
Maximum likelihood phylogeny of *Wolbachia* supergroups based on 16S ribosomal RNA, ftsZ and groEL genes. The tree was generated from 4,307 nucleotide alignment positions, with the GTR model under RAxML. Bootstrap values on branches result from 1,000 replicates. For strain accessions, see Supplementary Table S2 and S7. Color scheme and outgroups match those in Fig. 2, with the addition of Neo = *Neorickettsia sennetsu* PRJNA357 and Xen = *Candidatus* Xenolissoclinum pacificiensis PRJNA219341. Topology and support were consistent among analyses as described in Fig. 2.

To include the *Wolbachia* strain from the plant-parasitic nematode, *Radopholus similis*, (hereafter denoted wRad), we also reconstructed phylogenies for the three genes that were available from wRad (16S rRNA, *ftsZ*, and *groEL*). This analysis included 5 additional *Wolbachia* strains (Supplementary Table S7). The result was a strongly supported tree identical with that from previous larger datasets of 79 and 36 orthologs, with wPpe and wRad as sisters forming the earliest branch, followed by wPni, wBry from the mite *Bryobia sp.*, and wFol (Fig. 3). Additional analyses with varying alignment stringency (Gblocks), ML parameters, outgroup choices, and using MrBayes produced similar results (Supplementary Figs S16–S17). Single loci phylogenies (n > 100 strains) placed wPpe in the most basal position in the tree (Supplementary Figs S18–S21), with wPni, wBry and sometimes wBta from the whitefly *Bemisia tabaci* forming the next branches. CAT and CAT-GTR analyses produced trees with lower support, characterized by polytomies (Supplementary Table S5).

### Genome wide trends and gene content overlap between *Wolbachia*

**Figure 4:**
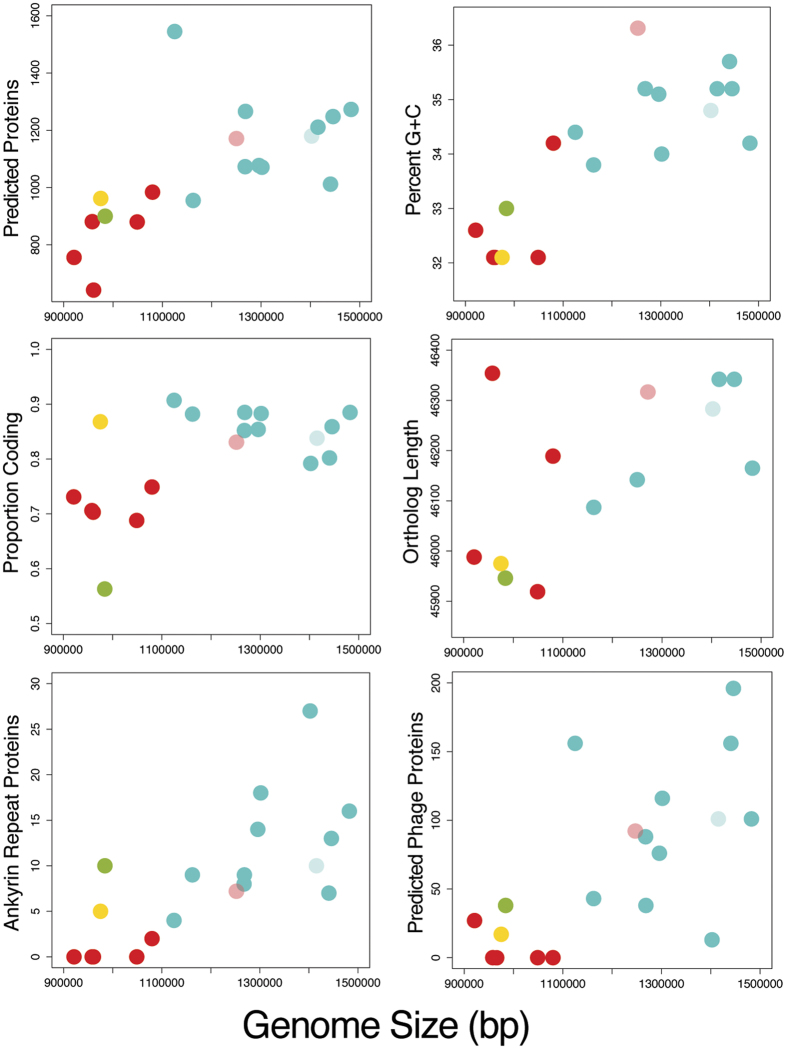
Various genomic features of *Wolbachia* strains depicting positive trends with increasing genome size for number of proteins, G + C content, proportion of the genome that is coding, total length of 79 concatenated orthologous genes, number of ankyrin repeats and number of predicted phage-like genes. Dots represent *Wolbachia* strains, with color scheme: yellow = group L (*Wolbachia* from *P. penetrans*, wPpe), green = group M, red = groups C + D, pink = group F, blue = groups A + B, light blue = group E.

To examine genomic similarity between wPpe and other *Wolbachia* strains, first, we compared overall genome properties (Fig. 4) with genome size. Strains wPpe (supergroup L) and wPni (supergroup M) were nested near the smaller end of the spectrum of genome size next to supergroup C strains, which had the smallest genomes. Strains with smaller genomes tended to have fewer proteins, lower G + C, a lower proportion of coding sequence, shorter protein (ortholog) length, fewer ankyrin repeats (33-residue motif alpha-helix proteins hypothesized to be involved in *Wolbachia*-host protein-protein interactions) and fewer predicted prophage or phage-like proteins (thought to be associated with *Wolbachia* phenotypes, like cytoplasmic incompatibility)^26^,^29^,^46^,^47^. Colors in Fig. 4 highlight the major groups, and show a positive trend with genome size for most features with a few exceptions. In particular, wPpe fits the trend except for proportion coding and wPni has more predicted ankyrins and quite low proportion coding. One *Wolbachia* strain, wOo from the filarial nematode *Onchocerca ochengi*, had notably longer ortholog lengths than others with similar genome sizes, and strain wDac, from the cochineal scale insect, was exceptional in having more predicted proteins and ankyrins than other *Wolbachia* strains with similar genome sizes. The wDac assembly also displayed more duplicated genes, including genes that are usually single-copy in *Wolbachia*, suggesting possible assembly artifacts resulting from mixed strains. Mutualist strain wCle, from the bedbug *Cimex lectularis*, had genome features fitting with the trends based on genome size rather than phenotype, i.e. it did not cluster with other mutualists from filarial nematodes. Genome-wide average amino acid identities (AAI) were as low as 66.2% between distantly related pairs (Supplementary Table S8; ANI values were in the range of 72–79%, considered too low to be reliable^48^), and AAI frequency distributions showed large overlap (Supplementary Fig. S22).

**Figure 5:**
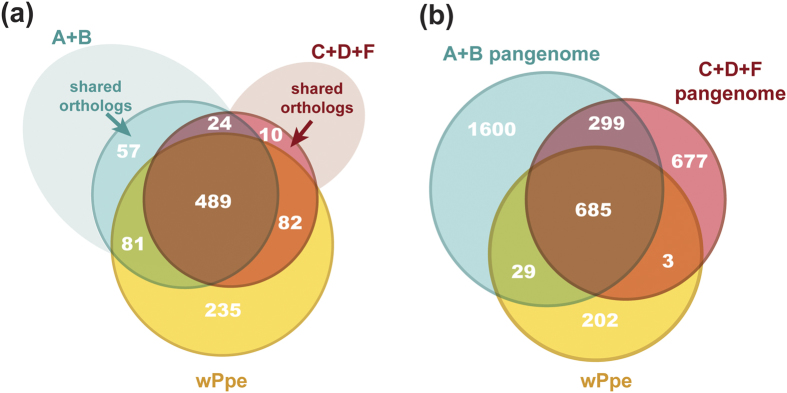
Gene content similarity between major *Wolbachia* groups. (**a**) Venn diagram of gene set overlap between groups A + B (blue) and C + D + F (red), where solid shading represents universally retained orthologous genes in each group and faint shading represents singletons and orthologs not universally shared. Yellow = gene set from *Wolbachia* from *Pratylenchus penetrans* (wPpe). Blended colors represent overlaps (purple = A + B + C + D + F, orange = C + D + F + wPpe, green = A + B + wPpe, brown = A + B + C + D + F + wPpe). (**b**) Venn diagram of the same groups in **a**, using the same color scheme, comparing the entire pangenome of each group only for genes with an assigned function.

**Figure 6:**
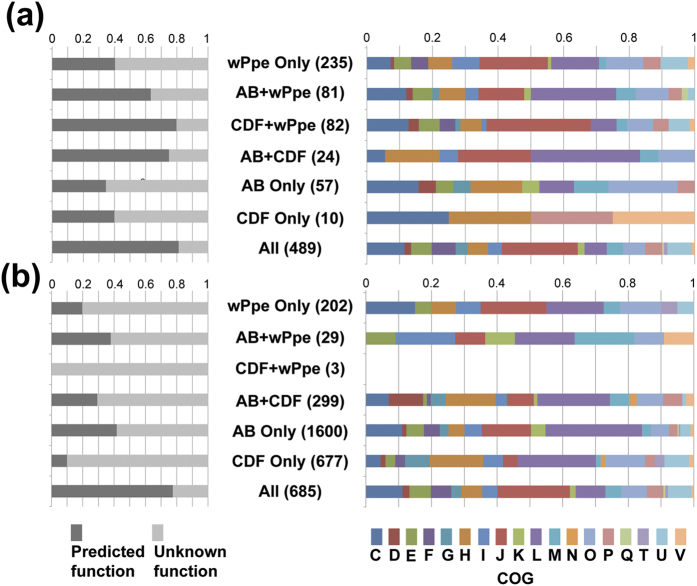
General gene functional classification for groups shown in Venn diagrams (Fig. 5) showing proportions of genes in each group sharing each function. (**a**) Core genome data for orthologs universally shared in AB, in CDF, and in overlaps between these with wPpe. (**b**) Pangenomes A + B and C + D + F and overlaps with wPpe. COG = categories of orthologous genes: C = Energy production and conversion, D = Cell cycle control, cell division, chromosome partitioning, E = Amino acid transport and metabolism, F = Nucleotide transport and metabolism, G = Carbohydrate transport and metabolism, H = Coenzyme transport and metabolism, I = Lipid transport and metabolism, J = Translation, ribosomal structure and biogenesis, K = Transcription, L = Replication, recombination and repair, M = Cell wall/membrane/envelope biogenesis, N = Cell motility, O = Posttranslational modification, protein turnover, chaperones, P = Inorganic ion transport and metabolism, Q = Secondary metabolites biosynthesis, transport and catabolism, T = Signal transduction mechanisms, U = Intracellular trafficking, secretion, and vesicular transport, V = Defense mechanisms.

Since wPpe formed the basal branch in our phylogenetic analyses, we investigated to what extent its gene content resembled each of the two large sister groups (A + B and C + D + F) by ortholog analysis (Fig. 5). First we compared universally shared orthologs, denoted “core genome”, from each of these two large groups, A + B and C + D + F (Fig. 5a). Slightly more orthologs were universally shared in A + B than in C + D + F (651 versus 605, respectively), with most of these (489) shared across all *Wolbachia* strains, including wPpe. About 20% of the universal core *Wolbachia* genome was comprised of uncharacterized genes with no match to known proteins, while the remaining core genome displayed a wide range of predicted functions (Fig. 6, Supplementary Table S9). Amongst the 235 genes in wPpe that were not universally present in A + B or C + D + F, 60% were uncharacterized, and the remainder had a functional profile, in terms of COG categories, that was similar to the core *Wolbachia* genome (Fig. 6). Nearly equal numbers of wPpe genes were shared with core A + B and core C + D + F genomes (81 and 82 genes, respectively), with the 81 genes shared between wPpe and A + B overlapping in a large proportion of genes for replication, recombination and repair (25%) while the 82 genes shared between wPpe and C + D + F had a large number of genes for translation, ribosomal structure and biogenesis (30%). There were just 24 core *Wolbachia* genes without orthologs in wPpe, consisting of a range of functions, with ~17% for coenzyme transport and metabolism (COG H). This functional group was also abundant in both the A + B core genes not shared with other *Wolbachia* groups (57 genes), and the C + D + F core genes not shared with other groups (10 genes).

Next, we analyzed the total gene sets for A + B and C + D + F, or “pangenome” for these groups to assess differences in total genetic repertoire (Figs 5b and 6, and Supplementary Table S9). The overlap in pangenomes between all *Wolbachia* groups and wPpe were similar to that of the core genomes, with 685 genes with 20% of these uncharacterized. These genes had a similar functional profile to the core genome. This analysis revealed higher proportions of uncharacterized genes for other overlapping groups (>60%), and otherwise, similar functional differences to that described above for core genomes.

A number of metabolic genes were found in wPpe that were not universally conserved in the core genomes A + B and C + D + F (Fig. 5, see Supplementary Table S9). Some of these were exclusively found in wPpe (i.e. not in the pangenomes of A + B or C + D + F), including *asd2*, *hemC*, *glyA*, *glnA*, *fabF*, *nfo*, *rmuC*, *ruvA*, and apocarotenoid-15,15-oxygenase. Five genes from the latter list (*asd2*, *hemC*, *glyA*, *fabF*, *rmuC*) represent highly diverged second copies of these normally single-copy genes. Amongst biosynthetic genes shared between wPpe and A + B were amino acid (*metK, argD, aspC*), B vitamin (*pdxJ, fgs*), terpenoids (*ispA*), and biotin transport (*bioY*) genes. Overlapping pangenomes of wPpe and C + D + F included genes for synthesis of amino acids (*gltA, proP, iscS, dapA, gltB, adiC*) and vitamins/cofactors (*coaE, coaD, hemE*). One out of four *Wolbachia* surface protein (*wsp*) family genes appeared to be missing in wPpe and wBm from *Brugia malayi* (Supplementary Table S10).

Among the genes shared across all *Wolbachia* strains, including wPpe, several were noteworthy, including numerous transposases, two competence genes (*comEC, comM*), a single riboflavin synthesis gene (*ribB*), and most of the 52 genes integral to iron metabolism (Supplementary Table S11). An analysis of gene-by-gene substitution rates (Ka) in these iron metabolism genes, comparing wPpe vs. wBm (*Wolbachia* from *Brugia malayi*) and wPpe vs. wAlbB (*Wolbachia* from *Aedes albopictus*), showed little variance between strains (Supplementary Table S11, and Supplementary Fig. S23). Several iron-related genes displayed low substitution rates (especially rhodocoxin, most NADH-quinone oxidoreductases, cytochrome c oxidases, and *nifU*), while the heme exporter protein B showed a high substitution rate. Several iron metabolism genes with partially described functions exhibited large variance in Ka between strains (Supplementary Fig. S23).

**Figure 7:**
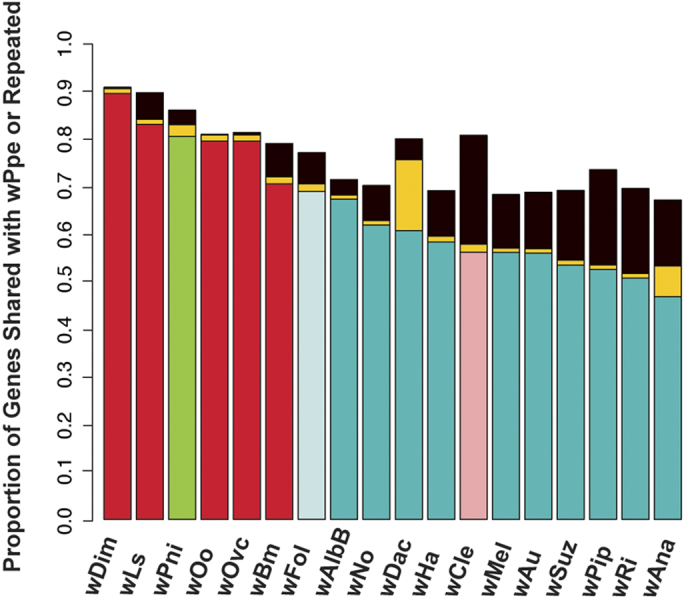
Gene repertoire similarity between several *Wolbachia* strains and *Wolbachia* from *Pratylenchus penetrans* (wPpe). For example, in strain wDim from *Dirofilaria immitis*, 90% of its genes have an ortholog in wPpe, whereas for strain wAna in *Drosophila ananassae*, 45% of its genes have orthologs in wPpe. Color scheme matches that in Fig. 4, except here yellow represents repetitive elements with orthologs in wPpe, and black indicates repetitive elements with no ortholog in wPpe.

To assess strain-specific gene repertoire similarity, we analyzed the proportion of each strain’s genes that had orthologs in wPpe (Fig. 7). wDim from the filarial nematode *Dirofilaria immitis* and wLs from the filarial nematode *Litomosoides sigmodontis* were most similar in gene repertoire to wPpe. The next most similar was wPni from the aphid *P. nigronervosa*, followed by the remaining three strains from filarial nematodes. Strain wCle from the bedbug *Cimex lectularius* shared fewer genes with wPpe than several members of the A + B, while the remaining strains in group A + B shared the lowest proportion of their genes with wPpe (Fig. 7). Repetitive elements appeared to have an inverse relationship in similarity to wPpe.

**Figure 8:**
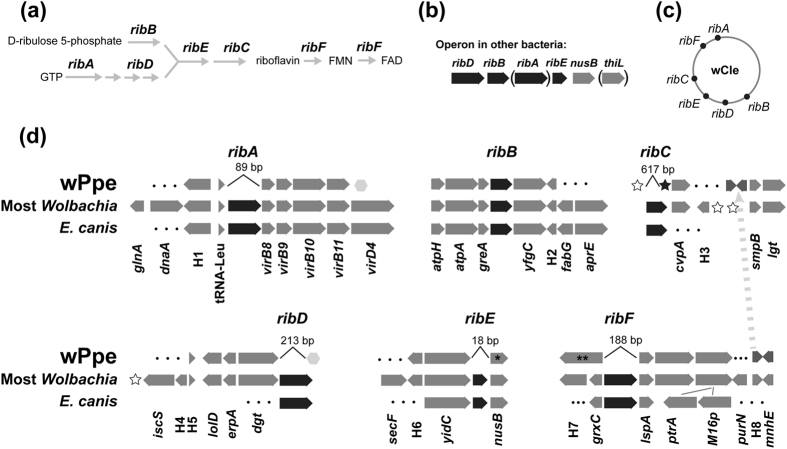
Predicted riboflavin synthesis capacity compared between *Wolbachia* from *Pratylenchus penetrans* (wPpe), most other *Wolbachia* strains, and outgroups. (**a**) General riboflavin biosynthesis pathway. (**b**) Riboflavin operon found in most bacteria. Parentheses = genes found outside the operon in alphaproteobacteria. (**c**) Riboflavin gene dispersal in a typical *Wolbachia* strain (wCle). Other strains vary in gene order and location. (**d**) Flanking regions for riboflavin synthesis genes, five of which were missing in wPpe. Grey block arrows = flanking genes, black block arrows = riboflavin synthesis genes. Numbers above missing riboflavin genes show length of intergenic sequence in wPpe. Dashed grey line shows genes H8 and *mnhE* normally near *ribF* in an alternate location in wPpe next to genes normally associated with *ribC* in other *Wolbachia*. Dots indicate cases where orthologs are located elsewhere in the genome. Open stars = *Wolbachia* transposases, black star = Bacteroidetes-like transposase, light grey hexagons = ankyrin repeat proteins, single asterisk = partial gene, double-asterisk = fused *hyPrx5* gene with *grxC*-like domain. H1 calcineurin-like phosphoesterase; H2 TrbC/VirB2 family protein; H4 alpha/beta hydrolase family protein; H6 retroviral aspartyl protease, H3, H5, and H7-H8 uncharacterized.

The wPpe draft genome presented here appeared to be missing some genes that are widely distributed in *Wolbachia*. For example, Fig. 5a shows 24 genes universally found in A + B + C + D + F *Wolbachia*, but not in wPpe. These included genes for iron-cluster assembly (*iscA*), riboflavin synthesis (*ribH1*), puromycin synthesis (*miaB*), and DNA repair (*uvrB, uvrC*). Five out of six riboflavin synthesis genes that are nearly universal in *Wolbachia* were apparently missing from wPpe (*ribA, ribC, ribD, ribE, ribF*). Since missing genes could result from technical artifacts (e.g. inconsistent coverage, contig breaks, disrupted by Ns, or filtered out with small contigs), we aligned flanking regions from diverged *Wolbachia* strains. Flanking gene order is often conserved, e.g. for riboflavin genes^7^. For wPpe, riboflavin synthesis gene flanking regions were conserved (Fig. 8a–d) in order and orientation. Nevertheless, all riboflavin synthesis genes except *ribB* were absent from wPpe. The intergenic spaces between missing genes ranged from 18 to 617 bp, and had no significant hits to databases in blastn and blastx searches. The phylogeny of *ribB* was complex, consistent with partial non-vertical transmission as in previous studies^7^ (Supplementary Fig. S23).

**Figure 9:**
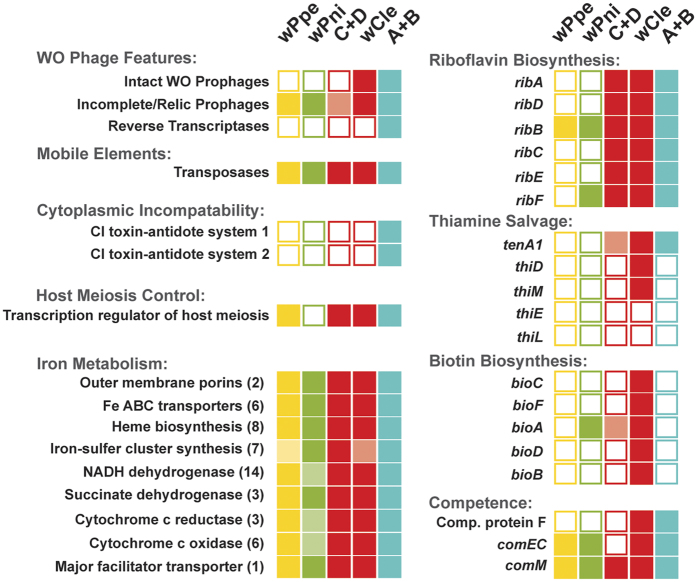
Summary of selected genetic features amongst wPpe and other *Wolbachia* strains, including those previously reported as important in the success of *Wolbachia*. Comparison between wPpe (yellow), wPni (green), mutualist group C + D (red), mutualist wCle from *Cimex lectularis* from group F (red), and mostly reproductive manipulators A + B (blue). Solid squares = homologous gene(s) are present, empty squares = homologous gene(s) are absent, lightly shaded squares = one or more genes absent from a gene set, or gene is only present in one or two strains from a group. Numbers in parentheses represent numbers of genes (listed in Supplementary Table S11).

While the assessment of other missing genes from this draft genome in wPpe remains tentative, awaiting a completed genome, we note that we found no evidence for homologs of the proposed toxin-antidote cytoplasmic incompatibility genes WP_0282/0283 and WP_0292/0293, and no evidence for the horizontally transferred biotin and thiamine synthesis operons found in wCle (Fig. 9).

## Discussion

Here we analyzed the first *Wolbachia* genome from a plant-parasitic nematode to help understand evolutionary patterns in this globally distributed genus, with members that are important targets for controlling diseases like malaria, dengue, and filariasis. Ribosomal rRNA 16S similarity and presence of homologs to all *Wolbachia* marker genes clearly place this bacteria from *P. penetrans* within the genus *Wolbachia*^49^,^50^,^51^, while phylogenomic results placed it at the base of the tree, suggesting that plant-parasitic nematodes were the first hosts for *Wolbachia*. Prior to a recent study showing *Wolbachia* in the plant-parasitic nematode *Radopholus similis*^39^, this result was not predicted^38^. Our findings, combined with other recent studies^4^,^44^,^52^,^53^ change the view of mutualism evolution in *Wolbachia*. Previously, evidence suggested that obligate mutualism evolved once in association with the transition to filarial nematode hosts^19^,^36^,^37^,^42^ and recent work suggested this could be the ancestral condition^33^ based on group C being the earliest-diverging clade. The present study suggests an earlier transition to mutualism before the transition to filarial nematode hosts, given the early place of the mutualist wPni, with later loss or gain in groups E and F^30^,^33^,^52^. Another perspective on these ubiquitous endosymbionts is that mutualism arose through horizontal gene transfer (HGT) from other endosymbionts^30^. While this appears to be true for strain wCle, which possesses largely intact non-*Wolbachia* operons for biotin and thiamin synthesis, this phenomenon does not appear to be widespread in *Wolbachia*. In contrast, the present study resolves past uncertainty about the place of the next branch, group E (represented here by wFol) in Collembola^37^,^54^,^55^. This soil-dwelling host appears to obligately depend on its *Wolbachia* for survival, suggesting mutualism is ancestral to group A + B and C + D + F. This would imply A + B reproductive parasite strains may have lost their beneficial effect. Thus, the major question becomes not only how obligate mutualism arose in filarial hosts, but whether (and how) it may have been lost in arthropod hosts.

Furthermore, our result showing a basal place for wPpe within *Wolbachia* indicates that this endosymbiont has invaded nematodes at least twice, implying that this endosymbiont so prevalent in arthropods, occurring in perhaps 66% of species^1^, was initially adapted to nematodes, as was suggested from earlier analyses on another nematode clade^33^. *Wolbachia* from plant-feeding specialist hosts formed the dominant basal branches of the trees in this study, presenting a new picture of the early ecological context in which *Wolbachia* may have arisen, contrasting with previous views^37^,^40^,^49^. Our result is consistent with recent analyses from several loci showing *Wolbachia* from sap-feeding hosts (e.g., aphids, sap-feeding spider mites, and whiteflies) generally emerged early in the *Wolbachia* tree^4^,^39^,^53^,^56^, although it is also noteworthy that *Wolbachia* appear to have re-invaded sap-feeding hosts later in the tree. Nevertheless, the dominance of plant diets at the root of the tree presents the hypothesis that *Wolbachia* evolved early as a supplier of nutrients missing in these host plant-juice diets. Most sap-specialists require one or more nutritional symbionts (e.g. *Buchnera* in aphids, *Portiera* in whiteflies, and *Cardinium* in spider mites), and dual endosymbioses, requiring cooperation between pairs of endosymbiont species, appears to be the rule rather than the exception in these systems^57^. A recent study demonstrated co-dependent co-obligatory nutritional symbiosis between *Wolbachia* and *Buchnera* in the banana aphid^44^.

Comparative genome analyses largely allied wPpe with mutualist *Wolbachia* strains from filarial nematodes. For example, in genome size, predicted proteins, proportion G + C, ortholog length, ankyrin repeats, and phage-like proteins, wPpe resembled groups C and D. However, we interpret this cautiously since theory predicts that both accumulation of A + T bias and genome streamlining will arise in any lineage exposed to sufficient vertical transmission and bottleneck. This is seen in a wide range of bacteria where the duration, degree, and type of host association appear to influence these genome features^57^,^58^,^59^,^60^. In this context, the genome features and gene content for group F (represented by wCle in Figs 4 and 7) are consistent with more recent acquisition of the mutualist lifestyle and vertical transmission mode in this strain, presumably through a change such as gain of B vitamin genes^30^ and host change^33^. However, gene repertoire analyses for single strains further support the association of the strains in plant-parasitic and filarial nematodes, particularly group C, with wPpe being the most similar to wDim. This result would seem to be consistent with the recent analyses pointing to group C as one of the earliest-branching clades of *Wolbachia*. In contrast, core and pangenome analyses presented a less obvious alliance between wPpe and filarial nematode mutualists, pointing instead to an intermediate state in terms of gene content between wPpe, mutualists, and reproductive manipulators.

What is the nature of the symbiosis between *Wolbachia* wPpe and its host nematode? Although our data show patterns in gene repertoire and overall genome features, it does not fully answer this question. Sex ratio distortion is a possibility to be further explored, but males are widely observed and thought to be required for reproduction in this nematode^61^. There is also so far, no clear genomic evidence for reproductive parasitism in wPpe; we found no homologs of intact WO phage genes or “toxin-antidote” genes WP_0282/0283 and WP_0292/0293 previously implicated in cytoplasmic incompatibility^26^,^29^,^62^. On the other hand, the dispersed tissue distribution of wPpe in our FISH analyses, which was similar to that of wRad^38^, more closely resembles the tissue distribution pattern found in reproductive manipulator *Wolbachia* strains. Conversely, most obligate mutualist *Wolbachia* strains are concentrated in specialized tissues like the syncytial lateral cord or paired bacteriomes and are found at 100% prevalence^6^,^17^, whereas wPpe appeared not to occur at 100% prevalence. However, the observed dense packing of wPpe in the anterior gut wall could indicate a possible beneficial association and is consistent with *Wolbachia* distribution in at least one filarial nematode, *Mansonella perforata*^32^.

Of the several types of nutritional supplementation proposed for *Wolbachia* including riboflavin, thiamine, biotin, and heme^7^,^9^,^16^,^17^, only heme synthesis would appear likely in wPpe, given the apparent absence of most genes for these other pathways. However, we note that because the wPpe genome is still in draft form, it is possible that some or all of the genes were absent due to technical artifacts. The strain sharing the greatest proportion of its genes with wPpe was wDim, which has been shown to synthesize heme in a stage-specific manner in synchrony with host nematode heme-binding proteins^27^. In light of the observation that iron and heme are often limiting in plant roots^63^ and must be *de novo* synthesized by nematodes, our data may further support the iron hypothesis^9^, raising the question of how *Wolbachia* may have contributed to iron/heme acquisition during the transition to a root-endoparasitic lifestyle of its nematode host.

While plant-parasitic nematode lineages appeared to form the earliest branches in this study, long branch attraction (LBA) could be an issue^36^. Absence of nucleotide saturation and consistent placement of wPpe in a basal or root-polytomy position in CAT + GTR Bayesian analyses provides some measure of confidence in the root, despite LBA^64^. Still, low node support, polytomies, outgroup recombination, and GC bias suggest the outgroups are not ideal. Nevertheless, when outgroup strains were removed CAT + GTR produced highly supported topologies matching those of other evolutionary models, with plant-parasitic nematode strains wPpe and wRad as sisters, despite long branches. These deep relatives suggest a long history in the nematodes, which is consistent with the presence of widespread ancient HGTs from *Wolbachia* to ecdysozoans^65^,^66^,^67^,^68^,^69^,^70^ and possible association with early colonization of land, a scenario that could be tested with further taxon sampling^71^. Our discovery of sequence divergence in wPpe suggests some *Wolbachia* PCR primers may have mismatches at priming sites, resulting in possible false negatives in past surveys. Furthermore, as shown in the present study for wPpe and wBm, PCR survey targets like *wsp* that are present in multi-gene families may become lost during reductive genome evolution, resulting in potential underestimation of *Wolbachia* prevalence.

In conclusion, our analyses of strain wPpe from *P. penetrans* establish that *Wolbachia* may have originated in plant-parasitic nematodes, thereafter adapting to other plant-specialist hosts, and later re-invading nematodes. Genomic and tissue distribution features of wPpe suggest affinities with mutualist and reproductive manipulator strains, but no evidence was found for cytoplasmic incompatibility or B-vitamin supplementation. In contrast, iron metabolism and heme synthesis appeared to be highly conserved. These, findings provide a context for understanding symbiotic transitions in these widespread and important intracellular bacteria. Currently *P. penetrans* cannot be easily maintained and manipulated to allow antibiotic symbiont-clearing and tests for fitness effects on *Wolbachia*, thus, while our data could not explicitly predict the phenotype of strain wPpe, they provide candidate pathways and genes of interest for further study.

## Methods

### Nematode sample collection

Nematodes identified morphologically as *Pratylenchus penetrans* were collected from field populations of cultivated raspberry (*Rubus idaeus*) in Washington, USA, transferred to mint (*Mentha* sp.), and grown in the greenhouse at the USDA-ARS in Corvallis, OR. Nematodes were extracted from roots. For FISH, 60 nematodes of mixed stages (adult females and juveniles) were transferred to water from the greenhouse population. For genome sequencing, approximately 14,700 nematodes were isolated and ground for 2 min with a motorized micropestle to disrupt the cuticles before DNA was isolated using Qiagen DNeasy Blood & Tissue Kit (Valencia, CA).

### Fluorescence *in situ* hybridization and confocal microscopy

FISH was performed following an established protocol^60^ with the probe ATTO 633 (red) 5′-TGA AAT CCG GCC GAA CCG AC-3′ designed (this study), which was based on the wMelPop probe W1^72^, and was similar in sequence, but shifted two bases downstream and extended by one base at the 3′ end. This probe targets the 16S rRNA of *Wolbachia* strains while having several mismatches to other bacteria, including sister alpha-proteobacteria such as known species of *Rickettsia*, *Anaplasma*, and *Ehrlichia*. While the original W1 probe would have matched the *Wolbachia* sequence from *P. penetrans*, it would have had 1 bp mismatch from the *R. similis Wolbachia* strain. The slight shift of position was chosen based on matches of the probe to the widest-possible range of *Wolbachia* strains, thereby reducing the likelihood of false-negatives, in case of rare point mutations in the 16S targets in our natural wPpe population. Conditions and reagents were identical to those described previously^60^, except for a decrease in formamide to 35% vol/vol to increase specificity of hybridization. Specimens were viewed on a Zeiss LSM 780 NLO Confocal Microscope at the Center for Genome Research and Bioinformatics (CGRB; Oregon State University, Corvallis, OR). Negative controls were prepared as above using the same steps for specimens without *Wolbachia*, to check for non-specific binding of the probe, and also without adding the probe, to check for autofluorescence.

### DNA library preparation and genome sequencing

After initial DNA shearing for 50 s using a Diagenode Bioruptor Pico (Denville, NJ) to obtain peak library fragment sizes of ~600–700 bp, genomic libraries were prepared using the Illumina TruSeq DNA Sample Preparation Kit (San Diego, CA) following the manufacturer’s instructions. Adapter-ligated targets of ~650–750 bp were gel-excised and sequencing was performed using the Illumina MiSeq system for 2 × 301 bp reads (paired-end) at the CGRB.

### Genome assembly and annotation

Illumina reads were trimmed and quality filtered using FASTX-Toolkit v.0.014 (http://hannonlab.csht.edu/fastx_toolkit/) and the genome was assembled using Velvet v.1.2.10^73^. After an initial assembly, contigs were subjected to BLAST+ v.2.2.29 (NCBI; National Center for Biotechnology Information) searches to a complete database of reference *Wolbachia* genomes. Based on these results, assemblies were repeated to optimize parameters (kmers, average coverage, and coverage cutoff) to improve the assembly for *Wolbachia*. For a range of kmers, all assembly scaffolds with BLAST (blastn evalue 1, minimum hit length 45 bp) similarity to *Wolbachia* were extended to fill regions with “N“s using GapFiller v.1-11^74^. Resulting scaffolds were aligned with Mauve 2.3.1 multiple sequence aligner^75^ to select representative long scaffolds with the fewest Ns from different kmers, and finally the assembly was inspected by mapping reads back to scaffolds in bwa and SAMtools^76^,^77^ to check for regions with unpaired reads suggestive of assembly errors. The final assembly was searched again with BLAST nr to check any misclassified scaffolds whose top hits did not match *Wolbachia*. For assembly of strains wFol and wPni, reads from the NCBI Sequence Read Archive (SRA) database were downloaded, quality filtered, and assembled using CLC Genomics Workbench (CLC Bio, Aurhus, Denmark). *Wolbachia* matches were then extracted and inspected as described above. While these steps allow little chance of losing genuine *Wolbachia* regions, it is possible that some of the target genomes could be lost, due to short contigs, large genetic distances to reference database sequences, and the presence of repetitive elements. Nevertheless, a majority of analyses in this study should not be biased significantly by a small amount of missing data (e.g. any analysis involving %GC, proportion coding, gene order within scaffolds, and sequence content for any genes found). Furthermore, we present in Supplementary Fig. S1, the low number and size of N blocks that point to a limited likelihood of missing data.

For consistency in subsequent comparative analyses, annotation was performed for all strains in this study using the Prokka package v1.10^78^, which combines BioPerl and Prodigal for *ab initio* gene prediction, HMMER3 for protein family profiles, BLAST+ for comparative annotation, Barrnap for rRNAs, Aragorn for tRNAs. Phage-like proteins and regions were also identified using PHAST^79^.

### Ortholog identification, phylogenomic and comparative genomic analyses

For phylogenomic analyses, we began with an alignment of 90 single-copy orthologous proteins identified previously^40^ as being in only one-copy in *Wolbachia* and outgroups, and having no recombination or nucleotide substitution saturation^37^. To this set of aligned genes, we added sequences for four more taxa: *Wolbachia* strain wCle whose genome was downloaded from NCBI GenBank, and strains assembled and annotated in the present study, wPpe, wFol and wPni. Homologous genes were easily found by BLAST and aligned in Geneious v.5.4.4 (created by Biomatters), except for wPni, for which some genes were not found or were only partial in the assembly. We also used BLAST to search for homologs from two more outgroups, *Neorickettsia sennetsu* and *Candidatus* Xenolissoclinum pacificiensis, but due to the distance between taxa, some orthologs could not be found or aligned confidently. Additional gene sets were downloaded from NCBI to include more taxa for smaller sets of genes (16S rRNA alone, ftsZ alone, and 16S rRNA + ftsZ + groEL concatenated). Initial translation-guided alignments were performed for nucleotide and protein sequences for each gene, then gene sets were concatenated into a supermatrix. Alignments were further refined by removing ambiguous positions and masking in Gblocks 0.91b^80^. Resulting aligned supermatrices were tested for the presence of recombination using the pairwise homoplasy index (PHI) and other statistics calculated with PhiPack^45^ with a window of 200 bp and 1,000 permutations both with and without outgroups and groups A + B. This alignment was also tested for nucleotide saturation by using Xia’s test in DAMBE v6.4.20^81^. Supermatrices were prepared and analyzed with a variety of alternate parameters to test the robustness of the phylogenetic signal and test for biases due to different evolutionary histories, including: using two alignment filtration (Gblock) stringencies, modifying nucleotide data by eliminating 3^rd^ codon positions and using RY coding, eliminating one or more outgroup taxa.

Maximum likelihood (ML) trees were reconstructed using RAxML-HPC2 v.8.0.24^82^ and Bayesian trees were reconstructed using MrBayes v3.2.6-svn run on XSEDE (CIPRES Science Gateway V 3.1). ML analysis of nucleotide alignments was performed under the GTR model with empirical base frequencies and likelihoods evaluated under the GAMMA model with free parameters estimated by RAxML, and 1,000 bootstrap replicates. ML analysis of protein sequences was performed with the PROTCATDAYHOFF substitution model with empirical base frequencies and 1,000 bootstrap replicates. Bayesian analysis was performed with the GTR + I + G model for 1,000,000 generations sampled every 500 generations, with 2 runs of 4 chains, with default priors and a burnin of 25%. Because of the long branch lengths between *Wolbachia* strains and available outgroups, additional tests were performed to explore the effects of long branch attraction or other biases and alternate root positions. Bayesian inference using the CAT and CAT + GTR infinite mixture models was performed in PhyloBayes v3.2e^64^ to better account for possible site-specific amino-acid or nucleotide differences, particularly among *Wolbachia* and outgroups. PhyloBayes was run with two chains > 10,000 cycles, optimizing convergence points and burnin sizes as recommended using bpcomp and tracecomp^64^. Alternative rooting was evaluated using methods reported previously^37^ by testing alternately constrained tree topologies against the unconstrained tree using the AU test in CONSEL v0.02^83^ for trees generated using PhyML v3.1^84^ under the GTR (nucleotide) and Dayhoff (amino acid) substitution models, with gamma distribution of 4 rate categories.

For remaining comparative genomic analyses, orthologs were identified using OrthoMCL^85^ (inflation value 1.5 and 60% match cutoff and evalue of 1e-3). Clusters of orthologous groups of proteins (COGs), other functional details, and pathways were mapped to genes using a range of online databases (MetaCyc, KEGG pathways, UniProtKB, and EMBL-EBI InterProt). Amino acid substitution rate analysis was calculated in KaKs_Calculator^86^. Genome-wide average nucleotide identity (ANI) and average amino acid identity (AAI) analyses were performed for pairs of strains, using ANI and AAI Calculator tools in the enveomics package^87^.

## Additional Information

**Accession codes:** The annotated draft genome of *Wolbachia* wPpe from *Pratylenchus penetrans* is deposited at DDBJ/ENA/GenBank under the accession MJMG00000000, version MJMG01000000. Raw MiSeq data is available under NCBI SRA number SRR3097580.

**How to cite this article**: Brown, A. M. V. *et al.* Genomic evidence for plant-parasitic nematodes as the earliest *Wolbachia* hosts. *Sci. Rep.* **6**, 34955; doi: 10.1038/srep34955 (2016).

## Supplementary Material

Supplementary Information
