# Supplementary material for: Genomic evidence for plant-parasitic nematodes as the earliest Wolbachia hosts
Source: Sci Rep. 2016 Oct 13;6:34955. doi: 10.1038/srep34955 (PMC5062116; doi:10.1038/srep34955)
Supplement: Supplementary Information [file srep34955-s1.pdf]

Supplementary Materials

Title: Genomic evidence for plant-parasitic nematodes as the earliest *Wolbachia* hosts

Authors and affiliations: Amanda M.V. Brown[1*], Sulochana K. Wasala[1], Dana K. Howe[1], Amy B. Peetz[2], Inga A. Zasada[2], Dee R. Denver[1]

[1] Department of Integrative Biology, 3029 Cordley Hall, Oregon State University, Corvallis, OR 97331 USA

[2] USDA-ARS Horticultural Crops Research Laboratory, 3420 NW Orchard Avenue, Corvallis, OR 97330, USA

* Author for Correspondence: Amanda M.V. Brown, Department of Integrative Biology, 3029 Cordley Hall, Oregon State University, Corvallis, OR 97331 USA, Phone 541-737-2993, Fax 541-737-0501, Email browaman@science.oregonstate.edu

Supplementary Table S1. Assembly details for wPpe (assembled with Velvet v1.2.10), and wPni and wFol (assembled with CLC Workbench from reads downloaded from NCBI SRA databases).

	wPpe	wPni	wFol
# Reads (Raw)	19,164,652	104,942,164	32,383,519
Read length	301	101	125
# bp (lengthX#reads)	5,768,560,252	10,599,158,564	4,047,939,875
# Scaffolds (total assembly)	807,072	244,874	31,251
N50 (total assembly)	5,531	419	10,523
Max. scaffold length	1,697,130	7,272	116,564
Sum of scaffold lengths (total assembly)	349,666,756	99,688,041	182,379,350
# Scaffolds matching *Wolbachia*	12	652	121
N50 (matching *Wolbachia*)	95,550	1,789	19,686
Coverage (*Wolbachia* scaffolds)	16.7X	2160X	40.7X
Max. scaffold length (matching *Wolbachia*)	350,570	7,272	99,880
Sum of scaffold lengths (matching *Wolbachia*)	975,127	984,055	1,402,517

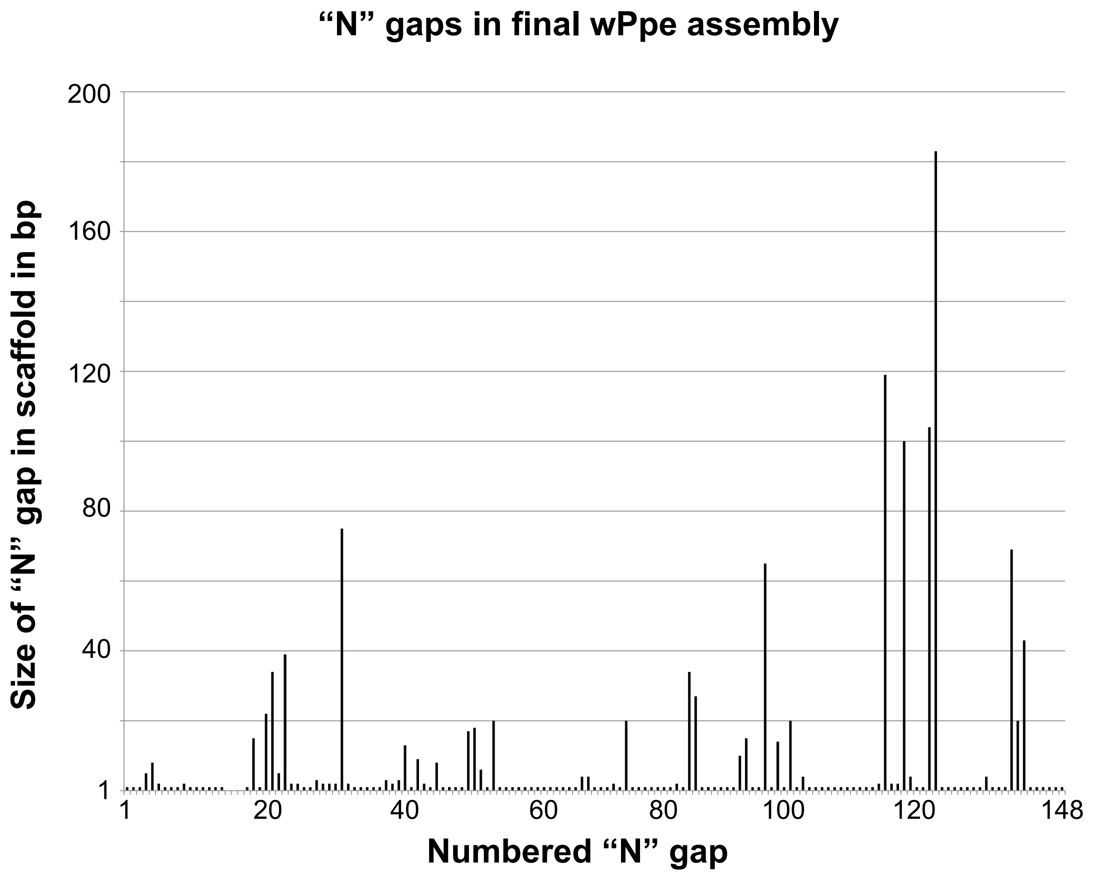

Supplementary Figure S1. Genome assembly "N" gaps (missing data in scaffolds denoted by one or more Ns). There were 148 gaps, most of these very small, with 6 gaps longer than 60 bp and the largest gap at 183 bp. The total number of Ns in the assembly was 1288, which if divided by the total assembly length (975,127) results in 0.13% of the assembly represented by Ns.

Supplementary Table S2. *Wolbachia* strains compared in genome-wide analyses.

Strain	Host group	Host species	Phenotype	Group	Genome [illegible]	Predicted [illegible]	Accession	Data status
wDim	N: filarial nematode	*Dirofilaria immitis*	M	C	921,018	756	NEMBASE4	wgs
wOo	N: filarial nematode	*Onchocerca ochengi*	M	C	957,990	881	NC_018267.1	complete
wOvc	N: filarial nematode	*Onchocerca volvulus*	M	C	960,618	642	NZ_HG810405	complete
wPpe	N: plant-parasitic nematode	*Pratylenchus penetrans*	?	L	975,127	962	XXXX	This study
wPni	A: aphid	*Pentalonia nigronervosa*	M	M	984,055	900	SRX766492	SRA
wLs	N: filarial nematode	*Litomosoides sigmodontis*	M	D	1,048,988	880	NEMBASE4	wgs
wBm	N: filarial nematode	*Brugia malayi*	M	D	1,080,084	984	NC_006833	complete
wDac	A: scale insect	*Dactylopius coccus*	?	B	1,124,974	1545	JMCJ01000000	wgs
wAlbB	A: mosquito	*Aedes albopictus*	CI	B	1,162,431	955	NZ_CAGB01000000	wgs
wCle	A: bedbug	*Cimex lectularius*	M	F	1,250,060	1152	NZ_AP013028	complete
wMel	A: fruit fly	*Drosophila melanogaster*	CI	A	1,267,782	1073	NC_002978	complete
wAu	A: fruit fly	*Drosophila simulans*	Non-CI	A	1,268,461	1266	LK055284	complete
wHa	A: fruit fly	*Drosophila simulans*	CI	A	1,295,804	1077	NC_021089	complete
wNo	A: fruit fly	*Drosophila simulans*	CI	B	1,301,823	1071	NC_021084	complete
wFol	A: springtail	*Folsomia candida*	PI/M	E	1,402,517	1180	SAMN02721328	SRA
wSuz	A: fruit fly	*Drosophila suzukii*	FM	A	1,415,350	1211	NZ_CAOU02000000	wgs
wAna	A: fruit fly	*Drosophila ananassae*	CI	A	1,440,750	1012	AAGB01000000	wgs
wRi	A: fruit fly	*Drosophila simulans*	CI, FM	A	1,445,873	1248	NC_12416	complete
wPipPel	A: mosquito	*Culex quinquefasciatus*	CI, FM	B	1,482,455	1273	NC_010981	complete

[1] Strains wPpe and wPni were named for their hosts *Pratylenchus penetrans* and *Pentalonia nigronervosa*, respectively. N: = nematode host, A: = arthropod host, M = obligate mutualist, CI = cytoplasmic incompatibility, FM = facultative mutualist conferring fitness benefits but not thought to be universally required, PI = pathogenesis inducing.

Supplementary Table S3. Output from recombination tests (Phi, NSS, Max chi-squared) generated in PhiPack with 1000 permutations and window of 200 bp performed on final 79 protein coding gene alignment (moderate stringency Gblock output), with all taxa included, outgroups (*Anaplasma* and *Ehrlichia* species) excluded, and outgroups and groups A+B excluded. NSS = Neighbour Similarity Score.

	p-value<br>NSS test	p-value<br>Max χ^2 test	p-value<br>Phi test
All taxa included	0.00e+0	0.00e+0	0.00e+0
Outgroups excluded	0.00e+0	0.00e+0	1.00e+00
Outgroups and A+B supergroups excluded	0.00e+0	0.00e+0	1.00e+00

Supplementary Table S4. Output from Xia's nucleotide saturation test generated in DAMBE5 on final 79 protein coding gene alignment (moderate stringency Gblock output), with all taxa included, including outgroups.

Test of substitution saturation (Xia et al. 2003; Xia and Lemey 2009)

Analysis performed on fully resolved sites only.
Testing whether the observed Iss is significantly lower than Iss.c.

```
Part I. For a symmetrical tree.
==============================================================
Prop. invar. sites            0.0000
Mean H                        0.5597
Standard Error                0.0024
Hmax                          1.8176
Iss                           0.3079
Iss.c                         0.8314
T                             215.3890
DF                            43629
Prob (Two-tailed)             0.0000
95% Lower Limit               0.3032
95% Upper Limit               0.3127

==============================================================
Part II. For an extreme asymmetrical (and generally very
unlikely) tree.
==============================================================
Iss.c                         0.6724
T                             149.9516
DF                            43629
Prob (Two-tailed)             0.0000

95% Lower Limit               0.3032
95% Upper Limit               0.3127

==============================================================

Interpretation of results:
            Significant Difference
            ----------------------
            Yes              No
-----------------------------------------------------
Iss < Iss.c    Little           Substantial
            saturation       saturation
-----------------------------------------------------
Iss > Iss.c    Useless          Very poor
            sequences        for phylogenetics
-----------------------------------------------------
```

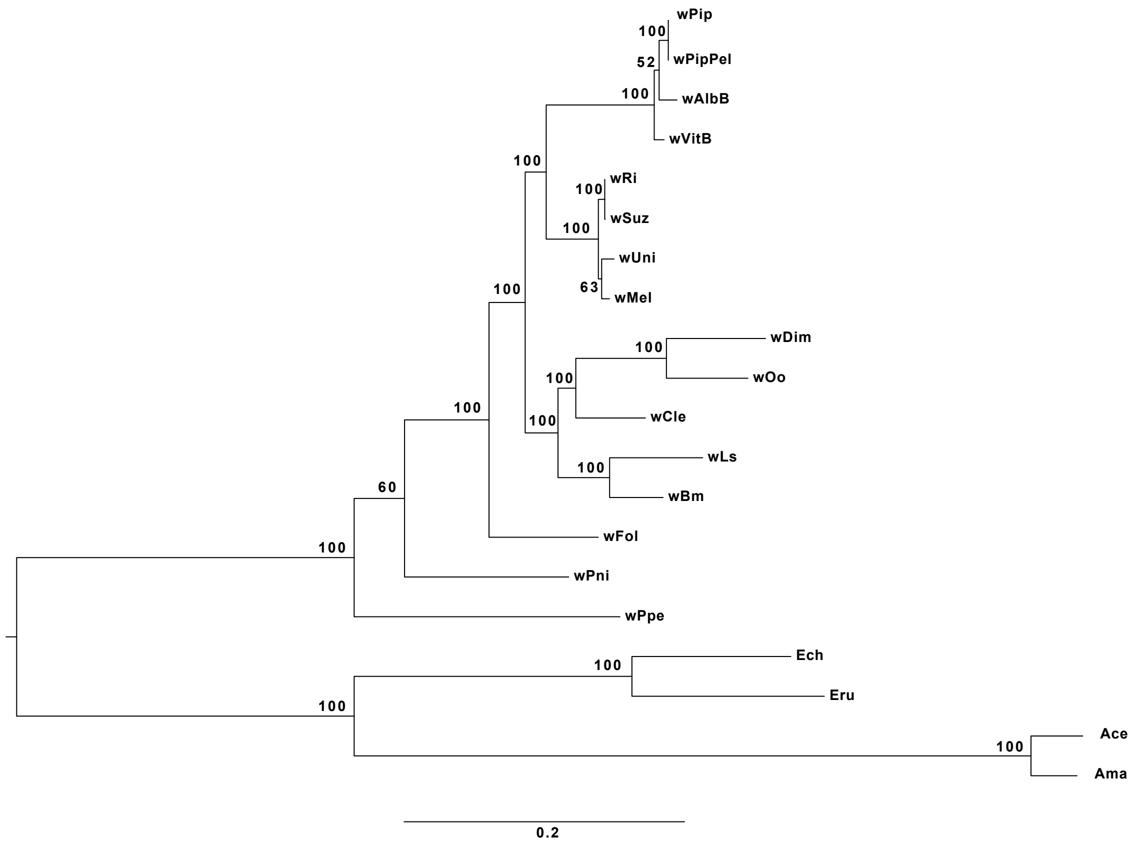

Supplementary Figure S2. Maximum likelihood phylogeny of *Wolbachia* strains and outgroups listed in Supplementary Table S2 based on protein sequences from 79 conserved single-copy orthologous genes, comprising 20,488 amino acid alignment positions with bootstrap values from 1,000 replicates shown on nodes.

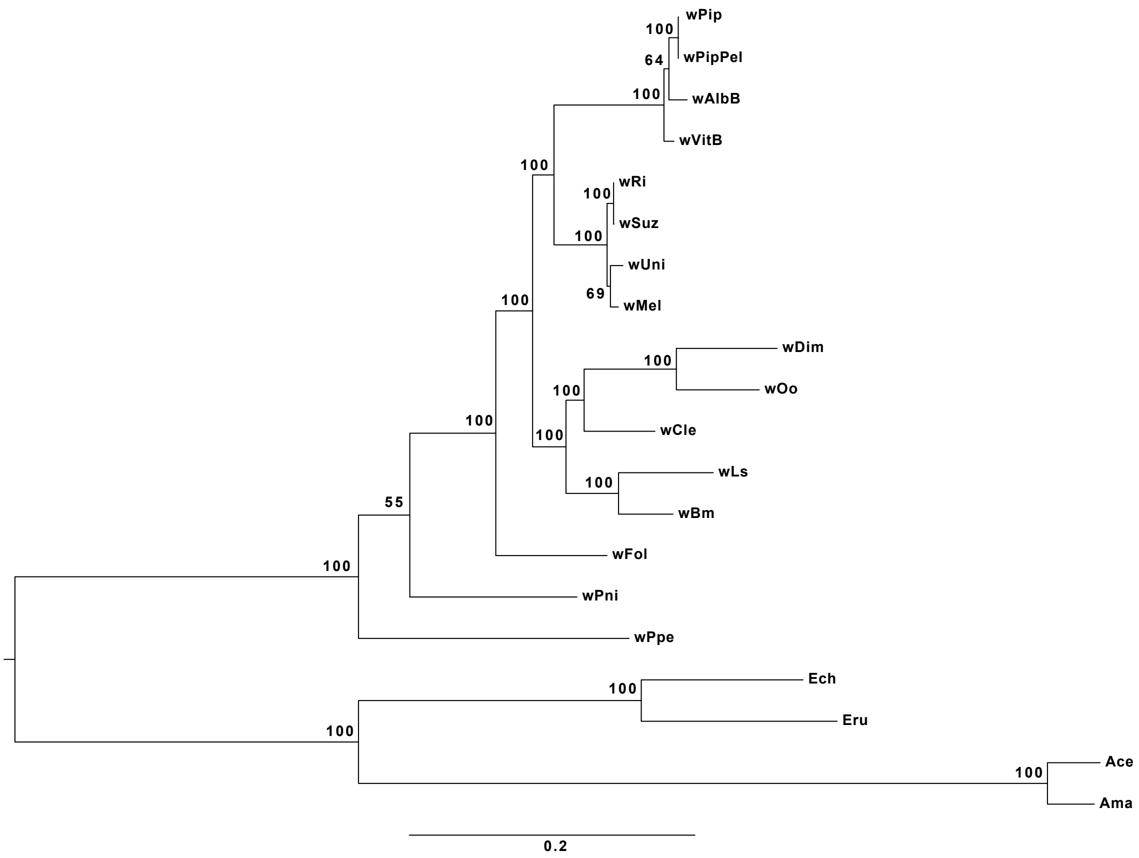

Supplementary Figure S3. Maximum likelihood phylogeny of *Wolbachia* strains and outgroups listed in Supplementary Table S2 based on protein sequences from 79 conserved single-copy orthologous genes, generated using the most stringent Gblocks parameters, resulting in 14,235 amino acid alignment positions. Bootstrap values from 1,000 replicates are shown on nodes.

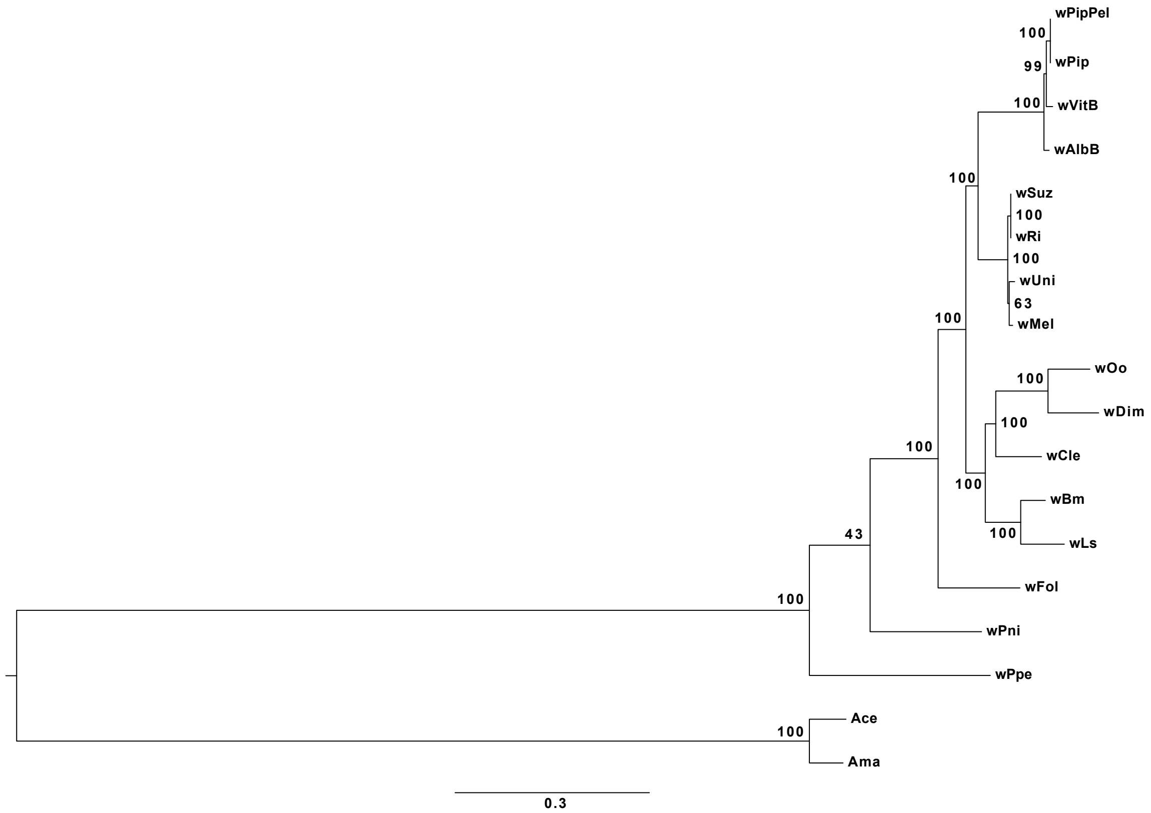

Supplementary Figure S4. Maximum likelihood phylogeny of *Wolbachia* strains including only *Anaplasma* spp. as outgroups, based on nucleotide sequences from 79 conserved single-copy orthologous genes, comprising 61,465 nucleotide alignment positions with bootstrap values from 1,000 replicates shown on nodes.

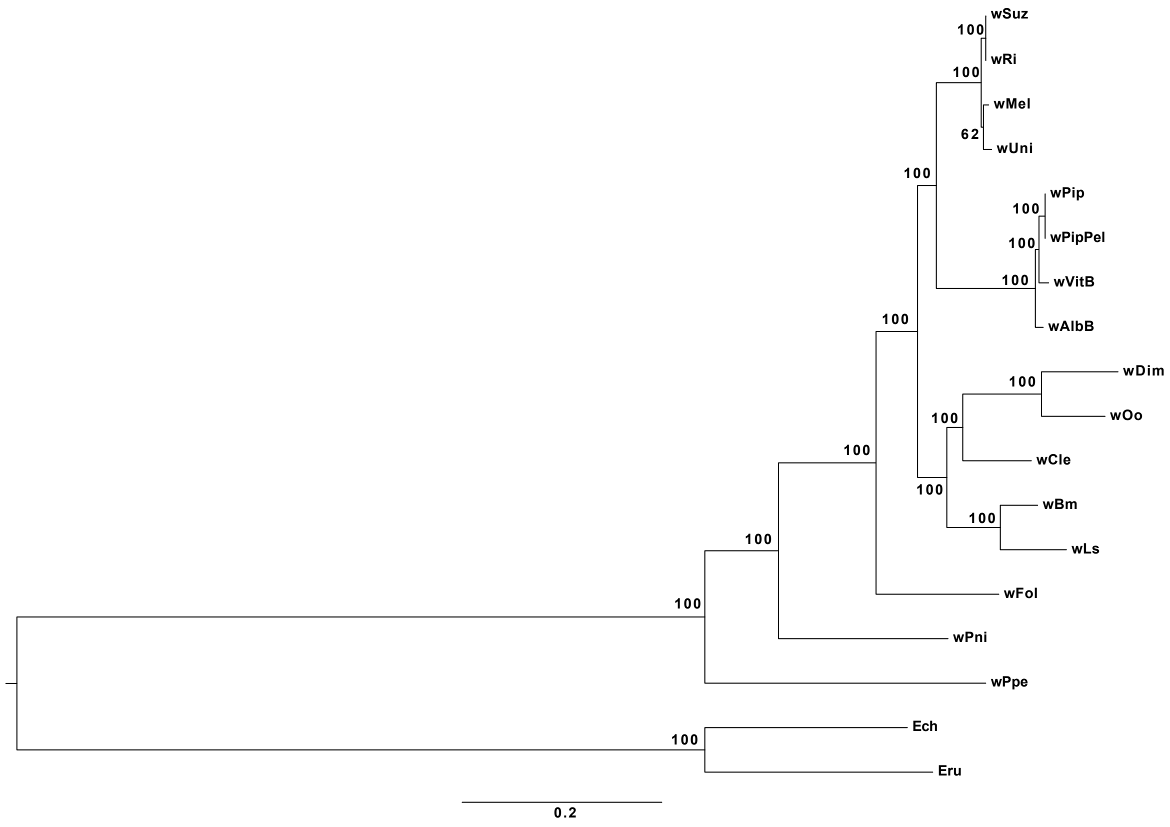

Supplementary Figure S5. Maximum likelihood phylogeny of *Wolbachia* strains including only *Ehrlichia* spp. as outgroups, based on nucleotide sequences from 79 conserved single-copy orthologous genes, comprising 61,465 nucleotide alignment positions with bootstrap values from 1,000 replicates shown on nodes.

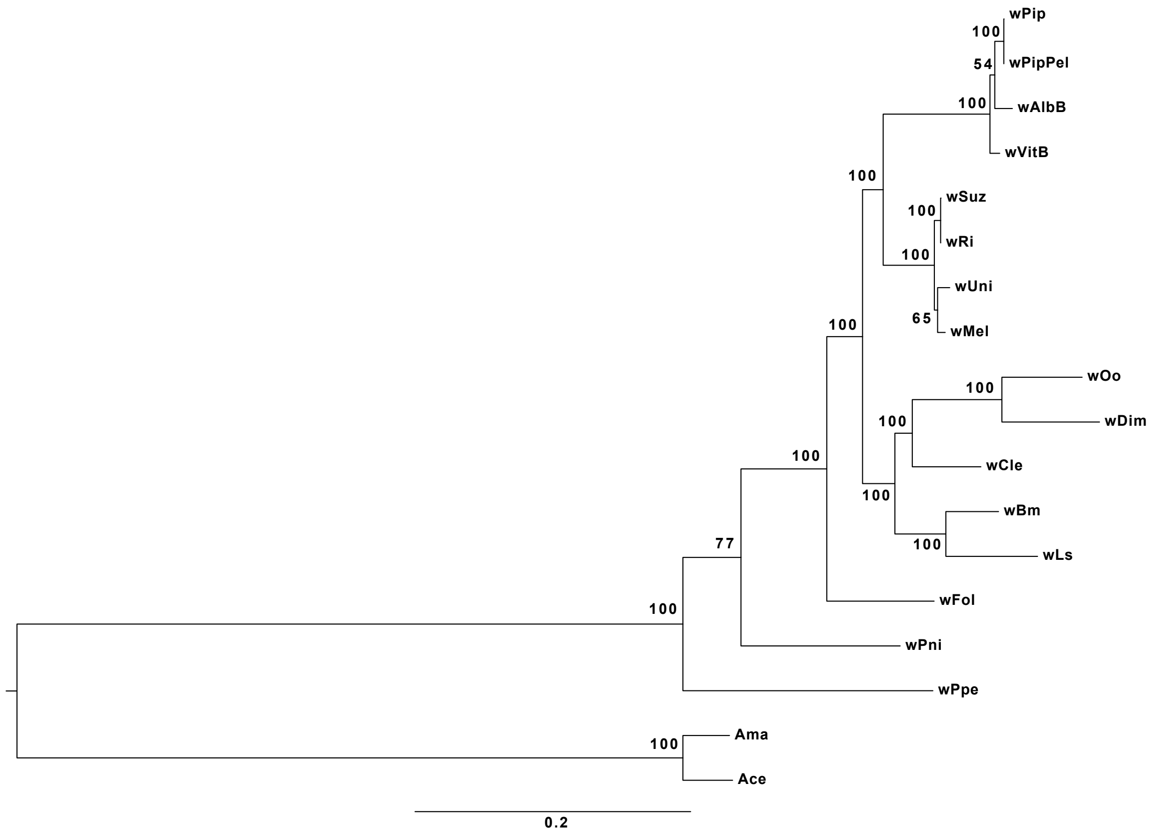

Supplementary Figure S6. Maximum likelihood phylogeny of *Wolbachia* strains including only *Anaplasma* spp. as outgroups, based on protein sequences from 79 conserved single-copy orthologous genes, comprising 20,488 amino acid alignment positions with bootstrap values from 1,000 replicates shown on nodes.

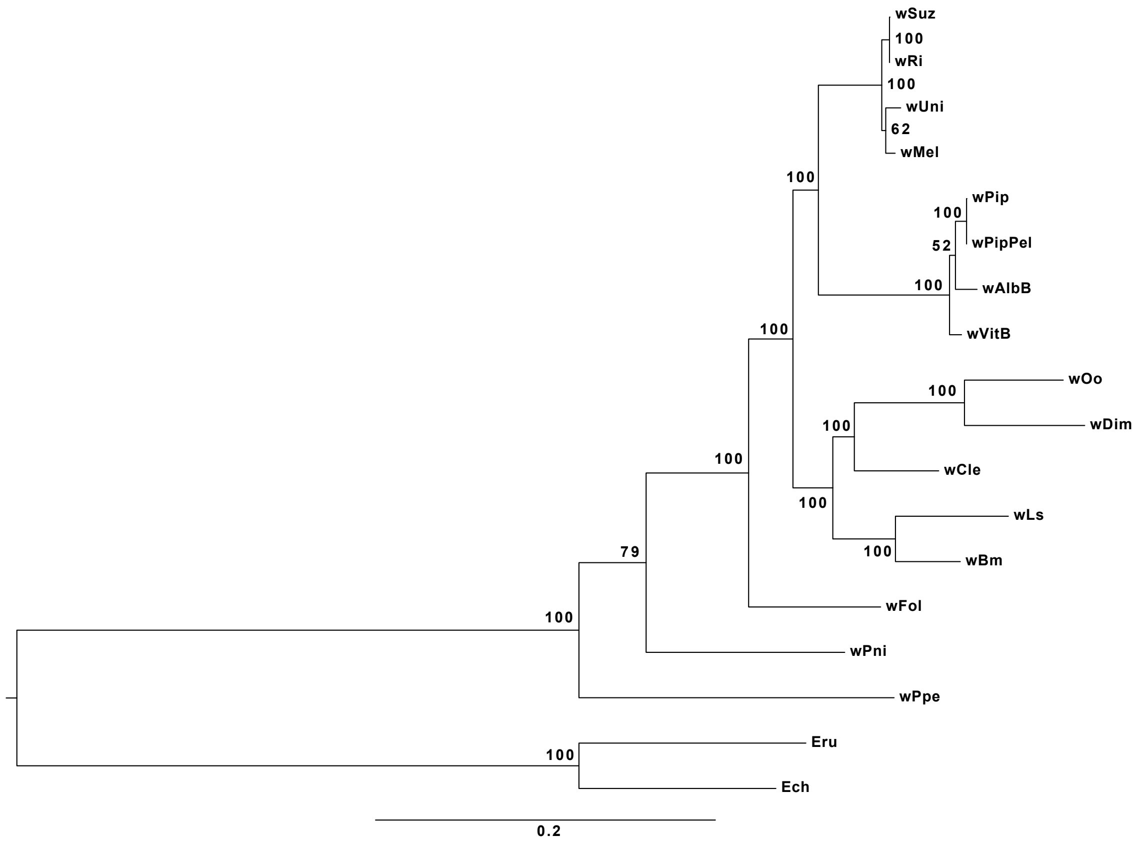

Supplementary Figure S7. Maximum likelihood phylogeny of *Wolbachia* strains including only *Ehrlichia* spp. as outgroups, based on protein sequences from 79 conserved single-copy orthologous genes, comprising 20,488 amino acid alignment positions with bootstrap values from 1,000 replicates shown on nodes.

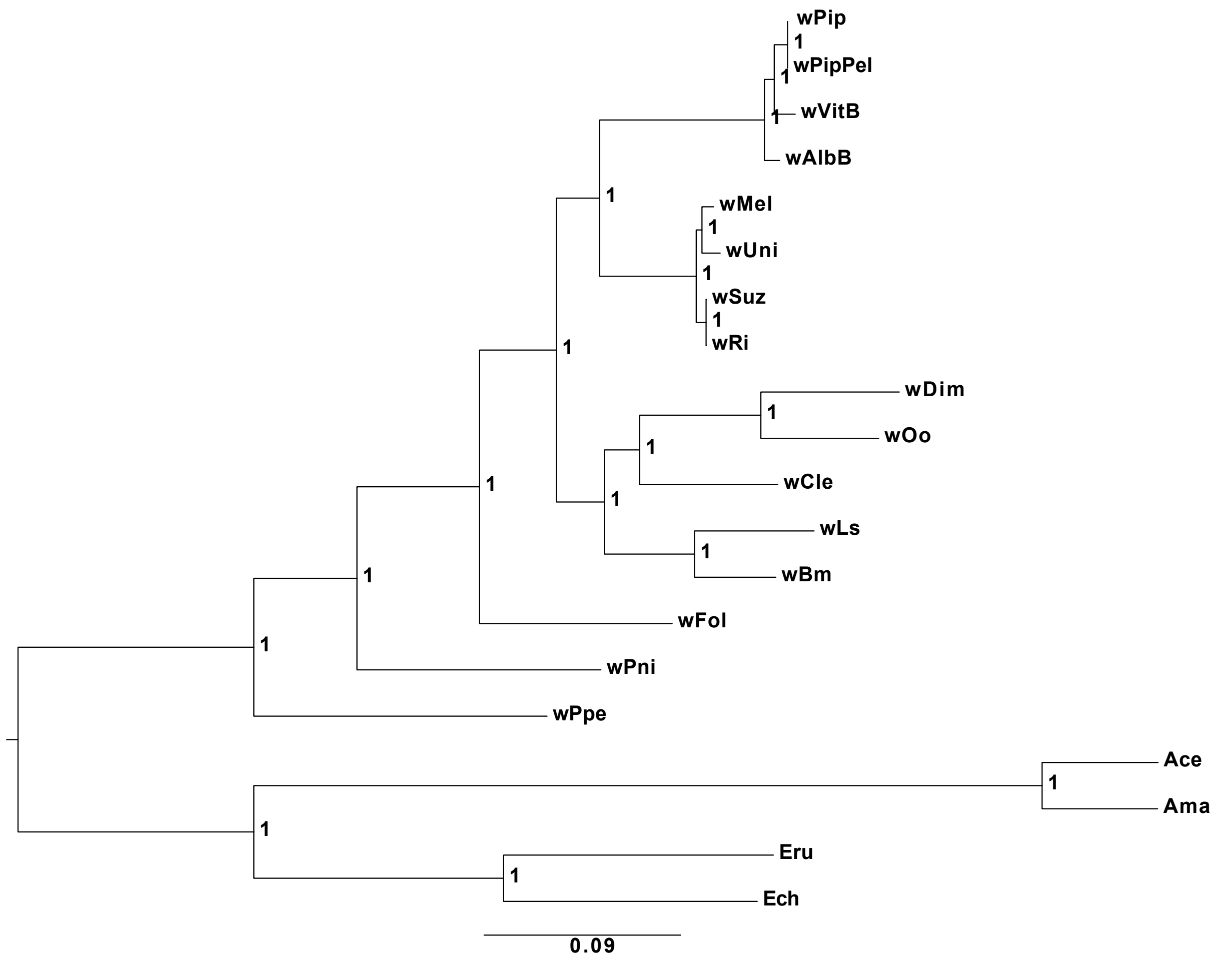

Supplementary Figure S8. Bayesian phylogeny of *Wolbachia* strains and outgroups listed in Supplementary Table S2 based on nucleotide sequences from 79 conserved single-copy orthologous genes, comprising 61,465 nucleotide alignment positions with posterior probabilities shown on nodes.

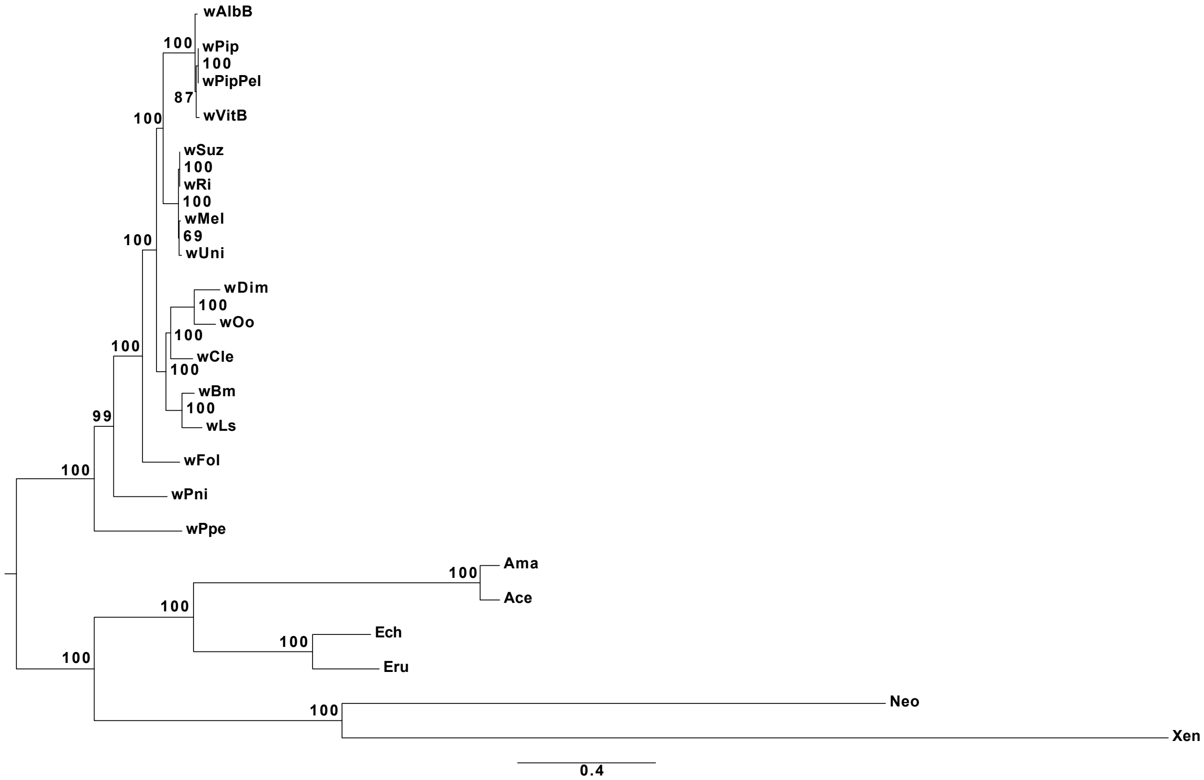

Supplementary Figure S9. Maximum likelihood phylogeny of *Wolbachia* strains and outgroups listed in Supplementary Table S2 with the addition of *Neorickettsia sennetsu* PRJNA357 (Neo) and *Candidatus* Xenolissoclinum pacificiensis PRJNA219341 (Xen), based on nucleotide sequences from 36 conserved single-copy orthologous genes, comprising 29,437 nucleotide alignment positions with bootstrap values from 1,000 replicates shown on nodes.

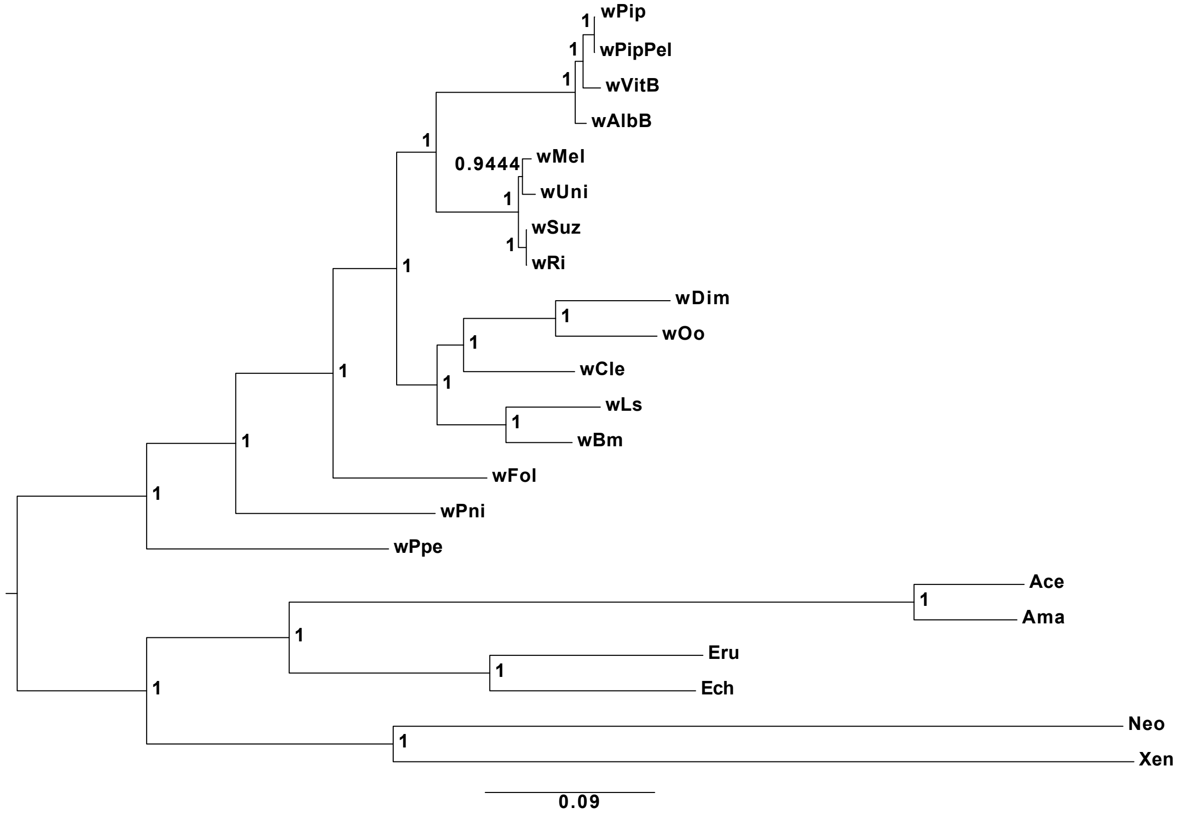

Supplementary Figure S10. Bayesian phylogeny of *Wolbachia* strains and outgroups listed in Supplementary Table S2 with the addition of *Neorickettsia sennetsu* PRJNA357 (Neo) and *Candidatus* Xenolissoclinum pacificiensis PRJNA219341 (Xen), based on nucleotide sequences from 36 conserved single-copy orthologous genes, comprising 29,437 nucleotide alignment positions with posterior probabilities shown on nodes.

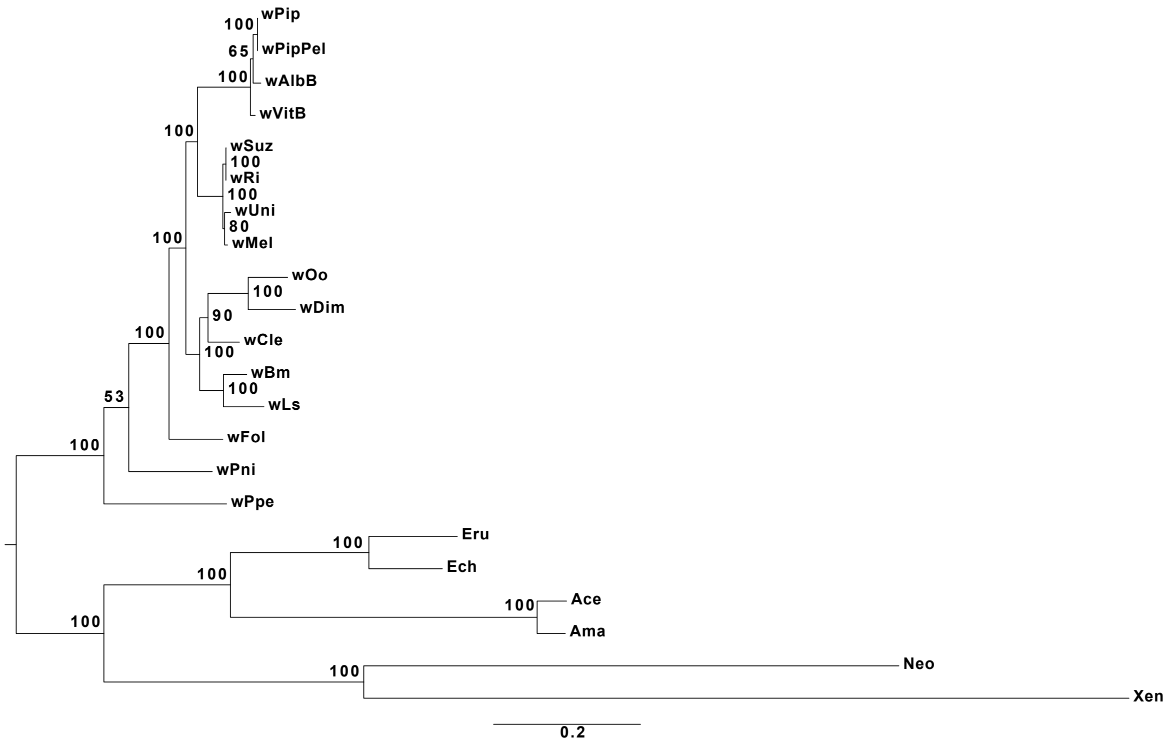

Supplementary Figure S11. Maximum likelihood phylogeny of *Wolbachia* strains and outgroups listed in Supplementary Table S2 with the addition of *Neorickettsia sennetsu* PRJNA357 (Neo) and *Candidatus* Xenolissoclinum pacificiensis PRJNA219341 (Xen), based on protein sequences from 36 conserved single-copy orthologous genes, comprising 9,812 amino acid alignment positions with bootstrap values from 1,000 replicates shown on nodes.

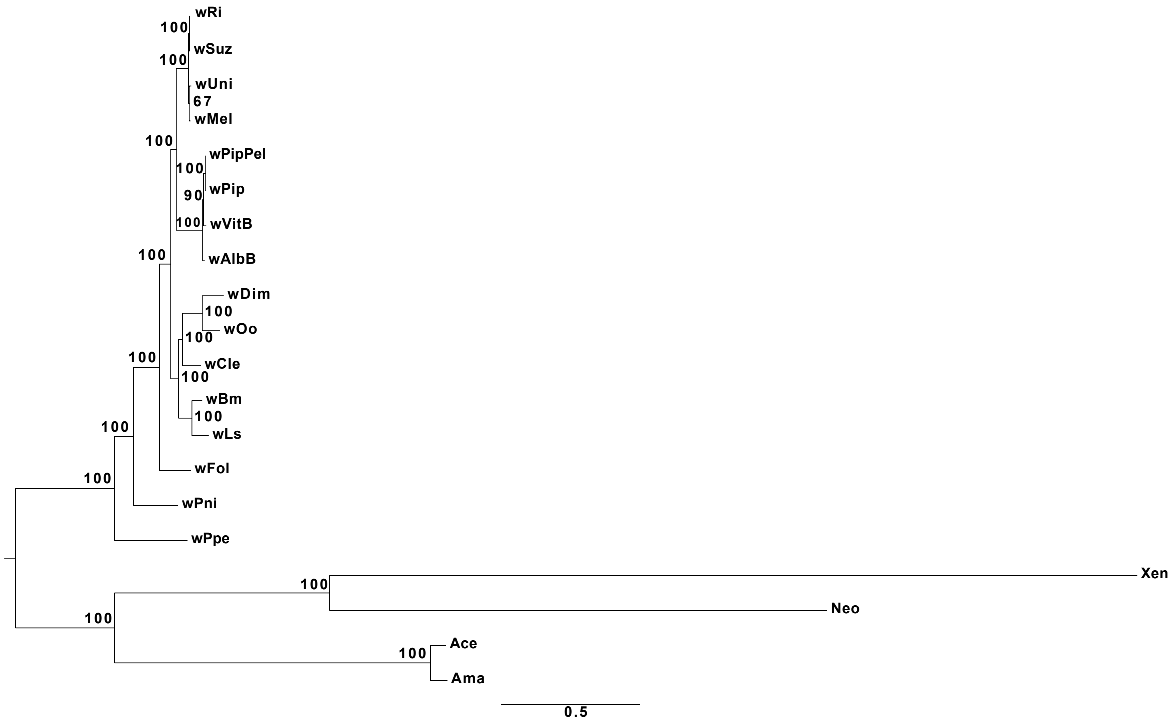

Supplementary Figure S12. Maximum likelihood phylogeny of *Wolbachia* strains with only *Anaplasma* spp. and *Neorickettsia sennetsu* PRJNA357 (Neo) and *Candidatus* Xenolissoclinum pacificiensis PRJNA219341 (Xen) as outgroups, based on nucleotide sequences from 36 conserved single-copy orthologous genes, comprising 29,437 nucleotide alignment positions with bootstrap values from 1,000 replicates shown on nodes.

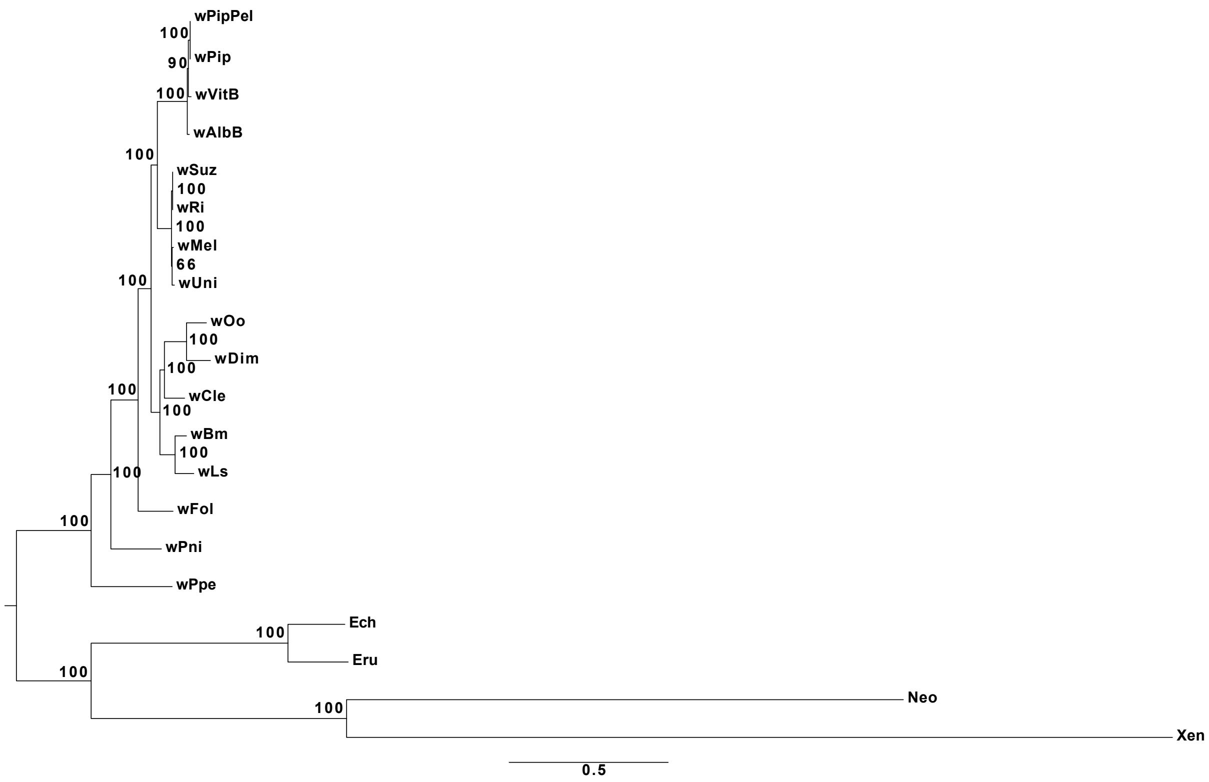

Supplementary Figure S13. Maximum likelihood phylogeny of *Wolbachia* strains with only *Ehrlichia* spp. and *Neorickettsia sennetsu* PRJNA357 (Neo) and *Candidatus* Xenolissoclinum pacificiensis PRJNA219341 (Xen) as outgroups, based on nucleotide sequences from 36 conserved single-copy orthologous genes, comprising 29,437 nucleotide alignment positions with bootstrap values from 1,000 replicates shown on nodes.

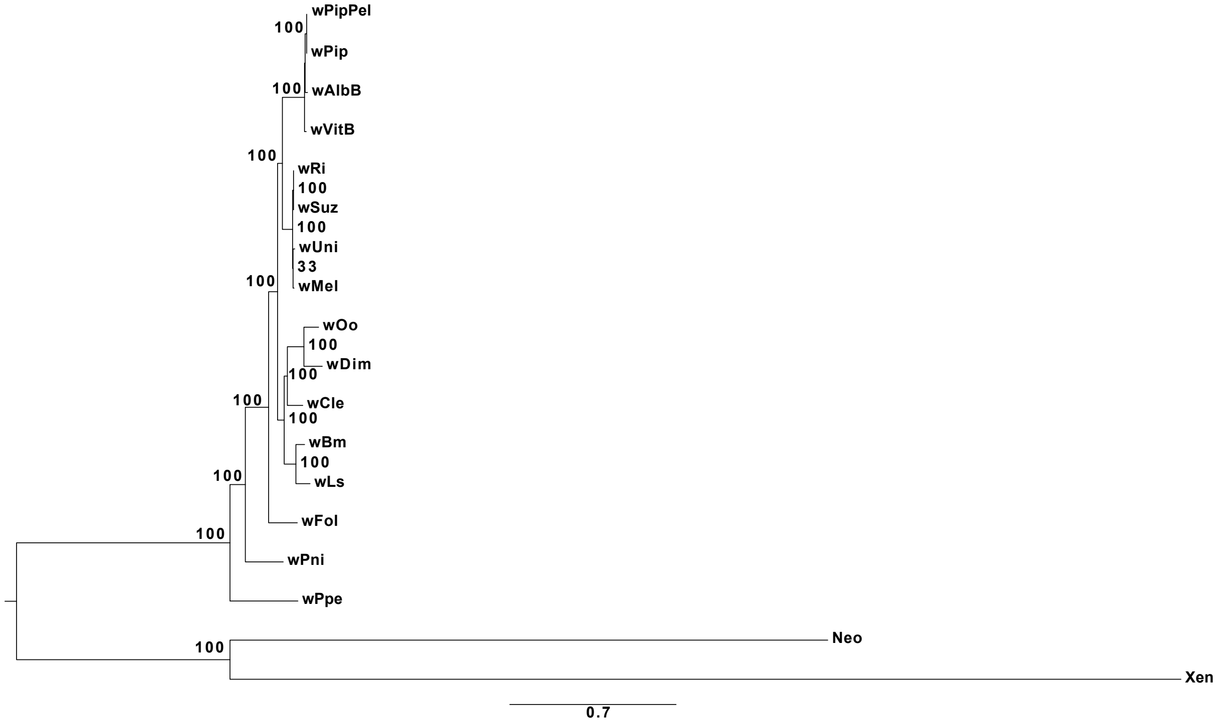

Supplementary Figure S14. Maximum likelihood phylogeny of *Wolbachia* strains with only *Neorickettsia sennetsu* PRJNA357 (Neo) and *Candidatus* Xenolissoclinum pacificiensis PRJNA219341 (Xen) as outgroups, based on nucleotide sequences from 36 conserved single-copy orthologous genes, comprising 29,437 nucleotide alignment positions with bootstrap values from 1,000 replicates shown on nodes.

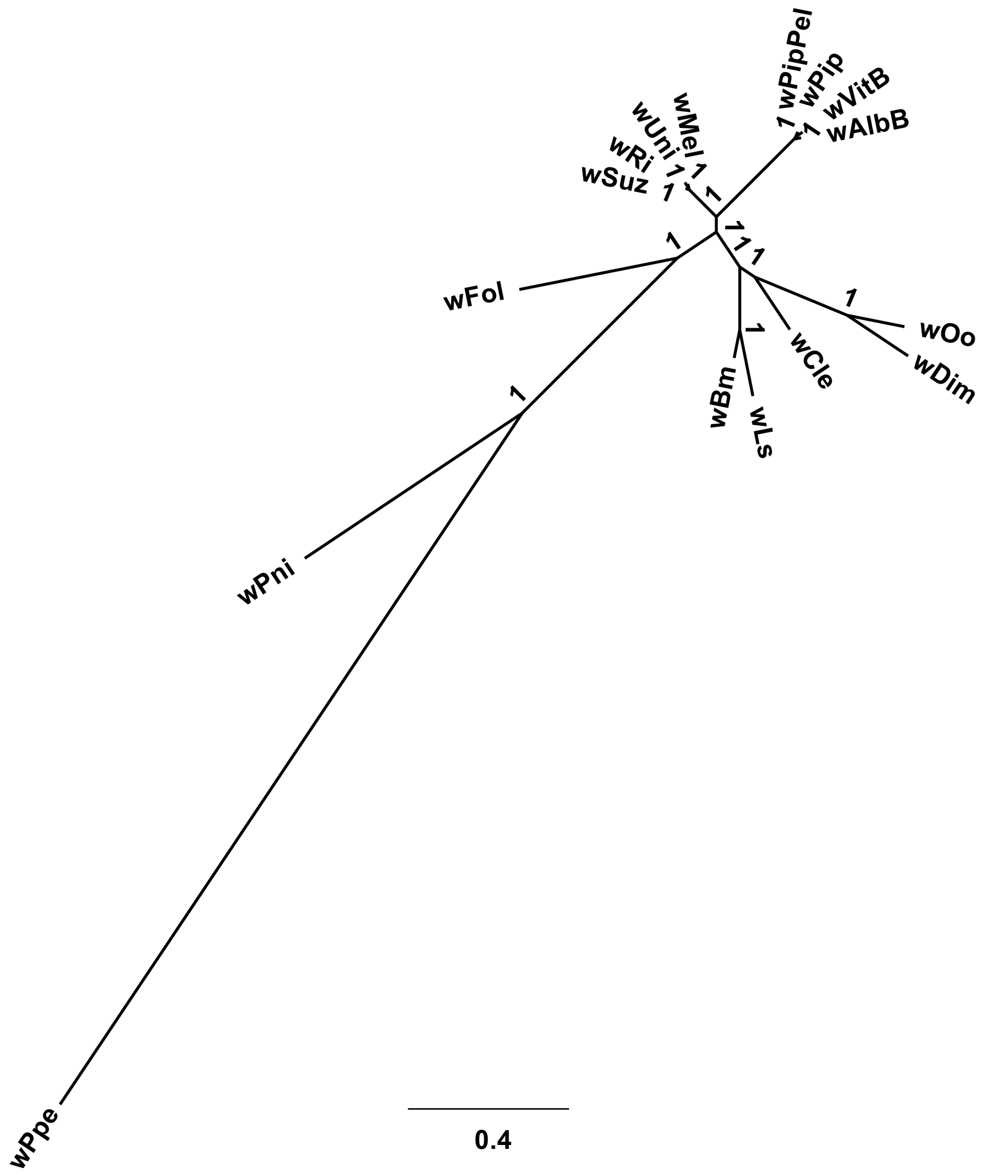

Supplementary Figure S15. Unrooted tree of *Wolbachia* strains without outgroups, generated in PhyloBayes under the CAT+GTR model, based on nucleotide sequences from 79 conserved single-copy orthologous genes, comprising 61,465 nucleotide alignment positions with posterior probabilities shown on nodes.

Supplementary Table S5. Summary of PhyloBayes results relating to support of root positions in *Wolbachia*, with posterior probabilities on nodes >0.90. Loci notation: 3 = 16S rRNA+ftsZ+groEL; 36 = 36conserved protein coding genes; 79 = 79 orthologous protein coding genes . a.a. = amino acid sequence.

Loci	Type	Taxa	Gblock stringency	Model	wPpe & wPni paired	wPpe & wPni root or wPpe polytomy at root	Additional notes
3	DNA	All	Normal	CAT-Poisson	0.95	Yes	Large root polytomy
3	DNA	+Neo +Xen	Normal	CAT-Poisson	0.95	Yes	Large root polytomy
3	DNA	All	Normal	CAT-GTR	1	Yes	Base taxa polytomy
3	DNA	+Neo +Xen	Normal	CAT-GTR	0.94	Yes	Large root polytomy
36	DNA	-Ehr	Normal	CAT-Poisson	0.93	Yes	Large root polytomy
36	DNA	-Ana	Normal	CAT-Poisson	0.91	Yes	Root with group C
36	a.a.	+N -Ehr/Ana	Normal	CAT-GTR	0.91	Yes	Root polytomy
79	DNA	All	Normal	CAT-GTR	1	Yes	Base taxa polytomy
79	DNA	All	High	CAT-GTR	1	Yes	
79	DNA	-Ana	Normal	CAT-Poisson	0.93	Yes	Root with grroup C
79	DNA	-Ehr	Normal	CAT-Poisson	No	Yes	Large root polytomy
79	DNA	-Ana	HIgh	CAT-Poisson	No	Yes	Large root polytomy
79	DNA	-Ehr	High	CAT-Poisson	0.91	Yes	Large root polytomy
79	a.a.	All	Normal	CAT-GTR	1	Yes	
79	a.a.	All	Normal	CAT-Poisson	No	Yes	Base taxa polytomy
79	a.a.	-Ana	Normal	CAT-GTR	0.97	Yes	
79	a.a.	-Ehr	Normal	CAT-GTR	0.94	Yes	Root polytomy
79	a.a.	-Ana	Normal	CAT-Poisson	No	Yes	Root polytomy
79	a.a.	-Ehr	Normal	CAT-Poisson	No	Yes	Root polytomy
79	a.a.	All	HIgh	CAT-Poisson	0.95	Yes	Root polytomy
79	a.a.	All	HIgh	CAT-GTR	1	Yes	Base taxa polytomy

Supplementary Table S6. Results of alternative root testing using the Approximately Unbiased test (AU) in CONSEL for alternative root constraints compared with the best unconstrained tree (tree testing in PhyML).

Rank	Constraint	Log likelihood of best tree	Obs	AU test	Bootstrap probability (NP)	AU confidence interval (at p = 0.05)
1	Unconstrained (wPpe root)	-253151.9	0.0	0.623	0.616	0
2	wPpe root	-253151.9	0.0	0.390	0.384	0
3	wPni root	-253219.2	67.3	6e-05	4e-04	35.2
4	wPpe after wFol	-253971.3	819.4	5e-55	0	721.1
5	wFol root	-254117.2	965.3	3e-08	0	860.9
6	wPpe in clade F	-255155.5	2003.7	2e-30	0	1820.1
7	wPpe in clade CDF	-256535.7	3383.8	1e-109	0	3169.7
8	wPpe in clade AB	-260321.8	7169.9	0.005	0	6880.4

Supplementary Table S7. Additional strain data for 16S, ftsZ and groEL phylogenetic analyses.

Strain	Host species	16S rRNA	ftsZ	groEL
wBta	A: whitefly, *Bemisia tabaci*	KF454771	HQ404795	JN896339
wCit	A: psyllid, *Diaphorina citri*	NZ_AMZJ00000000	NZ_AMZJ00000000	NZ_AMZJ00000000
wMen	A: insect, *Mengenilla moldrzyki*	AGDA010988940	AGDA01091666	AGDA01093537
wBry	A: mite, *Bryobia sp.*	EU499316	EU499321	EU499333
wRad	N: plant-parasitic nematode, *Raopholus similis*	KF059257	EU833483	EU833484

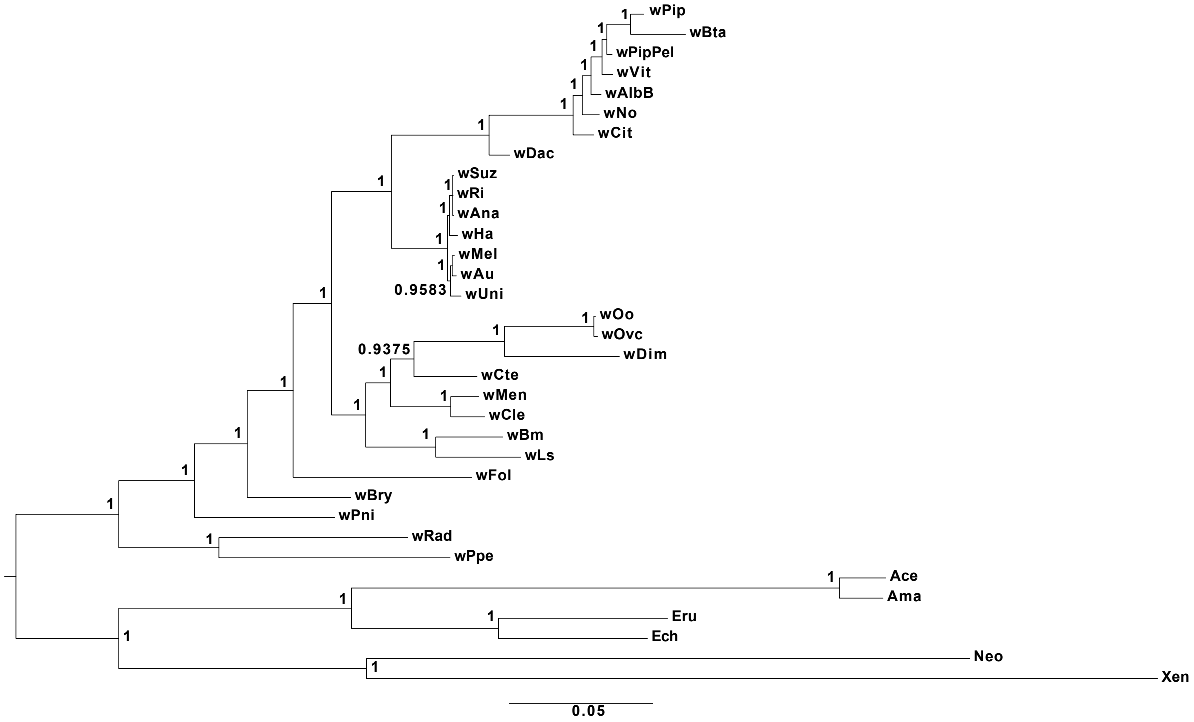

Supplementary Figure S16. Bayesian phylogeny of *Wolbachia* strains and outgroups listed in Supplementary Table S2 based on nucleotide sequences from 16S ribosomal RNA, ftsZ and groEL, comprising 4,307 nucleotide alignment positions with posterior probabilities shown on nodes.

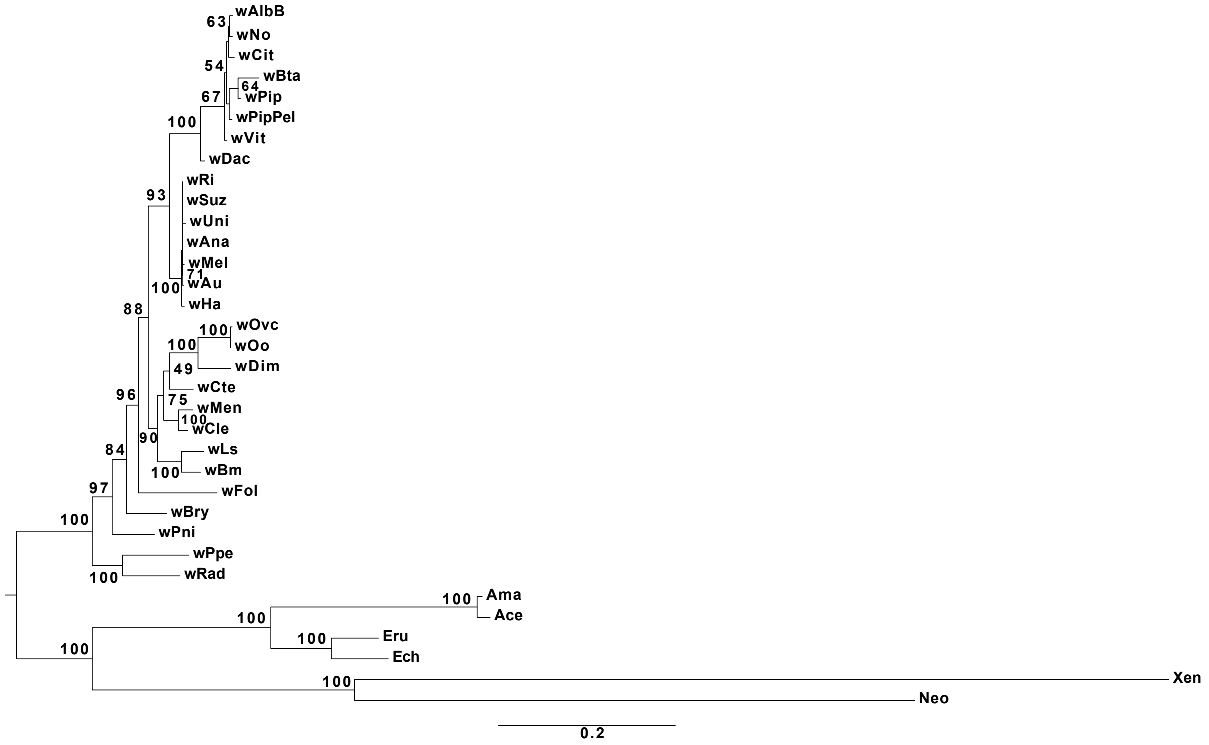

Supplementary Figure S17. Maximum likelihood phylogeny of *Wolbachia* strains and outgroups listed in Supplementary Table S2 based on nucleotide sequences from 16S ribosomal RNA, ftsZ and groEL, generated using the most stringent Gblocks parameters, resulting in 2,155 nucleotide alignment positions. Bootstrap values from 1,000 replicates are shown on nodes.

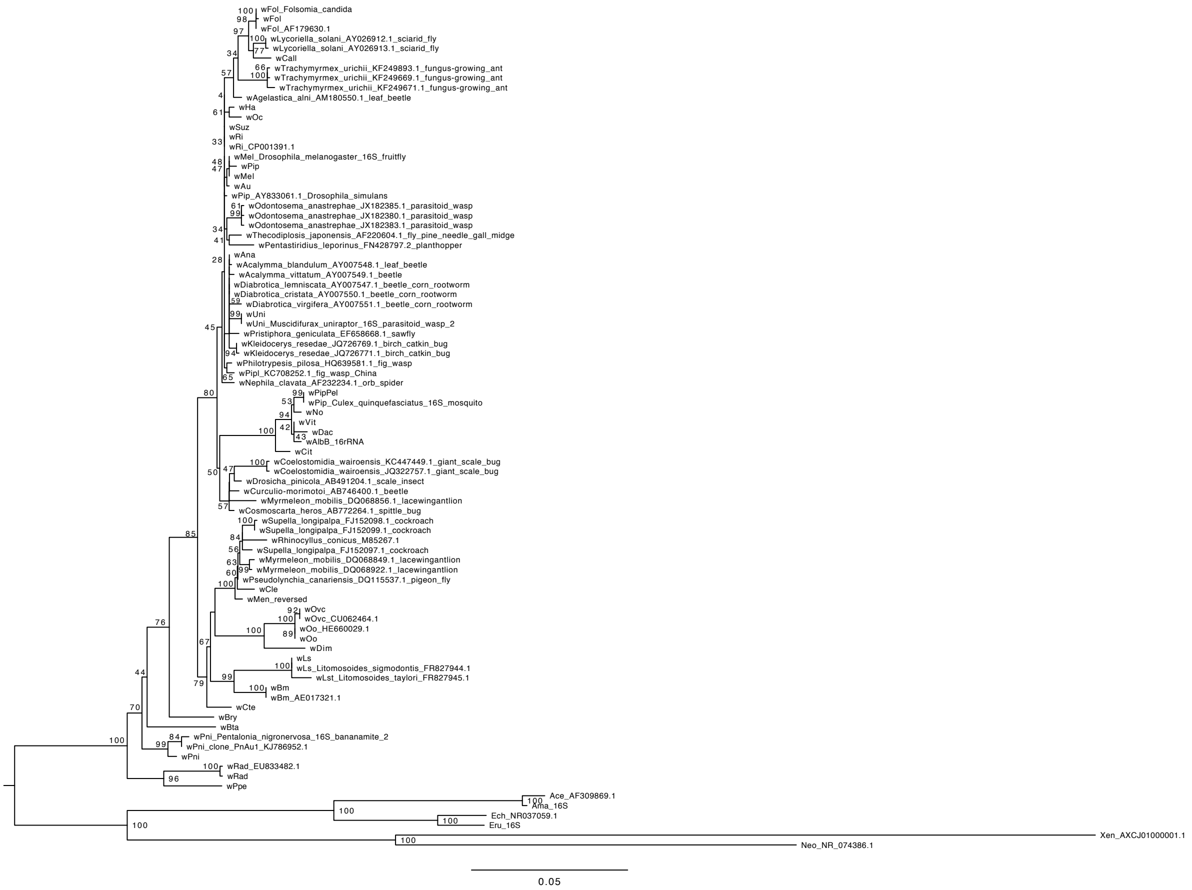

Supplementary Figure S18. Maximum likelihood phylogeny of 80 *Wolbachia* sequences representing 53 host-associated strains based on nucleotide sequences from 16S ribosomal RNA, comprising 1,491 nucleotide positions, with bootstrap values from 1,000 replicates shown on nodes.

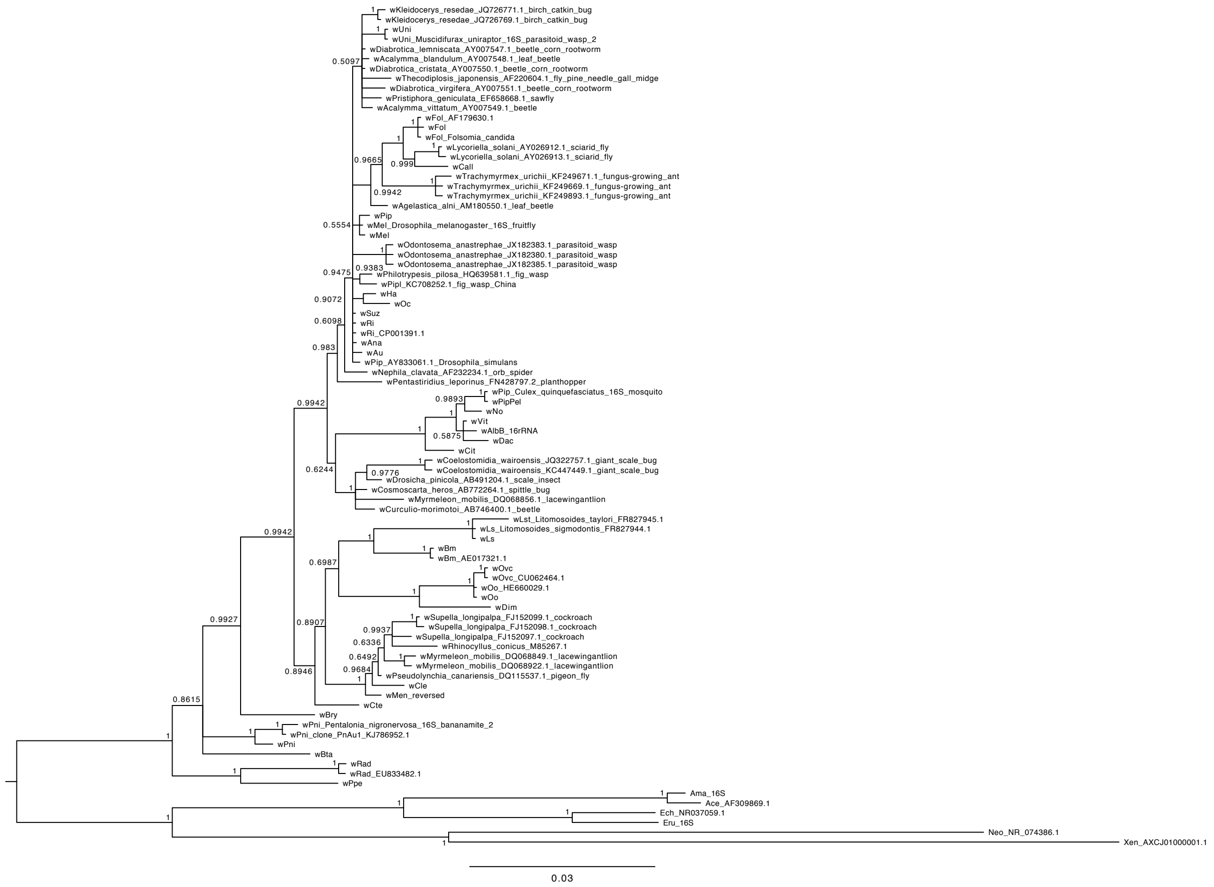

Supplementary Figure S19. Bayesian phylogeny of 80 *Wolbachia* sequences representing 53 host-associated strains based on nucleotide sequences from 16S ribosomal RNA, comprising 1,491 nucleotide positions, with posterior probabilities shown on nodes.

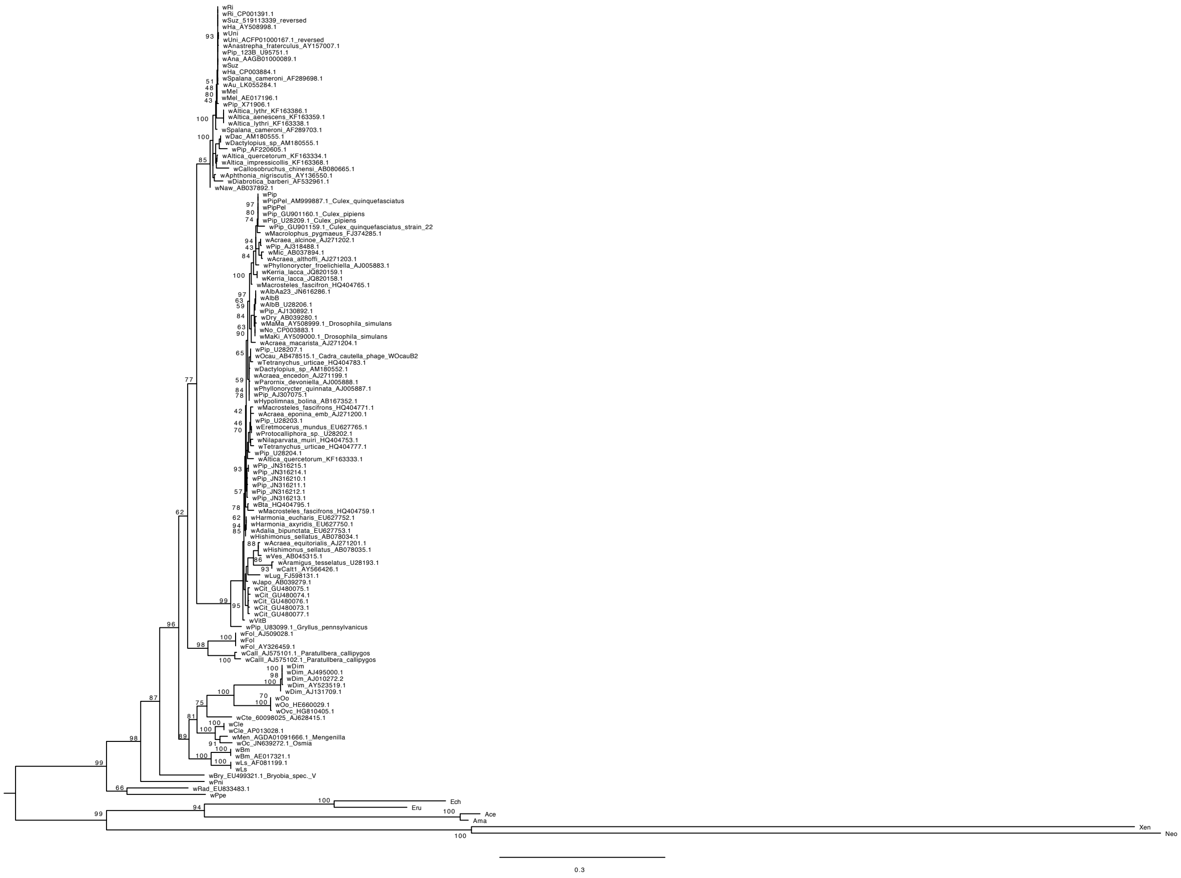

Supplementary Figure S20. Maximum likelihood phylogeny of 123 *Wolbachia* sequences representing 81 host-associated strains based on nucleotide sequences from the ftsZ gene, comprising 867 nucleotide positions, with bootstrap values from 1,000 replicates shown on nodes.

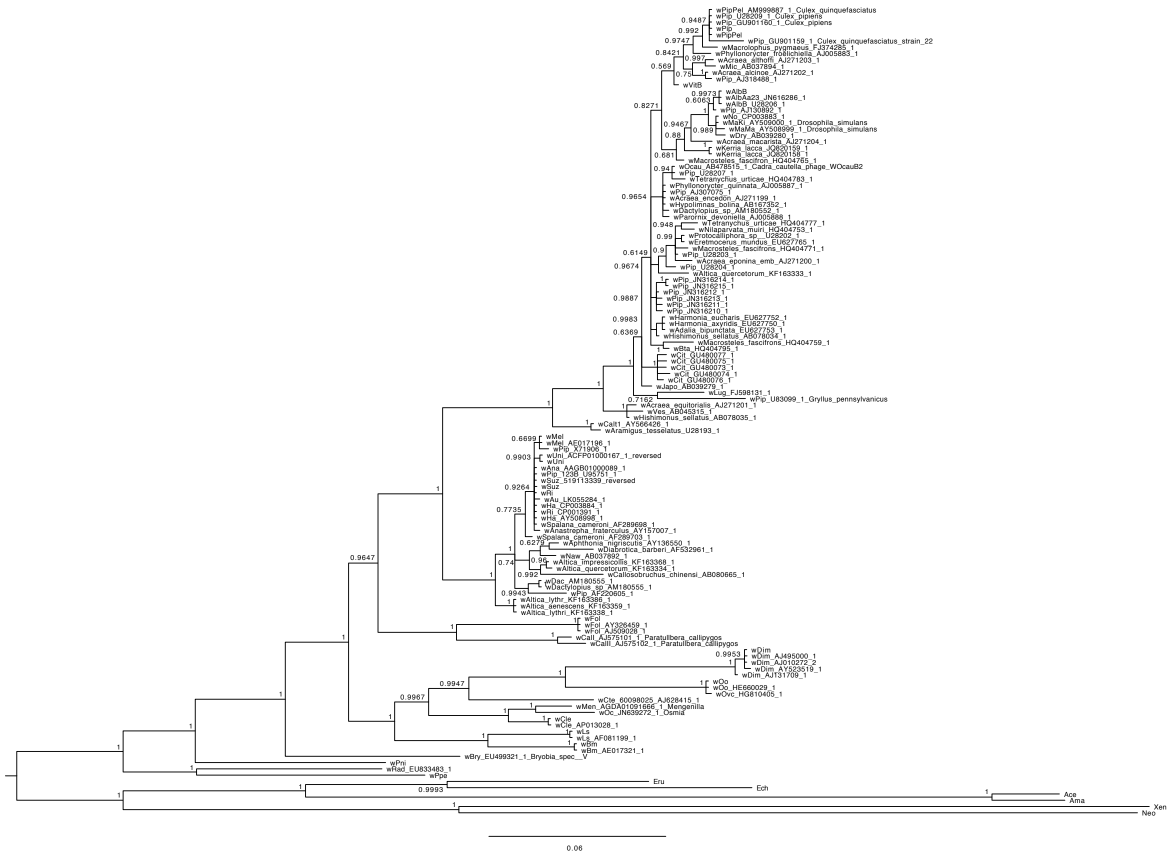

Supplementary Figure S21. Bayesian phylogeny of 123 *Wolbachia* sequences representing 81 host-associated strains based on nucleotide sequences from the ftsZ gene, comprising 867 nucleotide positions, with posterior probabilities shown on nodes.

Supplementary Table S8. Genome-wide percent amino acid identities (AAI), showing mean and standard deviation and number of proteins compared, generated with enveomics toolbox.

	wPni	wFol	wDim	wPipPel
wPpe	66.72 (s.d. 15.3) n= 554	66.20 (s.d. 14.5) n= 658	66.19 (s.d. 16.2) n= 609	66.96 (s.d. 13.3) n= 697
wPni		71.45 (s.d. 16.1) n= 555	69.76 (s.d. 13.2) n= 499	71.04 (s.d. 15.0) n= 585
wFol			73.56 (s.d. 12.7) n= 614	74.32 (s.d. 15.9) n= 794
wDim				76.44 (s.d. 11.1) n= 667

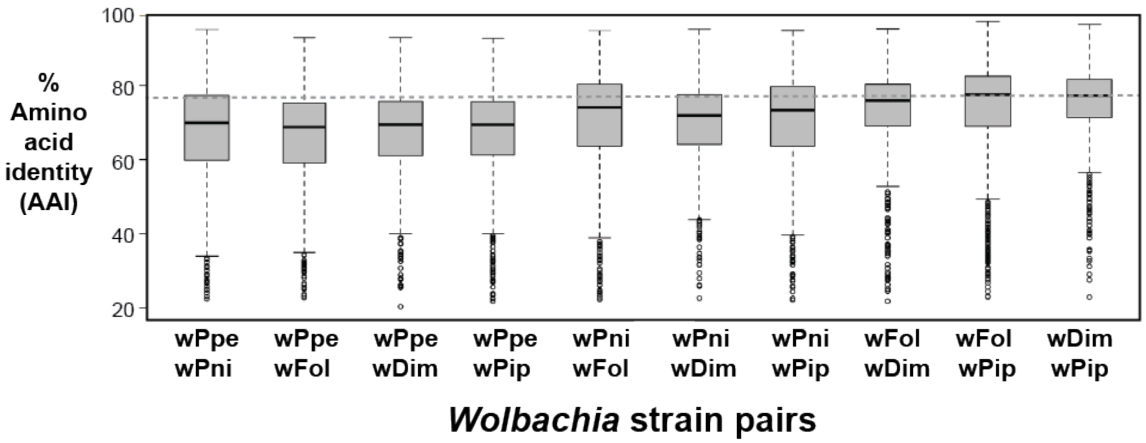

Supplementary Figure S22. Genome-wide percent amino acid identities (AAI), showing box-and-whisker plot distributions for pairwise comparisons, generated with enveomics toolbox. Dotted line shows the median for wPip (group B) versus wDim (group C).

Supplementary Table S9. List of genes from Venn diagrams (Figure 5), showing functional data (KEGG/InterPro/EcoCyc pathway) and categories of orthologous genes (COG). CDS = hypothetical protein with no homolog in current databases. COGs: C = Energy production and conversion, D = Cell cycle control, cell division, chromosome partitioning, E = Amino acid transport and metabolism, F = Nucleotide transport and metabolism, G = Carbohydrate transport and metabolism, H = Coenzyme transport and metabolism, I = Lipid transport and metabolism, J = Translation, ribosomal structure and biogenesis, K = Transcription, L = Replication, recombination and repair, M = Cell wall/membrane/envelope biogenesis, N = Cell motility, O = Posttranslational modification, protein turnover, chaperones, P = Inorganic ion transport and metabolism, Q = Secondary metabolites biosynthesis, transport and catabolism, R = General function prediction only, S = Function unknown, T = Signal transduction mechanisms, U = Intracellular trafficking, secretion, and vesicular transport, V = Defense mechanisms. Gene ID numbers refer to those in the annotations from the current study.

CORE Venn: 235 wPpe Only				
**ID**	**Gene Symbol**	**Gene Product**	**Pathway or Process**	**COG**
Ppee_00008	**-**	CDS	-	**S**
Ppee_00012	**-**	CDS	-	**S**
Ppee_00013	**-**	CDS	-	**S**
Ppee_00015	**-**	Ankyrin repeats (3 copies)	Ankyrin	**R**
Ppee_00018	**rpmG**	50S ribosomal protein L33	Ribosome	**J**
Ppee_00019	**ankX**	Phosphocholine transferase AnkX	Lipopolysaccharide biosynthesis	**I**
Ppee_00020	**plsC**	1-acyl-sn-glycerol-3-phosphate acyltransferase	Glycerolipid metabolism	**I**
Ppee_00036	**-**	CDS	-	**S**
Ppee_00038	**asd2**	Aspartate-semialdehyde dehydrogenase 2	Lysine biosynthesis	**E**
Ppee_00040	**dapA**	4-hydroxy-tetrahydrodipicolinate synthase	Lysine biosynthesis	**E**
Ppee_00041	**gltA**	Citrate synthase	Biosynthesis of amino acids	**E**
Ppee_00042	**rpsT**	30S ribosomal protein S20	Ribosome	**J**
Ppee_00043	**-**	CDS	-	**S**
Ppee_00044	**-**	CDS	-	**S**
Ppee_00045	**argS**	Arginine--tRNA ligase	Aminoacyl-tRNA biosynthesis	**J**
Ppee_00057	**ttg2**	Toluene tolerance, Ttg2	ABC transporters	**P**
Ppee_00058	**pasT**	Persistence and stress-resistance toxin PasT	Defense	**V**
Ppee_00059	**-**	CDS	-	**S**
Ppee_00062	**-**	CDS	-	**S**
Ppee_00070	**tsaC**	Threonylcarbamoyl-AMP synthase	Translation	**J**
Ppee_00074	**tatA**	Sec-independent protein translocase protein TatA	Bacterial secretion system	**U**
Ppee_00083	**-**	CDS	-	**S**
Ppee_00086	**-**	CDS	-	**S**
Ppee_00090	**-**	CDS	-	**S**
Ppee_00094	**-**	CDS	-	**S**
Ppee_00101	**-**	PD-(D/E)XK nuclease family transposase	Transposition	**L**
Ppee_00102	**-**	PD-(D/E)XK nuclease family transposase	Transposition	**L**
Ppee_00107	**sodB**	Superoxide dismutase [Fe]	FoxO signaling pathway	**L**
Ppee_00110	**pgpA**	Phosphatidylglycerophosphatase A	Glycerophospholipid metabolism	**I**
Ppee_00111	**-**	Acetyltransferase (GNAT) family protein	Stress response	**R**
Ppee_00112	**divL**	Sensor protein DivL	Signal transduction	**U**
Ppee_00117	**hemE**	Uroporphyrinogen decarboxylase	Heme biosynthesis	**H**
Ppee_00120	**-**	CDS	-	**S**
Ppee_00126	**-**	CDS	-	**S**
Ppee_00127	**ccmH**	Cytochrome c-type biogenesis protein CcmH precursor	Oxidative phosphorylation	**O**
Ppee_00130	**znuA**	High-affinity zinc uptake system protein ZnuA precursor	ABC transporters	**P**
Ppee_00133	**-**	Putative multidrug export ATP-binding/permease protein	Bacterial secretion system	**U**
Ppee_00134	**ankX**	Phosphocholine transferase AnkX	Lipopolysaccharide biosynthesis	**I**
Ppee_00135	**-**	Putative multidrug export ATP-binding/permease protein	Bacterial secretion system	**U**
Ppee_00136	**-**	CDS	-	**S**
Ppee_00137	**-**	CDS	-	**S**
Ppee_00138	**-**	CDS	-	**S**
Ppee_00139	**-**	CDS	-	**S**
Ppee_00141	**ppa**	Inorganic pyrophosphatase	Oxidative phosphorylation	**C**
Ppee_00143	**-**	CDS	-	**S**
Ppee_00144	**-**	CDS	-	**S**
Ppee_00145	**-**	PQ loop repeat protein	-	**R**
Ppee_00146	**-**	CDS	-	**S**
Ppee_00155	**-**	CDS	-	**S**
Ppee_00156	**tsaD**	tRNA N6-adenosine threonylcarbamoyltransferase	Translation	**J**
Ppee_00157	**def**	Peptide deformylase	Protein modification	**J**
Ppee_00161	**-**	CDS	-	**S**
Ppee_00168	**-**	CDS	-	**S**
Ppee_00173	**-**	CDS	-	**S**
Ppee_00177	**gntX**	DNA utilization protein GntX	Nucleoside catabolic process	**O**
Ppee_00180	**fmt**	Methionyl-tRNA formyltransferase	Aminoacyl-tRNA biosynthesis	**J**
Ppee_00181	**-**	CDS	-	**S**
Ppee_00182	**cysW**	Sulfate transport system permease protein CysW	Sulfur metabolism	**P**
Ppee_00184	**-**	CDS	-	**S**
Ppee_00185	**-**	CDS	-	**S**
Ppee_00190	**-**	CDS	-	**S**
Ppee_00192	**ankX**	Phosphocholine transferase AnkX	Lipopolysaccharide biosynthesis	**I**
Ppee_00193	**-**	Ankyrin repeats (3 copies)	Ankyrin	**R**
Ppee_00195	**-**	CDS	-	**S**
Ppee_00196	**-**	CDS	-	**S**
Ppee_00197	**-**	CDS	-	**S**
Ppee_00198	**-**	DNA mismatch repair protein	Mismatch repair	**L**
Ppee_00199	**-**	CDS	-	**S**
Ppee_00201	**-**	Apocarotenoid-15,15'-oxygenase	Retinal biosynthesis	**H**
Ppee_00204	**iscS**	Cysteine desulfurase	Alanine biosynthesis	**E**
Ppee_00206	**-**	CDS	-	**S**
Ppee_00208	**-**	3-methyladenine DNA glycosylase	Response to damage	**L**
Ppee_00210	**-**	CDS	-	**S**
Ppee_00211	**-**	CDS	-	**S**
Ppee_00220	**-**	CDS	-	**S**
Ppee_00225	**rpsD**	30S ribosomal protein S4	Ribosome	**J**
Ppee_00228	**-**	CDS	-	**S**
Ppee_00229	**-**	Ankyrin repeat protein	Ankyrin	**R**
Ppee_00230	**-**	Ankyrin repeat protein	Ankyrin	**R**
Ppee_00231	**-**	Ankyrin repeat protein	Ankyrin	**R**
Ppee_00232	**-**	CDS	-	**S**
Ppee_00237	**-**	CDS	-	**S**
Ppee_00238	**-**	CDS	-	**S**
Ppee_00239	**ctrA**	Cell cycle response regulator CtrA	Cell cycle control	**D**

Ppee_00243	**purA**	Adenylosuccinate synthetase	Alanine, aspartate and glutamate metabolism	**F**
Ppee_00251	**-**	CDS	-	**S**
Ppee_00252	**-**	Ankyrin repeats (3 copies)	Ankyrin	**R**
Ppee_00253	**-**	CDS	-	**S**
Ppee_00254	**-**	CDS	-	**S**
Ppee_00255	**-**	CDS	-	**S**
Ppee_00261	**opuAA**	Glycine betaine transport ATP-binding protein OpuAA	ABC transporters	**E**
Ppee_00263	**-**	CDS	-	**S**
Ppee_00266	**-**	CDS	-	**S**
Ppee_00267	**ccmE**	Cytochrome c-type biogenesis protein CcmE	Oxidative phosphorylation	**O**
Ppee_00273	**nfo**	Endonuclease 4	Base excision repair	**L**
Ppee_00277	**-**	CDS	-	**S**
Ppee_00280	**-**	Saccharopine dehydrogenase	Oxidoreductase	**C**
Ppee_00281	**-**	CDS	-	**S**
Ppee_00282	**-**	CDS	-	**S**
Ppee_00283	**-**	CDS	-	**S**
Ppee_00284	**-**	CDS	-	**S**
Ppee_00285	**-**	CDS	-	**S**
Ppee_00293	**-**	CDS	-	**S**
Ppee_00297	**yjdL**	putative dipeptide and tripeptide permease YjdL	Chaperone	**O**
Ppee_00302	**-**	CDS	-	**S**
Ppee_00303	**-**	CDS	-	**S**
Ppee_00304	**-**	RDD family protein	-	**R**
Ppee_00306	**-**	CDS	-	**S**
Ppee_00309	**-**	CDS	-	**S**
Ppee_00315	**-**	CDS	-	**S**
Ppee_00316	**-**	CDS	-	**S**
Ppee_00317	**-**	Anaphase-promoting complex, cyclosome, subunit 3	Protein-complex assembly	**R**
Ppee_00318	**-**	CDS	-	**S**
Ppee_00319	**-**	CDS	-	**S**
Ppee_00320	**-**	CDS	-	**S**
Ppee_00323	**-**	CDS	-	**S**
Ppee_00324	**-**	CDS	-	**S**
Ppee_00325	**-**	CDS	-	**S**
Ppee_00329	**-**	CDS	-	**S**
Ppee_00331	**lip-1**	Lipase 1 precursor	Fatty acid metabolism	**I**
Ppee_00333	**-**	Alpha/beta hydrolase family protein	-	**R**
Ppee_00335	**-**	CDS	-	**S**
Ppee_00336	**-**	CDS	-	**S**
Ppee_00337	**rmuC**	DNA recombination protein RmuC	Recombination	**R**
Ppee_00338	**-**	RmuC family protein	-	**R**
Ppee_00339	**-**	CDS	-	**S**
Ppee_00340	**rluC**	Ribosomal large subunit pseudouridine synthase C	Translation	**J**
Ppee_00341	**rluC**	Ribosomal large subunit pseudouridine synthase C	Translation	**J**
Ppee_00342	**hda**	DnaA regulatory inactivator Hda	Replication	**O**
Ppee_00343	**sucB**	Dihydrolipoyllysine-residue succinyltransferase component of 2-oxoglutarate dehydrogenase complex	Carbon metabolism	**C**
Ppee_00344	**-**	CDS	-	**S**
Ppee_00345	**-**	CDS	-	**S**
Ppee_00346	**hemC**	Porphobilinogen deaminase	Heme biosynthesis	**H**
Ppee_00347	**-**	CDS	-	**S**
Ppee_00348	**-**	CDS	-	**S**
Ppee_00349	**-**	CDS	-	**S**
Ppee_00350	**-**	CDS	-	**S**
Ppee_00351	**-**	NUDIX domain protein	Nucleoside modification	**L**
Ppee_00352	**cysS**	cysteinyl-tRNA synthetase	Aminoacyl-tRNA biosynthesis	**J**
Ppee_00353	**-**	CDS	-	**S**
Ppee_00354	**-**	CDS	-	**S**
Ppee_00355	**-**	CDS	-	**S**
Ppee_00356	**-**	CDS	-	**S**
Ppee_00357	**-**	CDS	-	**S**
Ppee_00358	**-**	CDS	-	**S**
Ppee_00359	**-**	CDS	-	**S**
Ppee_00360	**-**	CDS	-	**S**
Ppee_00363	**dnaA**	Chromosomal replication initiator protein DnaA	Two-component system	**L**
Ppee_00366	**-**	CDS	-	**S**
Ppee_00367	**-**	Putative multidrug export ATP-binding/permease protein	Bacterial secretion system	**U**
Ppee_00368	**folD**	Bifunctional protein FolD protein	Carbon metabolism	**H**
Ppee_00374	**hopD**	Squalene/phytoene synthase	Bacterial secretion system	**O**
Ppee_00377	**-**	CDS	-	**S**
Ppee_00378	**-**	CDS	-	**S**
Ppee_00379	**pth**	Peptidyl-tRNA hydrolase	Aminoacyl-tRNA hydrolase	**J**
Ppee_00389	**-**	Zinc-finger domain protein	-	**P**
Ppee_00390	**coaD**	Phosphopantetheine adenylyltransferase	Pantothenate and CoA biosynthesis	**H**
Ppee_00395	**-**	CDS	-	**S**
Ppee_00396	**-**	CDS	-	**S**
Ppee_00397	**miaA**	tRNA dimethylallyltransferase	Translation	**J**
Ppee_00398	**-**	CDS	-	**S**
Ppee_00399	**-**	CDS	-	**S**
Ppee_00400	**-**	CDS	-	**S**
Ppee_00403	**ruvA**	Holliday junction ATP-dependent DNA helicase RuvA	Homologous recombination	**L**
Ppee_00412	**fimC**	periplasmic chaperone	Chaperone	**O**
Ppee_00413	**fabZ**	3-hydroxyacyl-[acyl-carrier-protein] dehydratase FabZ	Fatty acid biosynthesis	**I**
Ppee_00415	**-**	CDS	-	**S**
Ppee_00416	**-**	CDS	-	**S**
Ppee_00419	**-**	anaerobic benzoate catabolism transcriptional regulator	DNA binding	**K**
Ppee_00420	**msp4**	Major surface antigen 4 precursor	Defense	**V**

Ppee_00421	**-**	CDS	-	**S**
Ppee_00432	**rpsJ**	30S ribosomal protein S10	Ribosome	**J**
Ppee_00433	**rplC**	50S ribosomal protein L3	Ribosome	**J**
Ppee_00434	**rplD**	50S ribosomal protein L4	Ribosome	**J**
Ppee_00435	**rplW**	50S ribosomal protein L23	Ribosome	**J**
Ppee_00436	**tuf**	elongation factor Tu	Translation	**J**
Ppee_00444	**-**	CDS	-	**S**
Ppee_00445	**-**	CDS	-	**S**
Ppee_00446	**-**	CDS	-	**S**
Ppee_00447	**-**	CDS	-	**S**
Ppee_00448	**-**	CDS	-	**S**
Ppee_00449	**-**	CDS	-	**S**
Ppee_00450	**-**	CDS	-	**S**
Ppee_00453	**xerC**	Tyrosine recombinase XerC	Recombination	**L**
Ppee_00455	**bamB**	outer membrane biogenesis protein BamB	Membrane assembly	**M**
Ppee_00457	**-**	PD-(D/E)XK nuclease family transposase	Transposition	**L**
Ppee_00458	**-**	Ankyrin repeats (3 copies)	Ankyrin	**R**
Ppee_00461	**-**	CDS	-	**S**
Ppee_00465	**-**	CDS	-	**S**
Ppee_00467	**mrpA**	Na(+)/H(+) antiporter subunit A	Antiporter	**P**
Ppee_00469	**mnmE**	tRNA modification GTPase MnmE	Translation	**J**
Ppee_00474	**-**	CDS	-	**S**
Ppee_00478	**coaE**	Dephospho-CoA kinase	Pantothenate and CoA biosynthesis	**H**
Ppee_00479	**proP**	Proline/betaine transporter	Transporter/osmoregulator	**GEP**
Ppee_00481	**-**	CDS	-	**S**
Ppee_00491	**tig**	Trigger factor	Chaperone	**O**
Ppee_00492	**clpP**	ATP-dependent Clp protease proteolytic subunit	Misfolded protein degradation	**O**
Ppee_00493	**clpP**	ATP-dependent Clp protease proteolytic subunit	Misfolded protein degradation	**O**
Ppee_00497	**-**	Ankyrin repeats (3 copies)	Ankyrin	**R**
Ppee_00499	**-**	CDS	-	**S**
Ppee_00502	**grpE**	heat shock protein GrpE	Stress response	**O**
Ppee_00503	**trpS**	Tryptophan--tRNA ligase	Aminoacyl-tRNA biosynthesis	**J**
Ppee_00504	**-**	CDS	-	**S**
Ppee_00506	**-**	CDS	-	**S**
Ppee_00514	**-**	CDS	-	**S**
Ppee_00524	**-**	CDS	-	**S**
Ppee_00528	**-**	Lipase (class 3)	Fatty acid metabolism	**I**
Ppee_00531	**-**	CDS	-	**S**
Ppee_00533	**-**	RDD family protein	-	**R**
Ppee_00537	**pcrA**	ATP-dependent DNA helicase PcrA	Replication	**L**
Ppee_00539	**-**	CDS	-	**S**
Ppee_00540	**bamA**	Outer membrane protein assembly factor BamA precursor	Membrane assembly	**M**
Ppee_00542	**secB**	Protein-export protein SecB	Bacterial secretion system	**U**
Ppee_00546	**-**	CDS	-	**S**
Ppee_00547	**-**	phosphoribosylformylglycinamidine synthase II	Purine metabolism	**L**
Ppee_00548	**-**	Putative pterin-4-alpha-carbinolamine dehydratase	Bacterial secretion system	**U**
Ppee_00549	**ispB**	Octaprenyl-diphosphate synthase	Terpenoid backbone biosynthesis	**H**
Ppee_00553	**-**	CDS	-	**S**
Ppee_00558	**-**	CDS	-	**S**
Ppee_00564	**-**	CDS	-	**S**
Ppee_00571	**-**	VirB8 protein	Bacterial secretion system	**U**
Ppee_00572	**-**	Ankyrin repeat protein	Ankyrin	**R**
Ppee_00578	**-**	CDS	-	**S**
Ppee_00579	**-**	CDS	-	**S**
Ppee_00580	**-**	CDS	-	**S**
Ppee_00584	**cycM**	Cytochrome c-552	Oxidative phosphorylation	**C**
Ppee_00588	**-**	CDS	-	**S**
Ppee_00590	**tldD**	protease TldD	Metalloprotease/peptide lysis	**R**
Ppee_00591	**-**	CDS	-	**S**
Ppee_00595	**-**	CDS	-	**S**
Ppee_00596	**polA**	DNA polymerase I, thermostable	Purine metabolism	**L**
Ppee_00601	**-**	CDS	-	**S**
Ppee_00603	**nuoJ**	NADH-quinone oxidoreductase subunit J	Oxidative phosphorylation	**C**
Ppee_00606	**nuoM**	NADH-quinone oxidoreductase subunit M	Oxidative phosphorylation	**C**
Ppee_00607	**nuoN**	NADH-quinone oxidoreductase subunit N	Oxidative phosphorylation	**C**
		**CORE Venn: 81 A+B+wPpe**		
**ID**	**Gene Symbol**	**Gene Product**	**Pathway or Process**	**COG**
AlbB_00006	**-**	CDS	-	**S**
AlbB_00007	**recO**	DNA repair protein RecO	Homologous recombination	**L**
AlbB_00055	**ruvC**	Crossover junction endodeoxyribonuclease RuvC	Homologous recombination	**L**
AlbB_00059	**metK**	S-adenosylmethionine synthase	Biosynthesis of amino acids	**E**
AlbB_00093	**rpoA**	DNA-directed RNA polymerase subunit alpha	Purine metabolism	**K**
AlbB_00122	**-**	SPFH domain / Band 7 family protein	General function prediction only	**R**
AlbB_00126	**-**	CDS	-	**S**
AlbB_00152	**glmS**	Glutamine--fructose-6-phosphate aminotransferase [isomerizing]	Alanine, aspartate and glutamate metabolism	**M**
AlbB_00172	**-**	Surface antigen	Defense	**R**
AlbB_00176	**-**	CDS	-	**S**
AlbB_00214	**tolB**	translocation protein TolB	Cell wall/membrane/envelope biogenesis	**M**
AlbB_00227	**bfr**	bacterioferritin	Porphyrin metabolism	**P**
AlbB_00243	**mdtM**	Multidrug resistance protein MdtM	Posttranslational modification, protein turnover, chaperones	**O**
AlbB_00264	**rluC**	Ribosomal large subunit pseudouridine synthase C	Translation, ribosomal structure and biogenesis	**J**
AlbB_00286	**ruvA**	Holliday junction ATP-dependent DNA helicase RuvA	Homologous recombination	**L**
AlbB_00287	**ruvA**	Holliday junction ATP-dependent DNA helicase RuvA	Homologous recombination	**L**

AlbB_00290	**-**	recombinase A	Recombination	**L**
AlbB_00292	**sspB**	ClpXP protease specificity-enhancing factor	General function prediction only	**R**
AlbB_00320	**argD**	Acetylornithine aminotransferase	Arginine biosynthesis	**E**
AlbB_00321	**pdhS**	Cell-division control histidine kinase PdhS	Energy production and conversion	**C**
AlbB_00375	**recF**	DNA replication and repair protein RecF	Homologous recombination	**L**
AlbB_00383	**-**	CDS	-	**S**
AlbB_00384	**rluC**	Ribosomal large subunit pseudouridine synthase C	Translation, ribosomal structure and biogenesis	**J**
AlbB_00409	**yciK**	putative oxidoreductase YciK	General function prediction only	**R**
AlbB_00439	**fgs**	Folylpolyglutamate synthase	Folate biosynthesis	**H**
AlbB_00442	**-**	CDS	-	**S**
AlbB_00462	**idiA**	Iron deficiency-induced protein A precursor	Inorganic ion transport and metabolism	**P**
AlbB_00469	**-**	CDS	-	**S**
AlbB_00474	**ankX**	Phosphocholine transferase AnkX	Lipopolysaccharide biosynthesis	
AlbB_00503	**-**	Sodium:dicarboxylate symporter family protein	Carbohydrate transport and metabolism	**G**
AlbB_00566	**dxr**	1-deoxy-D-xylulose 5-phosphate reductoisomerase	Terpenoid backbone biosynthesis	**I**
AlbB_00571	**-**	CDS	-	**S**
AlbB_00584	**egsA**	Glycerol-1-phosphate dehydrogenase [NAD(P)+]	Glycerophospholipid metabolism	**C**
AlbB_00623	**pdxH**	Pyridoxine/pyridoxamine 5'-phosphate oxidase	Pyridoxal-5'-phosphate biosynthesis	**C**
AlbB_00627	**mlaD**	putative phospholipid ABC transporter-binding protein MlaD	ABC transporters	**O**
AlbB_00660	**ycf3**	photosystem I assembly protein Ycf3	Energy production and conversion	**C**
AlbB_00661	**-**	CDS	-	**S**
AlbB_00662	**bioY**	Biotin transporter BioY	Cofactor transport	**H**
AlbB_00668	**aspC**	Aspartate aminotransferase	Asparagine biosynthesis	**E**
AlbB_00675	**-**	CDS	-	**S**
AlbB_00685	**-**	CDS	-	**S**
AlbB_00694	**ispH**	4-hydroxy-3-methylbut-2-enyl diphosphate reductase	Terpenoid backbone biosynthesis	**I**
AlbB_00695	**-**	CDS	-	**S**
AlbB_00696	**-**	CDS	-	**S**
AlbB_00716	**ycfH**	putative deoxyribonuclease YcfH	Fanconi anemia pathway	**L**
AlbB_00741	**zapA**	Cell division protein ZapA	Cell cycle control, cell division, chromosome partitioning	**D**
AlbB_00742	**hup**	DNA-binding protein HU	Posttranslational modification, protein turnover, chaperones	**O**
AlbB_00744	**trmB**	tRNA (guanine-N(7)-)-methyltransferase	Translation, ribosomal structure and biogenesis	**J**
AlbB_00752	**rsmA**	Ribosomal RNA small subunit methyltransferase A	Translation, ribosomal structure and biogenesis	**J**
AlbB_00759	**parB**	putative chromosome-partitioning protein ParB	Replication, recombination and repair	**L**
AlbB_00768	**-**	KAP family P-loop domain protein	General function prediction only	**R**
AlbB_00770	**recR**	Recombination protein RecR	Homologous recombination	**L**
AlbB_00776	**-**	CDS	-	**S**
AlbB_00782	**ispA**	Farnesyl diphosphate synthase	Terpenoid backbone biosynthesis	**H**
AlbB_00791	**argD**	Acetylornithine aminotransferase	Arginine biosynthesis	**E**
AlbB_00800	**-**	CDS	-	**S**
AlbB_00801	**ihfA**	Integration host factor subunit alpha	Replication, recombination and repair	**L**
AlbB_00832	**pgdA**	Peptidoglycan-N-acetylglucosamine deacetylase	Cell wall/membrane/envelope biogenesis	**M**
AlbB_00833	**comM**	Competence protein ComM	Replication, recombination and repair	**L**
AlbB_00876	**murJ**	putative peptidoglycan biosynthesis protein MurJ	General function prediction only	**R**
AlbB_00888	**-**	CDS	-	**S**
AlbB_00893	**pdxJ**	Pyridoxine 5'-phosphate synthase	Vitamin B metabolism	**H**
AlbB_00896	**-**	CDS	-	**S**
AlbB_00973	**ykfA**	putative murein peptide carboxypeptidase	Phenylpropanoid biosynthesis	**R**
AlbB_00982	**rmuC**	DNA recombination protein RmuC	Recombination	**L**
AlbB_00992	**-**	CDS	-	**S**
AlbB_01005	**gltP**	Proton glutamate symport protein	Energy production and conversion	**C**
AlbB_01021	**hofQ**	outer membrane porin HofQ	Intracellular trafficking, secretion, and vesicular transport	**U**
AlbB_01036	**-**	CDS	-	**S**
AlbB_01060	**rsmA**	Ribosomal RNA small subunit methyltransferase A	Translation, ribosomal structure and biogenesis	**J**
AlbB_01104	**-**	Anaphase-promoting complex, cyclosome, subunit 3	Protein-complex assembly	**R**
AlbB_01109	**clpA**	ATP-dependent Clp protease ATP-binding subunit ClpA	Posttranslational modification, protein turnover, chaperones	**O**
AlbB_01122	**pcaH**	Protocatechuate 3,4-dioxygenase beta chain	Secondary metabolites biosynthesis, transport and catabolism	**Q**
Anaa_00537	**-**	CDS	-	**S**
Anaa_00761	**rpmF**	50S ribosomal protein L32	Ribosome	**J**
Anaa_00762	**mlaE**	putative phospholipid ABC transporter permease protein MlaE	ABC transporters	**O**
Auuu_00015	**tuf**	elongation factor Tu	Translation	**J**
Auuu_00252	**-**	PD-(D/E)XK nuclease family transposase	Transposition	**L**
Cocc_00685	**recG**	ATP-dependent DNA helicase RecG	Homologous recombination	**LK**
Ppee_00630	**-**	CDS	-	**S**
Riii_00570	**-**	CDS	-	**S**

CORE Venn: 82 C+D+F+wPpe

**ID**	**Gene Symbol**	**Gene Product**	**Pathway or Process**	**COG**
AlbB_00026	**polA**	DNA polymerase I, thermostable	Purine metabolism	**L**
AlbB_00028	**-**	CDS	-	**S**
AlbB_00029	**xthA**	Exodeoxyribonuclease III	Base excision repair	**L**
AlbB_00069	**rpsJ**	30S ribosomal protein S10	Ribosome	**J**
AlbB_00070	**rplC**	50S ribosomal protein L3	Ribosome	**J**
AlbB_00071	**rplD**	50S ribosomal protein L4	Ribosome	**J**
AlbB_00072	**rplW**	50S ribosomal protein L23	Ribosome	**J**
AlbB_00073	**rplB**	50S ribosomal protein L2	Ribosome	**J**
AlbB_00087	**rpsE**	30S ribosomal protein S5	Ribosome	**J**
AlbB_00088	**rplO**	50S ribosomal protein L15	Ribosome	**J**

AlbB_00140	**argS**	Arginine--tRNA ligase	Aminoacyl-tRNA biosynthesis	**J**
AlbB_00210	**rpsD**	30S ribosomal protein S4	Ribosome	**J**
AlbB_00219	**-**	phosphoribosylformylglycinamidine synthase II	Purine metabolism	**R**
AlbB_00249	**engB**	putative GTP-binding protein EngB	Cell cycle control, cell division, chromosome partitioning	**D**
AlbB_00252	**purA**	Adenylosuccinate synthetase	Alanine, aspartate and glutamate metabolism	**F**
AlbB_00265	**proP**	Proline/betaine transporter	Mulitple COGs	**GEP**
AlbB_00274	**ccmE**	Cytochrome c-type biogenesis protein CcmE	Oxidative phosphorylation	**C**
AlbB_00275	**-**	CDS	-	**S**
AlbB_00277	**nuoN**	NADH-quinone oxidoreductase subunit N	Oxidative phosphorylation	**C**
AlbB_00278	**ndhD1**	NAD(P)H-quinone oxidoreductase chain 4 1	Carbohydrate transport and metabolism	**G**
AlbB_00281	**nuoJ**	NADH-quinone oxidoreductase subunit J	Oxidative phosphorylation	**C**
AlbB_00283	**pcrA**	ATP-dependent DNA helicase PcrA	Replication	**L**
AlbB_00302	**gltA**	Citrate synthase	Biosynthesis of amino acids	**C**
AlbB_00303	**rpsT**	30S ribosomal protein S20	Ribosome	**J**
AlbB_00304	**yihG**	putative acyltransferase YihG	Glycosylphosphatidylinositol(GPI)-anchor biosynthesis	**R**
AlbB_00307	**hda**	DnaA regulatory inactivator Hda	Replication	**O**
AlbB_00310	**trpS**	Tryptophan--tRNA ligase	Aminoacyl-tRNA biosynthesis	**J**
AlbB_00323	**tsaD**	tRNA N6-adenosine threonylcarbamoyltransferase	Translation	**J**
AlbB_00351	**coaE**	Dephospho-CoA kinase	Pantothenate and CoA biosynthesis	**H**
AlbB_00363	**ppa**	Inorganic pyrophosphatase	Oxidative phosphorylation	**C**
AlbB_00389	**mrpA**	Na(+)/H(+) antiporter subunit A	Inorganic ion transport and metabolism	**P**
AlbB_00390	**-**	VirB8 protein	Bacterial secretion system	**U**
AlbB_00393	**tsaC**	Threonylcarbamoyl-AMP synthase	Translation	**J**
AlbB_00394	**-**	CDS	-	**S**
AlbB_00395	**miaA**	tRNA dimethylallyltransferase	Zeatin biosynthesis	**J**
AlbB_00402	**rpmG2**	50S ribosomal protein L33 2	Ribosome	**J**
AlbB_00404	**znuA**	High-affinity zinc uptake system protein ZnuA precursor	ABC transporters	**P**
AlbB_00424	**pth**	Peptidyl-tRNA hydrolase	Aminoacyl-tRNA hydrolase	**J**
AlbB_00436	**bdbD**	Disulfide bond formation protein D precursor	General function prediction only (R)	**R**
AlbB_00437	**pasT**	Persistence and stress-resistance toxin PasT	Stress tolerance	**V**
AlbB_00479	**tatA**	Sec-independent protein translocase protein TatA	Bacterial secretion system	**U**
AlbB_00495	**ccmH**	Cytochrome c-type biogenesis protein CcmH precursor	Oxidative phosphorylation	**C**
AlbB_00496	**-**	CDS	-	**S**
AlbB_00517	**coaD**	Phosphopantetheine adenylyltransferase	Pantothenate and CoA biosynthesis	**H**
AlbB_00518	**-**	CDS	-	**S**
AlbB_00545	**-**	CDS	-	**S**
AlbB_00559	**pyrF**	Orotidine 5'-phosphate decarboxylase	Pyrimidine metabolism	**F**
AlbB_00600	**nuoF**	NADH-quinone oxidoreductase subunit F	Oxidative phosphorylation	**C**
AlbB_00604	**mnmE**	tRNA modification GTPase MnmE	Homologous recombination	**J**
AlbB_00631	**iscS**	Cysteine desulfurase	Alanine biosynthesis	**E**
AlbB_00653	**tldD**	protease TldD	Metalloprotease/peptide lysis	**R**
AlbB_00667	**hemE**	Uroporphyrinogen decarboxylase	Heme biosynthesis	**H**
AlbB_00699	**ssb**	Single-stranded DNA-binding protein	DNA replication	**L**
AlbB_00700	**dapA**	4-hydroxy-tetrahydrodipicolinate synthase	Lysine biosynthesis	**EM**
AlbB_00735	**gltB**	Glutamate synthase [NADPH] small chain	Biosynthesis of amino acids	**E**
AlbB_00736	**fabZ**	3-hydroxyacyl-[acyl-carrier-protein] dehydratase FabZ	Fatty acid biosynthesis	**I**
AlbB_00737	**-**	Outer membrane protein (OmpH-like)	Cell wall/membrane/envelope biogenesis	**M**
AlbB_00749	**sucC**	Succinyl-CoA ligase [ADP-forming] subunit beta	C5-Branched dibasic acid metabolism	**C**
AlbB_00750	**rpsU**	30S ribosomal protein S21	Ribosome	**J**
AlbB_00840	**divL**	Sensor protein DivL	Signal transduction	**U**
AlbB_00841	**-**	acetyltransferase	General function prediction only (R)	**R**
AlbB_00844	**dut**	Deoxyuridine 5'-triphosphate nucleotidohydrolase	Pyrimidine metabolism	**F**
AlbB_00857	**ctrA**	Cell cycle response regulator CtrA	Cell cycle control	**D**
AlbB_00880	**sodB**	Superoxide dismutase [Fe]	FoxO signaling pathway	
AlbB_00883	**tgt**	queuine tRNA-ribosyltransferase	Translation, ribosomal structure and biogenesis (J)	**J**
AlbB_00892	**hupB**	DNA-binding protein HRm	Posttranslational modification, protein turnover, chaperones	**O**
AlbB_00924	**cysW**	Sulfate transport system permease protein CysW	Sulfur metabolism	**P**
AlbB_00929	**adiC**	Arginine/agmatine antiporter	Arginine transport	**E**
AlbB_00998	**ftsQ**	cell division protein FtsQ	Cell division	**M**
AlbB_01053	**grpE**	heat shock protein GrpE	Chaperone	**O**
AlbB_01054	**ispB**	Octaprenyl-diphosphate synthase	Terpenoid backbone biosynthesis	**H**
AlbB_01080	**hflC**	Modulator of FtsH protease HflC	Protein modification	**O**
AlbB_01081	**hflK**	Modulator of FtsH protease HflK	Protein modification	**O**
AlbB_01098	**-**	Succinate dehydrogenase/Fumarate reductase transmembrane subunit	General function prediction only (R)	**R**
AlbB_01099	**-**	CDS	-	**S**
AlbB_01119	**secB**	Protein-export protein SecB	Bacterial secretion system	**U**
Anaa_01502	**-**	3-methyladenine DNA glycosylase	Response to damage	**L**
Anaa_01533	**-**	CDS	-	**S**
Bmmm_00063	**-**	CDS	-	**S**
Clee_00705	**iscS**	Cysteine desulfurase	Alanine biosynthesis	**E**
Cocc_01265	**valS**	Valine--tRNA ligase	Aminoacyl-tRNA biosynthesis	**J**
Haaa_00814	**-**	CDS	-	**S**

CORE Venn: 24 A+B+C+D+F

**ID**	**Gene Symbol**	**Gene Product**	**Pathway or Process**	**COG**
AlbB_00011	**clpB**	Chaperone protein ClpB	Chaperone	**O**
AlbB_00031	**-**	CDS	-	**S**
AlbB_00123	**mepM**	Murein DD-endopeptidase MepM	Cell wall assembly	**O**
AlbB_00183	**iscA**	Iron-binding protein IscA	Iron-cluster assembly	**H**
AlbB_00190	**uvrA**	UvrABC system protein A	Nucleotide excision repair	**L**
AlbB_00373	**gshB**	Glutathione synthetase	Glutathione biosynthesis	**H**

AlbB_00388	**dnaB**	Replicative DNA helicase	DNA replication	**L**
AlbB_00492	**-**	CDS	-	**S**
AlbB_00505	-	CDS	-	**S**
AlbB_00551	**bamA**	Outer membrane protein assembly factor BamA precursor	Membrane assembly	**M**
AlbB_00569	**proS**	Proline--tRNA ligase	Aminoacyl-tRNA biosynthesis	**J**
AlbB_00611	-	CDS	-	**S**
AlbB_00622	**mutM**	Formamidopyrimidine-DNA glycosylase	Base excision repair	**L**
AlbB_00663	**-**	Putative NAD(P)H nitroreductase	Oxidative phosphorylation	**C**
AlbB_00812	**dusC**	tRNA-dihydrouridine synthase C	Nitrotoluene degradation	**J**
AlbB_00847	**ribH1**	6,7-dimethyl-8-ribityllumazine synthase 1	Riboflavin metabolism	**H**
AlbB_00861	-	CDS	-	**S**
AlbB_00909	**miaB**	(Dimethylallyl)adenosine tRNA methylthiotransferase MiaB	Puromycin biosynthesis	**J**
AlbB_00928	**-**	CDS	-	**S**
AlbB_00938	**uvrB**	UvrABC system protein B	Nucleotide excision repair	**L**
AlbB_01043	**pld**	Phospholipase D precursor	CAMP signaling pathway	**I**
AlbB_01108	**cshA**	DEAD-box ATP-dependent RNA helicase CshA	RNA degradation	**L**
AlbB_01132	**uvrC**	UvrABC system protein C	Nucleotide excision repair	**L**
Anaa_00655	**rpmC**	50S ribosomal protein L29	Ribosome	**J**

CORE Venn: 57 A+B Only

**ID**	**Gene Symbol**	**Gene Product**	**Pathway or Process**	**COG**
AlbB_00016	-	CDS	-	**S**
AlbB_00019	**alsT**	Amino-acid carrier protein AlsT	Amino acid transport	**E**
AlbB_00022	**-**	Ankyrin repeats (3 copies)	Ankyrin	**R**
AlbB_00033	**smc**	Chromosome partition protein Smc	Cell cycle control, cell division, chromosome partitioning	**D**
AlbB_00037	**-**	CDS	-	**S**
AlbB_00048	**-**	CDS	-	**S**
AlbB_00057	**tenA**	Thiaminase-2	Thiamine biosynthesis/salvage	**H**
AlbB_00095	**-**	CDS	-	**S**
AlbB_00129	**-**	BolA-like protein	Transcription	**R**
AlbB_00148	**lpxD**	UDP-3-O-[3-hydroxymyristoyl] glucosamine N-acyltransferase	Lipopolysaccharide biosynthesis	**M**
AlbB_00160	-	CDS	-	**S**
AlbB_00161	**trkG**	Trk system potassium uptake protein TrkG	Inorganic ion transport and metabolism	**P**
AlbB_00187	-	TrbC/VIRB2 family protein	Bacterial secretion system	**O**
AlbB_00194	-	CDS	-	**S**
AlbB_00311	-	CDS	-	**S**
AlbB_00317	-	CDS	-	**S**
AlbB_00347	-	CDS	-	**S**
AlbB_00348	-	Phage portal protein	Replication, recombination and repair	**L**
AlbB_00352	-	CDS	-	**S**
AlbB_00362	-	CDS	-	**S**
AlbB_00371	-	CDS	-	**S**
AlbB_00391	-	CDS	-	**S**
AlbB_00420	**proP**	Proline/betaine transporter	Transporter/osmoregulator	**GEP**
AlbB_00490	**-**	CDS	-	**S**
AlbB_00554	**-**	Cytochrome C and Quinol oxidase polypeptide I	Oxidative phosphorylation	**C**
AlbB_00572	**-**	CDS	-	**S**
AlbB_00669	**sugE**	Quaternary ammonium compound-resistance protein SugE	Energy production and conversion	**C**
AlbB_00714	**-**	CDS	-	**S**
AlbB_00722	-	CDS	-	**S**
AlbB_00754	**-**	CDS	-	**S**
AlbB_00758	**parA**	Chromosome partitioning protein ParA	Replication, recombination and repair	**L**
AlbB_00802	**gudP**	putative glucarate transporter	Posttranslational modification, protein turnover, chaperones	**O**
AlbB_00837	-	EVE domain protein	-	**R**
AlbB_00855	-	CDS	-	**S**
AlbB_00863	-	CDS	-	**S**
AlbB_00868	**rmlD**	RmlD substrate binding domain protein	Cell wall/membrane/envelope biogenesis	**M**
AlbB_00900	**icaA**	N-glycosyltransferase	General function prediction only	**R**
AlbB_00905	-	CDS	-	**S**
AlbB_00935	**-**	Dna-J like membrane chaperone protein	Chaperone	**O**
AlbB_00953	**mdtC**	Multidrug resistance protein MdtC	Posttranslational modification, protein turnover, chaperones	**O**
AlbB_00959	**-**	PqqC-like protein	General function prediction only	**R**
AlbB_00961	**folP**	Dihydropteroate synthase	Folate biosynthesis	**H**
AlbB_00962	**dfrA**	Dihydrofolate reductase	Folate biosynthesis	**H**
AlbB_00966	**-**	CDS	-	**S**
AlbB_00980	**-**	CDS	-	**S**
AlbB_01019	**-**	CDS	-	**S**
AlbB_01042	**-**	CDS	-	**S**
AlbB_01068	-	CDS	-	**S**
AlbB_01100	**algA**	Alginate biosynthesis protein AlgA	Carbohydrate transport and metabolism	**G**
Anaa_00143	**-**	CDS	-	**S**
Auuu_00253	**-**	anaerobic benzoate catabolism transcriptional regulator	DNA binding	**K**
Bmmm_00419	**ankX**	Phosphocholine transferase AnkX	Lipopolysaccharide biosynthesis	
Clee_00317	**-**	CDS	-	**S**
Cocc_00996	**-**	CDS	-	**S**
Haaa_00260	**-**	Ankyrin repeats (3 copies)	Ankyrin	**R**
Nooo_00156	**-**	CDS	-	**S**
Pipp_00196	**-**	Na+/H+ antiporter family protein	Energy production and conversion	**C**

CORE Venn: 10 C+D+F Only

**ID**	**Gene Symbol**	**Gene Product**	**Pathway or Process**	**COG**
AlbB_00256	**-**	CDS	-	**S**
AlbB_00429	**msp4**	Major surface antigen 4 precursor	Defense	**V**

AlbB_00433	**-**	CDS	-	**S**
AlbB_00845	**-**	CDS	-	**S**
AlbB_00907	**-**	CDS	-	**S**
AlbB_00923	**-**	putative rhodanese-related sulfurtransferase	Iron metabolism	**P**
AlbB_01031	**ribF**	Riboflavin biosynthesis protein RibF	Riboflavin biosynthesis	**H**
Anaa_00342	**icd**	Isocitrate dehydrogenase [NADP]	Biosynthesis of amino acids	**C**
Auuu_01065	**-**	CDS	-	**S**
Bmmm_00026	**-**	CDS	-	**S**

CORE Venn: 489 A+B+C+D+F+wPpe

ID	Gene Symbol	Gene Product	Pathway or Process	COG
AlbB_00001	**pyrC**	Dihydroorotase	Pyrimidine metabolism	**F**
AlbB_00008	**pleD**	Response regulator PleD	Signal transduction mechanisms	**T**
AlbB_00009	**-**	CDS	-	**S**
AlbB_00010	**gdh**	NAD-specific glutamate dehydrogenase	Arginine biosynthesis	**G**
AlbB_00013	**-**	CDS	-	**S**
AlbB_00017	**plsY**	putative glycerol-3-phosphate acyltransferase	Glycerolipid metabolism	**I**
AlbB_00018	**alsT**	Amino-acid carrier protein AlsT	Amino acid transport	**E**
AlbB_00020	**ynjF**	CDP-alcohol phosphatidyltransferase	General function prediction only	**R**
AlbB_00021	**-**	phosphatidylserine decarboxylase	Lipid transport and metabolism	**I**
AlbB_00024	**gyrB**	DNA gyrase subunit B	Replication, recombination and repair	**L**
AlbB_00025	**gatA**	Glutamyl-tRNA(Gln) amidotransferase subunit A	Glutamate biosynthesis	**E**
AlbB_00030	**hemD**	uroporphyrinogen-III synthase	Heme biosynthesis	**H**
AlbB_00034	**algC**	Phosphomannomutase/phosphoglucomutase	Glycolysis / Gluconeogenesis	**G**
AlbB_00035	**-**	Colicin V production protein	Extracellular toxin production	**V**
AlbB_00039	**pheS**	Phenylalanine--tRNA ligase alpha subunit	Aminoacyl-tRNA biosynthesis	**J**
AlbB_00040	**glmU**	Bifunctional protein GlmU	Amino sugar and nucleotide sugar metabolism	**M**
AlbB_00045	**atpC**	ATP synthase epsilon chain	Oxidative phosphorylation	**C**
AlbB_00047	**pstA**	Phosphate transport system permease protein PstA	ABC transporters	**P**
AlbB_00052	**ubiG**	bifunctional 3-demethylubiquinone-9 3-methyltransferase/ 2-octaprenyl-6-hydroxy phenol methylase	Ubiquinone and other terpenoid-quinone biosynthesis	**H**
AlbB_00053	**nrdZ**	Ribonucleoside-diphosphate reductase NrdZ	Nucleotide transport and metabolism	**F**
AlbB_00054	**-**	Putative Holliday junction resolvase	Replication, recombination and repair	**L**
AlbB_00056	**ctaE**	Cytochrome c oxidase subunit 3	Oxidative phosphorylation	**C**
AlbB_00061	**rpmA**	50S ribosomal protein L27	Ribosome	**J**
AlbB_00062	**rplU**	50S ribosomal protein L21	Ribosome	**J**
AlbB_00064	**tal**	putative transaldolase	Carbon metabolism	**C**
AlbB_00065	**-**	CDS	-	**S**
AlbB_00066	**carA**	Carbamoyl-phosphate synthase small chain	Arginine biosynthesis	**EF**
AlbB_00074	**rpsS**	30S ribosomal protein S19	Ribosome	**J**
AlbB_00075	**rplV**	50S ribosomal protein L22	Ribosome	**J**
AlbB_00076	**rpsC**	30S ribosomal protein S3	Ribosome	**J**
AlbB_00077	**rplP**	50S ribosomal protein L16	Ribosome	**J**
AlbB_00079	**rpsQ**	30S ribosomal protein S17	Ribosome	**J**
AlbB_00080	**rplN**	50S ribosomal protein L14	Ribosome	**J**
AlbB_00081	**rplX**	50S ribosomal protein L24	Ribosome	**J**
AlbB_00082	**rplE**	50S ribosomal protein L5	Ribosome	**J**
AlbB_00083	**rpsN**	30S ribosomal protein S14	Ribosome	**J**
AlbB_00084	**rpsH**	30S ribosomal protein S8	Ribosome	**J**
AlbB_00085	**rplF**	50S ribosomal protein L6	Ribosome	**J**
AlbB_00086	**rplR**	50S ribosomal protein L18	Ribosome	**J**
AlbB_00089	**secY**	preprotein translocase subunit SecY	Bacterial secretion system	**U**
AlbB_00090	**adk**	adenylate kinase	Purine metabolism	**F**
AlbB_00091	**rpsM**	30S ribosomal protein S13	Ribosome	**J**
AlbB_00092	**rpsK**	30S ribosomal protein S11	Ribosome	**J**
AlbB_00094	**rplQ**	50S ribosomal protein L17	Ribosome	**J**
AlbB_00096	**atpH**	ATP synthase subunit delta	Oxidative phosphorylation	**C**
AlbB_00097	**atpA**	ATP synthase subunit alpha	Oxidative phosphorylation	**C**
AlbB_00098	**greA**	Transcription elongation factor GreA	Transcription	**K**
AlbB_00099	**ribB**	3,4-dihydroxy-2-butanone 4-phosphate synthase	Riboflavin biosynthesis	**H**
AlbB_00100	**yfgC**	TPR repeat-containing protein YfgC precursor	General function prediction only	**R**
AlbB_00101	**-**	TrbC/VIRB2 family protein	Bacterial secretion system	**U**
AlbB_00102	**fabG**	3-oxoacyl-[acyl-carrier-protein] reductase FabG	Biosynthesis of unsaturated fatty acids	**I**
AlbB_00120	**ppdK**	Pyruvate, phosphate dikinase	Carbon metabolism	**C**
AlbB_00124	**-**	putative transcriptional regulatory protein	General function prediction only	**R**
AlbB_00125	**bamA**	Outer membrane protein assembly factor BamA precursor	Membrane assembly	**M**
AlbB_00127	**pgsA**	CDP-diacylglycerol--glycerol-3-phosphate 3-phosphatidyltransferase	Glycerophospholipid metabolism	**I**
AlbB_00128	**maeB**	NADP-dependent malic enzyme	Carbon metabolism	**C**
AlbB_00130	**grxD**	Glutaredoxin-4	Posttranslational modification, protein turnover, chaperones	**O**
AlbB_00135	**bcr**	Bicyclomycin resistance protein	Amino acid transport and metabolism	**E**
AlbB_00136	**ubiE**	Ubiquinone/menaquinone biosynthesis C-methyltransferase UbiE	Ubiquinone and other terpenoid-quinone biosynthesis	**H**
AlbB_00137	**-**	Putative multidrug export ATP-binding/permease protein	Bacterial secretion system	**U**
AlbB_00141	**ccmA**	Cytochrome c biogenesis ATP-binding export protein CcmA	ABC transporters	**V**
AlbB_00142	**-**	CDS	-	**S**
AlbB_00143	**tadA**	tRNA-specific adenosine deaminase	Nucleotide transport and metabolism	**F**
AlbB_00146	**pyrG**	CTP synthase	Pyrimidine metabolism	**F**
AlbB_00147	**secG**	preprotein translocase subunit SecG	Bacterial secretion system	**U**
AlbB_00153	**yajC**	preprotein translocase subunit YajC	Bacterial secretion system	**U**
AlbB_00155	**rpsB**	30S ribosomal protein S2	Ribosome	**J**
AlbB_00156	**tsf**	Elongation factor Ts	Translation, ribosomal structure and biogenesis	**J**
AlbB_00157	**pyrH**	Uridylate kinase	Pyrimidine metabolism	**F**
AlbB_00158	**frr**	Ribosome-recycling factor	Translation, ribosomal structure and biogenesis	**J**

AlbB_00159	**pstS**	Phosphate-binding protein PstS precursor	ABC transporters	**P**
AlbB_00162	**dcd**	Deoxycytidine triphosphate deaminase	Pyrimidine metabolism	**F**
AlbB_00165	-	Calcineurin-like phosphoesterase	General function prediction only	**R**
AlbB_00167	**virB8**	Type IV secretion system protein virB8	Bacterial secretion system	**U**
AlbB_00168	**ptlF**	Type IV secretion system protein PtlF precursor	Bacterial secretion system	**U**
AlbB_00169	**virB10**	Type IV secretion system protein virB10	Bacterial secretion system	**U**
AlbB_00170	**virB11**	Type IV secretion system protein VirB11	Bacterial secretion system	**U**
AlbB_00171	**traG**	Conjugal transfer protein TraG	Replication, recombination and repair	**L**
AlbB_00175	**proP**	Proline/betaine transporter	Transporter/osmoregulator	**GEP**
AlbB_00181	**ftsY**	Signal recognition particle receptor FtsY	Bacterial secretion system	**U**
AlbB_00182	**nifU**	NifU-like protein	Energy production and conversion	**C**
AlbB_00184	-	CDS	-	**S**
AlbB_00185	**hscA**	chaperone protein HscA	Chaperone	**O**
AlbB_00186	**lepB**	Signal peptidase I	Protein export	**U**
AlbB_00188	**bfmBAB**	2-oxoisovalerate dehydrogenase subunit beta	Valine, leucine and isoleucine degradation	**E**
AlbB_00205	**lipA**	Lipoyl synthase	Lipoate biosynthesis	**H**
AlbB_00206	**rpmB**	50S ribosomal protein L28	Ribosome	**J**
AlbB_00207	**-**	CDS	-	**S**
AlbB_00208	**-**	CDS	-	**S**
AlbB_00211	**tktA**	Transketolase 1	Biosynthesis of amino acids	**G**
AlbB_00212	**prs**	Ribose-phosphate pyrophosphokinase	Pentose phosphate pathway	**F**
AlbB_00213	**gatA**	Glutamyl-tRNA(Gln) amidotransferase subunit A	Glutamate biosynthesis	**E**
AlbB_00215	**rnj1**	Ribonuclease J 1	Replication, recombination and repair	**L**
AlbB_00216	**dnaJ**	chaperone protein DnaJ	Posttranslational modification, protein turnover, chaperones	**O**
AlbB_00222	**fumC**	Fumarate hydratase class II	Carbon metabolism	**C**
AlbB_00225	**gph**	Phosphoglycolate phosphatase	Glyoxylate and dicarboxylate metabolism	**R**
AlbB_00226	**-**	RDD family protein	-	**R**
AlbB_00228	**-**	SURF1 family protein	General function prediction only	**R**
AlbB_00229	**murC**	UDP-N-acetylmuramate--L-alanine ligase	Peptidoglycan biosynthesis	**M**
AlbB_00231	**eno**	Enolase	Biosynthesis of amino acids	**G**
AlbB_00232	**obg**	GTPase Obg	General function prediction only	**R**
AlbB_00234	**ftsK**	DNA translocase FtsK	Cell cycle control, cell division, chromosome partitioning	**D**
AlbB_00235	**-**	CDS	-	**S**
AlbB_00236	**ispG**	4-hydroxy-3-methylbut-2-en-1-yl diphosphate synthase	Terpenoid backbone biosynthesis	**I**
AlbB_00237	**-**	CDS	-	**S**
AlbB_00245	-	Membrane transport protein	General function prediction only	**R**
AlbB_00250	**argB**	Acetylglutamate kinase	Arginine biosynthesis	**E**
AlbB_00257	**alsT**	Amino-acid carrier protein AlsT	Amino acid transport	**E**
AlbB_00268	**aspS**	Aspartate--tRNA ligase	Aminoacyl-tRNA biosynthesis	**J**
AlbB_00272	**tpiA**	Triosephosphate isomerase	Biosynthesis of amino acids	**G**
AlbB_00276	**birA**	Bifunctional ligase/repressor BirA	Biotin metabolism	**H**
AlbB_00279	**nuoL**	NADH-quinone oxidoreductase subunit L	Oxidative phosphorylation	**CP**
AlbB_00280	**nuoK**	NADH-quinone oxidoreductase subunit K	Oxidative phosphorylation	**C**
AlbB_00284	**rsbQ**	Sigma factor SigB regulation protein RsbQ	Transcription	**K**
AlbB_00297	**der**	GTPase Der	Translation, ribosomal structure and biogenesis	**J**
AlbB_00305	**xerD**	Tyrosine recombinase XerD	Recombination	**L**
AlbB_00306	**-**	CDS	-	**S**
AlbB_00318	**elbB**	Enhancing lycopene biosynthesis protein 2	Secondary metabolites biosynthesis, transport and catabolism	**Q**
AlbB_00325	**prsD**	Type I secretion system ATP-binding protein PrsD	Intracellular trafficking, secretion, and vesicular transport	**U**
AlbB_00341	**pdhC**	Dihydrolipoyllysine-residue acetyltransferase component of pyruvate dehydrogenase complex	Citrate cycle (TCA cycle)	**C**
AlbB_00350	**rnd**	ribonuclease D	Translation, ribosomal structure and biogenesis	**J**
AlbB_00354	**ftsH**	ATP-dependent zinc metalloprotease FtsH	Protein modification	**O**
AlbB_00357	**tilS**	tRNA(Ile)-lysidine synthase	Purine metabolism	**D**
AlbB_00358	**pal**	Outer membrane protein P6 precursor	Membrane assembly	**M**
AlbB_00359	**cpsA1**	Thermostable carboxypeptidase 1	General function prediction only	**R**
AlbB_00360	**mreB**	Rod shape-determining protein MreB	Cell cycle control, cell division, chromosome partitioning	**D**
AlbB_00361	**mnmA**	tRNA-specific 2-thiouridylase MnmA	Sulfur relay system	**H**
AlbB_00365	**-**	peptidylprolyl isomerase	General function prediction only	**R**
AlbB_00366	-	putative bifunctional glutamate synthase subunit beta/2-polyprenylphenol hydroxylase	Amino acid transport and metabolism	**E**
AlbB_00367	**-**	putative 5-formyltetrahydrofolate cyclo-ligase	One carbon pool by folate	**R**
AlbB_00369	-	putative disulfide oxidoreductase	General function prediction only	**R**
AlbB_00370	**lpd**	Dihydrolipoyl dehydrogenase	Carbon metabolism	**C**
AlbB_00372	**murG**	UDP-N-acetylglucosamine--N-acetylmuramyl-(pentapeptide) pyrophosphoryl-undecaprenol N-acetylglucosamine transferase	Peptidoglycan biosynthesis	**M**
AlbB_00374	**bcp**	Putative peroxiredoxin bcp	Phenylpropanoid biosynthesis	**O**
AlbB_00377	**kefC**	Glutathione-regulated potassium-efflux system protein KefC	Posttranslational modification, protein turnover, chaperones	**O**
AlbB_00378	**resA**	Thiol-disulfide oxidoreductase ResA	Replication, recombination and repair	**L**
AlbB_00379	**tmk**	Thymidylate kinase	Pyrimidine metabolism	**F**
AlbB_00392	**dnaX**	DNA polymerase III subunit tau	Purine metabolism	**L**
AlbB_00397	**murE**	UDP-N-acetylmuramoyl-L-alanyl-D-glutamate--2,6-diaminopimelate ligase	Lysine biosynthesis	**M**
AlbB_00398	**metC**	Cystathionine beta-lyase MetC	Methionine biosynthesis	**E**
AlbB_00399	**bolA**	transcriptional regulator BolA	Transcription	**T**
AlbB_00400	**dnaK**	Chaperone protein DnaK	RNA degradation	**O**
AlbB_00401	**rne**	Ribonuclease E	RNA degradation	**J**
AlbB_00405	**znuC**	Zinc import ATP-binding protein ZnuC	ABC transporters	**P**

AlbB_00406	**ubiB**	putative protein kinase UbiB	Glycosylphosphatidylinositol(GPI)-anchor biosynthesis	**HC**
AlbB_00408	**carB**	Carbamoyl-phosphate synthase large chain	Arginine biosynthesis	**EF**
AlbB_00410	**-**	CDS	-	**S**
AlbB_00413	**purF**	Amidophosphoribosyltransferase precursor	Alanine, aspartate and glutamate metabolism	**F**
AlbB_00415	**coq7**	2-nonaprenyl-3-methyl-6-methoxy-1,4-benzoquinol hydroxylase	Aminobenzoate degradation	
AlbB_00416	**-**	Uracil DNA glycosylase superfamily protein	General function prediction only	**R**
AlbB_00417	**def**	Peptide deformylase	Protein modification	**J**
AlbB_00418	**ftsA**	Cell division protein FtsA	Cell cycle control, cell division, chromosome partitioning	**D**
AlbB_00419	**map**	Methionine aminopeptidase	Translation, ribosomal structure and biogenesis	**J**
AlbB_00421	**rsmA**	Ribosomal RNA small subunit methyltransferase A	Translation, ribosomal structure and biogenesis	**J**
AlbB_00422	**-**	CDS	-	**S**
AlbB_00423	**rplY**	50S ribosomal protein L25	Ribosome	**J**
AlbB_00425	**truA**	tRNA pseudouridine synthase A	Translation, ribosomal structure and biogenesis	**J**
AlbB_00426	**dnaN**	DNA polymerase III subunit beta	Purine metabolism	**L**
AlbB_00427	**-**	CDS	-	**S**
AlbB_00428	**rpoH**	RNA polymerase sigma factor RpoH	Transcription	**K**
AlbB_00431	**dnaX**	DNA polymerase III subunit tau	Purine metabolism	**L**
AlbB_00434	**-**	Putative O-methyltransferase/MSMEI 4947	General function prediction only	**R**
AlbB_00441	**murD**	UDP-N-acetylmuramoylalanine--D-glutamate ligase	Peptidoglycan biosynthesis	**M**
AlbB_00445	**nth**	Endonuclease III	Base excision repair	**L**
AlbB_00446	**purH**	Bifunctional purine biosynthesis protein PurH	Purine metabolism	**F**
AlbB_00447	**fmt**	Methionyl-tRNA formyltransferase	Aminoacyl-tRNA biosynthesis	**J**
AlbB_00448	**rplT**	50S ribosomal protein L20	Ribosome	**J**
AlbB_00449	**rpmI**	50S ribosomal protein L35	Ribosome	**J**
AlbB_00450	**-**	PAS domain protein	General function prediction only	**R**
AlbB_00453	**alaS**	Alanine--tRNA ligase	Aminoacyl-tRNA biosynthesis	**J**
AlbB_00456	**gpmI**	2,3-bisphosphoglycerate-independent phosphoglycerate mutase	Glycolysis / Gluconeogenesis	**G**
AlbB_00458	**fabD**	Malonyl CoA-acyl carrier protein transacylase	Fatty acid biosynthesis	**I**
AlbB_00459	**rpmE**	50S ribosomal protein L31	Ribosome	**J**
AlbB_00460	**ppnK**	putative inorganic polyphosphate/ATP-NAD kinase	Nicotinate and nicotinamide metabolism	**R**
AlbB_00463	**pyrB**	Aspartate carbamoyltransferase	Alanine, aspartate and glutamate metabolism	**F**
AlbB_00464	**-**	Ankyrin repeats (3 copies)	Ankyrin	**R**
AlbB_00465	**pipB2**	Secreted effector protein pipB2	Secondary metabolites biosynthesis, transport and catabolism	**Q**
AlbB_00466	**gmk**	Guanylate kinase	Purine metabolism	**F**
AlbB_00468	**sdhA**	Succinate dehydrogenase flavoprotein subunit	Butanoate metabolism	**C**
AlbB_00470	**dapD**	2,3,4,5-tetrahydropyridine-2,6-dicarboxylate N-succinyltransferase	Lysine biosynthesis	**E**
AlbB_00471	**-**	CDS	-	**S**
AlbB_00472	**-**	CDS	-	**S**
AlbB_00478	**gapB**	Glyceraldehyde-3-phosphate dehydrogenase 2	Glycolysis / Gluconeogenesis	**E**
AlbB_00486	**cca**	CCA-adding enzyme	RNA transport	**J**
AlbB_00487	**typA**	GTP-binding protein TypA/BipA	Signal transduction mechanisms	**T**
AlbB_00488	**gltX1**	Glutamate--tRNA ligase 1	Translation, ribosomal structure and biogenesis	**J**
AlbB_00489	**holA**	DNA polymerase III subunit delta	Purine metabolism	**L**
AlbB_00491	**znuB**	High-affinity zinc uptake system membrane protein ZnuB	ABC transporters	**P**
AlbB_00493	**ispE**	4-diphosphocytidyl-2-C-methyl-D-erythritol kinase	Terpenoid backbone biosynthesis	**I**
AlbB_00497	**thcC**	Rhodocoxin	Inorganic ion transport and metabolism	**P**
AlbB_00501	**dnaG**	DNA primase	DNA replication	**L**
AlbB_00502	**ubiG**	Ubiquinone biosynthesis O-methyltransferase	Ubiquinone and other terpenoid-quinone biosynthesis	**H**
AlbB_00504	**tsaB**	tRNA threonylcarbamoyladenosine biosynthesis protein TsaB	Translation, ribosomal structure and biogenesis	**J**
AlbB_00507	**ccmB**	CcmB protein	ABC transporters	**O**
AlbB_00508	**dksA**	RNA polymerase-binding transcription factor DksA	Signal transduction mechanisms	**T**
AlbB_00512	**-**	RlpA-like protein precursor	General function prediction only	**R**
AlbB_00514	**ubiI**	2-octaprenylphenol hydroxylase	Coenzyme transport and metabolism	**H**
AlbB_00515	**-**	CDS	-	**S**
AlbB_00519	**cdsA**	Phosphatidate cytidylyltransferase	Glycerophospholipid metabolism	**I**
AlbB_00520	**ispU**	Ditrans,polycis-undecaprenyl-diphosphate synthase ((2E,6E)-farnesyl-diphosphate specific)	Terpenoid backbone biosynthesis	**I**
AlbB_00522	**sucB**	Dihydrolipoyllysine-residue succinyltransferase component of 2-oxoglutarate dehydrogenase complex	Carbon metabolism	**C**
AlbB_00524	**fabI**	Enoyl-[acyl-carrier-protein] reductase [NADH] FabI	Fatty acid biosynthesis	**I**
AlbB_00525	**-**	CDS	-	**S**
AlbB_00527	**ychF**	Ribosome-binding ATPase YchF	Translation, ribosomal structure and biogenesis	**J**
AlbB_00530	**glpX**	Fructose-1,6-bisphosphatase class 2	Carbon metabolism	**G**
AlbB_00533	**fbpC**	Fe(3+) ions import ATP-binding protein FbpC	ABC transporters	**P**
AlbB_00534	**-**	twin arginine translocase protein A	Amino acid transport and metabolism	**E**
AlbB_00536	**exoD**	Exopolysaccharide synthesis, ExoD	General function prediction only	**R**
AlbB_00537	**cysS**	Cysteine--tRNA ligase	Aminoacyl-tRNA biosynthesis	**J**
AlbB_00539	**-**	CDS	-	**S**
AlbB_00540	**rnhA**	Ribonuclease HI	DNA replication	**L**
AlbB_00541	**ctaB**	Protoheme IX farnesyltransferase	Chlorocyclohexane and chlorobenzene degradation	**O**
AlbB_00542	**ctaD**	Cytochrome c oxidase subunit 1	Oxidative phosphorylation	**C**
AlbB_00543	**ctaC**	Cytochrome c oxidase subunit 2 precursor	Oxidative phosphorylation	**C**
AlbB_00546	**-**	Acetoin:2,6-dichlorophenolindophenol oxidoreductase	Ketone degradation	**C**

AlbB_00547	**gatB**	Aspartyl/glutamyl-tRNA(Asn/Gln) amidotransferase subunit B	Glutamate biosynthesis	**J**
AlbB_00550	**lpd3**	Dihydrolipoyl dehydrogenase 3	Energy production and conversion	**C**
AlbB_00563	**dapB**	4-hydroxy-tetrahydrodipicolinate reductase	Lysine biosynthesis	**E**
AlbB_00564	**pstB**	Phosphate import ATP-binding protein PstB	ABC transporters	**P**
AlbB_00567	**-**	CDS	-	**S**
AlbB_00570	**acpS**	Holo-[acyl-carrier-protein] synthase	Pantothenate and CoA biosynthesis	**I**
AlbB_00577	**-**	Nitronate monooxygenase	General function prediction only	**R**
AlbB_00579	**nrdB**	Ribonucleoside-diphosphate reductase subunit beta	Purine metabolism	**F**
AlbB_00580	**bamE**	outer membrane biogenesis protein BamE	Membrane assembly	**M**
AlbB_00582	**metG**	Methionine--tRNA ligase	Selenocompound metabolism	**J**
AlbB_00583	**dapE**	Succinyl-diaminopimelate desuccinylase	Lysine biosynthesis	**E**
AlbB_00601	**infC**	Translation initiation factor IF-3	Translation, ribosomal structure and biogenesis	**J**
AlbB_00602	**thrS**	Threonine--tRNA ligase	Aminoacyl-tRNA biosynthesis	**J**
AlbB_00603	**nuoI**	NADH-quinone oxidoreductase subunit I	Oxidative phosphorylation	**C**
AlbB_00606	**fpr**	Ferredoxin--NADP reductase	Energy production and conversion	**C**
AlbB_00608	**mraY**	Phospho-N-acetylmuramoyl-pentapeptide-transferase	Peptidoglycan biosynthesis	**M**
AlbB_00609	**rnhB**	Ribonuclease HII	DNA replication	**L**
AlbB_00610	**ptlF**	Type IV secretion system protein PtlF precursor	Intracellular trafficking, secretion, and vesicular transport	**U**
AlbB_00612	**-**	CDS	-	**S**
AlbB_00613	**purM**	Phosphoribosylformylglycinamidine cyclo-ligase	Purine metabolism	**F**
AlbB_00614	**purC**	Phosphoribosylaminoimidazole-succinocarboxamide synthase	Purine metabolism	**F**
AlbB_00615	**trmD**	tRNA (guanine-N(1)-)-methyltransferase	Translation, ribosomal structure and biogenesis	**J**
AlbB_00617	**pstA**	Phosphate transport system permease protein PstA	ABC transporters	**P**
AlbB_00618	**-**	phosphoribosylformylglycinamidine synthase II	Purine metabolism	**F**
AlbB_00625	**minD**	Septum site-determining protein MinD	Cell cycle control, cell division, chromosome partitioning	**D**
AlbB_00628	**-**	NADH dehydrogenase	Energy production and conversion	**C**
AlbB_00629	**mrpD**	Na(+)/H(+) antiporter subunit D	Inorganic ion transport and metabolism	**P**
AlbB_00630	**mrdB**	Rod shape-determining protein RodA	Cell cycle control, cell division, chromosome partitioning	**D**
AlbB_00636	**mutL**	DNA mismatch repair protein MutL	Mismatch repair	**L**
AlbB_00657	**petA**	Ubiquinol-cytochrome c reductase iron-sulfur subunit	Oxidative phosphorylation	
AlbB_00658	**gyrA**	DNA gyrase subunit A	Replication, recombination and repair	**L**
AlbB_00666	**tlyC**	Hemolysin C	General function prediction only	**R**
AlbB_00673	**glyA**	Serine hydroxymethyltransferase	Glycine biosynthesis	**HE**
AlbB_00674	**pgk**	Phosphoglycerate kinase	Biosynthesis of amino acids	**G**
AlbB_00677	**rpe**	Ribulose-phosphate 3-epimerase	Biosynthesis of amino acids	**G**
AlbB_00678	**pnp**	Polyribonucleotide nucleotidyltransferase	Purine metabolism	**F**
AlbB_00679	**rpsO**	30S ribosomal protein S15	Ribosome	**J**
AlbB_00680	**truB**	tRNA pseudouridine synthase B	Translation, ribosomal structure and biogenesis	**J**
AlbB_00682	**dgt**	deoxyguanosinetriphosphate triphosphohydrolase-like protein	Purine metabolism	**F**
AlbB_00683	**erpA**	Iron-sulfur cluster insertion protein ErpA	Inorganic ion transport and metabolism	**P**
AlbB_00684	**lolD**	Lipoprotein-releasing system ATP-binding protein LolD	Two-component system	**O**
AlbB_00686	**-**	Alpha/beta hydrolase family protein	-	**R**
AlbB_00689	**ybhL**	Inner membrane protein YbhL	Intracellular trafficking, secretion, and vesicular transport	**U**
AlbB_00690	**ctaG**	Cytochrome c oxidase assembly protein CtaG	Oxidative phosphorylation	**C**
AlbB_00693	**qorA**	Quinone oxidoreductase 1	Energy production and conversion	**C**
AlbB_00702	**trxB**	Thioredoxin reductase	Pyrimidine metabolism	**O**
AlbB_00703	**tsaA**	putative peroxiredoxin	Translation, ribosomal structure and biogenesis	**J**
AlbB_00705	**accD5**	putative propionyl-CoA carboxylase beta chain 5	Fatty acid biosynthesis	**I**
AlbB_00709	**tyrS**	Tyrosine--tRNA ligase	Aminoacyl-tRNA biosynthesis	**J**
AlbB_00711	**rsfS**	Ribosomal silencing factor RsfS	Translation, ribosomal structure and biogenesis	**J**
AlbB_00715	**rppH**	RNA pyrophosphohydrolase	Carbapenem biosynthesis	**J**
AlbB_00717	**mdh**	Malate dehydrogenase	Carbon metabolism	**C**
AlbB_00718	**nuoC1**	NADH-quinone oxidoreductase subunit C 1	Oxidative phosphorylation	**C**
AlbB_00719	**nuoB**	NADH-quinone oxidoreductase subunit B	Oxidative phosphorylation	**C**
AlbB_00720	**ndhC**	NAD(P)H-quinone oxidoreductase subunit 3	Carbohydrate transport and metabolism	**G**
AlbB_00721	**-**	HIT-like protein	Base excision repair	**R**
AlbB_00723	**-**	CDS	-	**S**
AlbB_00724	**murF**	UDP-N-acetylmuramoyl-tripeptide--D-alanyl-D-alanine ligase	Lysine biosynthesis	**M**
AlbB_00727	**fabF**	3-oxoacyl-[acyl-carrier-protein] synthase 2	Fatty acid biosynthesis	**I**
AlbB_00728	**plsX**	Phosphate acyltransferase	Glycerolipid metabolism	**I**
AlbB_00732	**ffh**	Signal recognition particle protein	Bacterial secretion system	**U**
AlbB_00733	**hyfB**	Hydrogenase-4 component B	Mulitple COGs	**CP**
AlbB_00738	**bamA**	Outer membrane protein assembly factor BamA precursor	Membrane assembly	**M**
AlbB_00739	**mmpA**	Metalloprotease MmpA	Inorganic ion transport and metabolism	**P**
AlbB_00740	**ygfY**	Flavinator of succinate dehydrogenase	Transcription	**K**
AlbB_00743	**rpsA**	30S ribosomal protein S1	Ribosome	**C**
AlbB_00747	**dapF**	Diaminopimelate epimerase	Lysine biosynthesis	**E**
AlbB_00748	**sucC**	Succinyl-CoA ligase [ADP-forming] subunit beta	C5-Branched dibasic acid metabolism	**C**
AlbB_00755	**hemF**	Coproporphyrinogen-III oxidase, aerobic	Porphyrin metabolism	**H**
AlbB_00756	**-**	Blue-light-activated protein	General function prediction only	**R**
AlbB_00762	**rimM**	Ribosome maturation factor RimM	Translation, ribosomal structure and biogenesis	**J**
AlbB_00763	**efp**	Elongation factor P	Translation, ribosomal structure and biogenesis	**J**
AlbB_00764	**suhB**	Inositol-1-monophosphatase	Streptomycin biosynthesis	**G**
AlbB_00765	**hemA**	5-aminolevulinate synthase	Heme biosynthesis	**H**
AlbB_00767	**htpG**	Chaperone protein HtpG	Chaperone	**O**
AlbB_00775	**priA**	Primosomal protein N'	Homologous recombination	**L**
AlbB_00778	**thyX**	Thymidylate synthase ThyX	Pyrimidine metabolism	**E**

AlbB_00779	**murA**	UDP-N-acetylglucosamine 1-carboxyvinyltransferase	Peptidoglycan biosynthesis	**M**
AlbB_00780	**fabF**	3-oxoacyl-[acyl-carrier-protein] synthase 2	Fatty acid biosynthesis	**I**
AlbB_00781	**acpP**	Acyl carrier protein	Pantothenate and CoA biosynthesis	**IQ**
AlbB_00783	**slyD**	FKBP-type peptidyl-prolyl cis-trans isomerase	General function prediction only	**R**
AlbB_00784	**hslU**	ATP-dependent protease ATPase subunit HslU	Posttranslational modification, protein turnover, chaperones	**O**
AlbB_00785	**hslV**	ATP-dependent protease subunit HslV	Posttranslational modification, protein turnover, chaperones	**O**
AlbB_00786	**gshA**	Glutamate-cysteine ligase	Glutathione biosynthesis	**H**
AlbB_00787	**-**	CDS	-	**S**
AlbB_00788	**hemH**	Ferrochelatase	Heme biosynthesis	**H**
AlbB_00789	**-**	CDS	-	**S**
AlbB_00790	**ndk**	Nucleoside diphosphate kinase	Purine metabolism	**F**
AlbB_00792	**corC**	Magnesium and cobalt efflux protein CorC	Ion transport	**P**
AlbB_00793	**-**	CDS	-	**S**
AlbB_00794	**ubiD**	3-octaprenyl-4-hydroxybenzoate carboxy-lyase	Ubiquinone and other terpenoid-quinone biosynthesis	**H**
AlbB_00795	**folD**	Bifunctional protein FolD protein	Carbon metabolism	**H**
AlbB_00798	**leuS**	Leucine--tRNA ligase	Aminoacyl-tRNA biosynthesis	**J**
AlbB_00799	**nlpD**	Murein hydrolase activator NlpD precursor	Cell wall/membrane/envelope biogenesis	**M**
AlbB_00809	**purD**	Phosphoribosylamine--glycine ligase	Purine metabolism	**F**
AlbB_00810	**serS**	Serine--tRNA ligase	Aminoacyl-tRNA biosynthesis	**J**
AlbB_00814	**rplL**	50S ribosomal protein L7/L12	Ribosome	**J**
AlbB_00815	**rplJ**	50S ribosomal protein L10	Ribosome	**J**
AlbB_00816	**rplA**	50S ribosomal protein L1	Ribosome	**J**
AlbB_00817	**rplK**	50S ribosomal protein L11	Ribosome	**J**
AlbB_00818	**-**	CDS	-	**S**
AlbB_00821	**rpsG**	30S ribosomal protein S7	Ribosome	**J**
AlbB_00822	**rpsL**	30S ribosomal protein S12	Ribosome	**J**
AlbB_00823	**prmC**	Release factor glutamine methyltransferase	Aminobenzoate degradation	**J**
AlbB_00825	**osmY**	Osmotically-inducible protein Y precursor	General function prediction only	**R**
AlbB_00828	**clpP**	ATP-dependent Clp protease proteolytic subunit	Misfolded protein degradation	**O**
AlbB_00829	**clpA**	ATP-dependent Clp protease ATP-binding subunit ClpA	Misfolded protein degradation	**O**
AlbB_00831	**spoVD**	Stage V sporulation protein D	General function prediction only	**R**
AlbB_00838	**ybeY**	Endoribonuclease YbeY	Translation, ribosomal structure and biogenesis	**J**
AlbB_00839	**sdhB**	Succinate dehydrogenase iron-sulfur subunit	Butanoate metabolism	**C**
AlbB_00843	**gpsA**	Glycerol-3-phosphate dehydrogenase [NAD(P)+]	Glycerophospholipid metabolism	**C**
AlbB_00846	**-**	CDS	-	**S**
AlbB_00848	**yidC**	Membrane protein insertase YidC	Bacterial secretion system	**U**
AlbB_00852	**secF**	preprotein translocase subunit SecF	Bacterial secretion system	**U**
AlbB_00853	**dnaA**	Chromosomal replication initiator protein DnaA	Two-component system	**L**
AlbB_00854	**puuA**	Gamma-glutamylputrescine synthetase PuuA	Arginine and proline metabolism	
AlbB_00860	**nuoD**	NADH-quinone oxidoreductase subunit D	Oxidative phosphorylation	**C**
AlbB_00862	**-**	Nitronate monooxygenase	General function prediction only	**R**
AlbB_00864	**-**	CDS	-	**S**
AlbB_00865	**virB4**	Type IV secretion system protein virB4	Bacterial secretion system	**U**
AlbB_00866	**-**	CDS	-	**S**
AlbB_00869	**estB**	Carboxylesterase 2	Drug metabolism - other enzymes	
AlbB_00872	**mnmG**	tRNA uridine 5-carboxymethylaminomethyl modification enzyme MnmG	Translation, ribosomal structure and biogenesis	**J**
AlbB_00878	**-**	CDS	-	**S**
AlbB_00881	**ptrA**	Protease 3 precursor	Amino acid transport and metabolism	**E**
AlbB_00884	**nqo2**	NADH-quinone oxidoreductase chain 2	Carbohydrate transport and metabolism	**G**
AlbB_00887	**rlmE**	Ribosomal RNA large subunit methyltransferase E	Translation, ribosomal structure and biogenesis	**J**
AlbB_00889	**bepC**	Outer membrane efflux protein BepC precursor	Intracellular trafficking, secretion, and vesicular transport	**U**
AlbB_00890	**rplM**	50S ribosomal protein L13	Ribosome	**J**
AlbB_00891	**rpsI**	30S ribosomal protein S9	Ribosome	**J**
AlbB_00895	**gltX**	Glutamate--tRNA ligase	Porphyrin metabolism	**M**
AlbB_00897	**accA1**	Acetyl-/propionyl-coenzyme A carboxylase alpha chain	Fatty acid biosynthesis	**I**
AlbB_00898	**ctaA**	Heme A synthase	Benzoate degradation	**O**
AlbB_00901	**atpF**	F0F1 ATP synthase subunit B	Oxidative phosphorylation	**C**
AlbB_00902	**atpF2**	ATP synthase subunit b 2	Energy production and conversion	**C**
AlbB_00903	**atpE**	ATP synthase subunit c	Oxidative phosphorylation	**C**
AlbB_00904	**atpB**	ATP synthase subunit a	Oxidative phosphorylation	**C**
AlbB_00906	**pheT**	Phenylalanine--tRNA ligase beta subunit	Aminoacyl-tRNA biosynthesis	**J**
AlbB_00908	**ileS**	Isoleucine--tRNA ligase	Aminoacyl-tRNA biosynthesis	**J**
AlbB_00911	**recJ**	Single-stranded-DNA-specific exonuclease RecJ	Base excision repair	**L**
AlbB_00912	**rsmA**	Ribosomal RNA small subunit methyltransferase A	Translation, ribosomal structure and biogenesis	**J**
AlbB_00913	**-**	CDS	-	**S**
AlbB_00914	**-**	TrbL/VirB6 plasmid conjugal transfer protein	Bacterial secretion system	**U**
AlbB_00915	**-**	TrbL/VirB6 plasmid conjugal transfer protein	Bacterial secretion system	**U**
AlbB_00916	**-**	TrbL/VirB6 plasmid conjugal transfer protein	Bacterial secretion system	**U**
AlbB_00917	**-**	TrbL/VirB6 plasmid conjugal transfer protein	Bacterial secretion system	**U**
AlbB_00919	**-**	Type IV secretory pathway, VirB3-like protein	Defense mechanisms	**V**
AlbB_00920	**lysS**	Lysine--tRNA ligase	Aminoacyl-tRNA biosynthesis	**J**
AlbB_00925	**apaG**	CO2+/MG2+ efflux protein ApaG	Inorganic ion transport and metabolism	**P**
AlbB_00926	**purK**	N5-carboxyaminoimidazole ribonucleotide synthase	Purine metabolism	**F**
AlbB_00927	**asd2**	Aspartate-semialdehyde dehydrogenase 2	Lysine biosynthesis	**E**
AlbB_00932	**surE**	5'-nucleotidase SurE	Nicotinate and nicotinamide metabolism	**E**
AlbB_00933	**hisS**	Histidine--tRNA ligase	Aminoacyl-tRNA biosynthesis	**J**
AlbB_00934	**nfuA**	Fe/S biogenesis protein NfuA	Coenzyme transport and metabolism	**H**
AlbB_00939	**petC**	Cytochrome c1 precursor	Oxidative phosphorylation	**C**

AlbB_00944	**-**	CDS	-	**S**
AlbB_00946	**-**	putative 3'-5' exonuclease related to the exonuclease domain of PolB	Replication, recombination and repair	**L**
AlbB_00947	**era**	GTPase Era	Cell cycle control, cell division, chromosome partitioning	**D**
AlbB_00950	**rnd**	ribonuclease D	Translation, ribosomal structure and biogenesis	**J**
AlbB_00951	**trxA**	Thioredoxin	Mulitple COGs	**OC**
AlbB_00954	-	CDS	-	**S**
AlbB_00956	**rbfA**	Ribosome-binding factor A	Translation, ribosomal structure and biogenesis	**J**
AlbB_00957	**ubiA**	4-hydroxybenzoate octaprenyltransferase	Ubiquinone and other terpenoid-quinone biosynthesis	**H**
AlbB_00965	**-**	CDS	-	**S**
AlbB_00968	**rplI**	50S ribosomal protein L9	Ribosome	**J**
AlbB_00969	**rpsR**	30S ribosomal protein S18	Ribosome	**J**
AlbB_00970	**rpsF**	30S ribosomal protein S6	Ribosome	**J**
AlbB_00971	**dnaE1**	DNA polymerase III subunit alpha	Base excision repair	**L**
AlbB_00974	**lipB**	Octanoyltransferase	Lipoate biosynthesis	**H**
AlbB_00977	**mutS**	DNA mismatch repair protein MutS	Mismatch repair	**L**
AlbB_00979	**ccmF**	Cytochrome c-type biogenesis protein CcmF	Oxidative phosphorylation	**O**
AlbB_00981	**dacF**	D-alanyl-D-alanine carboxypeptidase DacF precursor	Cell wall/membrane/envelope biogenesis	**M**
AlbB_00986	**secA**	preprotein translocase subunit SecA	Bacterial secretion system	**MU**
AlbB_00993	**fdxA**	Ferredoxin 1	Inorganic ion transport and metabolism	**P**
AlbB_00997	**ddl**	D-alanine--D-alanine ligase	D-Alanine metabolism	**M**
AlbB_01004	**pyrE**	Orotate phosphoribosyltransferase	Pyrimidine metabolism	**F**
AlbB_01007	**hemC**	Porphobilinogen deaminase	Heme biosynthesis	**H**
AlbB_01009	**secD**	preprotein translocase subunit SecD	Bacterial secretion system	**U**
AlbB_01010	**-**	Surface antigen	Defense	**R**
AlbB_01017	**yqfL**	Putative pyruvate, phosphate dikinase regulatory protein	General function prediction only	**R**
AlbB_01018	**ccmC**	Heme exporter protein C	ABC transporters	**O**
AlbB_01022	**guaA**	GMP synthase [glutamine-hydrolyzing]	Purine metabolism	**F**
AlbB_01023	**terC**	Integral membrane protein TerC family protein	Inorganic ion transport and metabolism	**P**
AlbB_01024	-	Malonyl-CoA decarboxylase (MCD)	General function prediction only	**R**
AlbB_01027	**lepA**	Elongation factor 4	Cell wall/membrane/envelope biogenesis	**M**
AlbB_01029	-	CDS	-	**S**
AlbB_01030	**grxC**	Glutaredoxin-3	Posttranslational modification, protein turnover, chaperones	**O**
AlbB_01032	**lspA**	Lipoprotein signal peptidase	Protein export	**MU**
AlbB_01033	**ptrA**	Protease 3 precursor	Amino acid transport and metabolism	**E**
AlbB_01034	**ymfF**	Peptidase M16 inactive domain protein	General function prediction only	**R**
AlbB_01035	**purN**	Phosphoribosylglycinamide formyltransferase	Purine metabolism	**F**
AlbB_01037	**-**	putative monovalent cation/H+ antiporter subunit B	Bacterial secretion system	**U**
AlbB_01040	**smpB**	SsrA-binding protein	Posttranslational modification, protein turnover, chaperones	**O**
AlbB_01041	**lgt**	Prolipoprotein diacylglyceryl transferase	Glycosphingolipid biosynthesis - ganglio series	**M**
AlbB_01044	**ubiD**	3-octaprenyl-4-hydroxybenzoate carboxy-lyase	Ubiquinone and other terpenoid-quinone biosynthesis	**H**
AlbB_01046	**-**	DSBA-like thioredoxin domain protein	General function prediction only	**R**
AlbB_01051	**sucA**	2-oxoglutarate dehydrogenase E1 component	Carbon metabolism	**C**
AlbB_01055	**rpsP**	30S ribosomal protein S16	Ribosome	**J**
AlbB_01057	-	CDS	-	**S**
AlbB_01079	**degP1**	putative periplasmic serine endoprotease DegP-like precursor	Posttranslational modification, protein turnover, chaperones	**O**
AlbB_01084	**purE**	N5-carboxyaminoimidazole ribonucleotide mutase	Purine metabolism	**F**
AlbB_01085	-	CDS	-	**S**
AlbB_01086	**rpoZ**	DNA-directed RNA polymerase subunit omega	Purine metabolism	**K**
AlbB_01087	-	putative monovalent cation/H+ antiporter subunit B	Bacterial secretion system	**U**
AlbB_01088	**mrpG**	Na(+)/H(+) antiporter subunit G	Inorganic ion transport and metabolism	**P**
AlbB_01089	**mrpA**	Na(+)/H(+) antiporter subunit A	Inorganic ion transport and metabolism	**P**
AlbB_01090	**mrpB**	Na(+)/H(+) antiporter subunit B	Inorganic ion transport and metabolism	**P**
AlbB_01091	**mnhC1**	Na(+)/H(+) antiporter subunit C1	Inorganic ion transport and metabolism	**P**
AlbB_01092	**rpoD**	RNA polymerase sigma factor RpoD	Transcription	**K**
AlbB_01095	**-**	CDS	-	**S**
AlbB_01097	**sdhC**	Succinate dehydrogenase cytochrome b556 subunit	Butanoate metabolism	**C**
AlbB_01102	**lolC**	Lipoprotein-releasing system transmembrane protein LolC	ABC transporters	**E**
AlbB_01105	**atpG**	ATP synthase gamma chain	Oxidative phosphorylation	**C**
AlbB_01106	**pmbA**	peptidase PmbA	General function prediction only	**R**
AlbB_01107	**cspA**	Cold shock protein CspA	General function prediction only	**R**
AlbB_01111	**fbaB**	Fructose-bisphosphate aldolase class 1	Biosynthesis of amino acids	**G**
AlbB_01114	**pyrD**	Dihydroorotate dehydrogenase (quinone)	Pyrimidine metabolism	**F**
AlbB_01115	**rnc**	Ribonuclease 3	Transcription	**K**
AlbB_01116	**-**	CDS	-	**S**
AlbB_01117	**dnaQ**	DNA polymerase III subunit epsilon	Purine metabolism	**L**
AlbB_01118	-	Tim44-like domain protein	General function prediction only	**R**
AlbB_01120	**acnA**	Aconitate hydratase 1	Biosynthesis of amino acids	**C**
AlbB_01121	**putA**	Bifunctional protein PutA	Glutamate biosynthesis	**J**
AlbB_01123	**pepA**	Cytosol aminopeptidase	Arginine and proline metabolism	**E**
AlbB_01126	**nqo3**	NADH-quinone oxidoreductase chain 3	Carbohydrate transport and metabolism	**G**
AlbB_01127	**nuoH**	NADH-quinone oxidoreductase subunit H	Oxidative phosphorylation	**C**
AlbB_01128	**hemB**	Delta-aminolevulinic acid dehydratase	Heme biosynthesis	**H**
AlbB_01129	-	CDS	-	**S**
AlbB_01130	**glyQ**	Glycine--tRNA ligase alpha subunit	Aminoacyl-tRNA biosynthesis	**J**
AlbB_01133	-	CDS	-	**S**
AlbB_01134	**-**	Putative TrmH family tRNA/rRNA methyltransferase	General function prediction only	**R**

Anaa_00014	**-**	CDS	-	**S**
Anaa_00157	**-**	CDS	-	**S**
Anaa_00200	**ankX**	Phosphocholine transferase AnkX	Lipopolysaccharide biosynthesis	
Anaa_00214	**cycA**	Cytochrome c2	Oxidative phosphorylation	**C**
Anaa_00242	**-**	CDS	-	**S**
Anaa_00429	**ybaB**	Nucleoid-associated protein YbaB	Function unknown	**S**
Anaa_00435	**rplS**	50S ribosomal protein L19	Ribosome	**J**
Anaa_00830	**pepQ**	Xaa-Pro dipeptidase	Amino acid transport and metabolism	**E**
Anaa_00836	-	CDS	-	**S**
Anaa_00907	-	putative global regulator	Coenzyme transport and metabolism	**H**
Anaa_00908	**groS**	10 kDa chaperonin	Chaperone	**O**
Anaa_00909	**groL5**	60 kDa chaperonin 5	Chaperone	**O**
Anaa_00986	**addA**	ATP-dependent helicase/nuclease subunit A	Base excision repair	**L**
Anaa_01301	**mgtE**	Magnesium transporter MgtE	Inorganic ion transport and metabolism	**P**
Anaa_01302	**prfB**	Peptide chain release factor 2	Translation, ribosomal structure and biogenesis	**J**
Auuu_01094	-	Major Facilitator Superfamily protein	General function prediction only	**R**
Clee_00770	**infA**	Translation initiation factor IF-1	Translation, ribosomal structure and biogenesis	**J**
Clee_00771	**yhdE**	Maf-like protein YhdE	General function prediction only	**R**
Cocc_00220	-	CDS	-	**S**
Cocc_00642	**mtaB**	Threonylcarbamoyladenosine tRNA methylthiotransferase MtaB	Translation, ribosomal structure and biogenesis	**J**
Cocc_00653	**lon**	Lon protease	Posttranslational modification, protein turnover, chaperones	**O**
Cocc_00664	**prfA**	Peptide chain release factor 1	Translation, ribosomal structure and biogenesis	**J**
Cocc_00696	**guaB**	Inosine-5'-monophosphate dehydrogenase	Purine metabolism	**F**
Cocc_00727	**ispA**	Farnesyl diphosphate synthase	Terpenoid backbone biosynthesis	**H**
Cocc_00728	**prsE**	Type I secretion system membrane fusion protein PrsE	Intracellular trafficking, secretion, and vesicular transport	**U**
Cocc_00735	**topA**	DNA topoisomerase 1	Homologous recombination	**L**
Cocc_00781	**glyS**	Glycine--tRNA ligase beta subunit	Aminoacyl-tRNA biosynthesis	**J**
Cocc_00804	**rpoC**	DNA-directed RNA polymerase subunit beta'	Purine metabolism	**K**
Cocc_00809	**fusA**	Elongation factor G	Translation, ribosomal structure and biogenesis	**J**
Cocc_00819	**petB**	Cytochrome b	Oxidative phosphorylation	**C**
Cocc_00833	**ask**	Aspartokinase	Lysine biosynthesis	**E**
Cocc_00844	**infB**	Translation initiation factor IF-2	Translation, ribosomal structure and biogenesis	**J**
Cocc_00845	**addB**	ATP-dependent helicase/deoxyribonuclease subunit B	Basal transcription factors	**L**
Cocc_00865	**-**	CDS	-	**S**
Cocc_00907	**tig**	Trigger factor	Chaperone	**O**
Cocc_00953	**ftsZ**	Cell division protein FtsZ	Cell cycle control, cell division, chromosome partitioning	**D**
Cocc_00968	**virB4**	Type IV secretion system protein virB4	Bacterial secretion system	**U**
Cocc_00970	**atpD**	ATP synthase subunit beta	Oxidative phosphorylation	**C**
Haaa_00317	**ligA**	DNA ligase	Base excision repair	**U**
Haaa_00812	-	CDS	-	**S**
Ppee_00152	**murB**	UDP-N-acetylenolpyruvoylglucosamine reductase	Peptidoglycan biosynthesis	**M**
Ppee_00332	**ispDF**	Bifunctional enzyme IspD/IspF	Coenzyme transport and metabolism	**H**
Ppee_00334	**tqsA**	AI-2 transport protein TqsA	General function prediction only	**R**

PANGENOME Venn: 202 wPpe Only

**ID**	**Gene Symbol**	**Gene Product**	**Pathway or Process**	**COG**
Ppee_00008	-	CDS	-	**S**
Ppee_00012	-	CDS	-	**S**
Ppee_00013	-	CDS	-	**S**
Ppee_00038	**asd2**	Aspartate-semialdehyde dehydrogenase 2	Lysine biosynthesis	**E**
Ppee_00043	-	CDS	-	**S**
Ppee_00044	-	CDS	-	**S**
Ppee_00062	-	CDS	-	**S**
Ppee_00086	-	CDS	-	**S**
Ppee_00102	-	PD-(D/E)XK nuclease family transposase	Transposition	**L**
Ppee_00120	-	CDS	-	**S**
Ppee_00134	**ankX**	Phosphocholine transferase AnkX	Lipopolysaccharide biosynthesis	**I**
Ppee_00137	**-**	CDS	-	**S**
Ppee_00139	-	CDS	-	**S**
Ppee_00144	-	CDS	-	**S**
Ppee_00155	-	CDS	-	**S**
Ppee_00157	**def**	Peptide deformylase	Protein modification	**J**
Ppee_00161	-	CDS	-	**S**
Ppee_00180	**fmt**	Methionyl-tRNA formyltransferase	Aminoacyl-tRNA biosynthesis	**J**
Ppee_00181	-	CDS	-	**S**
Ppee_00184	-	CDS	-	**S**
Ppee_00185	-	CDS	-	**S**
Ppee_00192	**ankX**	Phosphocholine transferase AnkX	Lipopolysaccharide biosynthesis	**I**
Ppee_00195	-	CDS	-	**S**
Ppee_00196	-	CDS	-	**S**
Ppee_00197	-	CDS	-	**S**
Ppee_00198	-	DNA mismatch repair protein	Mismatch repair	**L**
Ppee_00201	-	Apocarotenoid-15,15'-oxygenase	Retinal biosynthesis	**H**
Ppee_00206	-	CDS	-	**S**
Ppee_00210	-	CDS	-	**S**
Ppee_00211	-	CDS	-	**S**
Ppee_00220	-	CDS	-	**S**
Ppee_00228	-	CDS	-	**S**
Ppee_00229	-	Ankyrin repeat protein	Ankyrin	**R**

Ppee_00230	**-**	Ankyrin repeat protein	Ankyrin	**R**
Ppee_00231	**-**	Ankyrin repeat protein	Ankyrin	**R**
Ppee_00232	**-**	CDS	-	**S**
Ppee_00237	**-**	CDS	-	**S**
Ppee_00251	**-**	CDS	-	**S**
Ppee_00252	**-**	Ankyrin repeats (3 copies)	Ankyrin	**R**
Ppee_00253	**-**	CDS	-	**S**
Ppee_00254	**-**	CDS	-	**S**
Ppee_00255	**-**	CDS	-	**S**
Ppee_00263	**-**	CDS	-	**S**
Ppee_00273	**nfo**	Endonuclease 4	Base excision repair	**L**
Ppee_00277	**-**	CDS	-	**S**
Ppee_00280	**-**	Saccharopine dehydrogenase	Oxidoreductase	**C**
Ppee_00281	**-**	CDS	-	**S**
Ppee_00282	**-**	CDS	-	**S**
Ppee_00283	**-**	CDS	-	**S**
Ppee_00284	**-**	CDS	-	**S**
Ppee_00285	**-**	CDS	-	**S**
Ppee_00302	**-**	CDS	-	**S**
Ppee_00303	**-**	CDS	-	**S**
Ppee_00304	**-**	RDD family protein	-	**R**
Ppee_00309	**-**	CDS	-	**S**
Ppee_00315	**-**	CDS	-	**S**
Ppee_00316	**-**	CDS	-	**S**
Ppee_00317	**-**	Anaphase-promoting complex, cyclosome, subunit 3	Protein-complex assembly	**R**
Ppee_00318	**-**	CDS	-	**S**
Ppee_00320	**-**	CDS	-	**S**
Ppee_00335	**-**	CDS	-	**S**
Ppee_00336	**-**	CDS	-	**S**
Ppee_00337	**rmuC**	DNA recombination protein RmuC	General function prediction only	**R**
Ppee_00338	**-**	RmuC family protein	General function prediction only	**R**
Ppee_00339	**-**	CDS	-	**S**
Ppee_00340	**rluC**	Ribosomal large subunit pseudouridine synthase C	Translation, ribosomal structure and biogenesis	**J**
Ppee_00341	**rluC**	Ribosomal large subunit pseudouridine synthase C	Translation, ribosomal structure and biogenesis	**J**
Ppee_00342	**hda**	DnaA regulatory inactivator Hda	Replication	**O**
Ppee_00343	**sucB**	Dihydrolipoyllysine-residue succinyltransferase component of 2-oxoglutarate dehydrogenase complex	Carbon metabolism	**C**
Ppee_00344	**-**	CDS	-	**S**
Ppee_00345	**-**	CDS	-	**S**
Ppee_00346	**hemC**	Porphobilinogen deaminase	Heme biosynthesis	**H**
Ppee_00347	**-**	CDS	-	**S**
Ppee_00348	**-**	CDS	-	**S**
Ppee_00350	**-**	CDS	-	**S**
Ppee_00351	**-**	NUDIX domain protein	Nucleoside modification	**L**
Ppee_00352	**cysS**	cysteinyl-tRNA synthetase	Aminoacyl-tRNA biosynthesis	**J**
Ppee_00354	**-**	CDS	-	**S**
Ppee_00355	**-**	CDS	-	**S**
Ppee_00356	**-**	CDS	-	**S**
Ppee_00357	**-**	CDS	-	**S**
Ppee_00368	**folD**	Bifunctional protein FolD protein	Carbon metabolism	**H**
Ppee_00374	**hopD**	Squalene/phytoene synthase	Bacterial secretion system	**O**
Ppee_00378	**-**	CDS	-	**S**
Ppee_00395	**-**	CDS	-	**S**
Ppee_00396	**-**	CDS	-	**S**
Ppee_00399	**-**	CDS	-	**S**
Ppee_00400	**-**	CDS	-	**S**
Ppee_00403	**ruvA**	Holliday junction ATP-dependent DNA helicase RuvA	Homologous recombination	**L**
Ppee_00415	**-**	CDS	-	**S**
Ppee_00421	**-**	CDS	-	**S**
Ppee_00436	**tuf**	elongation factor Tu	Translation	**J**
Ppee_00445	**-**	CDS	-	**S**
Ppee_00446	**-**	CDS	-	**S**
Ppee_00449	**-**	CDS	-	**S**
Ppee_00450	**-**	CDS	-	**S**
Ppee_00457	**-**	PD-(D/E)XK nuclease family transposase	Transposition	**L**
Ppee_00458	**-**	Ankyrin repeats (3 copies)	Ankyrin	**R**
Ppee_00461	**-**	CDS	-	**S**
Ppee_00465	**-**	CDS	-	**S**
Ppee_00474	**-**	CDS	-	**S**
Ppee_00481	**-**	CDS	-	**S**
Ppee_00492	**clpP**	ATP-dependent Clp protease proteolytic subunit	Misfolded protein degradation	**O**
Ppee_00493	**clpP**	ATP-dependent Clp protease proteolytic subunit	Misfolded protein degradation	**O**
Ppee_00499	**-**	CDS	-	**S**
Ppee_00504	**-**	CDS	-	**S**
Ppee_00514	**-**	CDS	-	**S**
Ppee_00539	**-**	CDS	-	**S**
Ppee_00546	**-**	CDS	-	**S**
Ppee_00548	**-**	Putative pterin-4-alpha-carbinolamine dehydratase	General function prediction only	**R**
Ppee_00553	**-**	CDS	-	**S**
Ppee_00564	**-**	CDS	-	**S**
Ppee_00578	**-**	CDS	-	**S**
Ppee_00579	**-**	CDS	-	**S**
Ppee_00580	**-**	CDS	-	**S**
Ppee_00584	**cycM**	Cytochrome c-552	Oxidative phosphorylation	**C**
Ppee_00588	**-**	CDS	-	**S**
Ppee_00591	**-**	CDS	-	**S**

Ppee_00612	**-**	CDS	-	**S**
Ppee_00614	**-**	CDS	-	**S**
Ppee_00615	**-**	CDS	-	**S**
Ppee_00616	**glyA**	Serine hydroxymethyltransferase	Glycine biosynthesis	**HE**
Ppee_00617	**-**	CDS	-	**S**
Ppee_00619	**-**	CDS	-	**S**
Ppee_00626	**-**	CDS	-	**S**
Ppee_00628	**-**	CDS	-	**S**
Ppee_00629	**-**	CDS	-	**S**
Ppee_00645	**-**	CDS	-	**S**
Ppee_00646	**-**	CDS	-	**S**
Ppee_00649	**yjdL**	putative dipeptide and tripeptide permease YjdL	Chaperone	**O**
Ppee_00660	**dnaG**	DNA primase	DNA replication	**L**
Ppee_00661	**-**	CDS	-	**S**
Ppee_00671	**-**	CDS	-	**S**
Ppee_00674	**glnA**	Glutamine synthetase	Arginine biosynthesis	**E**
Ppee_00686	**-**	CDS	-	**S**
Ppee_00687	**mreC**	Cell shape-determining protein MreC	Cell shape	**M**
Ppee_00699	**-**	CDS	-	**S**
Ppee_00705	**-**	CDS	-	**S**
Ppee_00707	**-**	CDS	-	**S**
Ppee_00708	**-**	CDS	-	**S**
Ppee_00709	**-**	CDS	-	**S**
Ppee_00710	**-**	CDS	-	**S**
Ppee_00715	**-**	CDS	-	**S**
Ppee_00719	**-**	Methylmalonyl-CoA carboxyltransferase 1.3S subunit	Propanoate metabolism	**C**
Ppee_00721	**-**	CDS	-	**S**
Ppee_00722	**-**	CDS	-	**S**
Ppee_00723	**-**	CDS	-	**S**
Ppee_00734	**fabF**	3-oxoacyl-[acyl-carrier-protein] synthase 2	Fatty acid biosynthesis	**I**
Ppee_00738	**-**	CDS	-	**S**
Ppee_00739	**-**	CDS	-	**S**
Ppee_00747	**valS**	Valine--tRNA ligase	Aminoacyl-tRNA biosynthesis	**J**
Ppee_00766	**-**	CDS	-	**S**
Ppee_00772	**-**	CDS	-	**S**
Ppee_00773	**-**	CDS	-	**S**
Ppee_00782	**-**	CDS	-	**S**
Ppee_00795	**-**	CDS	-	**S**
Ppee_00809	**-**	CDS	-	**S**
Ppee_00816	**-**	CDS	-	**S**
Ppee_00820	**tlcA**	ADP,ATP carrier protein 1	Energy production and conversion	**C**
Ppee_00821	**-**	CDS	-	**S**
Ppee_00824	**-**	periplasmic protein	-	**U**
Ppee_00825	**-**	CDS	-	**S**
Ppee_00826	**-**	CDS	-	**S**
Ppee_00827	**ybhL**	Inner membrane protein YbhL	Intracellular trafficking, secretion, and vesicular transport	**U**
Ppee_00828	**-**	CDS	-	**S**
Ppee_00835	**-**	CDS	-	**S**
Ppee_00840	**-**	CDS	-	**S**
Ppee_00850	**-**	Ankyrin repeats (3 copies)	Ankyrin	**R**
Ppee_00856	**-**	CDS	-	**S**
Ppee_00861	**-**	CDS	-	**S**
Ppee_00880	**-**	CDS	-	**S**
Ppee_00881	**gmhB**	D,D-heptose 1,7-bisphosphate phosphatase	ADP-L-glycero-β-D-manno-heptose biosynthesis	**M**
Ppee_00882	**-**	CDS	-	**S**
Ppee_00883	**-**	CDS	-	**S**
Ppee_00889	**-**	CDS	-	**S**
Ppee_00894	**-**	CDS	-	**S**
Ppee_00896	**-**	CDS	-	**S**
Ppee_00897	**-**	CDS	-	**S**
Ppee_00907	**-**	CDS	-	**S**
Ppee_00908	**-**	CDS	-	**S**
Ppee_00909	**rimL**	ribosomal-protein-L7/L12-serine acetyltransferase	Translation, ribosomal structure and biogenesis	**J**
Ppee_00917	**-**	CDS	-	**S**
Ppee_00922	**-**	CDS	-	**S**
Ppee_00941	**-**	CDS	-	**S**
Ppee_00951	**-**	Ankyrin repeats (3 copies)	Ankyrin	**R**
Ppee_00954	**-**	CDS	-	**S**
Ppee_00955	**-**	CDS	-	**S**
Ppee_00958	**sdhA**	Succinate dehydrogenase flavoprotein subunit	Butanoate metabolism	**C**
Ppee_00959	**-**	CDS	-	**S**
Ppee_00960	**-**	Ankyrin repeat protein	Ankyrin	**R**
Ppee_00961	**-**	CDS	-	**S**
Ppee_00962	**-**	Ankyrin repeats (3 copies)	Ankyrin	**R**
Ppee_00963	**-**	Ankyrin repeats (3 copies)	Ankyrin	**R**
Ppee_00969	**-**	CDS	-	**S**
Ppee_00975	**-**	CDS	-	**S**
Ppee_00984	**-**	CDS	-	**S**
Ppee_00991	**-**	CDS	-	**S**
Ppee_00992	**-**	CDS	-	**S**
Ppee_00993	**-**	CDS	-	**S**
Ppee_00994	**-**	CDS	-	**S**
Ppee_00995	**-**	CDS	-	**S**
Ppee_00998	**-**	CDS	-	**S**

PANGENOME Venn: 29 A+B+wPpe

ID	Gene Symbol	Gene Product	Pathway or Process	COG
AlbB_00269	**acm**	Lysozyme M1 precursor	Two-component system	**M**
AlbB_00289	**ankX**	Phosphocholine transferase AnkX	Lipopolysaccharide biosynthesis	**I**
AlbB_00309	**-**	Alpha/beta hydrolase family protein	-	**R**
AlbB_00368	**-**	CDS	-	**S**
AlbB_00592	**-**	CDS	-	**S**
AlbB_00744	**trmB**	tRNA (guanine-N(7)-)-methyltransferase	Translation, ribosomal structure and biogenesis	**J**
AlbB_00894	**hspA**	Spore protein SP21	General function prediction only	**R**
AlbB_01045	**-**	CDS	-	**S**
Anaa_00521	**-**	Transposase	Transposition	**L**
Anaa_00639	**-**	CDS	-	**S**
Anaa_00640	**-**	anaerobic benzoate catabolism transcriptional regulator	DNA binding	**K**
Anaa_01183	**-**	CDS	-	**S**
Anaa_01453	**dtpA**	Dipeptide and tripeptide permease A	Amino acid transport and metabolism	**E**
Auuu_00284	**repA**	Regulatory protein RepA	Replication, recombination and repair (L)	**L**
Auuu_00515	**-**	PQ loop repeat protein	General function prediction only	**R**
Auuu_00632	**ankX**	Phosphocholine transferase AnkX	Lipopolysaccharide biosynthesis	**I**
Cocc_01424	**clpB**	Chaperone protein ClpB	Chaperone	**O**
Nooo_00157	**-**	CDS	-	**S**
Pipp_00476	**-**	CDS	-	**S**
Ppee_00015	**-**	Ankyrin repeats (3 copies)	Ankyrin	**R**
Ppee_00036	**-**	CDS	-	**S**
Ppee_00238	**-**	CDS	-	**S**
Ppee_00377	**-**	CDS	-	**S**
Ppee_00420	**msp4**	Major surface antigen 4 precursor	Defense	**V**
Ppee_00540	**bamA**	Outer membrane protein assembly factor BamA precursor	Membrane assembly	**M**
Ppee_00572	**-**	Ankyrin repeat protein	Ankyrin	**R**
Ppee_00630	**-**	CDS	-	**S**
Ppee_00746	**-**	CDS	-	**S**
Riii_00570	**-**	CDS	-	**S**

**PANGENOME Venn: 3 C+D+F+wPpe**	**-**			
**ID**	**Gene Symbol**	**Gene Product**	**Pathway or Process**	**COG**
Bmmm_00063	**-**	CDS	-	**S**
Bmmm_00651	**-**	CDS	-	**S**
Clee_00674	**-**	CDS	-	**S**
	**-**			

PANGENOME Venn: 229 A+B+C+D+F

ID	Gene Symbol	Gene Product	Pathway or Process	COG
AlbB_00003	**-**	CDS	-	**S**
AlbB_00011	**clpB**	Chaperone protein ClpB	Chaperone	**O**
AlbB_00015	**zitB**	Zinc transporter ZitB	Inorganic ion transport and metabolism	**P**
AlbB_00016	**-**	CDS	-	**S**
AlbB_00019	**alsT**	Amino-acid carrier protein AlsT	Amino acid transport	**E**
AlbB_00022	**-**	Ankyrin repeats (3 copies)	Ankyrin	**R**
AlbB_00023	**-**	CDS	-	**S**
AlbB_00031	**-**	CDS	-	**S**
AlbB_00033	**smc**	Chromosome partition protein Smc	Cell cycle control, cell division, chromosome partitioning	**D**
AlbB_00036	**ribE**	Riboflavin synthase	Riboflavin biosynthesis	**H**
AlbB_00037	**-**	CDS	-	**S**
AlbB_00043	**-**	CDS	-	**S**
AlbB_00048	**-**	CDS	-	**S**
AlbB_00057	**tenA**	Thiaminase-2	Thiamine biosynthesis/salvage	**H**
AlbB_00058	**tenA**	Thiaminase-2	Thiamine biosynthesis/salvage	**H**
AlbB_00063	**smc**	Chromosome partition protein Smc	Cell cycle control, cell division, chromosome partitioning	**D**
AlbB_00095	**-**	CDS	-	**S**
AlbB_00121	**-**	CDS	-	**S**
AlbB_00123	**mepM**	Murein DD-endopeptidase MepM	Cell wall assembly	**O**
AlbB_00129	**-**	BolA-like protein	Transcription	**R**
AlbB_00134	**-**	CDS	-	**S**
AlbB_00148	**lpxD**	UDP-3-O-[3-hydroxymyristoyl] glucosamine N-acyltransferase	Lipopolysaccharide biosynthesis	**M**
AlbB_00149	**-**	CDS	-	**S**
AlbB_00150	**-**	CDS	-	**S**
AlbB_00161	**trkG**	Trk system potassium uptake protein TrkG	Inorganic ion transport and metabolism	**P**
AlbB_00163	**-**	CDS	-	**S**
AlbB_00183	**iscA**	Iron-binding protein IscA	Iron-cluster assembly	**H**
AlbB_00187	**-**	TrbC/VIRB2 family protein	General function prediction only	**R**
AlbB_00189	**-**	Ankyrin repeat protein	Ankyrin	**R**
AlbB_00190	**uvrA**	UvrABC system protein A	Nucleotide excision repair	**L**
AlbB_00194	**-**	CDS	-	**S**
AlbB_00195	**-**	CDS	-	**S**
AlbB_00198	**-**	CDS	-	**S**
AlbB_00199	**-**	CDS	-	**S**
AlbB_00201	**-**	CDS	-	**S**
AlbB_00203	**-**	Leucine Rich repeats (2 copies)	General function prediction only	**R**
AlbB_00224	**-**	CDS	-	**S**
AlbB_00230	**-**	CDS	-	**S**
AlbB_00233	**-**	YGGT family protein	General function prediction only	**R**
AlbB_00251	**-**	CDS	-	**S**
AlbB_00256	**-**	CDS	-	**S**

AlbB_00267	**-**	CDS	-	**S**
AlbB_00282	**-**	CDS	-	**S**
AlbB_00296	**ampD**	N-acetyl-anhydromuranmyl-L-alanine amidase	Defense mechanisms (V)	**V**
AlbB_00298	**-**	CDS	-	**S**
AlbB_00299	**ribD**	Riboflavin biosynthesis protein RibD	Riboflavin biosynthesis	**H**
AlbB_00311	**-**	CDS	-	**S**
AlbB_00327	**-**	CDS	-	**S**
AlbB_00335	**-**	CDS	-	**S**
AlbB_00338	**-**	CDS	-	**S**
AlbB_00344	**-**	Thiamin pyrophosphokinase, catalytic domain	Coenzyme transport and metabolism	**H**
AlbB_00347	**-**	CDS	-	**S**
AlbB_00348	**-**	Phage portal protein	Replication, recombination and repair	**L**
AlbB_00352	**-**	CDS	-	**S**
AlbB_00371	**-**	CDS	-	**S**
AlbB_00373	**gshB**	Glutathione synthetase	Glutathione biosynthesis	**H**
AlbB_00388	**dnaB**	Replicative DNA helicase	DNA replication	**L**
AlbB_00391	**-**	CDS	-	**S**
AlbB_00407	**-**	CDS	-	**S**
AlbB_00414	**-**	CDS	-	**S**
AlbB_00420	**proP**	Proline/betaine transporter	Transporter/osmoregulator	**GEP**
AlbB_00429	**msp4**	Major surface antigen 4 precursor	Defense	**V**
AlbB_00433	**-**	CDS	-	**S**
AlbB_00444	**-**	CDS	-	**S**
AlbB_00452	**-**	CDS	-	**S**
AlbB_00457	**-**	CDS	-	**S**
AlbB_00482	**-**	Caudovirus prohead protease	Nucleotide transport and metabolism	**F**
AlbB_00483	**smc**	Chromosome partition protein Smc	Cell cycle control, cell division, chromosome partitioning	**D**
AlbB_00490	**-**	CDS	-	**S**
AlbB_00492	**-**	CDS	-	**S**
AlbB_00498	**-**	Ankyrin repeats (3 copies)	Ankyrin	**R**
AlbB_00505	**-**	CDS	-	**S**
AlbB_00510	**dnaJ**	chaperone protein DnaJ	Chaperone	**O**
AlbB_00528	**-**	CDS	-	**S**
AlbB_00531	**-**	Ankyrin repeats (3 copies)	Ankyrin	**R**
AlbB_00551	**bamA**	Outer membrane protein assembly factor BamA precursor	Membrane assembly	**M**
AlbB_00554	**-**	Cytochrome C and Quinol oxidase polypeptide I	Energy production and conversion	**C**
AlbB_00558	**smc**	Chromosome partition protein Smc	Cell cycle control, cell division, chromosome partitioning	**D**
AlbB_00560	**-**	Phage capsid family protein	Replication, recombination and repair	**L**
AlbB_00561	**-**	Phage capsid family protein	Replication, recombination and repair	**L**
AlbB_00569	**proS**	Proline--tRNA ligase	Aminoacyl-tRNA biosynthesis	**J**
AlbB_00572	**-**	CDS	-	**S**
AlbB_00576	**-**	CDS	-	**S**
AlbB_00611	**-**	CDS	-	**S**
AlbB_00621	**-**	CDS	-	**S**
AlbB_00622	**mutM**	Formamidopyrimidine-DNA glycosylase	Base excision repair	**L**
AlbB_00632	**-**	CDS	-	**S**
AlbB_00651	**-**	CDS	-	**S**
AlbB_00663	**-**	Putative NAD(P)H nitroreductase	Oxidative phosphorylation	**C**
AlbB_00669	**sugE**	Quaternary ammonium compound-resistance protein SugE	General function prediction only	**R**
AlbB_00692	**-**	CDS	-	**S**
AlbB_00714	**-**	CDS	-	**S**
AlbB_00725	**-**	short chain dehydrogenase	General function prediction only	**R**
AlbB_00734	**-**	CDS	-	**S**
AlbB_00754	**-**	CDS	-	**S**
AlbB_00758	**parA**	Chromosome partitioning protein ParA	Replication, recombination and repair	**L**
AlbB_00766	**-**	Ankyrin repeats (3 copies)	Ankyrin	**R**
AlbB_00774	**-**	Ankyrin repeat protein	Ankyrin	**R**
AlbB_00777	**ankX**	Phosphocholine transferase AnkX	Lipopolysaccharide biosynthesis	**I**
AlbB_00797	**-**	CDS	-	**S**
AlbB_00802	**gudP**	putative glucarate transporter	Posttranslational modification, protein turnover, chaperones	**O**
AlbB_00812	**dusC**	tRNA-dihydrouridine synthase C	Nitrotoluene degradation	**J**
AlbB_00837	**-**	EVE domain protein	-	**R**
AlbB_00845	**-**	CDS	-	**S**
AlbB_00847	**ribH1**	6,7-dimethyl-8-ribityllumazine synthase 1	Riboflavin metabolism	**H**
AlbB_00855	**-**	CDS	-	**S**
AlbB_00856	**swrC**	Swarming motility protein SwrC	Cell motility	**N**
AlbB_00861	**-**	CDS	-	**S**
AlbB_00863	**-**	CDS	-	**S**
AlbB_00868	**rmlD**	RmlD substrate binding domain protein	Cell wall/membrane/envelope biogenesis	**M**
AlbB_00879	**-**	CDS	-	**S**
AlbB_00886	**-**	CDS	-	**S**
AlbB_00899	**-**	CDS	-	**S**
AlbB_00900	**icaA**	N-glycosyltransferase	General function prediction only	**R**
AlbB_00905	**-**	CDS	-	**S**
AlbB_00907	**-**	CDS	-	**S**
AlbB_00909	**miaB**	(Dimethylallyl)adenosine tRNA methylthiotransferase MiaB	Puromycin biosynthesis	**J**
AlbB_00922	**-**	CDS	-	**S**
AlbB_00923	**-**	putative rhodanese-related sulfurtransferase	General function prediction only	**R**
AlbB_00928	**-**	CDS	-	**S**
AlbB_00935	**-**	Dna-J like membrane chaperone protein	Chaperone	**O**
AlbB_00938	**uvrB**	UvrABC system protein B	Nucleotide excision repair	**L**
AlbB_00953	**mdtC**	Multidrug resistance protein MdtC	Defense	**O**
AlbB_00959	**-**	PqqC-like protein	General function prediction only	**R**
AlbB_00960	**folB**	Dihydroneopterin aldolase	Folate biosynthesis	**H**

AlbB_00961	**folP**	Dihydropteroate synthase	Folate biosynthesis	**H**
AlbB_00962	**dfrA**	Dihydrofolate reductase	Folate biosynthesis	**H**
AlbB_00966	**-**	CDS	-	**S**
AlbB_00972	**-**	Ankyrin repeat protein	Ankyrin	**R**
AlbB_00980	**-**	CDS	-	**S**
AlbB_01015	**-**	CDS	-	**S**
AlbB_01016	**rpmJ**	50S ribosomal protein L36	Ribosome	**J**
AlbB_01031	**ribF**	Riboflavin biosynthesis protein RibF	Riboflavin biosynthesis	**H**
AlbB_01042	**-**	CDS	-	**S**
AlbB_01043	**pld**	Phospholipase D precursor	CAMP signaling pathway	**I**
AlbB_01050	**-**	CDS	-	**S**
AlbB_01074	**-**	Ankyrin repeats (3 copies)	Ankyrin	**R**
AlbB_01075	**-**	CDS	-	**S**
AlbB_01077	**-**	CDS	-	**S**
AlbB_01078	**-**	Collagen triple helix repeat (20 copies)	General function prediction only	**R**
AlbB_01100	**algA**	Alginate biosynthesis protein AlgA	Carbohydrate transport and metabolism	**G**
AlbB_01108	**cshA**	DEAD-box ATP-dependent RNA helicase CshA	RNA degradation	**L**
AlbB_01132	**uvrC**	UvrABC system protein C	Nucleotide excision repair	**L**
Anaa_00024	**-**	CDS	-	**S**
Anaa_00037	**-**	CDS	-	**S**
Anaa_00069	**-**	Ankyrin repeat protein	Ankyrin	**R**
Anaa_00135	**ykfA**	putative murein peptide carboxypeptidase	Phenylpropanoid biosynthesis	**R**
Anaa_00143	**-**	CDS	-	**S**
Anaa_00153	**-**	CDS	-	**S**
Anaa_00224	**tgt**	queuine tRNA-ribosyltransferase	Translation, ribosomal structure and biogenesis	**J**
Anaa_00247	**mrdA**	penicillin-binding protein 2	Defense	**M**
Anaa_00298	**-**	Ankyrin repeat protein	Ankyrin	**R**
Anaa_00342	**icd**	Isocitrate dehydrogenase [NADP]	Biosynthesis of amino acids	**C**
Anaa_00375	**-**	PD-(D/E)XK nuclease family transposase	Transposition	**L**
Anaa_00422	**-**	Bacterial SH3 domain protein	General function prediction only	**R**
Anaa_00438	**-**	Transposase IS66 family protein	Transposition	**L**
Anaa_00445	**-**	Ankyrin repeats (3 copies)	Ankyrin	**R**
Anaa_00475	**-**	CDS	-	**S**
Anaa_00492	**relG**	Toxin RelG	Mulitple COGs	**JD**
Anaa_00576	**-**	CDS	-	**S**
Anaa_00607	**-**	CDS	-	**S**
Anaa_00653	**-**	CDS	-	**S**
Anaa_00655	**rpmC**	50S ribosomal protein L29	Ribosome	**J**
Anaa_00677	**-**	RDD family protein	-	**R**
Anaa_00758	**-**	CDS	-	**S**
Anaa_00774	**-**	CDS	-	**S**
Anaa_00788	**hlyD**	HlyD family secretion protein	Intracellular trafficking, secretion, and vesicular transport	**U**
Anaa_00790	**fic**	Adenosine monophosphate-protein transferase SoFic	Cell cycle control, cell division, chromosome partitioning	**D**
Anaa_00798	**-**	CDS	-	**S**
Anaa_00801	**-**	CDS	-	**S**
Anaa_00804	**-**	CDS	-	**S**
Anaa_00814	**ftsW**	Lipid II flippase FtsW	Cell cycle control, cell division, chromosome partitioning	**D**
Anaa_00823	**-**	CDS	-	**S**
Anaa_00896	**-**	CDS	-	**S**
Anaa_00920	**-**	Transposase DDE domain protein	Transposition	**L**
Anaa_00939	**-**	MgtE intracellular N domain protein	Inorganic ion transport and metabolism	**P**
Anaa_00996	**-**	CDS	-	**S**
Anaa_01034	**-**	CDS	-	**S**
Anaa_01059	**-**	CDS	-	**S**
Anaa_01087	**-**	CDS	-	**S**
Anaa_01102	**ribA**	GTP cyclohydrolase-2	Riboflavin biosynthesis	**H**
Anaa_01113	**-**	PD-(D/E)XK nuclease family transposase	Transposition	**L**
Anaa_01125	**-**	Phage portal protein, lambda family	Replication, recombination and repair	**L**
Anaa_01138	**-**	IncA protein	General function prediction only	**R**
Anaa_01223	**-**	CDS	-	**S**
Anaa_01232	**smc**	Chromosome partition protein Smc	Cell cycle control, cell division, chromosome partitioning	**D**
Anaa_01253	**-**	Coenzyme F420:L-glutamate ligase	General function prediction only	**R**
Anaa_01285	**-**	CDS	-	**S**
Anaa_01336	**-**	CDS	-	**S**
Anaa_01362	**-**	CDS	-	**S**
Anaa_01382	**fic**	Adenosine monophosphate-protein transferase SoFic	Cell cycle control, cell division, chromosome partitioning	**D**
Anaa_01395	**-**	CDS	-	**S**
Anaa_01417	**-**	CDS	-	**S**
Anaa_01444	**-**	CDS	-	**S**
Anaa_01499	**-**	CDS	-	**S**
Anaa_01522	**-**	CDS	-	**S**
Anaa_01562	**-**	CDS	-	**S**
Anaa_01563	**fic**	Adenosine monophosphate-protein transferase SoFic	Cell cycle control, cell division, chromosome partitioning	**D**
Anaa_01586	**novR**	Decarboxylase NovR	General function prediction only	**R**
Anaa_01618	**-**	CDS	-	**S**
Anaa_01634	**-**	CDS	-	**S**
Anaa_01635	**-**	Phage gp6-like head-tail connector protein	Replication, recombination and repair	**L**
Anaa_01654	**ftsI**	Peptidoglycan synthase FtsI	Peptidoglycan biosynthesis	**M**
Anaa_01709	**-**	PD-(D/E)XK nuclease family transposase	Transposition	**L**
Anaa_01734	**-**	CDS	-	**S**
Anaa_01744	**-**	CDS	-	**S**

Anaa_01755	**-**	CDS	-	**S**
Anaa_01759	**-**	Archaeal ATPase	General function prediction only	**R**
Auuu_00253	**-**	anaerobic benzoate catabolism transcriptional regulator	DNA binding	**K**
Auuu_00274	**-**	CDS	-	**S**
Auuu_00377	**-**	Acetyltransferase (GNAT) family protein	Stress response	**R**
Auuu_00417	**-**	CDS	-	**S**
Auuu_00634	**-**	Ankyrin repeats (3 copies)	Ankyrin	**R**
Auuu_00635	**-**	Piwi/Argonaute/Zwille siRNA-binding domain protein	General function prediction only	**R**
Auuu_00718	**-**	Terminase-like family protein	General function prediction only	**R**
Auuu_00821	**-**	CDS	-	**S**
Auuu_00828	**-**	CDS	-	**S**
Auuu_00937	**-**	CDS	-	**S**
Auuu_00971	**-**	CDS	-	**S**
Auuu_01065	**-**	CDS	-	**S**
Auuu_01281	**-**	TrbC/VIRB2 family protein	General function prediction only	**R**
Bmmm_00026	**-**	CDS	-	**S**
Bmmm_00070	**-**	CDS	-	**S**
Bmmm_00097	**rocD**	Ornithine aminotransferase	Arginine and proline metabolism	**G**
Bmmm_00128	**-**	CDS	-	**S**
Bmmm_00233	**-**	CDS	-	**S**
Bmmm_00278	**-**	CDS	-	**S**
Bmmm_00419	**ankX**	Phosphocholine transferase AnkX	Lipopolysaccharide biosynthesis	**I**
Bmmm_00553	**-**	CDS	-	**S**
Bmmm_00582	**-**	CDS	-	**S**
Bmmm_00780	**-**	CDS	-	**S**
Bmmm_00861	**-**	CDS	-	**S**
Bmmm_00979	**-**	CDS	-	**S**
Bmmm_01004	**-**	CDS	-	**S**
Bmmm_01026	**-**	CDS	-	**S**
Bmmm_01051	**-**	Sodium:dicarboxylate symporter family protein	Carbohydrate transport and metabolism	**G**
Bmmm_01069	**-**	CDS	-	**S**
Bmmm_01125	**-**	CDS	-	**S**
Bmmm_01142	**-**	CDS	-	**S**
Clee_00160	**-**	CDS	-	**S**
Clee_00166	**-**	Ankyrin repeat protein	Ankyrin	**R**
Clee_00180	**-**	RDD family protein	-	**R**
Clee_00200	**-**	CDS	-	**S**
Clee_00261	**-**	CDS	-	**S**
Clee_00263	**tnpA**	Transposase for transposon Tn5	Transposition	**L**
Clee_00302	**-**	Ankyrin repeat protein	Ankyrin	**R**
Clee_00317	**-**	CDS	-	**S**
Clee_00318	**-**	CDS	-	**S**
Clee_00326	**-**	CDS	-	**S**
Clee_00447	**parE1**	Plasmid stabilisation system protein	General function prediction only	**R**
Clee_00479	**-**	putative transporter	Carbohydrate transport and metabolism	**G**
Clee_00525	**-**	TrbC/VIRB2 family protein	General function prediction only	**R**
Clee_00541	**-**	CDS	-	**S**
Clee_00613	**trkA**	potassium transporter peripheral membrane component	Inorganic ion transport and metabolism	**P**
Clee_00651	**-**	CDS	-	**S**
Clee_00772	**-**	CDS	-	**S**
Clee_00937	**-**	Ankyrin repeats (3 copies)	Ankyrin	**R**
Clee_01008	**-**	CDS	-	**S**
Clee_01104	**relG**	Toxin RelG	Mulitple COGs	**JD**
Clee_01120	**-**	CDS	-	**S**
Clee_01203	**-**	CDS	-	**S**
Clee_01211	**-**	flagellar assembly protein H	Cell motility	**N**
Clee_01295	**-**	CDS	-	**S**
Cocc_00100	**recG**	ATP-dependent DNA helicase RecG	Homologous recombination	**LK**
Cocc_00168	**-**	CDS	-	**S**
Cocc_00188	**lpd**	Dihydrolipoyl dehydrogenase	Carbon metabolism	**C**
Cocc_00200	**ttg2**	Toluene tolerance, Ttg2	ABC transporters	**P**
Cocc_00215	**putA**	Bifunctional protein PutA	Glutamate biosynthesis	**J**
Cocc_00289	**mlaE**	putative phospholipid ABC transporter permease protein MlaE	ABC transporters	**O**
Cocc_00344	**-**	CDS	-	**S**
Cocc_00996	**-**	CDS	-	**S**
Dimm_00144	**-**	CDS	-	**S**
Dimm_00694	**-**	CDS	-	**S**
Haaa_00253	**-**	CDS	-	**S**
Haaa_00255	**-**	CDS	-	**S**
Haaa_00260	**-**	Ankyrin repeats (3 copies)	Ankyrin	**R**
Haaa_00299	**-**	Phage tail protein (Tail P2 I)	General function prediction only	**R**
Haaa_00624	**-**	CDS	-	**S**
Haaa_00813	**-**	CDS	-	**S**
Lsss_00273	**-**	CDS	-	**S**
Lsss_00583	**-**	CDS	-	**S**
Lsss_00721	**-**	CDS	-	**S**
Lsss_00825	**-**	CDS	-	**S**
Mell_00260	**-**	CDS	-	**S**
Nooo_00062	**-**	CDS	-	**S**
Nooo_00156	**-**	CDS	-	**S**
Oooo_00222	**-**	CDS	-	**S**
Pipp_00016	**-**	CDS	-	**S**
Pipp_00034	**-**	CDS	-	**S**
Pipp_00073	**-**	CDS	-	**S**
Pipp_00196	**-**	Na+/H+ antiporter family protein	Energy production and conversion	**C**
Pipp_00260	**-**	putative transposase	Transposition	**L**
Pipp_00281	**-**	Archaeal ATPase	General function prediction only	**R**
Pipp_00293	**-**	Patatin-like phospholipase	General function prediction only	**R**

Pipp_00469	**-**	Phage late control gene D protein (GPD)	General function prediction only	**R**
Pipp_01366	**-**	CDS	-	**S**
Riii_00595	**relE**	Toxin RelE	Mulitple COGs	**JD**
Riii_00618	**ywqF**	UDP-glucose 6-dehydrogenase YwqF	Pentose and glucuronate interconversions	**C**
Riii_01141	**-**	Collagen triple helix repeat (20 copies)	General function prediction only	**R**

PANGENOME Venn: 1600 A+B Only

ID	Gene Symbol	Gene Product	Pathway or Process	COG
AlbB_00002	**-**	CDS	-	**S**
AlbB_00004	**-**	CDS	-	**S**
AlbB_00012	**-**	CDS	-	**S**
AlbB_00027	**-**	CDS	-	**S**
AlbB_00032	**-**	CDS	-	**S**
AlbB_00038	**-**	CDS	-	**S**
AlbB_00041	**-**	CDS	-	**S**
AlbB_00042	**-**	CDS	-	**S**
AlbB_00044	**-**	CDS	-	**S**
AlbB_00049	**-**	CDS	-	**S**
AlbB_00104	**-**	CDS	-	**S**
AlbB_00105	**-**	CDS	-	**S**
AlbB_00111	**-**	CDS	-	**S**
AlbB_00112	**-**	CDS	-	**S**
AlbB_00117	**-**	anaerobic benzoate catabolism transcriptional regulator	DNA binding	**K**
AlbB_00118	**-**	anaerobic benzoate catabolism transcriptional regulator	DNA binding	**K**
AlbB_00119	**-**	CDS	-	**S**
AlbB_00131	**-**	CDS	-	**S**
AlbB_00138	**-**	CDS	-	**S**
AlbB_00139	**-**	CDS	-	**S**
AlbB_00144	**-**	CDS	-	**S**
AlbB_00145	**-**	CDS	-	**S**
AlbB_00151	**-**	CDS	-	**S**
AlbB_00160	**-**	CDS	-	**S**
AlbB_00164	**-**	CDS	-	**S**
AlbB_00173	**-**	CDS	-	**S**
AlbB_00180	**-**	CDS	-	**S**
AlbB_00193	**ftsH**	ATP-dependent zinc metalloprotease FtsH	Protein modification	**O**
AlbB_00196	**-**	CDS	-	**S**
AlbB_00200	**-**	CDS	-	**S**
AlbB_00202	**dnaQ**	DNA polymerase III subunit epsilon	Purine metabolism	**L**
AlbB_00204	**-**	CDS	-	**S**
AlbB_00221	**-**	Ankyrin repeats (3 copies)	Ankyrin	**R**
AlbB_00223	**-**	CDS	-	**S**
AlbB_00240	**-**	CDS	-	**S**
AlbB_00241	**-**	CDS	-	**S**
AlbB_00244	**aacA-aphD**	Bifunctional AAC/APH	Fatty acid biosynthesis	**R**
AlbB_00246	**-**	CDS	-	**S**
AlbB_00247	**-**	CDS	-	**S**
AlbB_00259	**-**	CDS	-	**S**
AlbB_00263	**-**	Ankyrin repeats (3 copies)	Ankyrin	**R**
AlbB_00266	**acm**	Lysozyme M1 precursor	Two-component system	**M**
AlbB_00270	**-**	CDS	-	**S**
AlbB_00273	**sucB**	Dihydrolipoyllysine-residue succinyltransferase component of 2-oxoglutarate dehydrogenase complex	Carbon metabolism	**C**
AlbB_00291	**-**	Ankyrin repeats (3 copies)	Ankyrin	**R**
AlbB_00294	**-**	CDS	-	**S**
AlbB_00295	**-**	CDS	-	**S**
AlbB_00300	**-**	CDS	-	**S**
AlbB_00312	**-**	CDS	-	**S**
AlbB_00313	**-**	CDS	-	**S**
AlbB_00314	**-**	CDS	-	**S**
AlbB_00317	**-**	CDS	-	**S**
AlbB_00324	**ankX**	Phosphocholine transferase AnkX	Lipopolysaccharide biosynthesis	
AlbB_00329	**pld**	Phospholipase D precursor	CAMP signaling pathway	**I**
AlbB_00331	**-**	CDS	-	**S**
AlbB_00332	**-**	CDS	-	**S**
AlbB_00337	**-**	CDS	-	**S**
AlbB_00339	**-**	CDS	-	**S**
AlbB_00340	**-**	CDS	-	**S**
AlbB_00342	**der**	GTPase Der	Translation, ribosomal structure and biogenesis	**J**
AlbB_00343	**-**	CDS	-	**S**
AlbB_00349	**-**	CDS	-	**S**
AlbB_00353	**-**	CDS	-	**S**
AlbB_00355	**ftsH**	ATP-dependent zinc metalloprotease FtsH	Protein modification	**O**
AlbB_00356	**ftsH1**	ATP-dependent zinc metalloprotease FtsH 1	Protein modification	**P**
AlbB_00362	**-**	CDS	-	**S**
AlbB_00380	**-**	CDS	-	**S**
AlbB_00385	**-**	CDS	-	**S**
AlbB_00396	**-**	CDS	-	**S**
AlbB_00403	**-**	CDS	-	**S**
AlbB_00411	**-**	TrbC/VIRB2 family protein	General function prediction only	**R**
AlbB_00412	**-**	CDS	-	**S**
AlbB_00432	**-**	CDS	-	**S**
AlbB_00440	**-**	CDS	-	**S**
AlbB_00443	**-**	CDS	-	**S**
AlbB_00451	**-**	CDS	-	**S**

AlbB_00475	**-**	CDS	-	**S**
AlbB_00476	**-**	CDS	-	**S**
AlbB_00477	**-**	CDS	-	**S**
AlbB_00480	**-**	CDS	-	**S**
AlbB_00484	**-**	CDS	-	**S**
AlbB_00485	**-**	CDS	-	**S**
AlbB_00506	**-**	CDS	-	**S**
AlbB_00513	**-**	CDS	-	**S**
AlbB_00521	**-**	CDS	-	**S**
AlbB_00523	**-**	CDS	-	**S**
AlbB_00526	**-**	CDS	-	**S**
AlbB_00535	**exoD**	Exopolysaccharide synthesis, ExoD	General function prediction only	**R**
AlbB_00552	**-**	CDS	-	**S**
AlbB_00553	**-**	CDS	-	**S**
AlbB_00555	**-**	CDS	-	**S**
AlbB_00557	**smc**	Chromosome partition protein Smc	Cell cycle control, cell division, chromosome partitioning	**D**
AlbB_00562	**-**	CDS	-	**S**
AlbB_00581	**-**	CDS	-	**S**
AlbB_00586	**-**	anaerobic benzoate catabolism transcriptional regulator	DNA binding	**K**
AlbB_00595	**-**	A1 Propeptide	-	**R**
AlbB_00596	**-**	CDS	-	**S**
AlbB_00597	**-**	CDS	-	**S**
AlbB_00619	**-**	CDS	-	**S**
AlbB_00637	**ankX**	Phosphocholine transferase AnkX	Lipopolysaccharide biosynthesis	**I**
AlbB_00649	**-**	CDS	-	**S**
AlbB_00654	**-**	CDS	-	**S**
AlbB_00655	**-**	CDS	-	**S**
AlbB_00656	**-**	CDS	-	**S**
AlbB_00664	**-**	CDS	-	**S**
AlbB_00671	**-**	CDS	-	**S**
AlbB_00688	**-**	CDS	-	**S**
AlbB_00691	**-**	CDS	-	**S**
AlbB_00701	**-**	CDS	-	**S**
AlbB_00704	**-**	CDS	-	**S**
AlbB_00706	**-**	CDS	-	**S**
AlbB_00707	**-**	M61 glycyl aminopeptidase	General function prediction only	**R**
AlbB_00708	**-**	CDS	-	**S**
AlbB_00710	**-**	CDS	-	**S**
AlbB_00712	**-**	CDS	-	**S**
AlbB_00713	**yfmT**	Putative aldehyde dehydrogenase YfmT	beta-Alanine metabolism	**R**
AlbB_00722	**-**	CDS	-	**S**
AlbB_00745	**-**	CDS	-	**S**
AlbB_00746	**-**	CDS	-	**S**
AlbB_00757	**-**	CDS	-	**S**
AlbB_00769	**-**	CDS	-	**S**
AlbB_00771	**-**	Ankyrin repeats (3 copies)	Ankyrin	**R**
AlbB_00772	**-**	CDS	-	**S**
AlbB_00773	**-**	CDS	-	**S**
AlbB_00796	**-**	CDS	-	**S**
AlbB_00803	**-**	Ankyrin repeat protein	Ankyrin	**R**
AlbB_00804	**-**	Ankyrin repeat protein	Ankyrin	**R**
AlbB_00824	**-**	CDS	-	**S**
AlbB_00850	**-**	CDS	-	**S**
AlbB_00851	**-**	CDS	-	**S**
AlbB_00858	**-**	CDS	-	**S**
AlbB_00867	**ankX**	Phosphocholine transferase AnkX	Lipopolysaccharide biosynthesis	**I**
AlbB_00873	**-**	CDS	-	**S**
AlbB_00875	**-**	CDS	-	**S**
AlbB_00877	**-**	CDS	-	**S**
AlbB_00882	**-**	CDS	-	**S**
AlbB_00910	**-**	CDS	-	**S**
AlbB_00936	**-**	Ankyrin repeats (3 copies)	Ankyrin	**R**
AlbB_00937	**-**	Ankyrin repeats (3 copies)	Ankyrin	**R**
AlbB_00941	**-**	Ankyrin repeat protein	Ankyrin	**R**
AlbB_00975	**-**	CDS	-	**S**
AlbB_00976	**-**	CDS	-	**S**
AlbB_00985	**-**	CDS	-	**S**
AlbB_00989	**-**	CDS	-	**S**
AlbB_00990	**-**	CDS	-	**S**
AlbB_00991	**-**	CDS	-	**S**
AlbB_00994	**-**	CDS	-	**S**
AlbB_00996	**-**	CDS	-	**S**
AlbB_00999	**-**	CDS	-	**S**
AlbB_01000	**-**	CDS	-	**S**
AlbB_01002	**-**	CDS	-	**S**
AlbB_01003	**-**	CDS	-	**S**
AlbB_01008	**-**	CDS	-	**S**
AlbB_01012	**-**	CDS	-	**S**
AlbB_01019	**-**	CDS	-	**S**
AlbB_01025	**-**	CDS	-	**S**
AlbB_01028	**-**	CDS	-	**S**
AlbB_01038	**-**	Ankyrin repeat protein	Ankyrin	**R**
AlbB_01047	**-**	CDS	-	**S**
AlbB_01048	**-**	CDS	-	**S**
AlbB_01049	**-**	CDS	-	**S**
AlbB_01052	**trpS**	Tryptophan--tRNA ligase	Aminoacyl-tRNA biosynthesis	**J**
AlbB_01068	**-**	CDS	-	**S**

AlbB_01069	**-**	CDS	-	**S**
AlbB_01071	**-**	Phage P2 GpU	Replication, recombination and repair	**L**
AlbB_01072	**-**	Phage Tail Protein X	Replication, recombination and repair	**L**
AlbB_01076	**-**	CDS	-	**S**
AlbB_01082	**-**	CDS	-	**S**
AlbB_01110	**-**	CDS	-	**S**
AlbB_01113	**-**	CDS	-	**S**
AlbB_01124	**-**	CDS	-	**S**
Anaa_00001	**-**	Transposase IS66 family protein	Transposition	**L**
Anaa_00002	**-**	CDS	-	**S**
Anaa_00004	**rpmB**	50S ribosomal protein L28	Ribosome	**J**
Anaa_00007	**-**	CDS	-	**S**
Anaa_00008	**-**	CDS	-	**S**
Anaa_00009	**mrdA**	penicillin-binding protein 2	Cell wall/membrane/envelope biogenesis	**M**
Anaa_00010	**-**	CDS	-	**S**
Anaa_00011	**-**	CDS	-	**S**
Anaa_00012	**-**	PD-(D/E)XK nuclease family transposase	Transposition	**L**
Anaa_00013	**-**	CDS	-	**S**
Anaa_00016	**-**	CDS	-	**S**
Anaa_00017	**-**	CDS	-	**S**
Anaa_00018	**-**	Transposase IS66 family protein	Transposition	**L**
Anaa_00019	**-**	Transposase IS116/IS110/IS902 family protein	Transposition	**L**
Anaa_00020	**-**	Transposase IS116/IS110/IS902 family protein	Transposition	**L**
Anaa_00021	**pheS**	Phenylalanine--tRNA ligase alpha subunit	Aminoacyl-tRNA biosynthesis	**J**
Anaa_00022	**-**	CDS	-	**S**
Anaa_00023	**-**	CDS	-	**S**
Anaa_00025	**-**	CDS	-	**S**
Anaa_00026	**dnaG**	DNA primase	DNA replication	**L**
Anaa_00027	**pgdA**	Peptidoglycan-N-acetylglucosamine deacetylase	General function prediction only	**R**
Anaa_00028	**mtaB**	Threonylcarbamoyladenosine tRNA methylthiotransferase MtaB	Translation, ribosomal structure and biogenesis	**J**
Anaa_00030	**ctaG**	Cytochrome c oxidase assembly protein CtaG	Oxidative phosphorylation	**C**
Anaa_00033	**petB**	Cytochrome b	Oxidative phosphorylation	**C**
Anaa_00034	**rne**	Ribonuclease E	RNA degradation	**J**
Anaa_00041	**-**	CDS	-	**S**
Anaa_00043	**lipA**	Lipoyl synthase	Lipoate biosynthesis	**H**
Anaa_00044	**secA**	preprotein translocase subunit SecA	Bacterial secretion system	**MU**
Anaa_00045	**guaA**	GMP synthase [glutamine-hydrolyzing]	Purine metabolism	**F**
Anaa_00047	**-**	CDS	-	**S**
Anaa_00050	**-**	CDS	-	**S**
Anaa_00052	**-**	CDS	-	**S**
Anaa_00055	**-**	Transposase IS66 family protein	Transposition	**L**
Anaa_00056	**sdhB**	Succinate dehydrogenase iron-sulfur subunit	Butanoate metabolism	**C**
Anaa_00057	**-**	CDS	-	**S**
Anaa_00059	**-**	CDS	-	**S**
Anaa_00060	**-**	CDS	-	**S**
Anaa_00061	**-**	Transposase	Transposition	**L**
Anaa_00062	**-**	CDS	-	**S**
Anaa_00064	**-**	Transposase IS66 family protein	Transposition	**L**
Anaa_00066	**metK**	S-adenosylmethionine synthase	Biosynthesis of amino acids	**H**
Anaa_00070	**trxB**	Thioredoxin reductase	Pyrimidine metabolism	**O**
Anaa_00071	**-**	CDS	-	**S**
Anaa_00072	**comM**	Competence protein ComM	Replication, recombination and repair	**L**
Anaa_00073	**-**	CDS	-	**S**
Anaa_00074	**murE**	UDP-N-acetylmuramoyl-L-alanyl-D-glutamate--2,6-diaminopimelate ligase	Lysine biosynthesis	**M**
Anaa_00076	**-**	CDS	-	**S**
Anaa_00080	**rplF**	50S ribosomal protein L6	Ribosome	**J**
Anaa_00081	**rpsH**	30S ribosomal protein S8	Ribosome	**J**
Anaa_00086	**-**	PD-(D/E)XK nuclease family transposase	Transposition	**L**
Anaa_00089	**zitB**	Zinc transporter ZitB	Inorganic ion transport and metabolism	**P**
Anaa_00090	**-**	CDS	-	**S**
Anaa_00093	**murE**	UDP-N-acetylmuramoyl-L-alanyl-D-glutamate--2,6-diaminopimelate ligase	Lysine biosynthesis	**M**
Anaa_00095	**nrdB**	Ribonucleoside-diphosphate reductase subunit beta	Purine metabolism	**R**
Anaa_00096	**-**	CDS	-	**S**
Anaa_00097	**-**	CDS	-	**S**
Anaa_00099	**slyD**	FKBP-type peptidyl-prolyl cis-trans isomerase	General function prediction only	**R**
Anaa_00100	**-**	CDS	-	**S**
Anaa_00101	**uvrA**	UvrABC system protein A	Nucleotide excision repair	**L**
Anaa_00102	**-**	Transposase IS66 family protein	Transposition	**L**
Anaa_00103	**uvrB**	UvrABC system protein B	Nucleotide excision repair	**L**
Anaa_00104	**fabF**	3-oxoacyl-[acyl-carrier-protein] synthase 2	Fatty acid biosynthesis	**I**
Anaa_00106	**-**	CDS	-	**S**
Anaa_00108	**-**	CDS	-	**S**
Anaa_00109	**-**	CDS	-	**S**
Anaa_00112	**-**	CDS	-	**S**
Anaa_00114	**-**	CDS	-	**S**
Anaa_00115	**-**	CDS	-	**S**
Anaa_00116	**-**	Na+/H+ antiporter family protein	Energy production and conversion	**C**
Anaa_00117	**-**	CDS	-	**S**
Anaa_00118	**-**	CDS	-	**S**
Anaa_00119	**-**	Phage terminase large subunit (GpA)	Replication, recombination and repair	**L**
Anaa_00124	**ubiD**	3-octaprenyl-4-hydroxybenzoate carboxy-lyase	Ubiquinone and other terpenoid-quinone biosynthesis	**H**
Anaa_00128	**recG**	ATP-dependent DNA helicase RecG	Homologous recombination	**LK**
Anaa_00130	**-**	CDS	-	**S**

Anaa_00131	**-**	CDS	-	**S**
Anaa_00132	**-**	Integrase core domain protein	General function prediction only	**R**
Anaa_00133	**tsaD**	tRNA N6-adenosine threonylcarbamoyltransferase	Translation	**J**
Anaa_00136	**-**	CDS	-	**S**
Anaa_00137	**-**	Reverse transcriptase (RNA-dependent DNA polymerase)	Reverse transcriptase	**R**
Anaa_00138	**-**	CDS	-	**S**
Anaa_00139	**-**	CDS	-	**S**
Anaa_00141	**-**	CDS	-	**S**
Anaa_00144	**rpsA**	30S ribosomal protein S1	Ribosome	**C**
Anaa_00145	**pyrE**	Orotate phosphoribosyltransferase	Pyrimidine metabolism	**F**
Anaa_00146	**typA**	GTP-binding protein TypA/BipA	Signal transduction mechanisms	**T**
Anaa_00147	**-**	CDS	-	**S**
Anaa_00148	**-**	CDS	-	**S**
Anaa_00151	**nuoD**	NADH-quinone oxidoreductase subunit D	Oxidative phosphorylation	**C**
Anaa_00152	**-**	Transposase	Transposition	**L**
Anaa_00155	**-**	CDS	-	**S**
Anaa_00156	**-**	Phospholipase/Carboxylesterase	General function prediction only	**R**
Anaa_00159	**-**	CDS	-	**S**
Anaa_00160	**thrS**	Threonine--tRNA ligase	Aminoacyl-tRNA biosynthesis	**J**
Anaa_00161	**-**	CDS	-	**S**
Anaa_00162	**gap**	Glyceraldehyde-3-phosphate dehydrogenase	Biosynthesis of amino acids	**E**
Anaa_00163	**fgs**	Folylpolyglutamate synthase	Folate biosynthesis	**C**
Anaa_00168	**-**	PD-(D/E)XK nuclease family transposase	Transposition	**L**
Anaa_00169	**-**	Group II intron, maturase-specific domain	General function prediction only	**R**
Anaa_00170	**-**	CDS	-	**S**
Anaa_00171	**rir2**	ribonucleotide-diphosphate reductase subunit beta	General function prediction only	**R**
Anaa_00173	**-**	Transposase	Transposition	**L**
Anaa_00175	**-**	CDS	-	**S**
Anaa_00177	**murE**	UDP-N-acetylmuramoyl-L-alanyl-D-glutamate--2,6-diaminopimelate ligase	Lysine biosynthesis	**M**
Anaa_00178	**hslU**	ATP-dependent protease ATPase subunit HslU	Posttranslational modification, protein turnover, chaperones	**O**
Anaa_00179	**-**	TrbL/VirB6 plasmid conjugal transfer protein	General function prediction only	**R**
Anaa_00180	**-**	CDS	-	**S**
Anaa_00181	**-**	CDS	-	**S**
Anaa_00182	**-**	CDS	-	**S**
Anaa_00183	**-**	TrbL/VirB6 plasmid conjugal transfer protein	General function prediction only	**R**
Anaa_00191	**plsX**	Phosphate acyltransferase	Glycerolipid metabolism	**I**
Anaa_00192	**-**	CDS	-	**S**
Anaa_00193	**-**	CDS	-	**S**
Anaa_00194	**-**	CDS	-	**S**
Anaa_00195	**-**	CDS	-	**S**
Anaa_00197	**uvrA**	UvrABC system protein A	Nucleotide excision repair	**L**
Anaa_00201	**-**	Ankyrin repeats (3 copies)	Ankyrin	**R**
Anaa_00202	**hslU**	ATP-dependent protease ATPase subunit HslU	Posttranslational modification, protein turnover, chaperones	**O**
Anaa_00203	**hslU**	ATP-dependent protease ATPase subunit HslU	Posttranslational modification, protein turnover, chaperones	**O**
Anaa_00204	**pepQ**	Xaa-Pro dipeptidase	Amino acid transport and metabolism	**E**
Anaa_00205	**-**	CDS	-	**S**
Anaa_00206	**-**	CDS	-	**S**
Anaa_00209	**-**	CDS	-	**S**
Anaa_00216	**-**	CDS	-	**S**
Anaa_00217	**-**	CDS	-	**S**
Anaa_00220	**pipB2**	Secreted effector protein pipB2	Secondary metabolites biosynthesis, transport and catabolism	**Q**
Anaa_00221	**proV**	Glycine betaine/L-proline transport ATP-binding protein ProV	ABC transporters	**E**
Anaa_00222	**infC**	Translation initiation factor IF-3	Translation, ribosomal structure and biogenesis	**J**
Anaa_00227	**rplE**	50S ribosomal protein L5	Ribosome	**J**
Anaa_00228	**rpsN**	30S ribosomal protein S14	Ribosome	**J**
Anaa_00230	**-**	PAAR motif protein	Cell wall/membrane/envelope biogenesis	**M**
Anaa_00231	**-**	Gene 25-like lysozyme	-	**R**
Anaa_00232	**-**	SPFH domain / Band 7 family protein	General function prediction only	**R**
Anaa_00233	**dnaJ**	chaperone protein DnaJ	Chaperone	**O**
Anaa_00234	**-**	CDS	-	**S**
Anaa_00240	**dnaX**	DNA polymerase III subunit tau	Purine metabolism	**L**
Anaa_00243	**rplJ**	50S ribosomal protein L10	Ribosome	**J**
Anaa_00245	**rpoBC**	Bifunctional DNA-directed RNA polymerase subunit beta-beta'	Transcription	**K**
Anaa_00249	**fabF**	3-oxoacyl-[acyl-carrier-protein] synthase 2	Fatty acid biosynthesis	**I**
Anaa_00250	**-**	CDS	-	**S**
Anaa_00251	**-**	Coenzyme F420:L-glutamate ligase	General function prediction only	**R**
Anaa_00253	**-**	CDS	-	**S**
Anaa_00254	**purN**	Phosphoribosylglycinamide formyltransferase	Purine metabolism	**F**
Anaa_00255	**-**	CDS	-	**S**
Anaa_00257	**-**	CDS	-	**S**
Anaa_00260	**-**	Phage-related minor tail protein	Replication, recombination and repair	**L**
Anaa_00261	**-**	CDS	-	**S**
Anaa_00262	**-**	CDS	-	**S**
Anaa_00265	**-**	CDS	-	**S**
Anaa_00268	**-**	Blue-light-activated protein	General function prediction only	**R**
Anaa_00270	**thrS**	Threonine--tRNA ligase	Aminoacyl-tRNA biosynthesis	**J**
Anaa_00271	**infC**	Translation initiation factor IF-3	Translation, ribosomal structure and biogenesis	**J**
Anaa_00272	**-**	CDS	-	**S**
Anaa_00275	**yabA**	DNA replication intiation control protein YabA	Replication	**L**
Anaa_00276	**-**	CDS	-	**S**

Anaa_00277	**-**	CDS	-	**S**
Anaa_00278	**acn**	Aconitate hydratase	Biosynthesis of amino acids	**C**
Anaa_00279	**-**	CDS	-	**S**
Anaa_00282	**-**	CDS	-	**S**
Anaa_00284	**-**	CDS	-	**S**
Anaa_00287	**-**	CDS	-	**S**
Anaa_00288	**-**	PD-(D/E)XK nuclease family transposase	Transposition	**L**
Anaa_00292	**ask**	Aspartokinase	Lysine biosynthesis	**E**
Anaa_00294	**hemC**	Porphobilinogen deaminase	Heme biosynthesis	**H**
Anaa_00297	**-**	CDS	-	**S**
Anaa_00299	**-**	CDS	-	**S**
Anaa_00300	**murB**	UDP-N-acetylenolpyruvoylglucosamine reductase	Peptidoglycan biosynthesis	**M**
Anaa_00302	**dnaX**	DNA polymerase III subunit tau	Purine metabolism	**L**
Anaa_00304	**pld**	Phospholipase D precursor	CAMP signaling pathway	**I**
Anaa_00307	**dnaG**	DNA primase	DNA replication	**L**
Anaa_00309	**ftsH**	ATP-dependent zinc metalloprotease FtsH	Protein modification	**O**
Anaa_00310	**ispH**	4-hydroxy-3-methylbut-2-enyl diphosphate reductase	Terpenoid backbone biosynthesis	**I**
Anaa_00312	**glmU**	bifunctional N-acetylglucosamine-1-phosphate uridyltransferase/glucosamine-1-phosphate acetyltransferase	Amino sugar and nucleotide sugar metabolism	**M**
Anaa_00315	**leuS**	Leucine--tRNA ligase	Aminoacyl-tRNA biosynthesis	**J**
Anaa_00317	**-**	CDS	-	**S**
Anaa_00319	**-**	CDS	-	**S**
Anaa_00320	**-**	CDS	-	**S**
Anaa_00322	**pstA**	Phosphate transport system permease protein PstA	ABC transporters	**P**
Anaa_00323	**atpD**	ATP synthase subunit beta	Oxidative phosphorylation	**C**
Anaa_00324	**truA**	tRNA pseudouridine synthase A	Translation, ribosomal structure and biogenesis	**J**
Anaa_00325	**-**	CDS	-	**S**
Anaa_00327	**ubiD**	3-octaprenyl-4-hydroxybenzoate carboxy-lyase	Ubiquinone and other terpenoid-quinone biosynthesis	**H**
Anaa_00329	**adk**	adenylate kinase	Purine metabolism	**F**
Anaa_00331	**typA**	GTP-binding protein TypA/BipA	Signal transduction mechanisms	**T**
Anaa_00333	**dnaJ**	chaperone protein DnaJ	Chaperone	**O**
Anaa_00337	**rplJ**	50S ribosomal protein L10	Ribosome	**J**
Anaa_00338	**-**	CDS	-	**S**
Anaa_00339	**-**	CDS	-	**S**
Anaa_00340	**hslU**	ATP-dependent protease ATPase subunit HslU	Posttranslational modification, protein turnover, chaperones	**O**
Anaa_00343	**-**	CDS	-	**S**
Anaa_00345	**-**	CDS	-	**S**
Anaa_00346	**-**	CDS	-	**S**
Anaa_00348	**-**	CDS	-	**S**
Anaa_00350	**-**	putative monovalent cation/H+ antiporter subunit B	Bacterial secretion system	**U**
Anaa_00351	**-**	CDS	-	**S**
Anaa_00354	**-**	CDS	-	**S**
Anaa_00355	**-**	CDS	-	**S**
Anaa_00356	**-**	CDS	-	**S**
Anaa_00358	**ftsW**	Lipid II flippase FtsW	Cell cycle control, cell division, chromosome partitioning	**D**
Anaa_00359	**exoD**	Exopolysaccharide synthesis, ExoD	General function prediction only	**R**
Anaa_00360	**rpoD**	RNA polymerase sigma factor RpoD	Transcription	**K**
Anaa_00362	**-**	CDS	-	**S**
Anaa_00364	**dnaN**	DNA polymerase III subunit beta	Purine metabolism	**L**
Anaa_00366	**rpoH**	RNA polymerase sigma factor RpoH	Transcription	**K**
Anaa_00367	**-**	Transposase	Transposition	**L**
Anaa_00371	**-**	Na+/H+ antiporter family protein	Energy production and conversion	**C**
Anaa_00376	**-**	CDS	-	**S**
Anaa_00377	**ftsK**	DNA translocase FtsK	Cell cycle control, cell division, chromosome partitioning	**D**
Anaa_00379	**uvrA**	UvrABC system protein A	Nucleotide excision repair	**L**
Anaa_00380	**uvrA**	UvrABC system protein A	Nucleotide excision repair	**L**
Anaa_00386	**-**	CDS	-	**S**
Anaa_00388	**ppdK**	Pyruvate, phosphate dikinase	Carbon metabolism	**C**
Anaa_00389	**rplF**	50S ribosomal protein L6	Ribosome	**J**
Anaa_00390	**rplR**	50S ribosomal protein L18	Ribosome	**J**
Anaa_00392	**-**	CDS	-	**S**
Anaa_00397	**ubiD**	3-octaprenyl-4-hydroxybenzoate carboxy-lyase	Ubiquinone and other terpenoid-quinone biosynthesis	**H**
Anaa_00400	**argS**	Arginine--tRNA ligase	Aminoacyl-tRNA biosynthesis	**J**
Anaa_00403	**-**	Transposase IS66 family protein	Transposition	**L**
Anaa_00404	**-**	CDS	-	**S**
Anaa_00405	**-**	CDS	-	**S**
Anaa_00406	**-**	CDS	-	**S**
Anaa_00407	**artM**	Arginine transport ATP-binding protein ArtM	ABC transporters	**E**
Anaa_00408	**-**	CDS	-	**S**
Anaa_00411	**-**	Helix-turn-helix	General function prediction only	**R**
Anaa_00412	**tktA**	Transketolase 1	Biosynthesis of amino acids	**G**
Anaa_00413	**-**	CDS	-	**S**
Anaa_00415	**rpsA**	30S ribosomal protein S1	Ribosome	**C**
Anaa_00417	**-**	CDS	-	**S**
Anaa_00418	**-**	CDS	-	**S**
Anaa_00420	**lipB**	Octanoyltransferase	Lipoate biosynthesis	**H**
Anaa_00421	**pdhC**	Dihydrolipoyllysine-residue acetyltransferase component of pyruvate dehydrogenase complex	Citrate cycle (TCA cycle)	**C**
Anaa_00424	**comM**	Competence protein ComM	Replication, recombination and repair	**L**
Anaa_00427	**-**	CDS	-	**S**
Anaa_00432	**pdhC**	Dihydrolipoyllysine-residue acetyltransferase component of pyruvate dehydrogenase complex	Citrate cycle (TCA cycle)	**C**

Anaa_00433	**pdhC**	Dihydrolipoyllysine-residue acetyltransferase component of pyruvate dehydrogenase complex	Citrate cycle (TCA cycle)	**C**
Anaa_00434	**-**	CDS	-	**S**
Anaa_00436	**petB**	Cytochrome b	Oxidative phosphorylation	**C**
Anaa_00440	**-**	pyruvate dehydrogenase subunit beta	Energy production and conversion	**C**
Anaa_00441	**-**	CDS	-	**S**
Anaa_00442	**-**	Phage portal protein, lambda family	Replication, recombination and repair	**L**
Anaa_00443	**-**	Phage-related baseplate assembly protein	Replication, recombination and repair	**L**
Anaa_00444	**-**	CDS	-	**S**
Anaa_00456	**-**	CDS	-	**S**
Anaa_00457	**-**	Group II intron, maturase-specific domain	General function prediction only	**R**
Anaa_00459	**-**	CDS	-	**S**
Anaa_00462	**-**	Transposase IS66 family protein	Transposition	**L**
Anaa_00463	**-**	CDS	-	**S**
Anaa_00469	**ubiB**	putative protein kinase UbiB	Glycosylphosphatidylinositol(GPI)-anchor biosynthesis	**HC**
Anaa_00472	**tktA**	Transketolase 1	Biosynthesis of amino acids	**G**
Anaa_00474	**pdhC**	Dihydrolipoyllysine-residue acetyltransferase component of pyruvate dehydrogenase complex	Citrate cycle (TCA cycle)	**C**
Anaa_00476	**pdhC**	Dihydrolipoyllysine-residue acetyltransferase component of pyruvate dehydrogenase complex	Citrate cycle (TCA cycle)	**C**
Anaa_00477	**-**	Bacterial SH3 domain protein	General function prediction only	**R**
Anaa_00478	**-**	PD-(D/E)XK nuclease family transposase	Transposition	**L**
Anaa_00479	**-**	CDS	-	**S**
Anaa_00480	**-**	CDS	-	**S**
Anaa_00481	**argS**	Arginine--tRNA ligase	Aminoacyl-tRNA biosynthesis	**J**
Anaa_00486	**clpA**	ATP-dependent Clp protease ATP-binding subunit ClpA	Misfolded protein degradation	**O**
Anaa_00490	**ftsK**	DNA translocase FtsK	Cell cycle control, cell division, chromosome partitioning	**D**
Anaa_00494	**-**	CDS	-	**S**
Anaa_00496	**ltrA**	Group II intron-encoded protein LtrA	Amino acid transport and metabolism	**E**
Anaa_00497	**cycA**	Cytochrome c2	Oxidative phosphorylation	**C**
Anaa_00498	**hemB**	Delta-aminolevulinic acid dehydratase	Heme biosynthesis	**H**
Anaa_00499	**-**	CDS	-	**S**
Anaa_00500	**-**	CDS	-	**S**
Anaa_00501	**-**	CDS	-	**S**
Anaa_00502	**-**	CDS	-	**S**
Anaa_00503	**yabA**	DNA replication intiation control protein YabA	Replication	**L**
Anaa_00504	**-**	CDS	-	**S**
Anaa_00505	**-**	Transposase IS116/IS110/IS902 family protein	Transposition	**L**
Anaa_00513	**-**	CDS	-	**S**
Anaa_00514	**tsaD**	tRNA N6-adenosine threonylcarbamoyltransferase	Translation	**J**
Anaa_00515	**-**	CDS	-	**S**
Anaa_00519	**dnaN**	DNA polymerase III subunit beta	Purine metabolism	**L**
Anaa_00526	**putA**	Bifunctional protein PutA	Glutamate biosynthesis	**J**
Anaa_00527	**tig**	Trigger factor	Chaperone	**O**
Anaa_00530	**rpoH**	RNA polymerase sigma factor RpoH	Transcription	**K**
Anaa_00532	**aspS**	Aspartate--tRNA ligase	Aminoacyl-tRNA biosynthesis	**J**
Anaa_00533	**acn**	Aconitate hydratase	Biosynthesis of amino acids	**C**
Anaa_00535	**-**	membrane protein insertase	Intracellular trafficking, secretion, and vesicular transport	**U**
Anaa_00536	**-**	CDS	-	**S**
Anaa_00538	**-**	Transposase IS66 family protein	Transposition	**L**
Anaa_00539	**-**	CDS	-	**S**
Anaa_00542	**-**	CDS	-	**S**
Anaa_00545	**-**	CDS	-	**S**
Anaa_00546	**glyA1**	Serine hydroxymethyltransferase 1	Mulitple COGs	**HE**
Anaa_00547	**-**	PD-(D/E)XK nuclease family transposase	Transposition	**L**
Anaa_00550	**-**	Phage portal protein, lambda family	Replication, recombination and repair	**L**
Anaa_00551	**sppA**	Putative signal peptide peptidase SppA	beta-Lactam resistance	**OU**
Anaa_00552	**-**	CDS	-	**S**
Anaa_00554	**-**	CDS	-	**S**
Anaa_00558	**-**	CDS	-	**S**
Anaa_00560	**-**	Integrase core domain protein	General function prediction only	**R**
Anaa_00561	**dnaX**	DNA polymerase III subunits gamma and tau	Purine metabolism	**L**
Anaa_00563	**-**	Surface antigen	Defense	**R**
Anaa_00564	**-**	CDS	-	**S**
Anaa_00567	**atpA**	ATP synthase subunit alpha	Oxidative phosphorylation	**C**
Anaa_00568	**atpA**	ATP synthase subunit alpha	Oxidative phosphorylation	**C**
Anaa_00569	**-**	CDS	-	**S**
Anaa_00573	**fabG**	3-oxoacyl-[acyl-carrier-protein] reductase FabG	Biosynthesis of unsaturated fatty acids	**I**
Anaa_00575	**-**	tetratricopeptide repeat protein	General function prediction only	**R**
Anaa_00579	**-**	CDS	-	**S**
Anaa_00580	**-**	CDS	-	**S**
Anaa_00583	**dgkA**	Diacylglycerol kinase	Glycerolipid metabolism	**M**
Anaa_00584	**-**	CDS	-	**S**
Anaa_00586	**tlyC**	Hemolysin C	General function prediction only	**R**
Anaa_00587	**-**	CDS	-	**S**
Anaa_00588	**-**	Bacterial SH3 domain protein	General function prediction only	**R**
Anaa_00594	**-**	CDS	-	**S**
Anaa_00601	**cydB**	Cytochrome bd-I ubiquinol oxidase subunit 2	Oxidative phosphorylation	**C**
Anaa_00602	**-**	CDS	-	**S**
Anaa_00604	**-**	CDS	-	**S**
Anaa_00608	**-**	CDS	-	**S**
Anaa_00616	**corC**	Magnesium and cobalt efflux protein CorC	Ion transport	**P**
Anaa_00618	**virB9**	Type IV secretion system protein virB9 precursor	Bacterial secretion system	**U**
Anaa_00621	**-**	Transposase IS66 family protein	Transposition	**L**
Anaa_00623	**-**	CDS	-	**S**

Anaa_00624	**-**	CDS	-	**S**
Anaa_00628	**ffh**	Signal recognition particle protein	Bacterial secretion system	**U**
Anaa_00629	**-**	CDS	-	**S**
Anaa_00633	**pyrC**	Dihydroorotase	Pyrimidine metabolism	**F**
Anaa_00634	**-**	CDS	-	**S**
Anaa_00635	**-**	CDS	-	**S**
Anaa_00638	**-**	CDS	-	**S**
Anaa_00642	**-**	CDS	-	**S**
Anaa_00643	**-**	CDS	-	**S**
Anaa_00646	**hisS**	histidyl-tRNA synthetase	Aminoacyl-tRNA biosynthesis	**J**
Anaa_00647	**-**	CDS	-	**S**
Anaa_00648	**plsX**	Phosphate acyltransferase	Glycerolipid metabolism	**I**
Anaa_00649	**ltrA**	Group II intron-encoded protein LtrA	Amino acid transport and metabolism	**E**
Anaa_00651	**-**	CDS	-	**S**
Anaa_00652	**-**	CDS	-	**S**
Anaa_00654	**rplP**	50S ribosomal protein L16	Ribosome	**J**
Anaa_00665	**truA**	tRNA pseudouridine synthase A	Translation, ribosomal structure and biogenesis	**J**
Anaa_00670	**-**	CDS	-	**S**
Anaa_00671	**-**	CDS	-	**S**
Anaa_00679	**-**	CDS	-	**S**
Anaa_00683	**rpsC**	30S ribosomal protein S3	Ribosome	**J**
Anaa_00684	**-**	CDS	-	**S**
Anaa_00688	**rpsQ**	30S ribosomal protein S17	Ribosome	**J**
Anaa_00690	**-**	Integrase core domain protein	General function prediction only	**R**
Anaa_00691	**-**	Gypsy protein	General function prediction only	**R**
Anaa_00698	**-**	CDS	-	**S**
Anaa_00701	**trxB**	Thioredoxin reductase	Pyrimidine metabolism	**O**
Anaa_00705	**-**	CDS	-	**S**
Anaa_00706	**-**	Polyprenyl synthetase	General function prediction only	**R**
Anaa_00710	**-**	CDS	-	**S**
Anaa_00712	**-**	anaerobic benzoate catabolism transcriptional regulator	DNA binding	**K**
Anaa_00713	**mnmG**	tRNA uridine 5-carboxymethylaminomethyl modification enzyme MnmG	Translation, ribosomal structure and biogenesis	**J**
Anaa_00714	**-**	CDS	-	**S**
Anaa_00721	**-**	CDS	-	**S**
Anaa_00723	**-**	CDS	-	**S**
Anaa_00725	**yabA**	DNA replication intiation control protein YabA	Replication	**L**
Anaa_00730	**-**	CDS	-	**S**
Anaa_00732	**-**	CDS	-	**S**
Anaa_00738	**murE**	UDP-N-acetylmuramoyl-L-alanyl-D-glutamate--2,6-diaminopimelate ligase	Lysine biosynthesis	**M**
Anaa_00739	**-**	CDS	-	**S**
Anaa_00743	**-**	CDS	-	**S**
Anaa_00747	**-**	CDS	-	**S**
Anaa_00748	**-**	Integrase core domain protein	General function prediction only	**R**
Anaa_00749	**-**	CDS	-	**S**
Anaa_00752	**ffh**	Signal recognition particle protein	Bacterial secretion system	**U**
Anaa_00753	**-**	PD-(D/E)XK nuclease family transposase	Transposition	**L**
Anaa_00755	**-**	CDS	-	**S**
Anaa_00759	**-**	Ankyrin repeat protein	Ankyrin	**R**
Anaa_00760	**plsX**	Phosphate acyltransferase	Glycerolipid metabolism	**I**
Anaa_00763	**glnQ**	Glutamine transport ATP-binding protein GlnQ	ABC transporters	**E**
Anaa_00764	**-**	Transposase IS66 family protein	Transposition	**L**
Anaa_00766	**-**	Ankyrin repeats (3 copies)	Ankyrin	**R**
Anaa_00768	**-**	Transposase IS66 family protein	Transposition	**L**
Anaa_00777	**-**	CDS	-	**S**
Anaa_00779	**-**	TrbC/VIRB2 family protein	General function prediction only	**R**
Anaa_00782	**-**	Transposase	Transposition	**L**
Anaa_00784	**-**	CDS	-	**S**
Anaa_00786	**hepT**	Heptaprenyl diphosphate synthase component 2	Terpenoid backbone biosynthesis	**K**
Anaa_00787	**-**	CDS	-	**S**
Anaa_00791	**-**	CDS	-	**S**
Anaa_00797	**dapD**	2,3,4,5-tetrahydropyridine-2,6-dicarboxylate N-succinyltransferase	Lysine biosynthesis	**E**
Anaa_00803	**sdhB**	Succinate dehydrogenase iron-sulfur subunit	Butanoate metabolism	**C**
Anaa_00807	**-**	CDS	-	**S**
Anaa_00808	**-**	Gypsy protein	General function prediction only	**R**
Anaa_00809	**-**	Gypsy protein	General function prediction only	**R**
Anaa_00810	**-**	CDS	-	**S**
Anaa_00812	**lipA**	Lipoyl synthase	Lipoate biosynthesis	**H**
Anaa_00816	**-**	CDS	-	**S**
Anaa_00821	**ldcA**	L,D-carboxypeptidase A	Defense mechanisms	**V**
Anaa_00822	**-**	CDS	-	**S**
Anaa_00827	**leuS**	Leucine--tRNA ligase	Aminoacyl-tRNA biosynthesis	**J**
Anaa_00831	**mnmG**	tRNA uridine 5-carboxymethylaminomethyl modification enzyme MnmG	Translation, ribosomal structure and biogenesis	**J**
Anaa_00842	**-**	Baseplate J-like protein	General function prediction only	**R**
Anaa_00843	**-**	Phage tail protein (Tail P2 I)	General function prediction only	**R**
Anaa_00846	**petB**	Cytochrome b	Oxidative phosphorylation	**C**
Anaa_00847	**petB**	Cytochrome b	Oxidative phosphorylation	**C**
Anaa_00848	**-**	CDS	-	**S**
Anaa_00855	**-**	PD-(D/E)XK nuclease family transposase	Transposition	**L**
Anaa_00858	**-**	CDS	-	**S**
Anaa_00859	**ltrA**	Group II intron-encoded protein LtrA	Amino acid transport and metabolism	**E**
Anaa_00860	**-**	CDS	-	**S**
Anaa_00862	**-**	CDS	-	**S**
Anaa_00865	**-**	CDS	-	**S**

Anaa_00868	**-**	CDS	-	**S**
Anaa_00869	**-**	Transposase IS116/IS110/IS902 family protein	Transposition	**L**
Anaa_00870	**yabA**	DNA replication intiation control protein YabA	Replication	**L**
Anaa_00883	**yabA**	DNA replication intiation control protein YabA	Replication	**L**
Anaa_00895	**hemC**	Porphobilinogen deaminase	Heme biosynthesis	**H**
Anaa_00897	**secA**	preprotein translocase subunit SecA	Bacterial secretion system	**MU**
Anaa_00898	**-**	CDS	-	**S**
Anaa_00912	**-**	CDS	-	**S**
Anaa_00919	**-**	CDS	-	**S**
Anaa_00925	**tir**	CAAX amino terminal protease self- immunity	General function prediction only	**R**
Anaa_00928	**-**	CDS	-	**S**
Anaa_00929	**-**	CDS	-	**S**
Anaa_00931	**-**	CDS	-	**S**
Anaa_00932	**qorA**	Quinone oxidoreductase 1	Energy production and conversion	**C**
Anaa_00942	**-**	CDS	-	**S**
Anaa_00965	**-**	CDS	-	**S**
Anaa_00968	**-**	RDD family protein	-	**R**
Anaa_00978	**-**	CDS	-	**S**
Anaa_00979	**-**	CDS	-	**S**
Anaa_00982	**rpoC**	DNA-directed RNA polymerase subunit beta'	Purine metabolism	**K**
Anaa_00983	**rpoC**	DNA-directed RNA polymerase subunit beta'	Purine metabolism	**K**
Anaa_00985	**-**	Ankyrin repeats (3 copies)	Ankyrin	**R**
Anaa_00988	**yazA**	GIY-YIG nuclease superfamily protein	Replication, recombination and repair	**L**
Anaa_00994	**-**	CDS	-	**S**
Anaa_00995	**-**	CDS	-	**S**
Anaa_00997	**-**	CDS	-	**S**
Anaa_01000	**-**	putative monovalent cation/H+ antiporter subunit B	Bacterial secretion system	**U**
Anaa_01005	**-**	CDS	-	**S**
Anaa_01008	**-**	CDS	-	**S**
Anaa_01009	**-**	CDS	-	**S**
Anaa_01010	**-**	PD-(D/E)XK nuclease family transposase	Transposition	**L**
Anaa_01013	**-**	CDS	-	**S**
Anaa_01022	**-**	CDS	-	**S**
Anaa_01026	**-**	CDS	-	**S**
Anaa_01031	**ihfA**	Integration host factor subunit alpha	Replication, recombination and repair	**L**
Anaa_01032	**yabA**	DNA replication intiation control protein YabA	Replication	**L**
Anaa_01033	**-**	CDS	-	**S**
Anaa_01035	**-**	CDS	-	**S**
Anaa_01041	**ankX**	Phosphocholine transferase AnkX	Lipopolysaccharide biosynthesis	
Anaa_01042	**-**	CDS	-	**S**
Anaa_01043	**-**	CDS	-	**S**
Anaa_01044	**-**	CDS	-	**S**
Anaa_01079	**-**	CDS	-	**S**
Anaa_01080	**-**	CDS	-	**S**
Anaa_01092	**-**	CDS	-	**S**
Anaa_01105	**nuoD**	NADH-quinone oxidoreductase subunit D	Oxidative phosphorylation	**C**
Anaa_01111	**-**	Integrase core domain protein	General function prediction only	**R**
Anaa_01112	**-**	CDS	-	**S**
Anaa_01116	**-**	Gene 25-like lysozyme	-	**R**
Anaa_01118	**-**	Phage-related baseplate assembly protein	Replication, recombination and repair	**L**
Anaa_01120	**-**	Prophage minor tail protein Z (GPZ)	Replication, recombination and repair	**L**
Anaa_01122	**-**	CDS	-	**S**
Anaa_01129	**-**	Transposase IS116/IS110/IS902 family protein	Transposition	**L**
Anaa_01130	**-**	Transposase	Transposition	**L**
Anaa_01131	**-**	Transposase IS66 family protein	Transposition	**L**
Anaa_01132	**-**	Transposase IS66 family protein	Transposition	**L**
Anaa_01136	**cysS**	Cysteine--tRNA ligase	Aminoacyl-tRNA biosynthesis	**J**
Anaa_01137	**dnaB**	Replicative DNA helicase	DNA replication	**L**
Anaa_01139	**-**	CDS	-	**S**
Anaa_01141	**-**	TrbC/VIRB2 family protein	General function prediction only	**R**
Anaa_01143	**yabA**	DNA replication intiation control protein YabA	Replication	**L**
Anaa_01144	**-**	Transposase IS66 family protein	Transposition	**L**
Anaa_01145	**-**	Transposase IS66 family protein	Transposition	**L**
Anaa_01152	**-**	CDS	-	**S**
Anaa_01155	**-**	PD-(D/E)XK nuclease family transposase	Transposition	**L**
Anaa_01156	**-**	CDS	-	**S**
Anaa_01157	**ltrA**	Group II intron-encoded protein LtrA	Amino acid transport and metabolism	**E**
Anaa_01158	**-**	CDS	-	**S**
Anaa_01160	**-**	CDS	-	**S**
Anaa_01175	**pepQ**	Xaa-Pro dipeptidase	Amino acid transport and metabolism	**E**
Anaa_01184	**-**	CDS	-	**S**
Anaa_01187	**-**	CDS	-	**S**
Anaa_01196	**-**	CDS	-	**S**
Anaa_01197	**argR**	Arginine repressor	Transcription	**K**
Anaa_01198	**artP**	Arginine-binding extracellular protein ArtP precursor	ABC transporters	**E**
Anaa_01199	**artQ**	Arginine transport system permease protein ArtQ	ABC transporters	**E**
Anaa_01200	**artM**	Arginine transport ATP-binding protein ArtM	ABC transporters	**E**
Anaa_01202	**-**	CDS	-	**S**
Anaa_01218	**icd**	isocitrate dehydrogenase	Biosynthesis of amino acids	**C**
Anaa_01219	**-**	pyruvate dehydrogenase subunit beta	Energy production and conversion	**C**
Anaa_01220	**ftsH**	ATP-dependent zinc metalloprotease FtsH	Protein modification	**O**
Anaa_01221	**-**	CDS	-	**S**
Anaa_01222	**-**	CDS	-	**S**
Anaa_01225	**-**	Ankyrin repeats (3 copies)	Ankyrin	**R**
Anaa_01226	**-**	CDS	-	**S**
Anaa_01231	**-**	CDS	-	**S**
Anaa_01242	**prsD**	Type I secretion system ATP-binding protein PrsD	Intracellular trafficking, secretion, and vesicular transport	**U**

Anaa_01244	**-**	CDS	-	**S**
Anaa_01254	**-**	CDS	-	**S**
Anaa_01263	-	CDS	-	**S**
Anaa_01264	**-**	CDS	-	**S**
Anaa_01281	**-**	Transposase	Transposition	**L**
Anaa_01291	-	Acetoin:2,6-dichlorophenolindophenol oxidoreductase	Ketone degradation	**C**
Anaa_01293	**-**	CDS	-	**S**
Anaa_01300	**mgtE**	Magnesium transporter MgtE	Inorganic ion transport and metabolism	**P**
Anaa_01306	-	Transposase	Transposition	**L**
Anaa_01307	**kefC**	Glutathione-regulated potassium-efflux system protein KefC	Posttranslational modification, protein turnover, chaperones	**O**
Anaa_01312	-	FtsH Extracellular	Protein modification	**O**
Anaa_01332	**-**	CDS	-	**S**
Anaa_01333	**-**	CDS	-	**S**
Anaa_01334	**-**	CDS	-	**S**
Anaa_01363	**-**	CDS	-	**S**
Anaa_01364	**-**	CDS	-	**S**
Anaa_01365	-	CDS	-	**S**
Anaa_01367	**-**	CDS	-	**S**
Anaa_01388	**pcrA**	ATP-dependent DNA helicase PcrA	Replication	**L**
Anaa_01392	**-**	CDS	-	**S**
Anaa_01393	**-**	Transposase	Transposition	**L**
Anaa_01415	-	GTP cyclohydrolase	General function prediction only	**R**
Anaa_01434	**mdtL**	Multidrug resistance protein MdtL	Posttranslational modification, protein turnover, chaperones	**O**
Anaa_01438	-	Transposase	Transposition	**L**
Anaa_01439	**-**	CDS	-	**S**
Anaa_01440	**-**	CDS	-	**S**
Anaa_01445	-	CDS	-	**S**
Anaa_01447	**mtaB**	Threonylcarbamoyladenosine tRNA methylthiotransferase MtaB	Translation, ribosomal structure and biogenesis	**J**
Anaa_01451	-	Transposase IS66 family protein	Transposition	**L**
Anaa_01466	**-**	CDS	-	**S**
Anaa_01480	**-**	CDS	-	**S**
Anaa_01484	**-**	CDS	-	**S**
Anaa_01517	**-**	CDS	-	**S**
Anaa_01518	**-**	CDS	-	**S**
Anaa_01523	**-**	CDS	-	**S**
Anaa_01526	**-**	CDS	-	**S**
Anaa_01535	-	CDS	-	**S**
Anaa_01537	**pstC**	phosphate transporter permease subunit PstC	ABC transporters	**P**
Anaa_01538	**-**	CDS	-	**S**
Anaa_01542	**-**	CDS	-	**S**
Anaa_01543	**-**	CDS	-	**S**
Anaa_01558	**-**	CDS	-	**S**
Anaa_01565	**-**	CDS	-	**S**
Anaa_01570	-	PD-(D/E)XK nuclease family transposase	Transposition	**L**
Anaa_01571	**-**	CDS	-	**S**
Anaa_01577	**-**	CDS	-	**S**
Anaa_01579	**-**	CDS	-	**S**
Anaa_01587	**-**	Transposase	Transposition	**L**
Anaa_01589	**-**	CDS	-	**S**
Anaa_01611	**-**	CDS	-	**S**
Anaa_01617	-	CDS	-	**S**
Anaa_01624	-	Transposase	Transposition	**L**
Anaa_01644	**-**	CDS	-	**S**
Anaa_01653	**ispH**	4-hydroxy-3-methylbut-2-enyl diphosphate reductase	Terpenoid backbone biosynthesis	**I**
Anaa_01662	-	Transposase	Transposition	**L**
Anaa_01664	**-**	CDS	-	**S**
Anaa_01670	**-**	CDS	-	**S**
Anaa_01678	**-**	CDS	-	**S**
Anaa_01681	**ddl**	D-alanine--D-alanine ligase	D-Alanine metabolism	**M**
Anaa_01684	**sugE**	Quaternary ammonium compound-resistance protein SugE	General function prediction only	**R**
Anaa_01691	**acnA**	Aconitate hydratase 1	Biosynthesis of amino acids	**C**
Anaa_01701	**-**	CDS	-	**S**
Anaa_01712	-	PD-(D/E)XK nuclease family transposase	Transposition	**L**
Anaa_01714	**exoD**	Exopolysaccharide synthesis, ExoD	General function prediction only	**R**
Anaa_01721	**glyS**	Glycine--tRNA ligase beta subunit	Aminoacyl-tRNA biosynthesis	**J**
Anaa_01738	-	Integrase core domain protein	General function prediction only	**R**
Anaa_01741	**-**	CDS	-	**S**
Anaa_01746	**-**	CDS	-	**S**
Anaa_01749	-	CDS	-	**S**
Anaa_01750	-	Transposase	Transposition	**L**
Anaa_01751	**-**	CDS	-	**S**
Anaa_01752	**-**	CDS	-	**S**
Anaa_01753	**-**	CDS	-	**S**
Anaa_01758	**-**	CDS	-	**S**
Anaa_01763	**gpW**	bacteriophage P2 baseplate assembly protein	Replication, recombination and repair	**L**
Auuu_00027	**-**	CDS	-	**S**
Auuu_00037	**-**	CDS	-	**S**
Auuu_00039	**ltrA**	Group II intron-encoded protein LtrA	Amino acid transport and metabolism	**E**
Auuu_00040	-	PD-(D/E)XK nuclease family transposase	Transposition	**L**
Auuu_00042	**tnpA**	Transposase for transposon Tn5	Transposition	**L**
Auuu_00043	-	CDS	-	**S**
Auuu_00044	-	CDS	-	**S**
Auuu_00056	-	Transposase	Transposition	**L**
Auuu_00110	-	CDS	-	**S**
Auuu_00120	**-**	CDS	-	**S**

Auuu_00121	-	Transposase	Transposition	**L**
Auuu_00129	-	CDS	-	**S**
Auuu_00134	-	CDS	-	**S**
Auuu_00147	-	CDS	-	**S**
Auuu_00204	-	CDS	-	**S**
Auuu_00210	-	CDS	-	**S**
Auuu_00212	-	CDS	-	**S**
Auuu_00246	-	CDS	-	**S**
Auuu_00256	-	anaerobic benzoate catabolism transcriptional regulator	DNA binding	**K**
Auuu_00257	-	Helix-turn-helix	General function prediction only	**R**
Auuu_00258	-	CDS	-	**S**
Auuu_00262	-	CDS	-	**S**
Auuu_00265	-	CDS	-	**S**
Auuu_00283	-	CDS	-	**S**
Auuu_00285	-	CDS	-	**S**
Auuu_00288	-	CDS	-	**S**
Auuu_00292	-	ParB-like nuclease domain protein	General function prediction only	**R**
Auuu_00293	**dpnA**	Modification methylase DpnIIB	Mismatch repair	**L**
Auuu_00296	-	Phage terminase large subunit (GpA)	Replication, recombination and repair	**L**
Auuu_00301	-	CDS	-	**S**
Auuu_00312	-	Phage tail protein (Tail P2 I)	General function prediction only	**R**
Auuu_00313	-	CDS	-	**S**
Auuu_00316	-	CDS	-	**S**
Auuu_00318	**hin**	DNA-invertase hin	Transcription	**K**
Auuu_00321	-	Ankyrin repeats (3 copies)	Ankyrin	**R**
Auuu_00322	-	CDS	-	**S**
Auuu_00324	-	CDS	-	**S**
Auuu_00326	-	CDS	-	**S**
Auuu_00340	-	CDS	-	**S**
Auuu_00353	-	Transposase IS116/IS110/IS902 family protein	Transposition	**L**
Auuu_00354	-	CDS	-	**S**
Auuu_00357	-	CDS	-	**S**
Auuu_00359	-	CDS	-	**S**
Auuu_00360	-	CDS	-	**S**
Auuu_00361	-	CDS	-	**S**
Auuu_00364	-	CDS	-	**S**
Auuu_00374	-	CDS	-	**S**
Auuu_00418	-	CDS	-	**S**
Auuu_00427	-	Ankyrin repeats (3 copies)	Ankyrin	**R**
Auuu_00438	-	CDS	-	**S**
Auuu_00440	**ltrA**	Group II intron-encoded protein LtrA	Amino acid transport and metabolism	**E**
Auuu_00443	-	CDS	-	**S**
Auuu_00447	-	CDS	-	**S**
Auuu_00449	-	CDS	-	**S**
Auuu_00483	-	CDS	-	**S**
Auuu_00492	-	CDS	-	**S**
Auuu_00495	-	CDS	-	**S**
Auuu_00499	-	CDS	-	**S**
Auuu_00507	-	CDS	-	**S**
Auuu_00536	**ltrA**	Group II intron-encoded protein LtrA	Amino acid transport and metabolism	**E**
Auuu_00539	-	Transposase	Transposition	**L**
Auuu_00556	-	PD-(D/E)XK nuclease family transposase	Transposition	**L**
Auuu_00615	-	CDS	-	**S**
Auuu_00618	-	CDS	-	**S**
Auuu_00627	-	Ankyrin repeat protein	Ankyrin	**R**
Auuu_00628	-	Ankyrin repeats (3 copies)	Ankyrin	**R**
Auuu_00629	-	CDS	-	**S**
Auuu_00631	-	Ankyrin repeats (3 copies)	Ankyrin	**R**
Auuu_00636	-	CDS	-	**S**
Auuu_00642	-	Phage tail protein (Tail P2 I)	General function prediction only	**R**
Auuu_00649	-	CDS	-	**S**
Auuu_00657	-	Phage portal protein, lambda family	Replication, recombination and repair	**L**
Auuu_00659	-	CDS	-	**S**
Auuu_00660	-	CDS	-	**S**
Auuu_00663	-	CDS	-	**S**
Auuu_00664	-	CDS	-	**S**
Auuu_00669	-	CDS	-	**S**
Auuu_00741	**appC**	Cytochrome bd-II ubiquinol oxidase subunit 1	Oxidative phosphorylation	**C**
Auuu_00749	-	CDS	-	**S**
Auuu_00750	-	CDS	-	**S**
Auuu_00767	-	CDS	-	**S**
Auuu_00815	-	CDS	-	**S**
Auuu_00826	-	CDS	-	**S**
Auuu_00833	-	CDS	-	**S**
Auuu_00864	-	CDS	-	**S**
Auuu_00865	-	Transposase	Transposition	**L**
Auuu_00875	-	CDS	-	**S**
Auuu_00886	-	CDS	-	**S**
Auuu_00938	-	CDS	-	**S**
Auuu_00964	-	Transposase DDE domain protein	Transposition	**L**
Auuu_00967	-	CDS	-	**S**
Auuu_00968	**tnpA**	Transposase for transposon Tn5	Transposition	**L**
Auuu_00970	-	CDS	-	**S**
Auuu_00972	-	CDS	-	**S**
Auuu_00973	-	CDS	-	**S**
Auuu_01011	-	Transposase	Transposition	**L**
Auuu_01012	-	DDE superfamily endonuclease	General function prediction only	**R**
Auuu_01017	-	CDS	-	**S**

Auuu_01030	**-**	CDS	-	**S**
Auuu_01050	**-**	CDS	-	**S**
Auuu_01058	**-**	Ankyrin repeats (3 copies)	Ankyrin	**R**
Auuu_01090	**-**	CDS	-	**S**
Auuu_01096	**-**	CDS	-	**S**
Auuu_01097	**-**	putative transporter	Carbohydrate transport and metabolism	**G**
Auuu_01098	**-**	CDS	-	**S**
Auuu_01306	**-**	CDS	-	**S**
Cocc_00001	-	CDS	-	**S**
Cocc_00002	**cysS**	Cysteine--tRNA ligase	Aminoacyl-tRNA biosynthesis	**J**
Cocc_00003	**-**	CDS	-	**S**
Cocc_00005	-	CDS	-	**S**
Cocc_00007	**-**	CDS	-	**S**
Cocc_00008	**-**	CDS	-	**S**
Cocc_00009	**gshA**	Glutamate-cysteine ligase	Glutathione biosynthesis	**H**
Cocc_00010	**gshA**	Glutamate-cysteine ligase	Glutathione biosynthesis	**H**
Cocc_00011	**gshA**	Glutamate-cysteine ligase	Glutathione biosynthesis	**H**
Cocc_00012	**nuoN**	NADH-quinone oxidoreductase subunit N	Oxidative phosphorylation	**C**
Cocc_00013	**secY**	preprotein translocase subunit SecY	Bacterial secretion system	**U**
Cocc_00014	**purK**	N5-carboxyaminoimidazole ribonucleotide synthase	Purine metabolism	**F**
Cocc_00015	**-**	CDS	-	**S**
Cocc_00016	**dxr**	1-deoxy-D-xylulose 5-phosphate reductoisomerase	Terpenoid backbone biosynthesis	**I**
Cocc_00017	**ubiH**	2-octaprenyl-6-methoxyphenol hydroxylase	Ubiquinone and other terpenoid-quinone biosynthesis	**HC**
Cocc_00018	**pstB**	Phosphate import ATP-binding protein PstB	ABC transporters	**P**
Cocc_00019	-	CDS	-	**S**
Cocc_00020	**gatA**	Glutamyl-tRNA(Gln) amidotransferase subunit A	Glutamate biosynthesis	**E**
Cocc_00021	**-**	CDS	-	**S**
Cocc_00022	-	CDS	-	**S**
Cocc_00023	**purA**	Adenylosuccinate synthetase	Alanine, aspartate and glutamate metabolism	**F**
Cocc_00025	**atpH**	F0F1 ATP synthase subunit delta	Oxidative phosphorylation	**C**
Cocc_00029	**rplI**	50S ribosomal protein L9	Ribosome	**J**
Cocc_00030	-	CDS	-	**S**
Cocc_00031	**putA**	Bifunctional protein PutA	Glutamate biosynthesis	**J**
Cocc_00032	**gpmI**	2,3-bisphosphoglycerate-independent phosphoglycerate mutase	Glycolysis / Gluconeogenesis	**G**
Cocc_00033	**-**	CDS	-	**S**
Cocc_00034	**guaB**	Inosine-5'-monophosphate dehydrogenase	Purine metabolism	**F**
Cocc_00035	**murD**	UDP-N-acetylmuramoyl-L-alanyl-D-glutamate synthetase	Peptidoglycan biosynthesis	**M**
Cocc_00037	**purD**	Phosphoribosylamine--glycine ligase	Purine metabolism	**F**
Cocc_00038	**pdhS**	Cell-division control histidine kinase PdhS	Energy production and conversion	**C**
Cocc_00039	**pheT**	Phenylalanine--tRNA ligase beta subunit	Aminoacyl-tRNA biosynthesis	**J**
Cocc_00040	**hemA**	5-aminolevulinate synthase	Heme biosynthesis	**H**
Cocc_00043	**mnmE**	tRNA modification GTPase MnmE	Homologous recombination	**J**
Cocc_00046	**-**	CDS	-	**S**
Cocc_00047	**bcp**	Putative peroxiredoxin bcp	Phenylpropanoid biosynthesis	**O**
Cocc_00048	-	CDS	-	**S**
Cocc_00052	**ribD**	Riboflavin biosynthesis protein RibD	Riboflavin biosynthesis	**H**
Cocc_00053	**rsfS**	Ribosomal silencing factor RsfS	Translation, ribosomal structure and biogenesis	**J**
Cocc_00055	**plsX**	Phosphate acyltransferase	Glycerolipid metabolism	**I**
Cocc_00057	**-**	CDS	-	**S**
Cocc_00058	**rnc**	Ribonuclease 3	Transcription	**K**
Cocc_00061	**rplM**	50S ribosomal protein L13	Ribosome	**J**
Cocc_00062	**ftsK**	DNA translocase FtsK	Cell cycle control, cell division, chromosome partitioning	**D**
Cocc_00064	**purD**	Phosphoribosylamine--glycine ligase	Purine metabolism	**F**
Cocc_00065	**serS**	Serine--tRNA ligase	Aminoacyl-tRNA biosynthesis	**J**
Cocc_00066	**sucC**	Succinyl-CoA ligase [ADP-forming] subunit beta	C5-Branched dibasic acid metabolism	**C**
Cocc_00067	**-**	RmuC family protein	General function prediction only	**R**
Cocc_00068	**polA**	DNA polymerase I	Purine metabolism	**L**
Cocc_00069	-	CDS	-	**S**
Cocc_00070	**purB**	Adenylosuccinate lyase	Alanine, aspartate and glutamate metabolism	**F**
Cocc_00071	**hslU**	ATP-dependent protease ATPase subunit HslU	Posttranslational modification, protein turnover, chaperones	**O**
Cocc_00072	**purA**	Adenylosuccinate synthetase	Alanine, aspartate and glutamate metabolism	**F**
Cocc_00073	**pyrG**	CTP synthase	Pyrimidine metabolism	**F**
Cocc_00074	-	CDS	-	**S**
Cocc_00075	**def**	Peptide deformylase	Translation, ribosomal structure and biogenesis	**J**
Cocc_00076	**lepB**	Signal peptidase I	Protein export	**U**
Cocc_00077	**-**	CDS	-	**S**
Cocc_00080	**ccmF**	Cytochrome c-type biogenesis protein CcmF	Oxidative phosphorylation	**O**
Cocc_00081	**-**	phosphatidylserine decarboxylase	Lipid transport and metabolism	**I**
Cocc_00082	**-**	CDS	-	**S**
Cocc_00083	**cspA**	Cold shock protein CspA	General function prediction only	**R**
Cocc_00085	**-**	CDS	-	**S**
Cocc_00086	**nuoB**	NADH-quinone oxidoreductase subunit B	Oxidative phosphorylation	**C**
Cocc_00089	-	CDS	-	**S**
Cocc_00090	**dxr**	1-deoxy-D-xylulose 5-phosphate reductoisomerase	Terpenoid backbone biosynthesis	**I**
Cocc_00091	-	TrbL/VirB6 plasmid conjugal transfer protein	General function prediction only	**R**
Cocc_00092	**map**	Methionine aminopeptidase	Translation, ribosomal structure and biogenesis	**J**
Cocc_00095	**iscS**	Cysteine desulfurase	Alanine biosynthesis	**E**
Cocc_00096	-	CDS	-	**S**
Cocc_00097	**nuoN**	NADH-quinone oxidoreductase subunit N	Oxidative phosphorylation	**C**

Cocc_00101	**-**	CDS	-	**S**
Cocc_00102	**plsY**	putative glycerol-3-phosphate acyltransferase	Glycerolipid metabolism	**I**
Cocc_00104	**fmt**	Methionyl-tRNA formyltransferase	Aminoacyl-tRNA biosynthesis	**J**
Cocc_00105	**virB4**	Type IV secretion system protein virB4	Bacterial secretion system	**U**
Cocc_00107	**guaA**	GMP synthase [glutamine-hydrolyzing]	Purine metabolism	**F**
Cocc_00108	**ywlF**	Putative sugar phosphate isomerase YwlF	Pentose and glucuronate interconversions	**G**
Cocc_00109	**birA**	Bifunctional ligase/repressor BirA	Biotin metabolism	**H**
Cocc_00110	**rplY**	50S ribosomal protein L25	Ribosome	**J**
Cocc_00111	**adk**	adenylate kinase	Purine metabolism	**F**
Cocc_00112	**rpsM**	30S ribosomal protein S13	Ribosome	**J**
Cocc_00113	**-**	CDS	-	**S**
Cocc_00114	**mlaE**	putative phospholipid ABC transporter permease protein MlaE	ABC transporters	**O**
Cocc_00115	**-**	CDS	-	**S**
Cocc_00116	**cysS**	Cysteine--tRNA ligase	Aminoacyl-tRNA biosynthesis	**J**
Cocc_00119	**pyrC**	Dihydroorotase	Pyrimidine metabolism	**F**
Cocc_00121	**-**	Holliday junction resolvase-like protein	Replication, recombination and repair	**L**
Cocc_00122	**infA**	Translation initiation factor IF-1	Translation, ribosomal structure and biogenesis	**J**
Cocc_00123	**-**	CDS	-	**S**
Cocc_00125	**-**	Calcineurin-like phosphoesterase	General function prediction only	**R**
Cocc_00126	**mutS**	DNA mismatch repair protein MutS	Mismatch repair	**L**
Cocc_00127	**mnmG**	tRNA uridine 5-carboxymethylaminomethyl modification enzyme MnmG	Translation, ribosomal structure and biogenesis	**J**
Cocc_00129	**-**	CDS	-	**S**
Cocc_00130	**rplC**	50S ribosomal protein L3	Ribosome	**J**
Cocc_00132	**ppa**	Inorganic pyrophosphatase	Oxidative phosphorylation	**C**
Cocc_00136	**-**	CDS	-	**S**
Cocc_00137	**-**	phosphoribosylformylglycinamidine synthase II	Purine metabolism	**R**
Cocc_00138	**accC**	Biotin carboxylase	Fatty acid biosynthesis	**I**
Cocc_00139	**rplL**	50S ribosomal protein L7/L12	Ribosome	**J**
Cocc_00140	**nuoF**	NADH-quinone oxidoreductase subunit F	Oxidative phosphorylation	**C**
Cocc_00141	**purC**	Phosphoribosylaminoimidazole-succinocarboxamide synthase	Purine metabolism	**F**
Cocc_00143	**mutL**	DNA mismatch repair protein MutL	Mismatch repair	**L**
Cocc_00144	**hslU**	ATP-dependent protease ATPase subunit HslU	Posttranslational modification, protein turnover, chaperones	**O**
Cocc_00145	**proP**	proline/glycine betaine transporter	Mulitple COGs	**GEP**
Cocc_00146	**-**	NADH-quinone oxidoreductase subunit 12	General function prediction only	**R**
Cocc_00147	**clpB**	Chaperone protein ClpB	Chaperone	**O**
Cocc_00148	**nuoH**	NADH-quinone oxidoreductase subunit H	Oxidative phosphorylation	**C**
Cocc_00150	**iscS**	Cysteine desulfurase	Alanine biosynthesis	**E**
Cocc_00151	**xerD**	Tyrosine recombinase XerD	Recombination	**L**
Cocc_00156	**-**	putative monovalent cation/H+ antiporter subunit B	Bacterial secretion system	**U**
Cocc_00157	**secA**	preprotein translocase subunit SecA	Bacterial secretion system	**MU**
Cocc_00158	**ubiA**	4-hydroxybenzoate octaprenyltransferase	Ubiquinone and other terpenoid-quinone biosynthesis	**H**
Cocc_00163	**traG**	Conjugal transfer protein TraG	Replication, recombination and repair	**L**
Cocc_00164	**ppdK**	Pyruvate, phosphate dikinase	Carbon metabolism	**C**
Cocc_00165	**secD**	preprotein translocase subunit SecD	Bacterial secretion system	**U**
Cocc_00167	**rplX**	50S ribosomal protein L24	Ribosome	**J**
Cocc_00169	**ctaD**	Cytochrome c oxidase subunit 1	Oxidative phosphorylation	**C**
Cocc_00170	**ctaC**	Cytochrome c oxidase subunit 2 precursor	Oxidative phosphorylation	**C**
Cocc_00171	**dnaE1**	DNA polymerase III subunit alpha	Base excision repair	**L**
Cocc_00172	**rpoB**	DNA-directed RNA polymerase subunit beta	Purine metabolism	**K**
Cocc_00173	**metG**	Methionine--tRNA ligase	Selenocompound metabolism	**J**
Cocc_00175	**truA**	tRNA pseudouridine synthase A	Translation, ribosomal structure and biogenesis	**J**
Cocc_00176	**-**	Type IV secretory pathway, VirB3-like protein	Defense mechanisms	**V**
Cocc_00177	**modB**	molybdate ABC transporter permease protein	Inorganic ion transport and metabolism	**P**
Cocc_00178	**mutL**	DNA mismatch repair protein MutL	Mismatch repair	**L**
Cocc_00179	**rpoC**	DNA-directed RNA polymerase subunit beta'	Purine metabolism	**K**
Cocc_00181	**trpS**	Tryptophan--tRNA ligase	Aminoacyl-tRNA biosynthesis	**J**
Cocc_00182	**miaB**	(Dimethylallyl)adenosine tRNA methylthiotransferase MiaB	Puromycin biosynthesis	**J**
Cocc_00183	**-**	Phage portal protein	Replication, recombination and repair	**L**
Cocc_00184	**gpmI**	2,3-bisphosphoglycerate-independent phosphoglycerate mutase	Glycolysis / Gluconeogenesis	**G**
Cocc_00185	**-**	CDS	-	**S**
Cocc_00186	**putA**	Bifunctional protein PutA	Glutamate biosynthesis	**J**
Cocc_00187	**lpd**	Dihydrolipoyl dehydrogenase	Carbon metabolism	**C**
Cocc_00189	**ankX**	Phosphocholine transferase AnkX	Lipopolysaccharide biosynthesis	
Cocc_00191	**nuoD**	NADH-quinone oxidoreductase subunit D	Oxidative phosphorylation	**C**
Cocc_00192	**accA1**	Acetyl-/propionyl-coenzyme A carboxylase alpha chain	Fatty acid biosynthesis	**I**
Cocc_00194	**gdh**	NAD-specific glutamate dehydrogenase	Arginine biosynthesis	**G**
Cocc_00195	**murA**	UDP-N-acetylglucosamine 1-carboxyvinyltransferase	Peptidoglycan biosynthesis	**M**
Cocc_00196	**znuB**	High-affinity zinc uptake system membrane protein ZnuB	ABC transporters	**P**
Cocc_00197	**ispU**	Ditrans,polycis-undecaprenyl-diphosphate synthase ((2E,6E)-farnesyl-diphosphate specific)	Terpenoid backbone biosynthesis	**I**
Cocc_00199	**-**	Nitronate monooxygenase	General function prediction only	**R**
Cocc_00201	**-**	CDS	-	**S**
Cocc_00203	**-**	CDS	-	**S**
Cocc_00204	**purF**	Amidophosphoribosyltransferase precursor	Alanine, aspartate and glutamate metabolism	**F**
Cocc_00206	**-**	CDS	-	**S**
Cocc_00213	**rpoD**	RNA polymerase sigma factor RpoD	Transcription	**K**
Cocc_00214	**gdh**	NAD-specific glutamate dehydrogenase	Arginine biosynthesis	**G**
Cocc_00217	**gltP**	Proton glutamate symport protein	Energy production and conversion	**C**
Cocc_00218	**tuf**	elongation factor Tu	Translation	**J**
Cocc_00219	**-**	CDS	-	**S**
Cocc_00221	**nqo3**	NADH-quinone oxidoreductase chain 3	Carbohydrate transport and metabolism	**G**

Cocc_00223	**pld**	Phospholipase D precursor	CAMP signaling pathway	**I**
Cocc_00224	**rpoC**	DNA-directed RNA polymerase subunit beta'	Purine metabolism	**K**
Cocc_00226	-	Surface antigen	Defense	**R**
Cocc_00228	**petB**	Cytochrome b	Oxidative phosphorylation	**C**
Cocc_00229	-	Phage portal protein	Replication, recombination and repair	**L**
Cocc_00230	**pnp**	Polyribonucleotide nucleotidyltransferase	Purine metabolism	**F**
Cocc_00231	**accD5**	putative propionyl-CoA carboxylase beta chain 5	Fatty acid biosynthesis	**I**
Cocc_00233	-	CDS	-	**S**
Cocc_00234	**dapB**	4-hydroxy-tetrahydrodipicolinate reductase	Lysine biosynthesis	**E**
Cocc_00236	-	CDS	-	**S**
Cocc_00237	**uvrD**	DNA helicase II	Mismatch repair	**L**
Cocc_00238	**obg**	GTPase Obg	General function prediction only	**R**
Cocc_00239	-	CDS	-	**S**
Cocc_00240	-	CDS	-	**S**
Cocc_00241	-	CDS	-	**S**
Cocc_00242	-	CDS	-	**S**
Cocc_00243	-	CDS	-	**S**
Cocc_00245	**ddl**	D-alanine--D-alanine ligase	D-Alanine metabolism	**M**
Cocc_00246	**kefC**	Glutathione-regulated potassium-efflux system protein KefC	Posttranslational modification, protein turnover, chaperones	**O**
Cocc_00248	-	CDS	-	**S**
Cocc_00249	**addA**	ATP-dependent helicase/nuclease subunit A	Base excision repair	**L**
Cocc_00252	**sdhA**	Succinate dehydrogenase flavoprotein subunit	Butanoate metabolism	**C**
Cocc_00253	**secA**	preprotein translocase subunit SecA	Bacterial secretion system	**MU**
Cocc_00255	-	CDS	-	**S**
Cocc_00256	**mdh**	Malate dehydrogenase	Carbon metabolism	**C**
Cocc_00257	**engB**	putative GTP-binding protein EngB	Cell cycle control, cell division, chromosome partitioning	**D**
Cocc_00259	-	CDS	-	**S**
Cocc_00260	**alsT**	Amino-acid carrier protein AlsT	Amino acid transport	**E**
Cocc_00262	**carB**	Carbamoyl-phosphate synthase large chain	Arginine biosynthesis	**EF**
Cocc_00263	**mreB**	Rod shape-determining protein MreB	Cell cycle control, cell division, chromosome partitioning	**D**
Cocc_00264	**uvrA**	UvrABC system protein A	Nucleotide excision repair	**L**
Cocc_00267	-	CDS	-	**S**
Cocc_00268	**bioY**	Biotin transporter BioY	Cofactor transport	**H**
Cocc_00271	-	CDS	-	**S**
Cocc_00272	**ssb**	Single-stranded DNA-binding protein	DNA replication	**L**
Cocc_00273	**ispH**	4-hydroxy-3-methylbut-2-enyl diphosphate reductase	Terpenoid backbone biosynthesis	**I**
Cocc_00274	**hflK**	Modulator of FtsH protease HflK	Protein modification	**O**
Cocc_00275	**ileS**	isoleucyl-tRNA synthetase	Aminoacyl-tRNA biosynthesis	**J**
Cocc_00276	-	CDS	-	**S**
Cocc_00277	-	CDS	-	**S**
Cocc_00278	**glyA**	Serine hydroxymethyltransferase	Glycine biosynthesis	**HE**
Cocc_00279	**ubiE**	Ubiquinone/menaquinone biosynthesis C-methyltransferase UbiE	Ubiquinone and other terpenoid-quinone biosynthesis	**H**
Cocc_00284	**virB9**	Type IV secretion system protein virB9 precursor	Bacterial secretion system	**U**
Cocc_00286	**ftsY**	Signal recognition particle receptor FtsY	Bacterial secretion system	**U**
Cocc_00290	**fbpC**	Fe(3+) ions import ATP-binding protein FbpC	ABC transporters	**P**
Cocc_00291	**ubiF**	2-octaprenyl-3-methyl-6-methoxy-1,4-benzoquinol hydroxylase	Coenzyme transport and metabolism	**H**
Cocc_00297	**hflC**	Modulator of FtsH protease HflC	Protein modification	**O**
Cocc_00298	**hflK**	Modulator of FtsH protease HflK	Protein modification	**O**
Cocc_00300	**hemF**	Coproporphyrinogen-III oxidase, aerobic	Porphyrin metabolism	**H**
Cocc_00302	**parA**	Chromosome partitioning protein ParA	Replication, recombination and repair	**L**
Cocc_00303	**fumC**	Fumarate hydratase class II	Carbon metabolism	**C**
Cocc_00304	**putA**	Bifunctional protein PutA	Glutamate biosynthesis	**J**
Cocc_00305	**proS**	Proline--tRNA ligase	Aminoacyl-tRNA biosynthesis	**J**
Cocc_00306	**acpS**	Holo-[acyl-carrier-protein] synthase	Pantothenate and CoA biosynthesis	**I**
Cocc_00307	**rpoA**	DNA-directed RNA polymerase subunit alpha	Purine metabolism	**K**
Cocc_00313	-	CDS	-	**S**
Cocc_00315	**accA1**	Acetyl-/propionyl-coenzyme A carboxylase alpha chain	Fatty acid biosynthesis	**I**
Cocc_00316	**arcD**	Arginine/ornithine antiporter	Amino acid transport	**E**
Cocc_00317	-	CDS	-	**S**
Cocc_00318	**fdxA**	Ferredoxin-2	Inorganic ion transport and metabolism	**P**
Cocc_00319	**purA**	Adenylosuccinate synthetase	Alanine, aspartate and glutamate metabolism	**F**
Cocc_00321	**dnaJ**	chaperone protein DnaJ	Chaperone	**O**
Cocc_00322	-	CDS	-	**S**
Cocc_00324	**lpdG**	Dihydrolipoamide dehydrogenase	Energy production and conversion	**C**
Cocc_00325	-	Alpha/beta hydrolase family protein	-	**R**
Cocc_00326	**suhB**	Inositol-1-monophosphatase	Streptomycin biosynthesis	**G**
Cocc_00327	**uvrA**	UvrABC system protein A	Nucleotide excision repair	**L**
Cocc_00329	**gap**	Glyceraldehyde-3-phosphate dehydrogenase	Biosynthesis of amino acids	**E**
Cocc_00330	**hemF**	Coproporphyrinogen-III oxidase, aerobic	Porphyrin metabolism	**H**
Cocc_00332	-	CDS	-	**S**
Cocc_00334	**gyrA**	DNA gyrase subunit A	Replication, recombination and repair	**L**
Cocc_00336	**carA**	Carbamoyl-phosphate synthase small chain	Arginine biosynthesis	**EF**
Cocc_00338	**pcaH**	Protocatechuate 3,4-dioxygenase beta chain	Secondary metabolites biosynthesis, transport and catabolism	**Q**
Cocc_00339	**accD5**	putative propionyl-CoA carboxylase beta chain 5	Fatty acid biosynthesis	**I**
Cocc_00341	**nqo2**	NADH-quinone oxidoreductase chain 2	Carbohydrate transport and metabolism	**G**
Cocc_00342	**maeB**	NADP-dependent malic enzyme	Carbon metabolism	**C**
Cocc_00343	**pmbA**	peptidase PmbA	General function prediction only	**R**
Cocc_00348	**lipA**	Lipoyl synthase	Lipoate biosynthesis	**H**
Cocc_00349	**hemH**	Ferrochelatase	Heme biosynthesis	**H**
Cocc_00350	-	CDS	-	**S**
Cocc_00354	**rpoD**	RNA polymerase sigma factor RpoD	Transcription	**K**
Cocc_00355	-	CDS	-	**S**

Cocc_00356	**-**	CDS	-	**S**
Cocc_00357	**rplQ**	50S ribosomal protein L17	Ribosome	**J**
Cocc_00364	**-**	CDS	-	**S**
Cocc_00367	**-**	CDS	-	**S**
Cocc_00368	**pyrG**	CTP synthase	Pyrimidine metabolism	**F**
Cocc_00369	**polA**	DNA polymerase I, thermostable	Purine metabolism	**L**
Cocc_00371	**fabI**	Enoyl-[acyl-carrier-protein] reductase [NADH] FabI	Fatty acid biosynthesis	**I**
Cocc_00372	**-**	Membrane transport protein	General function prediction only	**R**
Cocc_00376	**ctaD**	Cytochrome c oxidase subunit 1	Oxidative phosphorylation	**C**
Cocc_00377	**sdhA**	Succinate dehydrogenase flavoprotein subunit	Butanoate metabolism	**C**
Cocc_00378	**gmk**	Guanylate kinase	Purine metabolism	**F**
Cocc_00379	**ypwA**	Carboxypeptidase Taq (M32) metallopeptidase	General function prediction only	**R**
Cocc_00381	**glyA1**	Serine hydroxymethyltransferase 1	Mulitple COGs	**HE**
Cocc_00382	**-**	CDS	-	**S**
Cocc_00384	**hemB**	Delta-aminolevulinic acid dehydratase	Heme biosynthesis	**H**
Cocc_00385	**kgd**	Multifunctional 2-oxoglutarate metabolism enzyme	Energy production and conversion	**C**
Cocc_00387	**ctaE**	Cytochrome c oxidase subunit 3	Oxidative phosphorylation	**C**
Cocc_00393	**nrdZ**	Ribonucleoside-diphosphate reductase NrdZ	Nucleotide transport and metabolism	**F**
Cocc_00396	**murB**	UDP-N-acetylenolpyruvoylglucosamine reductase	Peptidoglycan biosynthesis	**M**
Cocc_00398	**ligA**	DNA ligase	Base excision repair	**U**
Cocc_00399	**uvrC**	UvrABC system protein C	Nucleotide excision repair	**L**
Cocc_00401	**rplD**	50S ribosomal protein L4	Ribosome	**J**
Cocc_00402	**-**	CDS	-	**S**
Cocc_00404	**erpA**	Iron-sulfur cluster insertion protein ErpA	Inorganic ion transport and metabolism	**P**
Cocc_00406	**lepA**	Elongation factor 4	Cell wall/membrane/envelope biogenesis	**M**
Cocc_00407	**uvrA**	UvrABC system protein A	Nucleotide excision repair	**L**
Cocc_00408	**ribF**	Riboflavin biosynthesis protein RibF	Riboflavin biosynthesis	**H**
Cocc_00409	**atpA**	ATP synthase subunit alpha	Oxidative phosphorylation	**C**
Cocc_00410	**-**	CDS	-	**S**
Cocc_00412	**lipA**	Lipoyl synthase	Lipoate biosynthesis	**H**
Cocc_00413	**-**	CDS	-	**S**
Cocc_00414	**dnaG**	DNA primase	DNA replication	**L**
Cocc_00416	**-**	CDS	-	**S**
Cocc_00419	**-**	CDS	-	**S**
Cocc_00420	**-**	CDS	-	**S**
Cocc_00425	**tatA**	Sec-independent protein translocase protein TatA	Bacterial secretion system	**U**
Cocc_00426	**-**	CDS	-	**S**
Cocc_00427	**-**	CDS	-	**S**
Cocc_00428	**comM**	Competence protein ComM	Replication, recombination and repair	**L**
Cocc_00429	**carB**	Carbamoyl-phosphate synthase large chain	Arginine biosynthesis	**EF**
Cocc_00432	**nrdZ**	Ribonucleoside-diphosphate reductase NrdZ	Nucleotide transport and metabolism	**F**
Cocc_00434	**-**	CDS	-	**S**
Cocc_00436	**holA**	DNA polymerase III subunit delta	Purine metabolism	**F**
Cocc_00437	**dapE**	Succinyl-diaminopimelate desuccinylase	Lysine biosynthesis	**E**
Cocc_00440	**rpoB**	DNA-directed RNA polymerase subunit beta	Purine metabolism	**K**
Cocc_00441	**polA**	DNA polymerase I	Purine metabolism	**L**
Cocc_00442	**-**	CDS	-	**S**
Cocc_00443	**polA**	DNA polymerase I, thermostable	Purine metabolism	**L**
Cocc_00445	**fabF**	3-oxoacyl-[acyl-carrier-protein] synthase 2	Fatty acid biosynthesis	**I**
Cocc_00446	**alaS**	Alanine--tRNA ligase	Aminoacyl-tRNA biosynthesis	**J**
Cocc_00447	**prfA**	Peptide chain release factor 1	Translation, ribosomal structure and biogenesis	**J**
Cocc_00448	**-**	putative monovalent cation/H+ antiporter subunit B	Bacterial secretion system	**U**
Cocc_00449	**leuS**	Leucine--tRNA ligase	Aminoacyl-tRNA biosynthesis	**J**
Cocc_00454	**rlmE**	Ribosomal RNA large subunit methyltransferase E	Translation, ribosomal structure and biogenesis	**J**
Cocc_00456	**ctrA**	Cell cycle response regulator CtrA	Cell cycle control	**D**
Cocc_00457	**rbfA**	Ribosome-binding factor A	Translation, ribosomal structure and biogenesis	**J**
Cocc_00458	**ubiA**	4-hydroxybenzoate octaprenyltransferase	Ubiquinone and other terpenoid-quinone biosynthesis	**H**
Cocc_00461	**rnj1**	Ribonuclease J 1	Replication, recombination and repair	**L**
Cocc_00462	**uvrA**	UvrABC system protein A	Nucleotide excision repair	**L**
Cocc_00463	**ptsA**	phosphate transporter permease subunit PtsA	Carbohydrate transport and metabolism	**G**
Cocc_00464	**-**	CDS	-	**S**
Cocc_00467	**-**	CDS	-	**S**
Cocc_00468	**ccmA**	Cytochrome c biogenesis ATP-binding export protein CcmA	ABC transporters	**V**
Cocc_00469	**atpA**	ATP synthase subunit alpha	Oxidative phosphorylation	**C**
Cocc_00473	**pleD**	Response regulator PleD	Signal transduction mechanisms	**T**
Cocc_00474	**secA**	preprotein translocase subunit SecA	Bacterial secretion system	**MU**
Cocc_00477	**cysS**	Cysteine--tRNA ligase	Aminoacyl-tRNA biosynthesis	**J**
Cocc_00480	**nuoL**	NADH-quinone oxidoreductase subunit L	Oxidative phosphorylation	**CP**
Cocc_00481	**purF**	Amidophosphoribosyltransferase precursor	Alanine, aspartate and glutamate metabolism	**F**
Cocc_00482	**-**	CDS	-	**S**
Cocc_00483	**prs**	Ribose-phosphate pyrophosphokinase	Pentose phosphate pathway	**F**
Cocc_00484	**rnd**	ribonuclease D	Translation, ribosomal structure and biogenesis	**J**
Cocc_00485	**pyrC**	Dihydroorotase	Pyrimidine metabolism	**F**
Cocc_00489	**sucA**	2-oxoglutarate dehydrogenase E1 component	Carbon metabolism	**C**
Cocc_00492	**-**	Blue-light-activated protein	General function prediction only	**R**
Cocc_00494	**mutL**	DNA mismatch repair protein MutL	Mismatch repair	**L**
Cocc_00495	**-**	Malonyl-CoA decarboxylase (MCD)	General function prediction only	**R**
Cocc_00500	**valS**	Valine--tRNA ligase	Aminoacyl-tRNA biosynthesis	**J**
Cocc_00502	**coq7**	2-nonaprenyl-3-methyl-6-methoxy-1,4-benzoquinol hydroxylase	Aminobenzoate degradation	
Cocc_00506	**uvrC**	UvrABC system protein C	Nucleotide excision repair	**L**
Cocc_00513	**qorA**	Quinone oxidoreductase 1	Energy production and conversion	**C**

Cocc_00516	**gpmI**	2,3-bisphosphoglycerate-independent phosphoglycerate mutase	Glycolysis / Gluconeogenesis	**G**
Cocc_00517	**surE**	5'-nucleotidase SurE	Nicotinate and nicotinamide metabolism	**E**
Cocc_00519	**-**	CDS	-	**S**
Cocc_00520	**pyrH**	Uridylate kinase	Pyrimidine metabolism	**F**
Cocc_00521	**frr**	Ribosome-recycling factor	Translation, ribosomal structure and biogenesis	**J**
Cocc_00523	**-**	CDS	-	**S**
Cocc_00525	**mgtE**	Magnesium transporter MgtE	Inorganic ion transport and metabolism	**P**
Cocc_00527	**recJ**	Single-stranded-DNA-specific exonuclease RecJ	Base excision repair	**L**
Cocc_00528	**-**	Transposase, Mutator family	Transposition	**L**
Cocc_00531	-	CDS	-	**S**
Cocc_00536	**uvrA**	UvrABC system protein A	Nucleotide excision repair	**L**
Cocc_00539	**glyQ**	Glycine--tRNA ligase alpha subunit	Aminoacyl-tRNA biosynthesis	**J**
Cocc_00541	**pheS**	Phenylalanine--tRNA ligase alpha subunit	Aminoacyl-tRNA biosynthesis	**J**
Cocc_00542	**dnaJ**	chaperone protein DnaJ	Chaperone	**O**
Cocc_00543	**rnj1**	Ribonuclease J 1	Replication, recombination and repair	**L**
Cocc_00545	**yhjE**	Inner membrane metabolite transport protein YhjE	Intracellular trafficking, secretion, and vesicular transport	**U**
Cocc_00546	**ftsH**	ATP-dependent zinc metalloprotease FtsH	Protein modification	**O**
Cocc_00549	**-**	CDS	-	**S**
Cocc_00550	**-**	Holliday junction resolvase-like protein	Replication, recombination and repair	**L**
Cocc_00553	**gltA**	Citrate synthase	Biosynthesis of amino acids	**C**
Cocc_00556	**priA**	Primosomal protein N'	Homologous recombination	**L**
Cocc_00559	**ihfA**	Integration host factor subunit alpha	Replication, recombination and repair	**L**
Cocc_00564	**rplE**	50S ribosomal protein L5	Ribosome	**J**
Cocc_00569	**-**	CDS	-	**S**
Cocc_00570	**pipB2**	Secreted effector protein pipB2	Secondary metabolites biosynthesis, transport and catabolism	**Q**
Cocc_00571	**acn**	Aconitate hydratase	Biosynthesis of amino acids	**C**
Cocc_00574	**-**	RNA 2'-O ribose methyltransferase substrate binding protein	Translation, ribosomal structure and biogenesis	**J**
Cocc_00577	**def**	Peptide deformylase	Protein modification	**J**
Cocc_00580	**murG**	UDP-N-acetylglucosamine--N-acetylmuramyl-(pentapeptide) pyrophosphoryl-undecaprenol N-acetylglucosamine transferase	Peptidoglycan biosynthesis	**M**
Cocc_00586	**algC**	Phosphomannomutase/phosphoglucomutase	Glycolysis / Gluconeogenesis	**G**
Cocc_00588	**-**	CDS	-	**S**
Cocc_00589	**clpA**	ATP-dependent Clp protease ATP-binding subunit ClpA	Misfolded protein degradation	**O**
Cocc_00595	**-**	CDS	-	**S**
Cocc_00596	**atpG**	ATP synthase gamma chain	Oxidative phosphorylation	**C**
Cocc_00600	**pyrDA**	Dihydroorotate dehydrogenase A (fumarate)	Pyrimidine metabolism	**F**
Cocc_00604	**atpD**	ATP synthase subunit beta	Oxidative phosphorylation	**C**
Cocc_00605	**pstA**	Phosphate transport system permease protein PstA	ABC transporters	**P**
Cocc_00606	**rpoC**	DNA-directed RNA polymerase subunit beta'	Purine metabolism	**K**
Cocc_00607	**rsmA**	Ribosomal RNA small subunit methyltransferase A	Translation, ribosomal structure and biogenesis	**J**
Cocc_00610	-	CDS	-	**S**
Cocc_00611	**metK**	S-adenosylmethionine synthase	Biosynthesis of amino acids	**H**
Cocc_00613	**carB**	Carbamoyl-phosphate synthase large chain	Arginine biosynthesis	**EF**
Cocc_00614	**-**	CDS	-	**S**
Cocc_00618	**rpsG**	30S ribosomal protein S7	Ribosome	**J**
Cocc_00622	**nuoF**	NADH-quinone oxidoreductase subunit F	Oxidative phosphorylation	**C**
Cocc_00625	**rbfA**	Ribosome-binding factor A	Translation, ribosomal structure and biogenesis	**J**
Cocc_00626	**infB**	Translation initiation factor IF-2	Translation, ribosomal structure and biogenesis	**J**
Cocc_00636	**-**	CDS	-	**S**
Cocc_00638	**-**	CDS	-	**S**
Cocc_00640	**-**	CDS	-	**S**
Cocc_00643	**-**	CDS	-	**S**
Cocc_00644	**ispB**	Octaprenyl-diphosphate synthase	Terpenoid backbone biosynthesis	**H**
Cocc_00650	**ctaD**	Cytochrome c oxidase subunit 1	Oxidative phosphorylation	**C**
Cocc_00651	**maeB**	NADP-dependent malic enzyme	Carbon metabolism	**C**
Cocc_00652	**pgsA**	CDP-diacylglycerol--glycerol-3-phosphate 3-phosphatidyltransferase	Glycerophospholipid metabolism	**I**
Cocc_00654	**rplB**	50S ribosomal protein L2	Ribosome	**J**
Cocc_00657	**-**	CDS	-	**S**
Cocc_00658	**xerD**	Tyrosine recombinase XerD	Recombination	**L**
Cocc_00661	**dgt**	deoxyguanosinetriphosphate triphosphohydrolase-like protein	Purine metabolism	**F**
Cocc_00669	**petA**	Ubiquinol-cytochrome c reductase iron-sulfur subunit	Oxidative phosphorylation	
Cocc_00670	**gyrA**	DNA gyrase subunit A	Replication, recombination and repair	**L**
Cocc_00674	**rpsA**	30S ribosomal protein S1	Ribosome	**C**
Cocc_00675	**-**	CDS	-	**S**
Cocc_00677	**nuoI**	NADH-quinone oxidoreductase subunit I	Oxidative phosphorylation	**C**
Cocc_00680	**-**	CDS	-	**S**
Cocc_00682	**nuoF**	NADH-quinone oxidoreductase subunit F	Oxidative phosphorylation	**C**
Cocc_00684	**rpsB**	30S ribosomal protein S2	Ribosome	**J**
Cocc_00691	**ubiB**	putative protein kinase UbiB	Glycosylphosphatidylinositol(GPI)-anchor biosynthesis	**HC**
Cocc_00692	**-**	CDS	-	**S**
Cocc_00698	**proP**	Proline/betaine transporter	Transporter/osmoregulator	**GEP**
Cocc_00699	**rsmA**	Ribosomal RNA small subunit methyltransferase A	Translation, ribosomal structure and biogenesis	**J**
Cocc_00700	**dnaX**	DNA polymerase III subunit tau	Purine metabolism	**L**
Cocc_00701	**-**	CDS	-	**S**
Cocc_00702	**estB**	Carboxylesterase 2	Drug metabolism - other enzymes	**V**
Cocc_00703	**virB9**	Type IV secretion system protein virB9 precursor	Bacterial secretion system	**U**

Cocc_00707	**rlmB**	23S rRNA (guanosine-2'-O-)-methyltransferase	Translation, ribosomal structure and biogenesis	**J**
Cocc_00717	**-**	peptidylprolyl isomerase	General function prediction only	**R**
Cocc_00719	**-**	CDS	-	**S**
Cocc_00722	**-**	CDS	-	**S**
Cocc_00730	**-**	CDS	-	**S**
Cocc_00732	**gyrB**	DNA gyrase subunit B	Replication, recombination and repair	**L**
Cocc_00737	**nusG**	transcription antitermination protein NusG	Transcription	**K**
Cocc_00739	**tuf**	elongation factor Tu	Translation	**J**
Cocc_00748	**pstS**	Phosphate-binding protein PstS precursor	ABC transporters	**P**
Cocc_00749	**-**	CDS	-	**S**
Cocc_00752	**alaS**	Alanine--tRNA ligase	Aminoacyl-tRNA biosynthesis	**J**
Cocc_00753	**miaA**	tRNA dimethylallyltransferase	Zeatin biosynthesis	**J**
Cocc_00756	**bcr**	Bicyclomycin resistance protein	Amino acid transport and metabolism	**E**
Cocc_00757	**accD5**	putative propionyl-CoA carboxylase beta chain 5	Fatty acid biosynthesis	**I**
Cocc_00759	**murF**	UDP-N-acetylmuramoyl-tripeptide--D-alanyl-D-alanine ligase	Lysine biosynthesis	**M**
Cocc_00764	**-**	CDS	-	**S**
Cocc_00772	**-**	Bacterial conjugation TrbI-like protein	General function prediction only	**R**
Cocc_00774	**coaD**	Phosphopantetheine adenylyltransferase	Pantothenate and CoA biosynthesis	**H**
Cocc_00775	**uvrA**	UvrABC system protein A	Nucleotide excision repair	**L**
Cocc_00784	**-**	CDS	-	**S**
Cocc_00786	**-**	CDS	-	**S**
Cocc_00792	**-**	putative monovalent cation/H+ antiporter subunit B	Bacterial secretion system	**U**
Cocc_00794	**-**	CDS	-	**S**
Cocc_00795	**-**	CDS	-	**S**
Cocc_00798	**xerC**	site-specific tyrosine recombinase XerC	Recombination	**L**
Cocc_00807	**purD**	Phosphoribosylamine--glycine ligase	Purine metabolism	**F**
Cocc_00812	**-**	CDS	-	**S**
Cocc_00813	**-**	CDS	-	**S**
Cocc_00815	**gap**	Glyceraldehyde-3-phosphate dehydrogenase	Biosynthesis of amino acids	**E**
Cocc_00825	**-**	CDS	-	**S**
Cocc_00830	**rnhB**	Ribonuclease HII	DNA replication	**L**
Cocc_00838	**rpmI**	50S ribosomal protein L35	Ribosome	**J**
Cocc_00839	**rplA**	50S ribosomal protein L1	Ribosome	**J**
Cocc_00841	**-**	CDS	-	**S**
Cocc_00855	**ppsR**	PEP synthetase regulatory protein	Replication, recombination and repair	**L**
Cocc_00857	**-**	CDS	-	**S**
Cocc_00858	**mrpD**	Na(+)/H(+) antiporter subunit D	Inorganic ion transport and metabolism	**P**
Cocc_00863	**gyrB**	DNA gyrase subunit B	Replication, recombination and repair	**L**
Cocc_00881	**-**	CDS	-	**S**
Cocc_00883	**-**	CDS	-	**S**
Cocc_00884	**-**	recombinase A	Recombination	**L**
Cocc_00889	**virB8**	Type IV secretion system protein virB8	Bacterial secretion system	**U**
Cocc_00890	**ribA**	GTP cyclohydrolase-2	Riboflavin biosynthesis	**H**
Cocc_00897	**-**	putative bifunctional glutamate synthase subunit beta/2-polyprenylphenol hydroxylase	Amino acid transport and metabolism	**E**
Cocc_00906	**clpP**	ATP-dependent Clp protease proteolytic subunit	Misfolded protein degradation	**O**
Cocc_00908	**rpoA**	DNA-directed RNA polymerase subunit alpha	Purine metabolism	**K**
Cocc_00910	**rpsM**	30S ribosomal protein S13	Ribosome	**J**
Cocc_00921	**-**	CDS	-	**S**
Cocc_00931	**nqo3**	NADH-quinone oxidoreductase chain 3	Carbohydrate transport and metabolism	**G**
Cocc_00933	**pepA**	Cytosol aminopeptidase	Arginine and proline metabolism	**E**
Cocc_00934	**mnmE**	tRNA modification GTPase MnmE	Homologous recombination	**J**
Cocc_00943	**-**	CDS	-	**S**
Cocc_00944	**-**	CDS	-	**S**
Cocc_00950	**-**	CDS	-	**S**
Cocc_00954	**-**	EVE domain protein	-	**R**
Cocc_00955	**tuf**	elongation factor Tu	Translation	**J**
Cocc_00965	**rpmH**	50S ribosomal protein L34	Ribosome	**J**
Cocc_00971	**-**	CDS	-	**S**
Cocc_00972	**uvrC**	UvrABC system protein C	Nucleotide excision repair	**L**
Cocc_00980	**mnmA**	tRNA-specific 2-thiouridylase MnmA	Sulfur relay system	**H**
Cocc_00983	**ubiD**	3-octaprenyl-4-hydroxybenzoate carboxy-lyase	Ubiquinone and other terpenoid-quinone biosynthesis	**H**
Cocc_01001	**-**	CDS	-	**S**
Cocc_01003	**acr1**	Fatty acyl-CoA reductase	Arginine biosynthesis	**V**
Cocc_01004	**-**	CDS	-	**S**
Cocc_01007	**sdhA**	Succinate dehydrogenase flavoprotein subunit	Butanoate metabolism	**C**
Cocc_01010	**gyrA**	DNA gyrase subunit A	Replication, recombination and repair	**L**
Cocc_01022	**-**	CDS	-	**S**
Cocc_01027	**-**	CDS	-	**S**
Cocc_01029	**-**	CDS	-	**S**
Cocc_01032	**rimM**	16S rRNA-processing protein RimM	Translation, ribosomal structure and biogenesis	**J**
Cocc_01053	**-**	CDS	-	**S**
Cocc_01054	**ileS**	Isoleucine--tRNA ligase	Aminoacyl-tRNA biosynthesis	**J**
Cocc_01055	**-**	CDS	-	**S**
Cocc_01069	**pyrB**	Aspartate carbamoyltransferase	Alanine, aspartate and glutamate metabolism	**F**
Cocc_01072	**tsaC**	Threonylcarbamoyl-AMP synthase	Translation	**J**
Cocc_01080	**parE1**	Plasmid stabilisation system protein	General function prediction only	**R**
Cocc_01083	**-**	CDS	-	**S**
Cocc_01089	**sucC**	Succinyl-CoA ligase [ADP-forming] subunit beta	C5-Branched dibasic acid metabolism	**C**
Cocc_01107	**-**	CDS	-	**S**
Cocc_01115	**-**	CDS	-	**S**
Cocc_01125	**znuC**	Zinc import ATP-binding protein ZnuC	ABC transporters	**P**
Cocc_01134	**-**	CDS	-	**S**
Cocc_01141	**iscS**	Cysteine desulfurase	Alanine biosynthesis	**E**

Cocc_01150	nuoD	NADH-quinone oxidoreductase subunit D	Oxidative phosphorylation	C
Cocc_01163	-	CDS	-	S
Cocc_01168	gyrA	DNA gyrase subunit A	Replication, recombination and repair	L
Cocc_01181	clpA	ATP-dependent Clp protease ATP-binding subunit ClpA	Misfolded protein degradation	O
Cocc_01187	mrdB	Rod shape-determining protein RodA	Cell cycle control, cell division, chromosome partitioning	D
Cocc_01189	-	CDS	-	S
Cocc_01191	aspC	Aspartate aminotransferase	Asparagine biosynthesis	E
Cocc_01195	uhpC	regulatory protein UhpC	Two-component system	G
Cocc_01196	lolD	Lipoprotein-releasing system ATP-binding protein LolD	Two-component system	O
Cocc_01200	-	CDS	-	S
Cocc_01211	-	CDS	-	S
Cocc_01212	-	CDS	-	S
Cocc_01220	adiC	Arginine/agmatine antiporter	Amino acid transport	E
Cocc_01232	-	CDS	-	S
Cocc_01236	-	Bacterial transferase hexapeptide (six repeats)	General function prediction only	R
Cocc_01248	-	CDS	-	S
Cocc_01257	leuS	Leucine--tRNA ligase	Aminoacyl-tRNA biosynthesis	J
Cocc_01283	miaB	(Dimethylallyl)adenosine tRNA methylthiotransferase MiaB	Puromycin biosynthesis	J
Cocc_01304	-	CDS	-	S
Cocc_01322	guaB	Inosine-5'-monophosphate dehydrogenase	Purine metabolism	F
Cocc_01339	secF	preprotein translocase subunit SecF	Bacterial secretion system	U
Cocc_01362	-	CDS	-	S
Cocc_01363	-	CDS	-	S
Cocc_01393	-	CDS	-	S
Cocc_01399	addA	ATP-dependent helicase/nuclease subunit A	Base excision repair	L
Cocc_01408	-	CDS	-	S
Cocc_01416	-	CDS	-	S
Cocc_01456	-	CDS	-	S
Cocc_01553	tuf	elongation factor Tu	Translation	J
Haaa_00040	yabA	Initiation-control protein YabA	Replication	L
Haaa_00089	-	CDS	-	S
Haaa_00103	-	CDS	-	S
Haaa_00104	-	CDS	-	S
Haaa_00117	-	CDS	-	S
Haaa_00119	-	CDS	-	S
Haaa_00137	-	CDS	-	S
Haaa_00163	-	CDS	-	S
Haaa_00169	-	CDS	-	S
Haaa_00236	-	CDS	-	S
Haaa_00237	ankX	Phosphocholine transferase AnkX	Lipopolysaccharide biosynthesis	
Haaa_00238	-	CDS	-	S
Haaa_00239	-	CDS	-	S
Haaa_00256	-	CDS	-	S
Haaa_00258	-	CDS	-	S
Haaa_00261	ankX	Phosphocholine transferase AnkX	Lipopolysaccharide biosynthesis	I
Haaa_00262	-	CDS	-	S
Haaa_00264	-	CDS	-	S
Haaa_00265	-	CDS	-	S
Haaa_00270	-	CDS	-	S
Haaa_00283	-	Phage portal protein, lambda family	Replication, recombination and repair	L
Haaa_00287	-	CDS	-	S
Haaa_00289	-	CDS	-	S
Haaa_00291	-	Transposase	Transposition	L
Haaa_00293	-	Prophage minor tail protein Z (GPZ)	Replication, recombination and repair	L
Haaa_00294	-	CDS	-	S
Haaa_00295	-	Phage-related baseplate assembly protein	Replication, recombination and repair	L
Haaa_00296	-	CDS	-	S
Haaa_00297	-	Gene 25-like lysozyme	-	R
Haaa_00298	-	Baseplate J-like protein	General function prediction only	R
Haaa_00309	ankX	Phosphocholine transferase AnkX	Lipopolysaccharide biosynthesis	I
Haaa_00311	-	CDS	-	S
Haaa_00318	-	anaerobic benzoate catabolism transcriptional regulator	DNA binding	K
Haaa_00361	smc	Chromosome partition protein Smc	Cell cycle control, cell division, chromosome partitioning	D
Haaa_00436	-	Transposase DDE domain protein	Transposition	L
Haaa_00439	-	CDS	-	S
Haaa_00440	-	CDS	-	S
Haaa_00441	secA	preprotein translocase subunit SecA	Bacterial secretion system	MU
Haaa_00442	secA	preprotein translocase subunit SecA	Bacterial secretion system	MU
Haaa_00470	-	CDS	-	S
Haaa_00493	-	Transposase	Transposition	L
Haaa_00494	-	CDS	-	S
Haaa_00496	-	CDS	-	S
Haaa_00497	-	CDS	-	S
Haaa_00498	-	CDS	-	S
Haaa_00500	-	CDS	-	S
Haaa_00501	-	CDS	-	S
Haaa_00502	-	CDS	-	S
Haaa_00527	-	CDS	-	S
Haaa_00536	-	CDS	-	S
Haaa_00542	-	CDS	-	S
Haaa_00543	-	Transposase	Transposition	L
Haaa_00554	-	CDS	-	S
Haaa_00575	-	CDS	-	S
Haaa_00579	-	Transposase DDE domain protein	Transposition	L
Haaa_00611	-	Transposase DDE domain protein	Transposition	L
Haaa_00622	-	CDS	-	S

Haaa_00628	**-**	CDS	-	**S**
Haaa_00630	**-**	Ankyrin repeats (3 copies)	Ankyrin	**R**
Haaa_00685	**-**	CDS	-	**S**
Haaa_00775	**-**	CDS	-	**S**
Haaa_00786	**-**	CDS	-	**S**
Haaa_00818	**-**	CDS	-	**S**
Haaa_00835	**yabA**	Initiation-control protein YabA	Replication	**L**
Haaa_00841	**-**	CDS	-	**S**
Haaa_00931	**-**	CDS	-	**S**
Haaa_00943	**-**	CDS	-	**S**
Haaa_01122	**-**	CDS	-	**S**
Haaa_01130	**-**	CDS	-	**S**
Mell_00040	**-**	CDS	-	**S**
Mell_00046	**-**	Reverse transcriptase (RNA-dependent DNA polymerase)	Reverse transcriptase	**R**
Mell_00051	**-**	CDS	-	**S**
Mell_00063	**-**	Transposase	Transposition	**L**
Mell_00099	**-**	CDS	-	**S**
Mell_00101	**-**	CDS	-	**S**
Mell_00114	**-**	Transposase	Transposition	**L**
Mell_00238	**-**	CDS	-	**S**
Mell_00254	**-**	Helix-turn-helix	General function prediction only	**R**
Mell_00257	**-**	CDS	-	**S**
Mell_00259	**-**	CDS	-	**S**
Mell_00262	**-**	CDS	-	**S**
Mell_00275	**-**	CDS	-	**S**
Mell_00284	**-**	Phage-related baseplate assembly protein	Replication, recombination and repair	**L**
Mell_00285	**-**	Phage-related baseplate assembly protein	Replication, recombination and repair	**L**
Mell_00351	**-**	Transposase	Transposition	**L**
Mell_00353	**-**	Integrase core domain protein	General function prediction only	**R**
Mell_00424	**yjdL**	putative dipeptide and tripeptide permease YjdL	Chaperone	**O**
Mell_00463	**-**	CDS	-	**S**
Mell_00464	**ftsH1**	ATP-dependent zinc metalloprotease FtsH 1	Protein modification	**P**
Mell_00503	**-**	CDS	-	**S**
Mell_00506	**rnhA**	Ribonuclease HI	DNA replication	**L**
Mell_00509	**wapA**	tRNA nuclease WapA precursor	Translation, ribosomal structure and biogenesis	**J**
Mell_00510	**ankX**	Phosphocholine transferase AnkX	Lipopolysaccharide biosynthesis	**I**
Mell_00512	**-**	CDS	-	**S**
Mell_00519	**-**	Transposase	Transposition	**L**
Mell_00536	**-**	PD-(D/E)XK nuclease family transposase	Transposition	**L**
Mell_00570	**-**	CDS	-	**S**
Mell_00586	**-**	CDS	-	**S**
Mell_00624	**-**	CDS	-	**S**
Mell_00645	**-**	CDS	-	**S**
Mell_00721	**-**	CDS	-	**S**
Mell_00790	**-**	CDS	-	**S**
Mell_00888	**-**	CDS	-	**S**
Mell_00889	**-**	CDS	-	**S**
Mell_00897	**-**	Transposase DDE domain protein	Transposition	**L**
Mell_00922	**-**	CDS	-	**S**
Mell_01107	**-**	CDS	-	**S**
Mell_01205	**-**	CDS	-	**S**
Mell_01221	**-**	CDS	-	**S**
Mell_01256	**-**	CDS	-	**S**
Mell_01258	**-**	CDS	-	**S**
Mell_01275	**-**	TrbC/VIRB2 family protein	General function prediction only	**R**
Mell_01301	**-**	CDS	-	**S**
Nooo_00023	**-**	CDS	-	**S**
Nooo_00027	**-**	CDS	-	**S**
Nooo_00055	**-**	CDS	-	**S**
Nooo_00056	**-**	CDS	-	**S**
Nooo_00058	**-**	CDS	-	**S**
Nooo_00059	**-**	CDS	-	**S**
Nooo_00061	**-**	CDS	-	**S**
Nooo_00070	**-**	CDS	-	**S**
Nooo_00071	**-**	Transposase IS116/IS110/IS902 family protein	Transposition	**L**
Nooo_00092	**-**	CDS	-	**S**
Nooo_00108	**-**	CDS	-	**S**
Nooo_00119	**-**	Gene 25-like lysozyme	-	**R**
Nooo_00120	**-**	CDS	-	**S**
Nooo_00125	**-**	CDS	-	**S**
Nooo_00127	**-**	CDS	-	**S**
Nooo_00132	**ankX**	Phosphocholine transferase AnkX	Lipopolysaccharide biosynthesis	**I**
Nooo_00144	**-**	CDS	-	**S**
Nooo_00145	**-**	Transposase	Transposition	**L**
Nooo_00147	**-**	CDS	-	**S**
Nooo_00155	**-**	CDS	-	**S**
Nooo_00160	**-**	CDS	-	**S**
Nooo_00170	**-**	CDS	-	**S**
Nooo_00226	**ltrA**	Group II intron-encoded protein LtrA	Amino acid transport and metabolism	**E**
Nooo_00233	**-**	CDS	-	**S**
Nooo_00259	**-**	CDS	-	**S**
Nooo_00348	**-**	CDS	-	**S**
Nooo_00349	**-**	CDS	-	**S**
Nooo_00355	**-**	Ankyrin repeats (3 copies)	Ankyrin	**R**
Nooo_00371	**-**	CDS	-	**S**
Nooo_00390	**-**	CDS	-	**S**
Nooo_00401	**-**	CDS	-	**S**

Nooo_00402	**-**	CDS	-	**S**
Nooo_00405	**-**	CDS	-	**S**
Nooo_00408	**-**	CDS	-	**S**
Nooo_00417	**-**	CDS	-	**S**
Nooo_00425	**-**	Ankyrin repeats (3 copies)	Ankyrin	**R**
Nooo_00454	**-**	CDS	-	**S**
Nooo_00455	**-**	CDS	-	**S**
Nooo_00466	**-**	CDS	-	**S**
Nooo_00467	**-**	Ankyrin repeats (3 copies)	Ankyrin	**R**
Nooo_00468	**-**	CDS	-	**S**
Nooo_00471	**-**	CDS	-	**S**
Nooo_00472	**-**	CDS	-	**S**
Nooo_00547	**-**	CDS	-	**S**
Nooo_00553	**-**	anaerobic benzoate catabolism transcriptional regulator	DNA binding	**K**
Nooo_00573	**-**	CDS	-	**S**
Nooo_00577	**mutM**	Formamidopyrimidine-DNA glycosylase	Base excision repair	**L**
Nooo_00596	**-**	CDS	-	**S**
Nooo_00625	**-**	CDS	-	**S**
Nooo_00626	**-**	CDS	-	**S**
Nooo_00674	**-**	CDS	-	**S**
Nooo_00715	**-**	CDS	-	**S**
Nooo_00716	**-**	CDS	-	**S**
Nooo_00723	**-**	Ankyrin repeats (3 copies)	Ankyrin	**R**
Nooo_00725	**-**	Piwi/Argonaute/Zwille siRNA-binding domain protein	General function prediction only	**R**
Nooo_00782	**-**	CDS	-	**S**
Nooo_00789	**-**	Baseplate J-like protein	General function prediction only	**R**
Nooo_00793	**tnpR**	Transposon gamma-delta resolvase	Transposition	**L**
Nooo_00830	**-**	CDS	-	**S**
Nooo_00831	**dnaJ**	chaperone protein DnaJ	Chaperone	**O**
Nooo_00859	**-**	CDS	-	**S**
Nooo_00922	**-**	CDS	-	**S**
Nooo_00945	**-**	CDS	-	**S**
Nooo_00950	**-**	CDS	-	**S**
Nooo_00970	**-**	CDS	-	**S**
Nooo_00980	**gpW**	bacteriophage P2 baseplate assembly protein	Replication, recombination and repair	**L**
Nooo_00983	**sppA**	Putative signal peptide peptidase SppA	beta-Lactam resistance	**OU**
Nooo_00984	**ankX**	Phosphocholine transferase AnkX	Lipopolysaccharide biosynthesis	**I**
Nooo_00985	**-**	OTU-like cysteine protease	General function prediction only	**R**
Nooo_00987	**-**	CDS	-	**S**
Nooo_00990	**-**	DNA mismatch repair protein	Replication, recombination and repair	**L**
Nooo_00991	**mutL**	DNA mismatch repair protein MutL	Mismatch repair	**L**
Nooo_00992	**-**	CDS	-	**S**
Nooo_01026	**-**	CDS	-	**S**
Nooo_01039	**-**	Ankyrin repeats (3 copies)	Ankyrin	**R**
Nooo_01048	**-**	CDS	-	**S**
Nooo_01050	**-**	CDS	-	**S**
Nooo_01092	**-**	CDS	-	**S**
Nooo_01093	**-**	CDS	-	**S**
Nooo_01096	**-**	CDS	-	**S**
Nooo_01097	**-**	CDS	-	**S**
Nooo_01110	**-**	CDS	-	**S**
Nooo_01118	**spvB**	Mono(ADP-ribosyl)transferase SpvB	General function prediction only	**R**
Nooo_01119	**-**	CDS	-	**S**
Nooo_01120	**-**	CDS	-	**S**
Nooo_01121	**-**	Kelch motif protein	General function prediction only	**R**
Nooo_01128	**-**	CDS	-	**S**
Nooo_01129	**-**	CDS	-	**S**
Nooo_01153	**-**	CDS	-	**S**
Nooo_01159	**-**	CDS	-	**S**
Nooo_01163	**-**	CDS	-	**S**
Nooo_01164	**-**	CDS	-	**S**
Nooo_01165	**-**	Ankyrin repeat protein	Ankyrin	**R**
Nooo_01166	**ankX**	Phosphocholine transferase AnkX	Lipopolysaccharide biosynthesis	**I**
Nooo_01167	**-**	Ankyrin repeats (3 copies)	Ankyrin	**R**
Nooo_01168	**-**	Ankyrin repeat protein	Ankyrin	**R**
Nooo_01171	**-**	CDS	-	**S**
Nooo_01209	**dnaQ**	DNA polymerase III subunit epsilon	Purine metabolism	**L**
Nooo_01210	**-**	CDS	-	**S**
Nooo_01232	**-**	CDS	-	**S**
Nooo_01240	**-**	CDS	-	**S**
Nooo_01245	**-**	Integrase core domain protein	General function prediction only	**R**
Nooo_01248	**-**	CDS	-	**S**
Nooo_01249	**-**	CDS	-	**S**
Pipp_00017	**-**	CDS	-	**S**
Pipp_00029	**-**	CDS	-	**S**
Pipp_00031	**-**	CDS	-	**S**
Pipp_00032	**-**	CDS	-	**S**
Pipp_00033	**-**	CDS	-	**S**
Pipp_00048	**-**	Transposase DDE domain protein	Transposition	**L**
Pipp_00052	**-**	Transposase, Mutator family	Transposition	**L**
Pipp_00053	**-**	Transposase, Mutator family	Transposition	**L**
Pipp_00058	**-**	CDS	-	**S**
Pipp_00063	**-**	CDS	-	**S**
Pipp_00069	**-**	CDS	-	**S**
Pipp_00070	**-**	Transposase IS200 like protein	Transposition	**L**
Pipp_00071	**-**	CDS	-	**S**
Pipp_00074	**-**	Transposase, Mutator family	Transposition	**L**
Pipp_00114	**-**	CDS	-	**S**

Pipp_00115	**-**	CDS	-	**S**
Pipp_00121	**-**	CDS	-	**S**
Pipp_00128	**-**	CDS	-	**S**
Pipp_00158	**-**	Transposase, Mutator family	Transposition	**L**
Pipp_00159	**-**	Transposase, Mutator family	Transposition	**L**
Pipp_00164	**-**	CDS	-	**S**
Pipp_00171	**-**	CDS	-	**S**
Pipp_00174	**-**	CDS	-	**S**
Pipp_00181	**-**	CDS	-	**S**
Pipp_00183	**-**	Group II intron-encoded protein LtrA	Amino acid transport and metabolism	**E**
Pipp_00184	**-**	CDS	-	**S**
Pipp_00204	**-**	Transposase	Transposition	**L**
Pipp_00217	**-**	CDS	-	**S**
Pipp_00232	**-**	CDS	-	**S**
Pipp_00237	**-**	Ankyrin repeats (3 copies)	Ankyrin	**R**
Pipp_00257	**-**	Transposase DDE domain protein	Transposition	**L**
Pipp_00258	**-**	CDS	-	**S**
Pipp_00261	**-**	CDS	-	**S**
Pipp_00262	**-**	CDS	-	**S**
Pipp_00264	**-**	Gene 25-like lysozyme	-	**R**
Pipp_00269	**-**	CDS	-	**S**
Pipp_00271	**-**	CDS	-	**S**
Pipp_00274	**-**	bacteriophage P2 baseplate assembly protein	Replication, recombination and repair	**L**
Pipp_00277	**-**	CDS	-	**S**
Pipp_00280	**-**	CDS	-	**S**
Pipp_00284	**-**	CDS	-	**S**
Pipp_00285	**-**	putative RNA polymerase sigma factor SigI	Transcription	**K**
Pipp_00286	**-**	CDS	-	**S**
Pipp_00287	**-**	CDS	-	**S**
Pipp_00288	**-**	CDS	-	**S**
Pipp_00291	**-**	CDS	-	**S**
Pipp_00301	**ltrA**	Group II intron-encoded protein LtrA	Amino acid transport and metabolism	**E**
Pipp_00310	**-**	CDS	-	**S**
Pipp_00311	**-**	anaerobic benzoate catabolism transcriptional regulator	DNA binding	**K**
Pipp_00319	**-**	CDS	-	**S**
Pipp_00320	**-**	Ankyrin repeats (3 copies)	Ankyrin	**R**
Pipp_00321	**-**	Ankyrin repeat protein	Ankyrin	**R**
Pipp_00328	**-**	Prophage minor tail protein Z (GPZ)	Replication, recombination and repair	**L**
Pipp_00344	**-**	Phage terminase large subunit (GpA)	Replication, recombination and repair	**L**
Pipp_00364	**-**	Transposase, Mutator family	Transposition	**L**
Pipp_00368	**dnaJ**	chaperone protein DnaJ	Chaperone	**O**
Pipp_00371	**res**	Type III restriction enzyme, res subunit	General function prediction only	**R**
Pipp_00372	**-**	CDS	-	**S**
Pipp_00381	**-**	Transposase, Mutator family	Transposition	**L**
Pipp_00394	**-**	CDS	-	**S**
Pipp_00397	**-**	Phage portal protein, lambda family	Replication, recombination and repair	**L**
Pipp_00416	**-**	CDS	-	**S**
Pipp_00423	**-**	CDS	-	**S**
Pipp_00439	**-**	CDS	-	**S**
Pipp_00440	**-**	Glyoxalase-like domain protein	General function prediction only	**R**
Pipp_00451	**-**	CDS	-	**S**
Pipp_00455	**-**	CDS	-	**S**
Pipp_00456	**-**	CDS	-	**S**
Pipp_00459	**-**	CDS	-	**S**
Pipp_00460	**-**	CDS	-	**S**
Pipp_00461	**-**	CDS	-	**S**
Pipp_00462	**-**	CDS	-	**S**
Pipp_00463	**-**	Phage tail sheath protein	Replication, recombination and repair	**L**
Pipp_00464	**-**	Phage tail tube protein FII	Replication, recombination and repair	**L**
Pipp_00466	**-**	Phage-related minor tail protein	Replication, recombination and repair	**L**
Pipp_00467	**-**	Phage P2 GpU	Replication, recombination and repair	**L**
Pipp_00472	**-**	Phospholipase D precursor	CAMP signaling pathway	**I**
Pipp_00474	**-**	CDS	-	**S**
Pipp_00475	**-**	anaerobic benzoate catabolism transcriptional regulator	DNA binding	**K**
Pipp_00477	**-**	CDS	-	**S**
Pipp_00483	**phnO**	aminoalkylphosphonic acid N-acetyltransferase	General function prediction only	**R**
Pipp_00484	**bacE**	Putative bacilysin exporter BacE	Defense mechanisms	**V**
Pipp_00490	**-**	CDS	-	**S**
Pipp_00493	**-**	CDS	-	**S**
Pipp_00500	**-**	CDS	-	**S**
Pipp_00535	**-**	CDS	-	**S**
Pipp_00536	**-**	CDS	-	**S**
Pipp_00547	**-**	CDS	-	**S**
Pipp_00723	**ftsH**	ATP-dependent zinc metalloprotease FtsH	Protein modification	**O**
Pipp_00769	**-**	Ankyrin repeats (3 copies)	Ankyrin	**R**
Pipp_00815	**-**	CDS	-	**S**
Pipp_00880	**-**	CDS	-	**S**
Pipp_00930	**-**	CDS	-	**S**
Pipp_00933	**-**	Phosphotransferase enzyme family protein	General function prediction only	**R**
Pipp_00950	**-**	CDS	-	**S**
Pipp_01023	**-**	Group II intron, maturase-specific domain	General function prediction only	**R**
Pipp_01060	**-**	CDS	-	**S**
Pipp_01071	**-**	Transposase, Mutator family	Transposition	**L**
Pipp_01094	**-**	CDS	-	**S**
Pipp_01169	**-**	CDS	-	**S**
Pipp_01193	**-**	CDS	-	**S**
Pipp_01202	**-**	Ankyrin repeats (3 copies)	Ankyrin	**R**
Pipp_01204	**-**	CDS	-	**S**

Pipp_01265	-	CDS	-	S
Pipp_01270	-	Surface antigen	Defense	R
Pipp_01273	-	CDS	-	S
Pipp_01327	-	CDS	-	S
Pipp_01328	-	CDS	-	S
Pipp_01352	-	CDS	-	S
Pipp_01407	-	CDS	-	S
Pipp_01408	-	CDS	-	S
Pipp_01418	-	Transposase, Mutator family	Transposition	L
Pipp_01420	-	Phage portal protein, lambda family	Replication, recombination and repair	L
Pipp_01423	-	Phage terminase large subunit (GpA)	Replication, recombination and repair	L
Riii_00139	-	Transposase	Transposition	L
Riii_00377	-	CDS	-	S
Riii_00394	-	CDS	-	S
Riii_00415	-	CDS	-	S
Riii_00497	-	CDS	-	S
Riii_00511	-	CDS	-	S
Riii_00519	-	Transposase	Transposition	L
Riii_00577	-	CDS	-	S
Riii_00580	-	CDS	-	S
Riii_00592	-	CDS	-	S
Riii_00596	-	CDS	-	S
Riii_00608	**hepA**	ATP-dependent helicase HepA	Replication	L
Riii_00609	**glmU**	Bifunctional protein GlmU	Amino sugar and nucleotide sugar metabolism	M
Riii_00610	**galE**	UDP-glucose 4-epimerase	Amino sugar and nucleotide sugar metabolism	M
Riii_00611	**mshA**	D-inositol 3-phosphate glycosyltransferase	Defense mechanisms	V
Riii_00612	-	CDS	-	S
Riii_00613	**phyH**	Phytanoyl-CoA dioxygenase (PhyH)	General function prediction only	R
Riii_00615	**ltaA**	L-allo-threonine aldolase	Carbohydrate transport and metabolism	G
Riii_00616	**ltaA**	L-allo-threonine aldolase	Carbohydrate transport and metabolism	G
Riii_00617	**glpT**	Glycerol-3-phosphate transporter	Carbohydrate transport and metabolism	G
Riii_00620	-	anaerobic benzoate catabolism transcriptional regulator	DNA binding	K
Riii_00625	-	PD-(D/E)XK nuclease family transposase	Transposition	L
Riii_00653	-	CDS	-	S
Riii_00654	-	Transposase	Transposition	L
Riii_00732	-	Ankyrin repeats (3 copies)	Ankyrin	R
Riii_00769	-	CDS	-	S
Riii_00853	-	CDS	-	S
Riii_00855	-	CDS	-	S
Riii_00859	-	Transposase IS66 family protein	Transposition	L
Riii_00871	-	Transposase IS66 family protein	Transposition	L
Riii_00872	-	Transposase IS66 family protein	Transposition	L
Riii_01021	-	CDS	-	S
Riii_01245	-	Transposase IS116/IS110/IS902 family protein	Transposition	L
Suzz_00001	-	CDS	-	S
Suzz_00040	-	CDS	-	S
Suzz_00545	-	CDS	-	S
Suzz_00609	-	CDS	-	S
Suzz_00647	-	CDS	-	S
Suzz_00871	-	CDS	-	S
Suzz_00938	-	CDS	-	S

PANGENOME Venn: 677 C+D+F Only

ID	Gene Symbol	Gene Product	Pathway or Process	COG
Bmmm_00034	-	CDS	-	S
Bmmm_00035	-	CDS	-	S
Bmmm_00038	-	CDS	-	S
Bmmm_00039	-	CDS	-	S
Bmmm_00040	-	CDS	-	S
Bmmm_00044	-	CDS	-	S
Bmmm_00045	-	CDS	-	S
Bmmm_00046	-	CDS	-	S
Bmmm_00047	-	CDS	-	S
Bmmm_00048	-	CDS	-	S
Bmmm_00054	-	CDS	-	S
Bmmm_00062	-	CDS	-	S
Bmmm_00069	-	CDS	-	S
Bmmm_00071	-	CDS	-	S
Bmmm_00081	-	CDS	-	S
Bmmm_00083	-	CDS	-	S
Bmmm_00086	-	CDS	-	S
Bmmm_00091	-	CDS	-	S
Bmmm_00092	-	CDS	-	S
Bmmm_00094	-	CDS	-	S
Bmmm_00095	**argD**	Acetylornithine aminotransferase	Arginine biosynthesis	E
Bmmm_00109	-	CDS	-	S
Bmmm_00110	-	CDS	-	S
Bmmm_00111	-	CDS	-	S
Bmmm_00119	-	CDS	-	S
Bmmm_00121	-	CDS	-	S
Bmmm_00124	-	CDS	-	S
Bmmm_00129	-	CDS	-	S
Bmmm_00132	**clpA**	ATP-dependent Clp protease ATP-binding subunit ClpA	Posttranslational modification, protein turnover, chaperones	O
Bmmm_00136	-	CDS	-	S
Bmmm_00137	-	putative CtpA-like serine protease	General function prediction only	R

Bmmm_00146	-	CDS	-	S
Bmmm_00147	-	CDS	-	S
Bmmm_00149	**proP**	Proline/betaine transporter	Transporter/osmoregulator	**GEP**
Bmmm_00150	-	CDS	-	S
Bmmm_00153	-	CDS	-	S
Bmmm_00177	-	CDS	-	S
Bmmm_00178	-	CDS	-	S
Bmmm_00189	-	CDS	-	S
Bmmm_00190	-	CDS	-	S
Bmmm_00191	-	Ankyrin repeats (3 copies)	Ankyrin	R
Bmmm_00192	-	CDS	-	S
Bmmm_00194	-	CDS	-	S
Bmmm_00209	-	Surface antigen	Defense	R
Bmmm_00219	-	CDS	-	S
Bmmm_00220	-	CDS	-	S
Bmmm_00223	-	CDS	-	S
Bmmm_00227	-	CDS	-	S
Bmmm_00229	-	CDS	-	S
Bmmm_00230	-	CDS	-	S
Bmmm_00232	-	CDS	-	S
Bmmm_00234	-	CDS	-	S
Bmmm_00236	-	CDS	-	S
Bmmm_00238	-	PD-(D/E)XK nuclease family transposase	Transposition	L
Bmmm_00248	-	CDS	-	S
Bmmm_00251	-	CDS	-	S
Bmmm_00271	-	CDS	-	S
Bmmm_00273	-	CDS	-	S
Bmmm_00277	-	CDS	-	S
Bmmm_00280	-	CDS	-	S
Bmmm_00281	-	CDS	-	S
Bmmm_00283	-	CDS	-	S
Bmmm_00287	-	CDS	-	S
Bmmm_00288	-	CDS	-	S
Bmmm_00289	-	CDS	-	S
Bmmm_00291	-	CDS	-	S
Bmmm_00292	-	CDS	-	S
Bmmm_00293	-	CDS	-	S
Bmmm_00311	-	CDS	-	S
Bmmm_00325	-	CDS	-	S
Bmmm_00328	-	CDS	-	S
Bmmm_00346	-	CDS	-	S
Bmmm_00347	-	CDS	-	S
Bmmm_00349	-	CDS	-	S
Bmmm_00360	-	CDS	-	S
Bmmm_00367	-	CDS	-	S
Bmmm_00375	-	CDS	-	S
Bmmm_00376	-	CDS	-	S
Bmmm_00378	-	CDS	-	S
Bmmm_00381	-	CDS	-	S
Bmmm_00391	-	CDS	-	S
Bmmm_00392	-	CDS	-	S
Bmmm_00408	-	CDS	-	S
Bmmm_00414	-	CDS	-	S
Bmmm_00420	-	CDS	-	S
Bmmm_00421	-	CDS	-	S
Bmmm_00428	-	CDS	-	S
Bmmm_00497	-	CDS	-	S
Bmmm_00503	-	CDS	-	S
Bmmm_00507	-	CDS	-	S
Bmmm_00522	-	CDS	-	S
Bmmm_00526	-	PD-(D/E)XK nuclease family transposase	Transposition	L
Bmmm_00527	**sucA**	2-oxoglutarate dehydrogenase E1 component	Carbon metabolism	C
Bmmm_00540	-	Ankyrin repeats (3 copies)	Ankyrin	R
Bmmm_00551	-	CDS	-	S
Bmmm_00552	-	Ankyrin repeats (3 copies)	Ankyrin	R
Bmmm_00554	-	CDS	-	S
Bmmm_00555	-	CDS	-	S
Bmmm_00556	-	CDS	-	S
Bmmm_00557	-	CDS	-	S
Bmmm_00566	-	putative ABC transporter ATP-binding protein	General function prediction only	R
Bmmm_00567	-	CDS	-	S
Bmmm_00568	-	CDS	-	S
Bmmm_00570	-	CDS	-	S
Bmmm_00572	-	CDS	-	S
Bmmm_00583	-	CDS	-	S
Bmmm_00588	-	CDS	-	S
Bmmm_00590	-	CDS	-	S
Bmmm_00591	-	CDS	-	S
Bmmm_00602	-	CDS	-	S
Bmmm_00607	-	CDS	-	S
Bmmm_00617	-	CDS	-	S
Bmmm_00620	-	CDS	-	S
Bmmm_00635	-	CDS	-	S
Bmmm_00636	-	CDS	-	S
Bmmm_00638	-	CDS	-	S
Bmmm_00644	-	CDS	-	S
Bmmm_00650	-	CDS	-	S
Bmmm_00652	-	CDS	-	S

Bmmm_00653	**tolB**	translocation protein TolB	Cell wall/membrane/envelope biogenesis	**M**
Bmmm_00665	**-**	CDS	-	**S**
Bmmm_00667	**-**	CDS	-	**S**
Bmmm_00670	**-**	CDS	-	**S**
Bmmm_00671	**-**	CDS	-	**S**
Bmmm_00673	**-**	CDS	-	**S**
Bmmm_00680	**algA**	Alginate biosynthesis protein AlgA	Carbohydrate transport and metabolism	**G**
Bmmm_00698	**-**	CDS	-	**S**
Bmmm_00699	**-**	CDS	-	**S**
Bmmm_00705	**-**	CDS	-	**S**
Bmmm_00707	-	CDS	-	**S**
Bmmm_00714	**-**	CDS	-	**S**
Bmmm_00716	**-**	CDS	-	**S**
Bmmm_00721	**-**	CDS	-	**S**
Bmmm_00732	**-**	CDS	-	**S**
Bmmm_00733	**-**	CDS	-	**S**
Bmmm_00734	-	CDS	-	**S**
Bmmm_00735	**-**	CDS	-	**S**
Bmmm_00736	**-**	CDS	-	**S**
Bmmm_00738	**-**	CDS	-	**S**
Bmmm_00740	**-**	CDS	-	**S**
Bmmm_00748	**-**	CDS	-	**S**
Bmmm_00750	**-**	CDS	-	**S**
Bmmm_00756	**-**	CDS	-	**S**
Bmmm_00758	-	CDS	-	**S**
Bmmm_00759	**-**	CDS	-	**S**
Bmmm_00760	**-**	CDS	-	**S**
Bmmm_00763	-	CDS	-	**S**
Bmmm_00833	**pdxH**	Pyridoxine/pyridoxamine 5'-phosphate oxidase	Pyridoxal-5'-phosphate biosynthesis	**C**
Bmmm_00835	**-**	CDS	-	**S**
Bmmm_00837	**-**	CDS	-	**S**
Bmmm_00838	**-**	Competence protein	Replication, recombination and repair	**L**
Bmmm_00840	**-**	CDS	-	**S**
Bmmm_00848	**-**	CDS	-	**S**
Bmmm_00862	**-**	CDS	-	**S**
Bmmm_00864	**-**	CDS	-	**S**
Bmmm_00889	**-**	CDS	-	**S**
Bmmm_00890	**-**	CDS	-	**S**
Bmmm_00894	-	CDS	-	**S**
Bmmm_00895	-	PD-(D/E)XK nuclease family transposase	Transposition	**L**
Bmmm_00896	**-**	CDS	-	**S**
Bmmm_00897	**-**	CDS	-	**S**
Bmmm_00903	-	CDS	-	**S**
Bmmm_00910	**-**	CDS	-	**S**
Bmmm_00945	**-**	CDS	-	**S**
Bmmm_00952	-	CDS	-	**S**
Bmmm_00971	-	CDS	-	**S**
Bmmm_00973	**-**	CDS	-	**S**
Bmmm_00977	**-**	CDS	-	**S**
Bmmm_00994	**-**	CDS	-	**S**
Bmmm_00999	**-**	Ankyrin repeats (3 copies)	Ankyrin	**R**
Bmmm_01000	-	CDS	-	**S**
Bmmm_01001	-	CDS	-	**S**
Bmmm_01002	**-**	CDS	-	**S**
Bmmm_01003	**-**	CDS	-	**S**
Bmmm_01005	**-**	CDS	-	**S**
Bmmm_01023	**-**	CDS	-	**S**
Bmmm_01024	**-**	CDS	-	**S**
Bmmm_01028	**-**	CDS	-	**S**
Bmmm_01030	**-**	CDS	-	**S**
Bmmm_01031	-	CDS	-	**S**
Bmmm_01042	**-**	CDS	-	**S**
Bmmm_01043	**-**	CDS	-	**S**
Bmmm_01049	**-**	CDS	-	**S**
Bmmm_01050	**-**	CDS	-	**S**
Bmmm_01071	**-**	CDS	-	**S**
Bmmm_01076	**-**	CDS	-	**S**
Bmmm_01077	-	CDS	-	**S**
Bmmm_01079	**-**	CDS	-	**S**
Bmmm_01080	**-**	CDS	-	**S**
Bmmm_01090	-	CDS	-	**S**
Bmmm_01092	**radA**	DNA repair protein RadA	Posttranslational modification, protein turnover, chaperones	**O**
Bmmm_01099	**-**	CDS	-	**S**
Bmmm_01117	**-**	CDS	-	**S**
Bmmm_01131	**-**	CDS	-	**S**
Bmmm_01135	-	CDS	-	**S**
Bmmm_01136	**-**	CDS	-	**S**
Bmmm_01139	**-**	CDS	-	**S**
Bmmm_01141	-	CDS	-	**S**
Bmmm_01143	**-**	CDS	-	**S**
Clee_00015	-	Na+/H+ antiporter family protein	Energy production and conversion	**C**
Clee_00021	-	CDS	-	**S**
Clee_00052	**divL**	Sensor protein DivL	Signal transduction	**U**
Clee_00064	**-**	CDS	-	**S**
Clee_00066	**ydhC**	Inner membrane transport protein YdhC	Intracellular trafficking, secretion, and vesicular transport	**U**

Clee_00067	**ydhC**	Inner membrane transport protein YdhC	Intracellular trafficking, secretion, and vesicular transport	**U**
Clee_00089	**-**	CDS	-	**S**
Clee_00090	**-**	CDS	-	**S**
Clee_00101	**-**	CDS	-	**S**
Clee_00105	**-**	CDS	-	**S**
Clee_00106	**-**	CDS	-	**S**
Clee_00121	**-**	CDS	-	**S**
Clee_00134	**-**	CDS	-	**S**
Clee_00135	**-**	CDS	-	**S**
Clee_00143	**gudP**	putative glucarate transporter	Posttranslational modification, protein turnover, chaperones	**O**
Clee_00165	**-**	Ankyrin repeat protein	Ankyrin	**R**
Clee_00181	**-**	CDS	-	**S**
Clee_00193	**-**	CDS	-	**S**
Clee_00194	**-**	CDS	-	**S**
Clee_00195	**-**	CDS	-	**S**
Clee_00196	-	CDS	-	**S**
Clee_00260	**trkG**	Trk system potassium uptake protein TrkG	Inorganic ion transport and metabolism	**P**
Clee_00262	**-**	CDS	-	**S**
Clee_00266	**-**	CDS	-	**S**
Clee_00272	**-**	CDS	-	**S**
Clee_00283	**-**	CDS	-	**S**
Clee_00291	**-**	CDS	-	**S**
Clee_00299	**thiM**	Hydroxyethylthiazole kinase	Thiamine metabolism	**H**
Clee_00300	**thiD**	Hydroxymethylpyrimidine/phosphomethylpyrimidine kinase	Thiamine biosynthesis/salvage	**H**
Clee_00301	**tenA**	Thiaminase-2	Thiamine biosynthesis/salvage	**H**
Clee_00304	**-**	CDS	-	**S**
Clee_00305	-	Recombinase	Recombination	**L**
Clee_00342	**-**	PD-(D/E)XK nuclease family transposase	Transposition	**L**
Clee_00360	**purB**	Adenylosuccinate lyase	Alanine, aspartate and glutamate metabolism	**F**
Clee_00384	**-**	TrbC/VIRB2 family protein	Bacterial secretion system	**U**
Clee_00387	-	CDS	-	**S**
Clee_00390	-	CDS	-	**S**
Clee_00431	**-**	Transposase DDE domain protein	Transposition	**L**
Clee_00443	**-**	CDS	-	**S**
Clee_00450	**-**	CDS	-	**S**
Clee_00451	**-**	CDS	-	**S**
Clee_00458	**-**	CDS	-	**S**
Clee_00470	**-**	CDS	-	**S**
Clee_00472	**-**	CDS	-	**S**
Clee_00483	**-**	CDS	-	**S**
Clee_00484	-	CDS	-	**S**
Clee_00487	**-**	CDS	-	**S**
Clee_00488	**-**	CDS	-	**S**
Clee_00494	-	CDS	-	**S**
Clee_00495	-	CDS	-	**S**
Clee_00496	**-**	CDS	-	**S**
Clee_00497	-	CDS	-	**S**
Clee_00498	**-**	CDS	-	**S**
Clee_00499	**relG**	Toxin RelG	Mulitple COGs	**JD**
Clee_00500	-	CDS	-	**S**
Clee_00501	-	CDS	-	**S**
Clee_00503	-	CDS	-	**S**
Clee_00504	-	CDS	-	**S**
Clee_00526	-	CDS	-	**S**
Clee_00532	**ankX**	Phosphocholine transferase AnkX	Lipopolysaccharide biosynthesis	**I**
Clee_00538	**-**	PD-(D/E)XK nuclease family transposase	Transposition	**L**
Clee_00539	**-**	CDS	-	**S**
Clee_00549	**clpA**	ATP-dependent Clp protease ATP-binding subunit ClpA	Posttranslational modification, protein turnover, chaperones	**O**
Clee_00559	-	PD-(D/E)XK nuclease family transposase	Transposition	**L**
Clee_00575	-	CDS	-	**S**
Clee_00579	-	Ankyrin repeats (3 copies)	Ankyrin	**R**
Clee_00581	**pleD**	Response regulator PleD	Signal transduction mechanisms	**T**
Clee_00591	**metK**	S-adenosylmethionine synthase	Biosynthesis of amino acids	**H**
Clee_00628	**-**	CDS	-	**S**
Clee_00629	-	putative transporter	Carbohydrate transport and metabolism	**G**
Clee_00630	-	bicyclomycin/multidrug efflux system	General function prediction only	**R**
Clee_00635	**-**	CDS	-	**S**
Clee_00647	-	CDS	-	**S**
Clee_00668	**pvuIIM**	Modification methylase PvuII	Replication, recombination and repair	**L**
Clee_00696	**-**	CDS	-	**S**
Clee_00706	-	Ankyrin repeats (3 copies)	Ankyrin	**R**
Clee_00725	**-**	CDS	-	**S**
Clee_00732	-	flagellar assembly protein H	Cell motility	**N**
Clee_00751	**-**	CDS	-	**S**
Clee_00773	-	CDS	-	**S**
Clee_00774	**-**	CDS	-	**S**
Clee_00775	-	CDS	-	**S**
Clee_00776	-	CDS	-	**S**
Clee_00785	-	CDS	-	**S**
Clee_00789	-	CDS	-	**S**
Clee_00810	-	CDS	-	**S**
Clee_00835	-	CDS	-	**S**
Clee_00850	-	CDS	-	**S**
Clee_00862	**-**	CDS	-	**S**

Clee_00863	**-**	CDS	-	**S**
Clee_00864	**-**	CDS	-	**S**
Clee_00867	**-**	CDS	-	**S**
Clee_00868	**-**	CDS	-	**S**
Clee_00874	**-**	CDS	-	**S**
Clee_00881	**-**	CDS	-	**S**
Clee_00882	**ankX**	Phosphocholine transferase AnkX	Lipopolysaccharide biosynthesis	**I**
Clee_00888	**-**	CDS	-	**S**
Clee_00896	**pvuIIR**	Type-2 restriction enzyme PvuII	Replication, recombination and repair	**L**
Clee_00911	**-**	CDS	-	**S**
Clee_00924	**-**	Ankyrin repeat protein	Ankyrin	**R**
Clee_00925	**-**	CDS	-	**S**
Clee_00928	**-**	CDS	-	**S**
Clee_00935	**-**	PD-(D/E)XK nuclease family transposase	Transposition	**L**
Clee_00936	**-**	PD-(D/E)XK nuclease family transposase	Transposition	**L**
Clee_00940	**-**	CDS	-	**S**
Clee_00941	**ankX**	Phosphocholine transferase AnkX	Lipopolysaccharide biosynthesis	**I**
Clee_00942	**-**	CDS	-	**S**
Clee_00947	**-**	CDS	-	**S**
Clee_00948	**-**	CDS	-	**S**
Clee_00949	**-**	PD-(D/E)XK nuclease family transposase	Transposition	**L**
Clee_00955	**bioA**	Adenosylmethionine-8-amino-7-oxononanoate aminotransferase	Biotin biosynthesis	**H**
Clee_00956	**bioD**	ATP-dependent dethiobiotin synthetase BioD	Biotin biosynthesis	**H**
Clee_00957	**bioC**	Malonyl-[acyl-carrier protein] O-methyltransferase	Biotin biosynthesis	**H**
Clee_00958	**-**	CDS	-	**S**
Clee_00959	**bioF**	8-amino-7-oxononanoate synthase 2	Biotin biosynthesis	**H**
Clee_00960	**bioB**	Biotin synthase	Biotin biosynthesis	**H**
Clee_00965	**-**	PD-(D/E)XK nuclease family transposase	Transposition	**L**
Clee_00993	**-**	CDS	-	**S**
Clee_01003	**-**	CDS	-	**S**
Clee_01015	**-**	Major Facilitator Superfamily protein	General function prediction only	**R**
Clee_01022	**-**	CDS	-	**S**
Clee_01055	**-**	CDS	-	**S**
Clee_01063	**pleC**	Non-motile and phage-resistance protein	Defense mechanisms	**V**
Clee_01078	**-**	CDS	-	**S**
Clee_01081	**-**	CDS	-	**S**
Clee_01087	**-**	Ankyrin repeats (3 copies)	Ankyrin	**R**
Clee_01102	**-**	Ankyrin repeat protein	Ankyrin	**R**
Clee_01114	**-**	CDS	-	**S**
Clee_01124	**-**	CDS	-	**S**
Clee_01127	**-**	CDS	-	**S**
Clee_01137	**-**	CDS	-	**S**
Clee_01146	**-**	CDS	-	**S**
Clee_01147	**-**	Surface antigen	Defense	**R**
Clee_01149	**-**	CDS	-	**S**
Clee_01185	**-**	CDS	-	**S**
Clee_01188	**-**	CDS	-	**S**
Clee_01194	**-**	CDS	-	**S**
Clee_01196	**-**	CDS	-	**S**
Clee_01213	**-**	CDS	-	**S**
Clee_01228	**-**	CDS	-	**S**
Clee_01230	**-**	CDS	-	**S**
Clee_01239	**-**	CDS	-	**S**
Clee_01240	**-**	CDS	-	**S**
Clee_01244	**-**	CDS	-	**S**
Clee_01249	**-**	CDS	-	**S**
Clee_01256	**-**	CDS	-	**S**
Clee_01275	**-**	CDS	-	**S**
Clee_01310	**-**	CDS	-	**S**
Clee_01313	**-**	CDS	-	**S**
Clee_01317	**-**	Ankyrin repeat protein	Ankyrin	**R**
Clee_01318	**-**	CDS	-	**S**
Clee_01331	**-**	Ankyrin repeats (3 copies)	Ankyrin	**R**
Clee_01343	**-**	CDS	-	**S**
Clee_01350	**-**	CDS	-	**S**
Clee_01356	**-**	CDS	-	**S**
Clee_01371	**-**	CDS	-	**S**
Clee_01381	**-**	CDS	-	**S**
Clee_01384	**-**	CDS	-	**S**
Clee_01392	**-**	CDS	-	**S**
Clee_01394	**-**	CDS	-	**S**
Clee_01397	**-**	CDS	-	**S**
Clee_01402	**-**	CDS	-	**S**
Clee_01403	**-**	CDS	-	**S**
Clee_01404	**-**	CDS	-	**S**
Clee_01405	**-**	CDS	-	**S**
Dimm_00012	**-**	CDS	-	**S**
Dimm_00018	**-**	CDS	-	**S**
Dimm_00020	**-**	CDS	-	**S**
Dimm_00028	**-**	CDS	-	**S**
Dimm_00031	**-**	CDS	-	**S**
Dimm_00061	**-**	CDS	-	**S**
Dimm_00064	**-**	CDS	-	**S**
Dimm_00066	**ispH**	4-hydroxy-3-methylbut-2-enyl diphosphate reductase	Terpenoid backbone biosynthesis	**I**
Dimm_00075	**-**	CDS	-	**S**
Dimm_00087	**-**	CDS	-	**S**
Dimm_00088	**-**	CDS	-	**S**
Dimm_00111	**-**	CDS	-	**S**

Dimm_00113	**-**	CDS	-	**S**
Dimm_00122	**-**	CDS	-	**S**
Dimm_00154	**-**	CDS	-	**S**
Dimm_00195	**secE**	preprotein translocase subunit SecE	Bacterial secretion system	**U**
Dimm_00206	**-**	CDS	-	**S**
Dimm_00207	**-**	CDS	-	**S**
Dimm_00218	**-**	CDS	-	**S**
Dimm_00260	**-**	CDS	-	**S**
Dimm_00288	**-**	CDS	-	**S**
Dimm_00292	**-**	CDS	-	**S**
Dimm_00303	**-**	CDS	-	**S**
Dimm_00311	**-**	CDS	-	**S**
Dimm_00312	**-**	CDS	-	**S**
Dimm_00325	**-**	CDS	-	**S**
Dimm_00369	**-**	CDS	-	**S**
Dimm_00371	**-**	CDS	-	**S**
Dimm_00395	**-**	CDS	-	**S**
Dimm_00396	**-**	CDS	-	**S**
Dimm_00398	**-**	CDS	-	**S**
Dimm_00407	**-**	CDS	-	**S**
Dimm_00419	**-**	CDS	-	**S**
Dimm_00423	**-**	CDS	-	**S**
Dimm_00430	**-**	CDS	-	**S**
Dimm_00432	**-**	CDS	-	**S**
Dimm_00451	**-**	CDS	-	**S**
Dimm_00452	**-**	CDS	-	**S**
Dimm_00456	**-**	CDS	-	**S**
Dimm_00461	**-**	CDS	-	**S**
Dimm_00469	**-**	CDS	-	**S**
Dimm_00485	**-**	CDS	-	**S**
Dimm_00488	**-**	CDS	-	**S**
Dimm_00509	**-**	CDS	-	**S**
Dimm_00511	**-**	CDS	-	**S**
Dimm_00515	**-**	CDS	-	**S**
Dimm_00520	**-**	CDS	-	**S**
Dimm_00522	**-**	CDS	-	**S**
Dimm_00585	**-**	CDS	-	**S**
Dimm_00606	**-**	CDS	-	**S**
Dimm_00610	**-**	CDS	-	**S**
Dimm_00611	**-**	CDS	-	**S**
Dimm_00629	**-**	CDS	-	**S**
Dimm_00648	**exoD**	Exopolysaccharide synthesis, ExoD	General function prediction only	**R**
Dimm_00670	**-**	CDS	-	**S**
Dimm_00675	**-**	CDS	-	**S**
Dimm_00711	**-**	CDS	-	**S**
Dimm_00717	**-**	CDS	-	**S**
Dimm_00738	**-**	CDS	-	**S**
Dimm_00742	**-**	CDS	-	**S**
Dimm_00743	**priA**	primosome assembly protein PriA	Homologous recombination	**L**
Dimm_00744	**-**	CDS	-	**S**
Dimm_00746	**-**	CDS	-	**S**
Dimm_00749	**-**	CDS	-	**S**
Dimm_00757	**-**	CDS	-	**S**
Dimm_00764	**-**	CDS	-	**S**
Dimm_00772	**-**	CDS	-	**S**
Dimm_00774	**-**	CDS	-	**S**
Dimm_00791	**-**	CDS	-	**S**
Dimm_00794	**-**	CDS	-	**S**
Lsss_00025	**-**	CDS	-	**S**
Lsss_00039	**-**	CDS	-	**S**
Lsss_00044	**-**	CDS	-	**S**
Lsss_00046	**-**	CDS	-	**S**
Lsss_00060	**-**	CDS	-	**S**
Lsss_00062	**-**	CDS	-	**S**
Lsss_00075	**-**	CDS	-	**S**
Lsss_00078	**-**	CDS	-	**S**
Lsss_00081	**-**	CDS	-	**S**
Lsss_00096	**-**	CDS	-	**S**
Lsss_00103	**-**	CDS	-	**S**
Lsss_00106	**-**	CDS	-	**S**
Lsss_00107	**-**	CDS	-	**S**
Lsss_00108	**-**	CDS	-	**S**
Lsss_00127	**-**	CDS	-	**S**
Lsss_00130	**-**	CDS	-	**S**
Lsss_00193	**-**	CDS	-	**S**
Lsss_00203	**-**	CDS	-	**S**
Lsss_00210	**-**	CDS	-	**S**
Lsss_00249	**-**	CDS	-	**S**
Lsss_00250	**-**	CDS	-	**S**
Lsss_00253	**-**	CDS	-	**S**
Lsss_00254	**-**	CDS	-	**S**
Lsss_00256	**-**	CDS	-	**S**
Lsss_00276	**recG**	ATP-dependent DNA helicase RecG	Homologous recombination	**LK**
Lsss_00282	**-**	CDS	-	**S**
Lsss_00290	**-**	CDS	-	**S**
Lsss_00317	**-**	CDS	-	**S**
Lsss_00327	**-**	CDS	-	**S**
Lsss_00343	**-**	CDS	-	**S**

Lsss_00349	**-**	CDS	-	**S**
Lsss_00351	**-**	CDS	-	**S**
Lsss_00354	**-**	CDS	-	**S**
Lsss_00355	**-**	CDS	-	**S**
Lsss_00360	**-**	CDS	-	**S**
Lsss_00375	**-**	CDS	-	**S**
Lsss_00376	**-**	CDS	-	**S**
Lsss_00377	**eno**	Enolase	Biosynthesis of amino acids	**G**
Lsss_00378	**eno**	Enolase	Biosynthesis of amino acids	**G**
Lsss_00379	**eno**	Enolase	Biosynthesis of amino acids	**G**
Lsss_00380	**obg**	GTPase Obg	General function prediction only	**R**
Lsss_00381	**cgtA**	GTPase CgtA	Cell cycle control, cell division, chromosome partitioning	**D**
Lsss_00382	**-**	CDS	-	**S**
Lsss_00393	**-**	CDS	-	**S**
Lsss_00395	**-**	CDS	-	**S**
Lsss_00398	**-**	CDS	-	**S**
Lsss_00407	**-**	CDS	-	**S**
Lsss_00417	**-**	CDS	-	**S**
Lsss_00441	**-**	CDS	-	**S**
Lsss_00443	**-**	CDS	-	**S**
Lsss_00451	**-**	CDS	-	**S**
Lsss_00457	**-**	CDS	-	**S**
Lsss_00462	**-**	CDS	-	**S**
Lsss_00465	**-**	CDS	-	**S**
Lsss_00479	**-**	CDS	-	**S**
Lsss_00481	**-**	CDS	-	**S**
Lsss_00485	**-**	CDS	-	**S**
Lsss_00503	**-**	CDS	-	**S**
Lsss_00510	**-**	CDS	-	**S**
Lsss_00511	**-**	CDS	-	**S**
Lsss_00514	**-**	CDS	-	**S**
Lsss_00515	**-**	CDS	-	**S**
Lsss_00517	**-**	CDS	-	**S**
Lsss_00529	**-**	CDS	-	**S**
Lsss_00530	**-**	CDS	-	**S**
Lsss_00533	**-**	CDS	-	**S**
Lsss_00536	**-**	CDS	-	**S**
Lsss_00544	**-**	CDS	-	**S**
Lsss_00545	**-**	CDS	-	**S**
Lsss_00553	**-**	CDS	-	**S**
Lsss_00554	**-**	CDS	-	**S**
Lsss_00555	**-**	CDS	-	**S**
Lsss_00556	**-**	CDS	-	**S**
Lsss_00561	**argD**	Acetylornithine aminotransferase	Arginine biosynthesis	**E**
Lsss_00592	**-**	CDS	-	**S**
Lsss_00593	**-**	CDS	-	**S**
Lsss_00617	**-**	CDS	-	**S**
Lsss_00632	**-**	CDS	-	**S**
Lsss_00654	**-**	CDS	-	**S**
Lsss_00658	**-**	CDS	-	**S**
Lsss_00661	**-**	CDS	-	**S**
Lsss_00664	**-**	CDS	-	**S**
Lsss_00691	**-**	CDS	-	**S**
Lsss_00693	**-**	CDS	-	**S**
Lsss_00695	**-**	CDS	-	**S**
Lsss_00704	**-**	CDS	-	**S**
Lsss_00706	**-**	CDS	-	**S**
Lsss_00714	**-**	CDS	-	**S**
Lsss_00715	**-**	CDS	-	**S**
Lsss_00717	**-**	CDS	-	**S**
Lsss_00729	**-**	CDS	-	**S**
Lsss_00765	**tuf**	elongation factor Tu	Translation	**J**
Lsss_00766	**tuf**	elongation factor Tu	Translation	**J**
Lsss_00771	**-**	CDS	-	**S**
Lsss_00772	**-**	CDS	-	**S**
Lsss_00775	**-**	CDS	-	**S**
Lsss_00776	**-**	CDS	-	**S**
Lsss_00777	**-**	CDS	-	**S**
Lsss_00778	**-**	CDS	-	**S**
Lsss_00783	**-**	CDS	-	**S**
Lsss_00784	**-**	CDS	-	**S**
Lsss_00787	**-**	CDS	-	**S**
Lsss_00795	**-**	CDS	-	**S**
Lsss_00801	**-**	CDS	-	**S**
Lsss_00811	**-**	CDS	-	**S**
Lsss_00818	**-**	CDS	-	**S**
Lsss_00819	**-**	CDS	-	**S**
Lsss_00843	**-**	CDS	-	**S**
Lsss_00851	**-**	CDS	-	**S**
Lsss_00854	**-**	CDS	-	**S**
Lsss_00855	**-**	CDS	-	**S**
Lsss_00857	**-**	CDS	-	**S**
Lsss_00858	**-**	CDS	-	**S**
Lsss_00859	**-**	CDS	-	**S**
Lsss_00861	**-**	CDS	-	**S**
Lsss_00871	**-**	CDS	-	**S**
Lsss_00886	**-**	CDS	-	**S**

Lsss_00896	**-**	CDS	-	**S**
Lsss_00909	**-**	CDS	-	**S**
Lsss_00910	-	CDS	-	**S**
Lsss_00917	**hscA**	chaperone protein HscA	Posttranslational modification, protein turnover, chaperones	**O**
Lsss_00918	**hscA**	chaperone protein HscA	Posttranslational modification, protein turnover, chaperones	**O**
Oooo_00001	**-**	CDS	-	**S**
Oooo_00004	**pdxJ**	Pyridoxine 5'-phosphate synthase	Vitamin B metabolism	**H**
Oooo_00016	**-**	CDS	-	**S**
Oooo_00017	**-**	CDS	-	**S**
Oooo_00028	-	CDS	-	**S**
Oooo_00037	**-**	CDS	-	**S**
Oooo_00038	**-**	CDS	-	**S**
Oooo_00060	**-**	CDS	-	**S**
Oooo_00064	**-**	CDS	-	**S**
Oooo_00077	**glyS**	glycyl-tRNA synthetase subunit beta	Aminoacyl-tRNA biosynthesis	**J**
Oooo_00082	-	CDS	-	**S**
Oooo_00087	**-**	CDS	-	**S**
Oooo_00094	**-**	CDS	-	**S**
Oooo_00095	**-**	CDS	-	**S**
Oooo_00098	**-**	CDS	-	**S**
Oooo_00108	-	CDS	-	**S**
Oooo_00109	**-**	CDS	-	**S**
Oooo_00114	-	CDS	-	**S**
Oooo_00159	-	CDS	-	**S**
Oooo_00178	**-**	CDS	-	**S**
Oooo_00180	**-**	CDS	-	**S**
Oooo_00184	**-**	CDS	-	**S**
Oooo_00186	**-**	CDS	-	**S**
Oooo_00194	**-**	CDS	-	**S**
Oooo_00197	-	CDS	-	**S**
Oooo_00205	**-**	CDS	-	**S**
Oooo_00208	**-**	CDS	-	**S**
Oooo_00209	**-**	CDS	-	**S**
Oooo_00210	**-**	CDS	-	**S**
Oooo_00212	**-**	CDS	-	**S**
Oooo_00213	**-**	CDS	-	**S**
Oooo_00214	**-**	CDS	-	**S**
Oooo_00216	**-**	CDS	-	**S**
Oooo_00223	-	CDS	-	**S**
Oooo_00233	**-**	CDS	-	**S**
Oooo_00245	-	CDS	-	**S**
Oooo_00246	-	CDS	-	**S**
Oooo_00258	**-**	CDS	-	**S**
Oooo_00260	**-**	CDS	-	**S**
Oooo_00282	-	CDS	-	**S**
Oooo_00294	**-**	CDS	-	**S**
Oooo_00297	-	CDS	-	**S**
Oooo_00302	**-**	CDS	-	**S**
Oooo_00305	**-**	CDS	-	**S**
Oooo_00306	**-**	CDS	-	**S**
Oooo_00323	**-**	CDS	-	**S**
Oooo_00339	-	CDS	-	**S**
Oooo_00340	-	CDS	-	**S**
Oooo_00347	**-**	CDS	-	**S**
Oooo_00356	**ribF**	Riboflavin biosynthesis protein RibF	Riboflavin biosynthesis	**H**
Oooo_00363	-	CDS	-	**S**
Oooo_00368	**-**	CDS	-	**S**
Oooo_00371	**-**	CDS	-	**S**
Oooo_00387	**clpA**	ATP-dependent Clp protease ATP-binding subunit ClpA	Posttranslational modification, protein turnover, chaperones	**O**
Oooo_00388	-	CDS	-	**S**
Oooo_00389	**clpA**	ATP-dependent Clp protease ATP-binding subunit ClpA	Posttranslational modification, protein turnover, chaperones	**O**
Oooo_00390	-	CDS	-	**S**
Oooo_00391	**-**	CDS	-	**S**
Oooo_00396	**-**	CDS	-	**S**
Oooo_00398	-	CDS	-	**S**
Oooo_00403	**-**	CDS	-	**S**
Oooo_00408	**-**	CDS	-	**S**
Oooo_00410	-	CDS	-	**S**
Oooo_00411	-	CDS	-	**S**
Oooo_00439	-	CDS	-	**S**
Oooo_00451	-	CDS	-	**S**
Oooo_00461	**-**	CDS	-	**S**
Oooo_00466	**-**	CDS	-	**S**
Oooo_00470	-	CDS	-	**S**
Oooo_00476	-	CDS	-	**S**
Oooo_00478	**cutA**	Divalent-cation tolerance protein CutA	Inorganic ion transport and metabolism	**P**
Oooo_00484	-	CDS	-	**S**
Oooo_00494	-	CDS	-	**S**
Oooo_00516	-	CDS	-	**S**
Oooo_00549	-	CDS	-	**S**
Oooo_00566	**-**	CDS	-	**S**
Oooo_00569	**-**	CDS	-	**S**
Oooo_00583	-	CDS	-	**S**
Oooo_00584	**-**	CDS	-	**S**

Oooo_00588	**-**	CDS	-	**S**
Oooo_00590	**-**	CDS	-	**S**
Oooo_00591	**-**	CDS	-	**S**
Oooo_00616	**-**	CDS	-	**S**
Oooo_00620	**-**	CDS	-	**S**
Oooo_00630	**-**	CDS	-	**S**
Oooo_00660	**-**	CDS	-	**S**
Oooo_00662	**-**	CDS	-	**S**
Oooo_00663	**-**	CDS	-	**S**
Oooo_00666	**-**	CDS	-	**S**
Oooo_00673	**-**	CDS	-	**S**
Oooo_00687	**-**	CDS	-	**S**
Oooo_00689	**-**	CDS	-	**S**
Oooo_00694	**-**	CDS	-	**S**
Oooo_00701	**-**	CDS	-	**S**
Oooo_00702	**-**	CDS	-	**S**
Oooo_00711	**-**	CDS	-	**S**
Oooo_00725	**-**	CDS	-	**S**
Oooo_00750	**-**	CDS	-	**S**
Oooo_00755	**-**	CDS	-	**S**
Oooo_00757	**-**	CDS	-	**S**
Oooo_00758	**-**	CDS	-	**S**
Oooo_00765	**-**	CDS	-	**S**
Oooo_00774	**-**	CDS	-	**S**
Oooo_00827	**-**	CDS	-	**S**
Oooo_00832	**-**	CDS	-	**S**
Ovcc_00010	**purC**	Phosphoribosylaminoimidazole-succinocarboxamide synthase	Purine metabolism	**F**
Ovcc_00017	**-**	CDS	-	**S**
Ovcc_00130	**-**	CDS	-	**S**
Ovcc_00165	**-**	CDS	-	**S**
Ovcc_00255	**-**	CDS	-	**S**
Ovcc_00287	**-**	CDS	-	**S**
Ovcc_00363	**-**	CDS	-	**S**
Ovcc_00427	**-**	CDS	-	**S**
Ovcc_00496	**-**	CDS	-	**S**
Ovcc_00530	**-**	CDS	-	**S**
Ovcc_00552	**-**	CDS	-	**S**
Ovcc_00589	**-**	CDS	-	**S**
Ovcc_00664	**-**	CDS	-	**S**
Ovcc_00690	**-**	CDS	-	**S**
Ovcc_00739	**-**	CDS	-	**S**
Ovcc_00776	**-**	CDS	-	**S**

**PANGENOME Venn: 685 A+B+C+D+F+wPpe**				
**ID**	**Gene Symbol**	**Gene Product**	**Pathway or Process**	**COG**
AlbB_00001	**pyrC**	Dihydroorotase	Pyrimidine metabolism	**F**
AlbB_00006	**-**	CDS	-	**S**
AlbB_00007	**recO**	DNA repair protein RecO	Homologous recombination	**L**
AlbB_00008	**pleD**	Response regulator PleD	Signal transduction mechanisms	**T**
AlbB_00009	**-**	CDS	-	**S**
AlbB_00010	**gdh**	NAD-specific glutamate dehydrogenase	Arginine biosynthesis	**G**
AlbB_00013	**-**	CDS	-	**S**
AlbB_00017	**plsY**	putative glycerol-3-phosphate acyltransferase	Glycerolipid metabolism	**I**
AlbB_00018	**alsT**	Amino-acid carrier protein AlsT	Amino acid transport	**E**
AlbB_00020	**ynjF**	CDP-alcohol phosphatidyltransferase	General function prediction only	**R**
AlbB_00021	**-**	phosphatidylserine decarboxylase	Lipid transport and metabolism (I)	**I**
AlbB_00024	**gyrB**	DNA gyrase subunit B	Replication, recombination and repair	**L**
AlbB_00025	**gatA**	Glutamyl-tRNA(Gln) amidotransferase subunit A	Glutamate biosynthesis	**E**
AlbB_00026	**polA**	DNA polymerase I, thermostable	Purine metabolism	**L**
AlbB_00028	**-**	CDS	-	**S**
AlbB_00029	**xthA**	Exodeoxyribonuclease III	Base excision repair	**L**
AlbB_00030	**hemD**	uroporphyrinogen-III synthase	Heme biosynthesis	**H**
AlbB_00034	**algC**	Phosphomannomutase/phosphoglucomutase	Glycolysis / Gluconeogenesis	**G**
AlbB_00035	**-**	Colicin V production protein	Extracellular toxin production	**V**
AlbB_00039	**pheS**	Phenylalanine--tRNA ligase alpha subunit	Aminoacyl-tRNA biosynthesis	**J**
AlbB_00040	**glmU**	Bifunctional protein GlmU	Amino sugar and nucleotide sugar metabolism	**M**
AlbB_00045	**atpC**	ATP synthase epsilon chain	Oxidative phosphorylation	**C**
AlbB_00047	**pstA**	Phosphate transport system permease protein PstA	ABC transporters	**P**
AlbB_00052	**ubiG**	bifunctional 3-demethylubiquinone-9 3-methyltransferase/ 2-octaprenyl-6-hydroxy phenol methylase	Ubiquinone and other terpenoid-quinone biosynthesis	**H**
AlbB_00053	**nrdZ**	Ribonucleoside-diphosphate reductase NrdZ	Nucleotide transport and metabolism	**F**
AlbB_00054	**-**	Putative Holliday junction resolvase	Replication, recombination and repair	**L**
AlbB_00055	**ruvC**	Crossover junction endodeoxyribonuclease RuvC	Homologous recombination	**L**
AlbB_00056	**ctaE**	Cytochrome c oxidase subunit 3	Oxidative phosphorylation	**C**
AlbB_00059	**metK**	S-adenosylmethionine synthase	Biosynthesis of amino acids	**H**
AlbB_00061	**rpmA**	50S ribosomal protein L27	Ribosome	**J**
AlbB_00062	**rplU**	50S ribosomal protein L21	Ribosome	**J**
AlbB_00064	**tal**	putative transaldolase	Carbon metabolism	**C**
AlbB_00065	**-**	CDS	-	**S**
AlbB_00066	**carA**	Carbamoyl-phosphate synthase small chain	Arginine biosynthesis	**E**
AlbB_00069	**rpsJ**	30S ribosomal protein S10	Ribosome	**J**
AlbB_00070	**rplC**	50S ribosomal protein L3	Ribosome	**J**
AlbB_00071	**rplD**	50S ribosomal protein L4	Ribosome	**J**
AlbB_00072	**rplW**	50S ribosomal protein L23	Ribosome	**J**
AlbB_00073	**rplB**	50S ribosomal protein L2	Ribosome	**J**
AlbB_00074	**rpsS**	30S ribosomal protein S19	Ribosome	**J**
AlbB_00075	**rplV**	50S ribosomal protein L22	Ribosome	**J**

AlbB_00076	**rpsC**	30S ribosomal protein S3	Ribosome	**J**
AlbB_00077	**rplP**	50S ribosomal protein L16	Ribosome	**J**
AlbB_00079	**rpsQ**	30S ribosomal protein S17	Ribosome	**J**
AlbB_00080	**rplN**	50S ribosomal protein L14	Ribosome	**J**
AlbB_00081	**rplX**	50S ribosomal protein L24	Ribosome	**J**
AlbB_00082	**rplE**	50S ribosomal protein L5	Ribosome	**J**
AlbB_00083	**rpsN**	30S ribosomal protein S14	Ribosome	**J**
AlbB_00084	**rpsH**	30S ribosomal protein S8	Ribosome	**J**
AlbB_00085	**rplF**	50S ribosomal protein L6	Ribosome	**J**
AlbB_00086	**rplR**	50S ribosomal protein L18	Ribosome	**J**
AlbB_00087	**rpsE**	30S ribosomal protein S5	Ribosome	**J**
AlbB_00088	**rplO**	50S ribosomal protein L15	Ribosome	**J**
AlbB_00089	**secY**	preprotein translocase subunit SecY	Bacterial secretion system	**U**
AlbB_00090	**adk**	adenylate kinase	Purine metabolism	**F**
AlbB_00091	**rpsM**	30S ribosomal protein S13	Ribosome	**J**
AlbB_00092	**rpsK**	30S ribosomal protein S11	Ribosome	**J**
AlbB_00093	**rpoA**	DNA-directed RNA polymerase subunit alpha	Purine metabolism	**K**
AlbB_00094	**rplQ**	50S ribosomal protein L17	Ribosome	**J**
AlbB_00096	**atpH**	ATP synthase subunit delta	Oxidative phosphorylation	**C**
AlbB_00097	**atpA**	ATP synthase subunit alpha	Oxidative phosphorylation	**C**
AlbB_00098	**greA**	Transcription elongation factor GreA	Transcription	**K**
AlbB_00099	**ribB**	3,4-dihydroxy-2-butanone 4-phosphate synthase	Riboflavin biosynthesis	**H**
AlbB_00100	**yfgC**	TPR repeat-containing protein YfgC precursor	General function prediction only	**R**
AlbB_00101	**-**	TrbC/VIRB2 family protein	Bacterial secretion system	**U**
AlbB_00102	**fabG**	3-oxoacyl-[acyl-carrier-protein] reductase FabG	Biosynthesis of unsaturated fatty acids	**I**
AlbB_00120	**ppdK**	Pyruvate, phosphate dikinase	Carbon metabolism	**C**
AlbB_00122	**-**	SPFH domain / Band 7 family protein	General function prediction only	**R**
AlbB_00124	**-**	putative transcriptional regulatory protein	General function prediction only	**R**
AlbB_00125	**bamA**	Outer membrane protein assembly factor BamA precursor	Membrane assembly	**M**
AlbB_00126	**-**	CDS	-	**S**
AlbB_00127	**pgsA**	CDP-diacylglycerol--glycerol-3-phosphate 3-phosphatidyltransferase	Glycerophospholipid metabolism	**I**
AlbB_00128	**maeB**	NADP-dependent malic enzyme	Carbon metabolism	**C**
AlbB_00130	**grxD**	Glutaredoxin-4	Posttranslational modification, protein turnover, chaperones	**O**
AlbB_00133	**-**	PD-(D/E)XK nuclease family transposase	Transposition	**L**
AlbB_00135	**bcr**	Bicyclomycin resistance protein	Amino acid transport and metabolism	**E**
AlbB_00136	**ubiE**	Ubiquinone/menaquinone biosynthesis C-methyltransferase UbiE	Ubiquinone and other terpenoid-quinone biosynthesis	**H**
AlbB_00137	-	Putative multidrug export ATP-binding/permease protein	Bacterial secretion system	**U**
AlbB_00140	**argS**	Arginine--tRNA ligase	Aminoacyl-tRNA biosynthesis	**J**
AlbB_00141	**ccmA**	Cytochrome c biogenesis ATP-binding export protein CcmA	ABC transporters	**V**
AlbB_00142	**-**	CDS	-	**S**
AlbB_00143	**tadA**	tRNA-specific adenosine deaminase	Nucleotide transport and metabolism	**F**
AlbB_00146	**pyrG**	CTP synthase	Pyrimidine metabolism	**F**
AlbB_00147	**secG**	preprotein translocase subunit SecG	Bacterial secretion system	**U**
AlbB_00152	**glmS**	Glutamine--fructose-6-phosphate aminotransferase [isomerizing]	Alanine, aspartate and glutamate metabolism	**M**
AlbB_00153	**yajC**	preprotein translocase subunit YajC	Bacterial secretion system	**U**
AlbB_00155	**rpsB**	30S ribosomal protein S2	Ribosome	**J**
AlbB_00156	**tsf**	Elongation factor Ts	Translation	**J**
AlbB_00157	**pyrH**	Uridylate kinase	Pyrimidine metabolism	**F**
AlbB_00158	**frr**	Ribosome-recycling factor	Translation, ribosomal structure and biogenesis	**J**
AlbB_00159	**pstS**	Phosphate-binding protein PstS precursor	ABC transporters	**P**
AlbB_00162	**dcd**	Deoxycytidine triphosphate deaminase	Pyrimidine metabolism	**F**
AlbB_00165	**-**	Calcineurin-like phosphoesterase	General function prediction only	**R**
AlbB_00167	**virB8**	Type IV secretion system protein virB8	Bacterial secretion system	**U**
AlbB_00168	**ptlF**	Type IV secretion system protein PtlF precursor	Bacterial secretion system	**U**
AlbB_00169	**virB10**	Type IV secretion system protein virB10	Bacterial secretion system	**U**
AlbB_00170	**virB11**	Type IV secretion system protein VirB11	Bacterial secretion system	**U**
AlbB_00171	**traG**	Conjugal transfer protein TraG	Replication, recombination and repair	**L**
AlbB_00172	**-**	Surface antigen	Defense	**R**
AlbB_00175	**proP**	Proline/betaine transporter	Transporter/osmoregulator	**GEP**
AlbB_00176	**-**	CDS	-	**S**
AlbB_00181	**ftsY**	Signal recognition particle receptor FtsY	Bacterial secretion system	**U**
AlbB_00182	**nifU**	NifU-like protein	Energy production and conversion	**C**
AlbB_00184	**-**	CDS	-	**S**
AlbB_00185	**hscA**	chaperone protein HscA	Chaperone	**O**
AlbB_00186	**lepB**	Signal peptidase I	Protein export	**U**
AlbB_00188	**bfmBAB**	2-oxoisovalerate dehydrogenase subunit beta	Valine, leucine and isoleucine degradation	**E**
AlbB_00191	**gntX**	DNA utilization protein GntX	Nucleoside catabolic process	**O**
AlbB_00192	**ankX**	Phosphocholine transferase AnkX	Lipopolysaccharide biosynthesis	**I**
AlbB_00205	**lipA**	Lipoyl synthase	Lipoate biosynthesis	**H**
AlbB_00206	**rpmB**	50S ribosomal protein L28	Ribosome	**J**
AlbB_00207	**-**	CDS	-	**S**
AlbB_00208	**-**	CDS	-	**S**
AlbB_00210	**rpsD**	30S ribosomal protein S4	Ribosome	**J**
AlbB_00211	**tktA**	Transketolase 1	Biosynthesis of amino acids	**G**
AlbB_00212	**prs**	Ribose-phosphate pyrophosphokinase	Pentose phosphate pathway	**F**
AlbB_00213	**gatA**	Glutamyl-tRNA(Gln) amidotransferase subunit A	Glutamate biosynthesis	**E**
AlbB_00214	**tolB**	translocation protein TolB	Translation	**M**
AlbB_00215	**rnj1**	Ribonuclease J 1	Replication, recombination and repair	**L**
AlbB_00216	**dnaJ**	chaperone protein DnaJ	Chaperone	**O**
AlbB_00218	**-**	CDS	-	**S**
AlbB_00219	-	phosphoribosylformylglycinamidine synthase II	Purine metabolism	**F**
AlbB_00222	**fumC**	Fumarate hydratase class II	Carbon metabolism	**C**

AlbB_00225	**gph**	Phosphoglycolate phosphatase	Glyoxylate and dicarboxylate metabolism	**R**
AlbB_00226	**-**	RDD family protein	-	**R**
AlbB_00227	**bfr**	bacterioferritin	Porphyrin metabolism	**P**
AlbB_00228	**-**	SURF1 family protein	General function prediction only	**R**
AlbB_00229	**murC**	UDP-N-acetylmuramate--L-alanine ligase	Peptidoglycan biosynthesis	**M**
AlbB_00231	**eno**	Enolase	Biosynthesis of amino acids	**G**
AlbB_00232	**obg**	GTPase Obg	General function prediction only	**R**
AlbB_00234	**ftsK**	DNA translocase FtsK	Cell cycle control, cell division, chromosome partitioning	**D**
AlbB_00235	**-**	CDS	-	**S**
AlbB_00236	**ispG**	4-hydroxy-3-methylbut-2-en-1-yl diphosphate synthase	Terpenoid backbone biosynthesis	**I**
AlbB_00237	**-**	CDS	-	**S**
AlbB_00243	**mdtM**	Multidrug resistance protein MdtM	Posttranslational modification, protein turnover, chaperones	**O**
AlbB_00245	**-**	Membrane transport protein	Intracellular trafficking, secretion, and vesicular transport	**U**
AlbB_00249	**engB**	putative GTP-binding protein EngB	Cell cycle control, cell division, chromosome partitioning	**D**
AlbB_00250	**argB**	Acetylglutamate kinase	Arginine biosynthesis	**E**
AlbB_00252	**purA**	Adenylosuccinate synthetase	Alanine, aspartate and glutamate metabolism	**F**
AlbB_00257	**alsT**	Amino-acid carrier protein AlsT	Amino acid transport	**E**
AlbB_00264	**rluC**	Ribosomal large subunit pseudouridine synthase C	Translation, ribosomal structure and biogenesis	**J**
AlbB_00265	**proP**	Proline/betaine transporter	Mulitple COGs	**GEP**
AlbB_00268	**aspS**	Aspartate--tRNA ligase	Aminoacyl-tRNA biosynthesis	**J**
AlbB_00272	**tpiA**	Triosephosphate isomerase	Biosynthesis of amino acids	**G**
AlbB_00274	**ccmE**	Cytochrome c-type biogenesis protein CcmE	Oxidative phosphorylation	**O**
AlbB_00275	**-**	CDS	-	**S**
AlbB_00276	**birA**	Bifunctional ligase/repressor BirA	Biotin metabolism	**H**
AlbB_00277	**nuoN**	NADH-quinone oxidoreductase subunit N	Oxidative phosphorylation	**C**
AlbB_00278	**ndhD1**	NAD(P)H-quinone oxidoreductase chain 4 1	Carbohydrate transport and metabolism	**G**
AlbB_00279	**nuoL**	NADH-quinone oxidoreductase subunit L	Oxidative phosphorylation	**CP**
AlbB_00280	**nuoK**	NADH-quinone oxidoreductase subunit K	Oxidative phosphorylation	**C**
AlbB_00281	**nuoJ**	NADH-quinone oxidoreductase subunit J	Oxidative phosphorylation	**C**
AlbB_00283	**pcrA**	ATP-dependent DNA helicase PcrA	Replication	**L**
AlbB_00284	**rsbQ**	Sigma factor SigB regulation protein RsbQ	Transcription	**K**
AlbB_00286	**ruvA**	Holliday junction ATP-dependent DNA helicase RuvA	Homologous recombination	**L**
AlbB_00287	**ruvA**	Holliday junction ATP-dependent DNA helicase RuvA	Homologous recombination	**L**
AlbB_00290	**-**	recombinase A	Recombination	**L**
AlbB_00292	**sspB**	ClpXP protease specificity-enhancing factor	General function prediction only	**R**
AlbB_00297	**der**	GTPase Der	Translation, ribosomal structure and biogenesis	**J**
AlbB_00302	**gltA**	Citrate synthase	Biosynthesis of amino acids	**C**
AlbB_00303	**rpsT**	30S ribosomal protein S20	Ribosome	**J**
AlbB_00304	**yihG**	putative acyltransferase YihG	Glycosylphosphatidylinositol(GPI)-anchor biosynthesis	**R**
AlbB_00305	**xerD**	Tyrosine recombinase XerD	Recombination	**L**
AlbB_00306	**-**	CDS	-	**S**
AlbB_00307	**hda**	DnaA regulatory inactivator Hda	Replication	**O**
AlbB_00310	**trpS**	Tryptophan--tRNA ligase	Aminoacyl-tRNA biosynthesis	**J**
AlbB_00315	**-**	CDS	-	**S**
AlbB_00318	**elbB**	Enhancing lycopene biosynthesis protein 2	Secondary metabolites biosynthesis, transport and catabolism	**Q**
AlbB_00319	**-**	CDS	-	**S**
AlbB_00320	**argD**	Acetylornithine aminotransferase	Arginine biosynthesis	**E**
AlbB_00321	**pdhS**	Cell-division control histidine kinase PdhS	Cell division	**C**
AlbB_00322	**-**	CDS	-	**S**
AlbB_00323	**tsaD**	tRNA N6-adenosine threonylcarbamoyltransferase	Translation	**J**
AlbB_00325	**prsD**	Type I secretion system ATP-binding protein PrsD	Bacterial secretion system	**U**
AlbB_00341	**pdhC**	Dihydrolipoyllysine-residue acetyltransferase component of pyruvate dehydrogenase complex	Citrate cycle (TCA cycle)	**C**
AlbB_00350	**rnd**	ribonuclease D	Translation, ribosomal structure and biogenesis	**J**
AlbB_00351	**coaE**	Dephospho-CoA kinase	Pantothenate and CoA biosynthesis	**H**
AlbB_00354	**ftsH**	ATP-dependent zinc metalloprotease FtsH	Protein modification	**O**
AlbB_00357	**tilS**	tRNA(Ile)-lysidine synthase	Purine metabolism	**D**
AlbB_00358	**pal**	Outer membrane protein P6 precursor	Cell wall/membrane/envelope biogenesis	**M**
AlbB_00359	**cpsA1**	Thermostable carboxypeptidase 1	General function prediction only	**R**
AlbB_00360	**mreB**	Rod shape-determining protein MreB	Cell cycle control, cell division, chromosome partitioning	**D**
AlbB_00361	**mnmA**	tRNA-specific 2-thiouridylase MnmA	Sulfur relay system	**H**
AlbB_00363	**ppa**	Inorganic pyrophosphatase	Oxidative phosphorylation	**C**
AlbB_00365	**-**	peptidylprolyl isomerase	General function prediction only	**R**
AlbB_00366	**-**	putative bifunctional glutamate synthase subunit beta/2-polyprenylphenol hydroxylase	Amino acid transport and metabolism	**E**
AlbB_00367	**-**	putative 5-formyltetrahydrofolate cyclo-ligase	One carbon pool by folate	**R**
AlbB_00369	**-**	putative disulfide oxidoreductase	General function prediction only	**R**
AlbB_00370	**lpd**	Dihydrolipoyl dehydrogenase	Carbon metabolism	**C**
AlbB_00372	**murG**	UDP-N-acetylglucosamine--N-acetylmuramyl-(pentapeptide) pyrophosphoryl-undecaprenol N-acetylglucosamine transferase	Peptidoglycan biosynthesis	**M**
AlbB_00374	**bcp**	Putative peroxiredoxin bcp	Phenylpropanoid biosynthesis	**O**
AlbB_00375	**recF**	DNA replication and repair protein RecF	Homologous recombination	**L**
AlbB_00377	**kefC**	Glutathione-regulated potassium-efflux system protein KefC	Posttranslational modification, protein turnover, chaperones	**O**
AlbB_00378	**resA**	Thiol-disulfide oxidoreductase ResA	Replication, recombination and repair	**L**

AlbB_00379	**tmk**	Thymidylate kinase	Pyrimidine metabolism	**F**
AlbB_00382	**-**	ComEC family competence protein	Replication, recombination and repair	**L**
AlbB_00383	**-**	CDS	-	**S**
AlbB_00384	**rluC**	Ribosomal large subunit pseudouridine synthase C	Translation, ribosomal structure and biogenesis	**J**
AlbB_00386	**-**	RDD family protein	-	**R**
AlbB_00389	**mrpA**	Na(+)/H(+) antiporter subunit A	Inorganic ion transport and metabolism	**P**
AlbB_00390	**-**	VirB8 protein	Bacterial secretion system	**U**
AlbB_00392	**dnaX**	DNA polymerase III subunit tau	Purine metabolism	**L**
AlbB_00393	**tsaC**	Threonylcarbamoyl-AMP synthase	Translation	**J**
AlbB_00394	**-**	CDS	-	**S**
AlbB_00395	**miaA**	tRNA dimethylallyltransferase	Zeatin biosynthesis	**J**
AlbB_00397	**murE**	UDP-N-acetylmuramoyl-L-alanyl-D-glutamate--2,6-diaminopimelate ligase	Lysine biosynthesis	**M**
AlbB_00398	**metC**	Cystathionine beta-lyase MetC	Methionine biosynthesis	**E**
AlbB_00399	**bolA**	transcriptional regulator BolA	Transcription	**T**
AlbB_00400	**dnaK**	Chaperone protein DnaK	RNA degradation	**O**
AlbB_00401	**rne**	Ribonuclease E	RNA degradation	**J**
AlbB_00402	**rpmG2**	50S ribosomal protein L33 2	Translation, ribosomal structure and biogenesis	**J**
AlbB_00404	**znuA**	High-affinity zinc uptake system protein ZnuA precursor	ABC transporters	**P**
AlbB_00405	**znuC**	Zinc import ATP-binding protein ZnuC	ABC transporters	**P**
AlbB_00406	**ubiB**	putative protein kinase UbiB	Glycosylphosphatidylinositol(GPI)-anchor biosynthesis	**HC**
AlbB_00408	**carB**	Carbamoyl-phosphate synthase large chain	Arginine biosynthesis	**E**
AlbB_00409	**yciK**	putative oxidoreductase YciK	Energy production and conversion	**C**
AlbB_00410	**-**	CDS	-	**S**
AlbB_00413	**purF**	Amidophosphoribosyltransferase precursor	Alanine, aspartate and glutamate metabolism	**F**
AlbB_00415	**coq7**	2-nonaprenyl-3-methyl-6-methoxy-1,4-benzoquinol hydroxylase	Aminobenzoate degradation	
AlbB_00416	**-**	Uracil DNA glycosylase superfamily protein	General function prediction only	**R**
AlbB_00417	**def**	Peptide deformylase	Protein modification	**J**
AlbB_00418	**ftsA**	Cell division protein FtsA	Cell division	**D**
AlbB_00419	**map**	Methionine aminopeptidase	Translation, ribosomal structure and biogenesis	**J**
AlbB_00421	**rsmA**	Ribosomal RNA small subunit methyltransferase A	Translation, ribosomal structure and biogenesis	**J**
AlbB_00422	**-**	CDS	-	**S**
AlbB_00423	**rplY**	50S ribosomal protein L25	Ribosome	**J**
AlbB_00424	**pth**	Peptidyl-tRNA hydrolase	Aminoacyl-tRNA hydrolase	**J**
AlbB_00425	**truA**	tRNA pseudouridine synthase A	Translation, ribosomal structure and biogenesis	**J**
AlbB_00426	**dnaN**	DNA polymerase III subunit beta	Purine metabolism	**L**
AlbB_00427	**-**	CDS	-	**S**
AlbB_00428	**rpoH**	RNA polymerase sigma factor RpoH	Transcription	**K**
AlbB_00431	**dnaX**	DNA polymerase III subunit tau	Purine metabolism	**L**
AlbB_00434	**-**	Putative O-methyltransferase/MSMEI 4947	General function prediction only	**R**
AlbB_00436	**bdbD**	Disulfide bond formation protein D precursor	General function prediction only	**R**
AlbB_00437	**pasT**	Persistence and stress-resistance toxin PasT	Stress tolerance	**V**
AlbB_00438	**ttg2**	Toluene tolerance, Ttg2	ABC transporters	**P**
AlbB_00439	**fgs**	Folylpolyglutamate synthase	Folate biosynthesis	**C**
AlbB_00441	**murD**	UDP-N-acetylmuramoylalanine--D-glutamate ligase	Peptidoglycan biosynthesis	**M**
AlbB_00442	**-**	CDS	-	**S**
AlbB_00445	**nth**	Endonuclease III	Base excision repair	**L**
AlbB_00446	**purH**	Bifunctional purine biosynthesis protein PurH	Purine metabolism	**F**
AlbB_00447	**fmt**	Methionyl-tRNA formyltransferase	Aminoacyl-tRNA biosynthesis	**J**
AlbB_00448	**rplT**	50S ribosomal protein L20	Ribosome	**J**
AlbB_00449	**rpmI**	50S ribosomal protein L35	Ribosome	**J**
AlbB_00450	**-**	PAS domain protein	General function prediction only	**R**
AlbB_00453	**alaS**	Alanine--tRNA ligase	Aminoacyl-tRNA biosynthesis	**J**
AlbB_00456	**gpmI**	2,3-bisphosphoglycerate-independent phosphoglycerate mutase	Glycolysis / Gluconeogenesis	**G**
AlbB_00458	**fabD**	Malonyl CoA-acyl carrier protein transacylase	Fatty acid biosynthesis	**I**
AlbB_00459	**rpmE**	50S ribosomal protein L31	Ribosome	**J**
AlbB_00460	**ppnK**	putative inorganic polyphosphate/ATP-NAD kinase	Nicotinate and nicotinamide metabolism	**R**
AlbB_00461	**-**	CDS	-	**S**
AlbB_00462	**idiA**	Iron deficiency-induced protein A precursor	Iron metabolism	**P**
AlbB_00463	**pyrB**	Aspartate carbamoyltransferase	Alanine, aspartate and glutamate metabolism	**F**
AlbB_00464	**-**	Ankyrin repeats (3 copies)	Ankyrin	**R**
AlbB_00465	**pipB2**	Secreted effector protein pipB2	Secondary metabolites biosynthesis, transport and catabolism	**Q**
AlbB_00466	**gmk**	Guanylate kinase	Purine metabolism	**F**
AlbB_00468	**sdhA**	Succinate dehydrogenase flavoprotein subunit	Butanoate metabolism	**C**
AlbB_00469	**-**	CDS	-	**S**
AlbB_00470	**dapD**	2,3,4,5-tetrahydropyridine-2,6-dicarboxylate N-succinyltransferase	Lysine biosynthesis	**E**
AlbB_00471	**-**	CDS	-	**S**
AlbB_00472	**-**	CDS	-	**S**
AlbB_00474	**ankX**	Phosphocholine transferase AnkX	Lipopolysaccharide biosynthesis	**I**
AlbB_00478	**gapB**	Glyceraldehyde-3-phosphate dehydrogenase 2	Glycolysis / Gluconeogenesis	**E**
AlbB_00479	**tatA**	Sec-independent protein translocase protein TatA	Bacterial secretion system	**U**
AlbB_00486	**cca**	CCA-adding enzyme	RNA transport	**J**
AlbB_00487	**typA**	GTP-binding protein TypA/BipA	Signal transduction mechanisms	**T**
AlbB_00488	**gltX1**	Glutamate--tRNA ligase 1	Translation, ribosomal structure and biogenesis	**J**
AlbB_00489	**holA**	DNA polymerase III subunit delta	Purine metabolism	**L**
AlbB_00491	**znuB**	High-affinity zinc uptake system membrane protein ZnuB	ABC transporters	**P**

AlbB_00493	**ispE**	4-diphosphocytidyl-2-C-methyl-D-erythritol kinase	Terpenoid backbone biosynthesis	**I**
AlbB_00495	**ccmH**	Cytochrome c-type biogenesis protein CcmH precursor	Oxidative phosphorylation	**O**
AlbB_00496	-	CDS	-	**S**
AlbB_00497	**thcC**	Rhodocoxin	Inorganic ion transport and metabolism	**P**
AlbB_00501	**dnaG**	DNA primase	DNA replication	**L**
AlbB_00502	**ubiG**	Ubiquinone biosynthesis O-methyltransferase	Ubiquinone and other terpenoid-quinone biosynthesis	**H**
AlbB_00503	**-**	Sodium:dicarboxylate symporter family protein	Carbohydrate transport and metabolism	**G**
AlbB_00504	**tsaB**	tRNA threonylcarbamoyladenosine biosynthesis protein TsaB	Translation, ribosomal structure and biogenesis	**J**
AlbB_00507	**ccmB**	CcmB protein	ABC transporters	**O**
AlbB_00508	**dksA**	RNA polymerase-binding transcription factor DksA	Signal transduction mechanisms	**T**
AlbB_00512	**-**	RlpA-like protein precursor	General function prediction only	**R**
AlbB_00514	**ubiI**	2-octaprenylphenol hydroxylase	Coenzyme transport and metabolism	**H**
AlbB_00515	**-**	CDS	-	**S**
AlbB_00517	**coaD**	Phosphopantetheine adenylyltransferase	Pantothenate and CoA biosynthesis	**H**
AlbB_00518	-	CDS	-	**S**
AlbB_00519	**cdsA**	Phosphatidate cytidylyltransferase	Glycerophospholipid metabolism	**I**
AlbB_00520	**ispU**	Ditrans,polycis-undecaprenyl-diphosphate synthase ((2E,6E)-farnesyl-diphosphate specific)	Terpenoid backbone biosynthesis	**I**
AlbB_00522	**sucB**	Dihydrolipoyllysine-residue succinyltransferase component of 2-oxoglutarate dehydrogenase complex	Carbon metabolism	**C**
AlbB_00524	**fabI**	Enoyl-[acyl-carrier-protein] reductase [NADH] FabI	Fatty acid biosynthesis	**I**
AlbB_00525	**-**	CDS	-	**S**
AlbB_00527	**ychF**	Ribosome-binding ATPase YchF	Translation, ribosomal structure and biogenesis	**J**
AlbB_00530	**glpX**	Fructose-1,6-bisphosphatase class 2	Carbon metabolism	**G**
AlbB_00533	**fbpC**	Fe(3+) ions import ATP-binding protein FbpC	ABC transporters	**P**
AlbB_00534	-	twin arginine translocase protein A	Amino acid transport and metabolism	**E**
AlbB_00536	**exoD**	Exopolysaccharide synthesis, ExoD	General function prediction only	**R**
AlbB_00537	**cysS**	Cysteine--tRNA ligase	Aminoacyl-tRNA biosynthesis	**J**
AlbB_00539	-	CDS	-	**S**
AlbB_00540	**rnhA**	Ribonuclease HI	DNA replication	**L**
AlbB_00541	**ctaB**	Protoheme IX farnesyltransferase	Chlorocyclohexane and chlorobenzene degradation	**O**
AlbB_00542	**ctaD**	Cytochrome c oxidase subunit 1	Oxidative phosphorylation	**C**
AlbB_00543	**ctaC**	Cytochrome c oxidase subunit 2 precursor	Oxidative phosphorylation	**C**
AlbB_00545	-	CDS	-	**S**
AlbB_00546	-	Acetoin:2,6-dichlorophenolindophenol oxidoreductase	Ketone degradation	**C**
AlbB_00547	**gatB**	Aspartyl/glutamyl-tRNA(Asn/Gln) amidotransferase subunit B	Glutamate biosynthesis	**J**
AlbB_00549	**xerD**	Tyrosine recombinase XerD	Recombination	**L**
AlbB_00550	**lpd3**	Dihydrolipoyl dehydrogenase 3	Energy production and conversion	**C**
AlbB_00559	**pyrF**	Orotidine 5'-phosphate decarboxylase	Pyrimidine metabolism	**F**
AlbB_00563	**dapB**	4-hydroxy-tetrahydrodipicolinate reductase	Lysine biosynthesis	**E**
AlbB_00564	**pstB**	Phosphate import ATP-binding protein PstB	ABC transporters	**P**
AlbB_00565	-	Ankyrin repeats (3 copies)	Ankyrin	**R**
AlbB_00566	**dxr**	1-deoxy-D-xylulose 5-phosphate reductoisomerase	Terpenoid backbone biosynthesis	**I**
AlbB_00567	-	CDS	-	**S**
AlbB_00570	**acpS**	Holo-[acyl-carrier-protein] synthase	Pantothenate and CoA biosynthesis	**I**
AlbB_00571	**-**	CDS	-	**S**
AlbB_00577	**-**	Nitronate monooxygenase	General function prediction only	**R**
AlbB_00579	**nrdB**	Ribonucleoside-diphosphate reductase subunit beta	Purine metabolism	**F**
AlbB_00580	**bamE**	outer membrane biogenesis protein BamE	Membrane assembly	**M**
AlbB_00582	**metG**	Methionine--tRNA ligase	Selenocompound metabolism	**J**
AlbB_00583	**dapE**	Succinyl-diaminopimelate desuccinylase	Lysine biosynthesis	**E**
AlbB_00584	**egsA**	Glycerol-1-phosphate dehydrogenase [NAD(P)+]	Glycerophospholipid metabolism	**C**
AlbB_00600	**nuoF**	NADH-quinone oxidoreductase subunit F	Oxidative phosphorylation	**C**
AlbB_00601	**infC**	Translation initiation factor IF-3	Translation	**J**
AlbB_00602	**thrS**	Threonine--tRNA ligase	Aminoacyl-tRNA biosynthesis	**J**
AlbB_00603	**nuoI**	NADH-quinone oxidoreductase subunit I	Oxidative phosphorylation	**C**
AlbB_00604	**mnmE**	tRNA modification GTPase MnmE	Homologous recombination	**J**
AlbB_00606	**fpr**	Ferredoxin--NADP reductase	Energy production and conversion	**C**
AlbB_00608	**mraY**	Phospho-N-acetylmuramoyl-pentapeptide-transferase	Peptidoglycan biosynthesis	**M**
AlbB_00609	**rnhB**	Ribonuclease HII	DNA replication	**L**
AlbB_00610	**ptlF**	Type IV secretion system protein PtlF precursor	Bacterial secretion system	**U**
AlbB_00612	-	CDS	-	**S**
AlbB_00613	**purM**	Phosphoribosylformylglycinamidine cyclo-ligase	Purine metabolism	**F**
AlbB_00614	**purC**	Phosphoribosylaminoimidazole-succinocarboxamide synthase	Purine metabolism	**F**
AlbB_00615	**trmD**	tRNA (guanine-N(1)-)-methyltransferase	Translation, ribosomal structure and biogenesis	**J**
AlbB_00617	**pstA**	Phosphate transport system permease protein PstA	ABC transporters	**P**
AlbB_00618	-	phosphoribosylformylglycinamidine synthase II	Purine metabolism	**F**
AlbB_00623	**pdxH**	Pyridoxine/pyridoxamine 5'-phosphate oxidase	Pyridoxal-5'-phosphate biosynthesis	**C**
AlbB_00625	**minD**	Septum site-determining protein MinD	Cell cycle control, cell division, chromosome partitioning	**D**
AlbB_00627	**mlaD**	putative phospholipid ABC transporter-binding protein MlaD	ABC transporters	**O**
AlbB_00628	**-**	NADH dehydrogenase	Energy production and conversion	**C**
AlbB_00629	**mrpD**	Na(+)/H(+) antiporter subunit D	Inorganic ion transport and metabolism	**P**
AlbB_00630	**mrdB**	Rod shape-determining protein RodA	Cell cycle control, cell division, chromosome partitioning	**D**
AlbB_00631	**iscS**	Cysteine desulfurase	Alanine biosynthesis	**E**
AlbB_00636	**mutL**	DNA mismatch repair protein MutL	Mismatch repair	**L**
AlbB_00653	**tldD**	protease TldD	Metalloprotease/peptide lysis	**R**
AlbB_00657	**petA**	Ubiquinol-cytochrome c reductase iron-sulfur subunit	Oxidative phosphorylation	
AlbB_00658	**gyrA**	DNA gyrase subunit A	Replication, recombination and repair	**L**
AlbB_00660	**ycf3**	photosystem I assembly protein Ycf3	Energy production and conversion	**C**
AlbB_00661	-	CDS	-	**S**
AlbB_00662	**bioY**	Biotin transporter BioY	Cofactor transport	**H**

AlbB_00666	**tlyC**	Hemolysin C	General function prediction only	**R**
AlbB_00667	**hemE**	Uroporphyrinogen decarboxylase	Heme biosynthesis	**H**
AlbB_00668	**aspC**	Aspartate aminotransferase	Asparagine biosynthesis	**E**
AlbB_00670	**-**	CDS	-	**S**
AlbB_00673	**glyA**	Serine hydroxymethyltransferase	Glycine biosynthesis	**HE**
AlbB_00674	**pgk**	Phosphoglycerate kinase	Biosynthesis of amino acids	**G**
AlbB_00675	**-**	CDS	-	**S**
AlbB_00677	**rpe**	Ribulose-phosphate 3-epimerase	Biosynthesis of amino acids	**G**
AlbB_00678	**pnp**	Polyribonucleotide nucleotidyltransferase	Purine metabolism	**F**
AlbB_00679	**rpsO**	30S ribosomal protein S15	Ribosome	**J**
AlbB_00680	**truB**	tRNA pseudouridine synthase B	Translation, ribosomal structure and biogenesis	**J**
AlbB_00682	**dgt**	deoxyguanosinetriphosphate triphosphohydrolase-like protein	Purine metabolism	**F**
AlbB_00683	**erpA**	Iron-sulfur cluster insertion protein ErpA	Iron metabolism	**P**
AlbB_00684	**lolD**	Lipoprotein-releasing system ATP-binding protein LolD	Two-component system	**O**
AlbB_00685	**-**	CDS	-	**S**
AlbB_00686	**-**	Alpha/beta hydrolase family protein	-	**R**
AlbB_00689	**ybhL**	Inner membrane protein YbhL	Intracellular trafficking, secretion, and vesicular transport	**U**
AlbB_00690	**ctaG**	Cytochrome c oxidase assembly protein CtaG	Oxidative phosphorylation	**C**
AlbB_00693	**qorA**	Quinone oxidoreductase 1	Energy production and conversion	**C**
AlbB_00694	**ispH**	4-hydroxy-3-methylbut-2-enyl diphosphate reductase	Terpenoid backbone biosynthesis	**I**
AlbB_00695	**-**	CDS	-	**S**
AlbB_00696	**-**	CDS	-	**S**
AlbB_00697	**-**	CDS	-	**S**
AlbB_00699	**ssb**	Single-stranded DNA-binding protein	DNA replication	**L**
AlbB_00700	**dapA**	4-hydroxy-tetrahydrodipicolinate synthase	Lysine biosynthesis	**EM**
AlbB_00702	**trxB**	Thioredoxin reductase	Pyrimidine metabolism	**O**
AlbB_00703	**tsaA**	putative peroxiredoxin	Translation, ribosomal structure and biogenesis	**J**
AlbB_00705	**accD5**	putative propionyl-CoA carboxylase beta chain 5	Fatty acid biosynthesis	**I**
AlbB_00709	**tyrS**	Tyrosine--tRNA ligase	Aminoacyl-tRNA biosynthesis	**J**
AlbB_00711	**rsfS**	Ribosomal silencing factor RsfS	Translation, ribosomal structure and biogenesis	**J**
AlbB_00715	**rppH**	RNA pyrophosphohydrolase	Carbapenem biosynthesis	**J**
AlbB_00716	**ycfH**	putative deoxyribonuclease YcfH	Fanconi anemia pathway	**L**
AlbB_00717	**mdh**	Malate dehydrogenase	Carbon metabolism	**C**
AlbB_00718	**nuoC1**	NADH-quinone oxidoreductase subunit C 1	Energy production and conversion	**C**
AlbB_00719	**nuoB**	NADH-quinone oxidoreductase subunit B	Oxidative phosphorylation	**C**
AlbB_00720	**ndhC**	NAD(P)H-quinone oxidoreductase subunit 3	Carbohydrate transport and metabolism	**G**
AlbB_00721	**-**	HIT-like protein	Base excision repair	**R**
AlbB_00723	**-**	CDS	-	**S**
AlbB_00724	**murF**	UDP-N-acetylmuramoyl-tripeptide--D-alanyl-D-alanine ligase	Lysine biosynthesis	**M**
AlbB_00727	**fabF**	3-oxoacyl-[acyl-carrier-protein] synthase 2	Fatty acid biosynthesis	**I**
AlbB_00728	**plsX**	Phosphate acyltransferase	Glycerolipid metabolism	**I**
AlbB_00731	**yxeO**	putative amino-acid import ATP-binding protein YxeO	Amino acid transport and metabolism	**E**
AlbB_00732	**ffh**	Signal recognition particle protein	Bacterial secretion system	**U**
AlbB_00733	**hyfB**	Hydrogenase-4 component B	Mulitple COGs	**CP**
AlbB_00735	**gltB**	Glutamate synthase [NADPH] small chain	Biosynthesis of amino acids	**E**
AlbB_00736	**fabZ**	3-hydroxyacyl-[acyl-carrier-protein] dehydratase FabZ	Fatty acid biosynthesis	**I**
AlbB_00737	**-**	Outer membrane protein (OmpH-like)	Cell wall/membrane/envelope biogenesis	**M**
AlbB_00738	**bamA**	Outer membrane protein assembly factor BamA precursor	Membrane assembly	**M**
AlbB_00739	**mmpA**	Metalloprotease MmpA	Inorganic ion transport and metabolism	**P**
AlbB_00740	**ygfY**	Flavinator of succinate dehydrogenase	Transcription	**K**
AlbB_00741	**zapA**	Cell division protein ZapA	Cell division	**D**
AlbB_00742	**hup**	DNA-binding protein HU	Posttranslational modification, protein turnover, chaperones	**O**
AlbB_00743	**rpsA**	30S ribosomal protein S1	Ribosome	**C**
AlbB_00747	**dapF**	Diaminopimelate epimerase	Lysine biosynthesis	**E**
AlbB_00748	**sucC**	Succinyl-CoA ligase [ADP-forming] subunit beta	C5-Branched dibasic acid metabolism	**C**
AlbB_00749	**sucC**	Succinyl-CoA ligase [ADP-forming] subunit beta	C5-Branched dibasic acid metabolism	**C**
AlbB_00750	**rpsU**	30S ribosomal protein S21	Ribosome	**J**
AlbB_00752	**rsmA**	Ribosomal RNA small subunit methyltransferase A	Translation, ribosomal structure and biogenesis	**J**
AlbB_00755	**hemF**	Coproporphyrinogen-III oxidase, aerobic	Porphyrin metabolism	**H**
AlbB_00756	**-**	Blue-light-activated protein	General function prediction only	**R**
AlbB_00759	**parB**	putative chromosome-partitioning protein ParB	Replication, recombination and repair	**L**
AlbB_00762	**rimM**	Ribosome maturation factor RimM	Translation, ribosomal structure and biogenesis	**J**
AlbB_00763	**efp**	Elongation factor P	Translation, ribosomal structure and biogenesis	**J**
AlbB_00764	**suhB**	Inositol-1-monophosphatase	Streptomycin biosynthesis	**G**
AlbB_00765	**hemA**	5-aminolevulinate synthase	Heme biosynthesis	**H**
AlbB_00767	**htpG**	Chaperone protein HtpG	Chaperone	**O**
AlbB_00768	**-**	KAP family P-loop domain protein	General function prediction only	**R**
AlbB_00770	**recR**	Recombination protein RecR	Homologous recombination	**L**
AlbB_00775	**priA**	Primosomal protein N'	Homologous recombination	**L**
AlbB_00776	**-**	CDS	-	**S**
AlbB_00778	**thyX**	Thymidylate synthase ThyX	Pyrimidine metabolism	**E**
AlbB_00779	**murA**	UDP-N-acetylglucosamine 1-carboxyvinyltransferase	Peptidoglycan biosynthesis	**M**
AlbB_00780	**fabF**	3-oxoacyl-[acyl-carrier-protein] synthase 2	Fatty acid biosynthesis	**I**
AlbB_00781	**-**	Acyl carrier protein	Pantothenate and CoA biosynthesis	**R**
AlbB_00782	**ispA**	Farnesyl diphosphate synthase	Terpenoid backbone biosynthesis	**H**
AlbB_00783	**slyD**	FKBP-type peptidyl-prolyl cis-trans isomerase	General function prediction only	**R**
AlbB_00784	**hslU**	ATP-dependent protease ATPase subunit HslU	Posttranslational modification, protein turnover, chaperones	**O**

AlbB_00785	**hslV**	ATP-dependent protease subunit HslV	Posttranslational modification, protein turnover, chaperones	**O**
AlbB_00786	**gshA**	Glutamate-cysteine ligase	Glutathione biosynthesis	**H**
AlbB_00787	**-**	CDS	-	**S**
AlbB_00788	**hemH**	Ferrochelatase	Heme biosynthesis	**H**
AlbB_00789	**-**	CDS	-	**S**
AlbB_00790	**ndk**	Nucleoside diphosphate kinase	Purine metabolism	**F**
AlbB_00791	**argD**	Acetylornithine aminotransferase	Arginine biosynthesis	**E**
AlbB_00792	**corC**	Magnesium and cobalt efflux protein CorC	Ion transport	**P**
AlbB_00793	**-**	CDS	-	**S**
AlbB_00794	**ubiD**	3-octaprenyl-4-hydroxybenzoate carboxy-lyase	Ubiquinone and other terpenoid-quinone biosynthesis	**H**
AlbB_00795	**folD**	Bifunctional protein FolD protein	Carbon metabolism	**H**
AlbB_00798	**leuS**	Leucine--tRNA ligase	Aminoacyl-tRNA biosynthesis	**J**
AlbB_00799	**nlpD**	Murein hydrolase activator NlpD precursor	Cell wall/membrane/envelope biogenesis	**M**
AlbB_00800	**-**	CDS	-	**S**
AlbB_00801	**ihfA**	Integration host factor subunit alpha	Replication, recombination and repair	**L**
AlbB_00809	**purD**	Phosphoribosylamine--glycine ligase	Purine metabolism	**F**
AlbB_00810	**serS**	Serine--tRNA ligase	Aminoacyl-tRNA biosynthesis	**J**
AlbB_00814	**rplL**	50S ribosomal protein L7/L12	Ribosome	**J**
AlbB_00815	**rplJ**	50S ribosomal protein L10	Ribosome	**J**
AlbB_00816	**rplA**	50S ribosomal protein L1	Ribosome	**J**
AlbB_00817	**rplK**	50S ribosomal protein L11	Ribosome	**J**
AlbB_00818	**-**	CDS	-	**S**
AlbB_00821	**rpsG**	30S ribosomal protein S7	Ribosome	**J**
AlbB_00822	**rpsL**	30S ribosomal protein S12	Ribosome	**J**
AlbB_00823	**prmC**	Release factor glutamine methyltransferase	Aminobenzoate degradation	**J**
AlbB_00825	**osmY**	Osmotically-inducible protein Y precursor	General function prediction only	**R**
AlbB_00828	**clpP**	ATP-dependent Clp protease proteolytic subunit	Misfolded protein degradation	**O**
AlbB_00829	**clpA**	ATP-dependent Clp protease ATP-binding subunit ClpA	Misfolded protein degradation	**O**
AlbB_00831	**spoVD**	Stage V sporulation protein D	General function prediction only	**R**
AlbB_00832	**pgdA**	Peptidoglycan-N-acetylglucosamine deacetylase	General function prediction only	**R**
AlbB_00833	**comM**	Competence protein ComM	Replication, recombination and repair	**L**
AlbB_00835	**-**	CDS	-	**S**
AlbB_00838	**ybeY**	Endoribonuclease YbeY	Translation, ribosomal structure and biogenesis	**J**
AlbB_00839	**sdhB**	Succinate dehydrogenase iron-sulfur subunit	Butanoate metabolism	**C**
AlbB_00840	**divL**	Sensor protein DivL	Signal transduction	**U**
AlbB_00841	**-**	acetyltransferase	General function prediction only	**R**
AlbB_00842	**pgpA**	Phosphatidylglycerophosphatase A	Glycerophospholipid metabolism	**I**
AlbB_00843	**gpsA**	Glycerol-3-phosphate dehydrogenase [NAD(P)+]	Glycerophospholipid metabolism	**C**
AlbB_00844	**dut**	Deoxyuridine 5'-triphosphate nucleotidohydrolase	Pyrimidine metabolism	**F**
AlbB_00846	**-**	CDS	-	**S**
AlbB_00848	**yidC**	Membrane protein insertase YidC	Bacterial secretion system	**U**
AlbB_00852	**secF**	preprotein translocase subunit SecF	Bacterial secretion system	**U**
AlbB_00853	**dnaA**	Chromosomal replication initiator protein DnaA	Two-component system	**L**
AlbB_00854	**puuA**	Gamma-glutamylputrescine synthetase PuuA	Arginine and proline metabolism	
AlbB_00857	**ctrA**	Cell cycle response regulator CtrA	Cell cycle control	**D**
AlbB_00860	**nuoD**	NADH-quinone oxidoreductase subunit D	Oxidative phosphorylation	**C**
AlbB_00862	**-**	Nitronate monooxygenase	General function prediction only	**R**
AlbB_00864	**-**	CDS	-	**S**
AlbB_00865	**virB4**	Type IV secretion system protein virB4	Bacterial secretion system	**U**
AlbB_00866	**-**	CDS	-	**S**
AlbB_00869	**estB**	Carboxylesterase 2	Drug metabolism - other enzymes	
AlbB_00872	**mnmG**	tRNA uridine 5-carboxymethylaminomethyl modification enzyme MnmG	Translation, ribosomal structure and biogenesis	**J**
AlbB_00876	**murJ**	putative peptidoglycan biosynthesis protein MurJ	Cell wall/membrane/envelope biogenesis	**M**
AlbB_00878	**-**	CDS	-	**S**
AlbB_00880	**sodB**	Superoxide dismutase [Fe]	FoxO signaling pathway	
AlbB_00881	**ptrA**	Protease 3 precursor	Amino acid transport and metabolism	**E**
AlbB_00883	**tgt**	queuine tRNA-ribosyltransferase	Translation, ribosomal structure and biogenesis	**J**
AlbB_00884	**nqo2**	NADH-quinone oxidoreductase chain 2	Carbohydrate transport and metabolism	**G**
AlbB_00887	**rlmE**	Ribosomal RNA large subunit methyltransferase E	Translation, ribosomal structure and biogenesis	**J**
AlbB_00888	**-**	CDS	-	**S**
AlbB_00889	**bepC**	Outer membrane efflux protein BepC precursor	Intracellular trafficking, secretion, and vesicular transport	**U**
AlbB_00890	**rplM**	50S ribosomal protein L13	Ribosome	**J**
AlbB_00891	**rpsI**	30S ribosomal protein S9	Ribosome	**J**
AlbB_00892	**hupB**	DNA-binding protein HRm	Posttranslational modification, protein turnover, chaperones	**O**
AlbB_00893	**pdxJ**	Pyridoxine 5'-phosphate synthase	Vitamin B metabolism	**H**
AlbB_00895	**gltX**	Glutamate--tRNA ligase	Porphyrin metabolism	**M**
AlbB_00896	**-**	CDS	-	**S**
AlbB_00897	**accA1**	Acetyl-/propionyl-coenzyme A carboxylase alpha chain	Fatty acid biosynthesis	**I**
AlbB_00898	**ctaA**	Heme A synthase	Benzoate degradation	**O**
AlbB_00901	**atpF**	F0F1 ATP synthase subunit B	Oxidative phosphorylation	**C**
AlbB_00902	**atpF2**	ATP synthase subunit b 2	Energy production and conversion	**C**
AlbB_00903	**atpE**	ATP synthase subunit c	Oxidative phosphorylation	**C**
AlbB_00904	**atpB**	ATP synthase subunit a	Oxidative phosphorylation	**C**
AlbB_00906	**pheT**	Phenylalanine--tRNA ligase beta subunit	Aminoacyl-tRNA biosynthesis	**J**
AlbB_00908	**ileS**	Isoleucine--tRNA ligase	Aminoacyl-tRNA biosynthesis	**J**
AlbB_00911	**recJ**	Single-stranded-DNA-specific exonuclease RecJ	Base excision repair	**L**
AlbB_00912	**rsmA**	Ribosomal RNA small subunit methyltransferase A	Translation, ribosomal structure and biogenesis	**J**

AlbB_00913	**-**	CDS	-	**S**
AlbB_00914	**-**	TrbL/VirB6 plasmid conjugal transfer protein	Bacterial secretion system	**U**
AlbB_00915	**-**	TrbL/VirB6 plasmid conjugal transfer protein	Bacterial secretion system	**U**
AlbB_00916	**-**	TrbL/VirB6 plasmid conjugal transfer protein	Bacterial secretion system	**U**
AlbB_00917	**-**	TrbL/VirB6 plasmid conjugal transfer protein	Bacterial secretion system	**U**
AlbB_00919	**-**	Type IV secretory pathway, VirB3-like protein	Bacterial secretion system	**V**
AlbB_00920	**lysS**	Lysine--tRNA ligase	Aminoacyl-tRNA biosynthesis	**J**
AlbB_00924	**cysW**	Sulfate transport system permease protein CysW	Sulfur metabolism	**P**
AlbB_00925	**apaG**	CO2+/MG2+ efflux protein ApaG	Inorganic ion transport and metabolism	**P**
AlbB_00926	**purK**	N5-carboxyaminoimidazole ribonucleotide synthase	Purine metabolism	**F**
AlbB_00927	**asd2**	Aspartate-semialdehyde dehydrogenase 2	Lysine biosynthesis	**E**
AlbB_00929	**adiC**	Arginine/agmatine antiporter	Amino acid transport and metabolism	**E**
AlbB_00932	**surE**	5'-nucleotidase SurE	Nicotinate and nicotinamide metabolism	**E**
AlbB_00933	**hisS**	Histidine--tRNA ligase	Aminoacyl-tRNA biosynthesis	**J**
AlbB_00934	**nfuA**	Fe/S biogenesis protein NfuA	Coenzyme transport and metabolism	**H**
AlbB_00939	**petC**	Cytochrome c1 precursor	Oxidative phosphorylation	**C**
AlbB_00944	**-**	CDS	-	**S**
AlbB_00945	**-**	Lipase (class 3)	Fatty acid metabolism	**I**
AlbB_00946	**-**	putative 3'-5' exonuclease related to the exonuclease domain of PolB	Replication, recombination and repair	**L**
AlbB_00947	**era**	GTPase Era	Cell cycle control, cell division, chromosome partitioning	**D**
AlbB_00948	**-**	CDS	-	**S**
AlbB_00950	**rnd**	ribonuclease D	Translation, ribosomal structure and biogenesis	**J**
AlbB_00951	**trxA**	Thioredoxin	Mulitple COGs	**OC**
AlbB_00954	**-**	CDS	-	**S**
AlbB_00956	**rbfA**	Ribosome-binding factor A	Translation, ribosomal structure and biogenesis	**J**
AlbB_00957	**ubiA**	4-hydroxybenzoate octaprenyltransferase	Ubiquinone and other terpenoid-quinone biosynthesis	**H**
AlbB_00965	**-**	CDS	-	**S**
AlbB_00968	**rplI**	50S ribosomal protein L9	Ribosome	**J**
AlbB_00969	**rpsR**	30S ribosomal protein S18	Ribosome	**J**
AlbB_00970	**rpsF**	30S ribosomal protein S6	Ribosome	**J**
AlbB_00971	**dnaE1**	DNA polymerase III subunit alpha	Base excision repair	**L**
AlbB_00973	**ykfA**	putative murein peptide carboxypeptidase	Phenylpropanoid biosynthesis	**R**
AlbB_00974	**lipB**	Octanoyltransferase	Lipoate biosynthesis	**H**
AlbB_00977	**mutS**	DNA mismatch repair protein MutS	Mismatch repair	**L**
AlbB_00979	**ccmF**	Cytochrome c-type biogenesis protein CcmF	Oxidative phosphorylation	**O**
AlbB_00981	**dacF**	D-alanyl-D-alanine carboxypeptidase DacF precursor	Cell wall/membrane/envelope biogenesis	**M**
AlbB_00982	**rmuC**	DNA recombination protein RmuC	Recombination	**L**
AlbB_00983	**-**	CDS	-	**S**
AlbB_00984	**-**	CDS	-	**S**
AlbB_00986	**secA**	preprotein translocase subunit SecA	Bacterial secretion system	**MU**
AlbB_00992	**-**	CDS	-	**S**
AlbB_00993	**fdxA**	Ferredoxin 1	Iron metabolism	**P**
AlbB_00997	**ddl**	D-alanine--D-alanine ligase	D-Alanine metabolism	**M**
AlbB_00998	**ftsQ**	cell division protein FtsQ	Cell division	**M**
AlbB_01004	**pyrE**	Orotate phosphoribosyltransferase	Pyrimidine metabolism	**F**
AlbB_01005	**gltP**	Proton glutamate symport protein	Energy production and conversion	**C**
AlbB_01007	**hemC**	Porphobilinogen deaminase	Heme biosynthesis	**H**
AlbB_01009	**secD**	preprotein translocase subunit SecD	Bacterial secretion system	**U**
AlbB_01010	**-**	Surface antigen	Defense	**R**
AlbB_01013	**-**	CDS	-	**S**
AlbB_01017	**yqfL**	Putative pyruvate, phosphate dikinase regulatory protein	General function prediction only	**R**
AlbB_01018	**ccmC**	Heme exporter protein C	ABC transporters	**O**
AlbB_01021	**hofQ**	outer membrane porin HofQ	Intracellular trafficking, secretion, and vesicular transport	**U**
AlbB_01022	**guaA**	GMP synthase [glutamine-hydrolyzing]	Purine metabolism	**F**
AlbB_01023	**terC**	Integral membrane protein TerC family protein	Inorganic ion transport and metabolism	**P**
AlbB_01024	**-**	Malonyl-CoA decarboxylase (MCD)	General function prediction only	**R**
AlbB_01026	**-**	CDS	-	**S**
AlbB_01027	**lepA**	Elongation factor 4	Cell wall/membrane/envelope biogenesis	**M**
AlbB_01029	**-**	CDS	-	**S**
AlbB_01030	**grxC**	Glutaredoxin-3	Posttranslational modification, protein turnover, chaperones	**O**
AlbB_01032	**lspA**	Lipoprotein signal peptidase	Protein export	**MU**
AlbB_01033	**ptrA**	Protease 3 precursor	Amino acid transport and metabolism	**E**
AlbB_01034	**ymfF**	Peptidase M16 inactive domain protein	General function prediction only	**R**
AlbB_01035	**purN**	Phosphoribosylglycinamide formyltransferase	Purine metabolism	**F**
AlbB_01036	**-**	CDS	-	**S**
AlbB_01037	**-**	putative monovalent cation/H+ antiporter subunit B	Bacterial secretion system	**U**
AlbB_01040	**smpB**	SsrA-binding protein	Posttranslational modification, protein turnover, chaperones	**O**
AlbB_01041	**lgt**	Prolipoprotein diacylglyceryl transferase	Glycosphingolipid biosynthesis - ganglio series	**M**
AlbB_01044	**ubiD**	3-octaprenyl-4-hydroxybenzoate carboxy-lyase	Ubiquinone and other terpenoid-quinone biosynthesis	**H**
AlbB_01046	**-**	DSBA-like thioredoxin domain protein	General function prediction only	**R**
AlbB_01051	**sucA**	2-oxoglutarate dehydrogenase E1 component	Carbon metabolism	**C**
AlbB_01053	**grpE**	heat shock protein GrpE	Chaperone	**O**
AlbB_01054	**ispB**	Octaprenyl-diphosphate synthase	Terpenoid backbone biosynthesis	**H**
AlbB_01055	**rpsP**	30S ribosomal protein S16	Ribosome	**J**
AlbB_01057	**-**	CDS	-	**S**

AlbB_01060	**rsmA**	Ribosomal RNA small subunit methyltransferase A	Translation, ribosomal structure and biogenesis	**J**
AlbB_01079	**degP1**	putative periplasmic serine endoprotease DegP-like precursor	Posttranslational modification, protein turnover, chaperones	**O**
AlbB_01080	**hflC**	Modulator of FtsH protease HflC	Protein modification	**O**
AlbB_01081	**hflK**	Modulator of FtsH protease HflK	Protein modification	**O**
AlbB_01084	**purE**	N5-carboxyaminoimidazole ribonucleotide mutase	Purine metabolism	**F**
AlbB_01085	**-**	CDS	-	**S**
AlbB_01086	**rpoZ**	DNA-directed RNA polymerase subunit omega	Purine metabolism	**K**
AlbB_01087	**-**	putative monovalent cation/H+ antiporter subunit B	Bacterial secretion system	**U**
AlbB_01088	**mrpG**	Na(+)/H(+) antiporter subunit G	Inorganic ion transport and metabolism	**P**
AlbB_01089	**mrpA**	Na(+)/H(+) antiporter subunit A	Inorganic ion transport and metabolism	**P**
AlbB_01090	**mrpB**	Na(+)/H(+) antiporter subunit B	Inorganic ion transport and metabolism	**P**
AlbB_01091	**mnhC1**	Na(+)/H(+) antiporter subunit C1	Inorganic ion transport and metabolism	**P**
AlbB_01092	**rpoD**	RNA polymerase sigma factor RpoD	Transcription	**K**
AlbB_01093	**-**	CDS	-	**S**
AlbB_01095	**-**	CDS	-	**S**
AlbB_01097	**sdhC**	Succinate dehydrogenase cytochrome b556 subunit	Butanoate metabolism	**C**
AlbB_01098	**-**	Succinate dehydrogenase/Fumarate reductase transmembrane subunit	Energy production and conversion	**C**
AlbB_01099	**-**	CDS	-	**S**
AlbB_01102	**lolC**	Lipoprotein-releasing system transmembrane protein LolC	ABC transporters	**E**
AlbB_01103	**-**	CDS	-	**S**
AlbB_01104	**-**	Anaphase-promoting complex, cyclosome, subunit 3	Protein-complex assembly	**R**
AlbB_01105	**atpG**	ATP synthase gamma chain	Oxidative phosphorylation	**C**
AlbB_01106	**pmbA**	peptidase PmbA	General function prediction only	**R**
AlbB_01107	**cspA**	Cold shock protein CspA	Posttranslational modification, protein turnover, chaperones	**O**
AlbB_01109	**clpA**	ATP-dependent Clp protease ATP-binding subunit ClpA	Misfolded protein degradation	**O**
AlbB_01111	**fbaB**	Fructose-bisphosphate aldolase class 1	Biosynthesis of amino acids	**G**
AlbB_01114	**pyrD**	Dihydroorotate dehydrogenase (quinone)	Pyrimidine metabolism	**F**
AlbB_01115	**rnc**	Ribonuclease 3	Transcription	**K**
AlbB_01116	**-**	CDS	-	**S**
AlbB_01117	**dnaQ**	DNA polymerase III subunit epsilon	Purine metabolism	**L**
AlbB_01118	**-**	Tim44-like domain protein	General function prediction only	**R**
AlbB_01119	**secB**	Protein-export protein SecB	Bacterial secretion system	**U**
AlbB_01120	**acnA**	Aconitate hydratase 1	Biosynthesis of amino acids	**C**
AlbB_01121	**putA**	Bifunctional protein PutA	Glutamate biosynthesis	**J**
AlbB_01122	**pcaH**	Protocatechuate 3,4-dioxygenase beta chain	Secondary metabolites biosynthesis, transport and catabolism	**Q**
AlbB_01123	**pepA**	Cytosol aminopeptidase	Arginine and proline metabolism	**E**
AlbB_01126	**nqo3**	NADH-quinone oxidoreductase chain 3	Carbohydrate transport and metabolism	**G**
AlbB_01127	**nuoH**	NADH-quinone oxidoreductase subunit H	Oxidative phosphorylation	**C**
AlbB_01128	**hemB**	Delta-aminolevulinic acid dehydratase	Heme biosynthesis	**H**
AlbB_01129	**-**	CDS	-	**S**
AlbB_01130	**glyQ**	Glycine--tRNA ligase alpha subunit	Aminoacyl-tRNA biosynthesis	**J**
AlbB_01133	-	CDS	-	**S**
AlbB_01134	-	Putative TrmH family tRNA/rRNA methyltransferase	General function prediction only	**R**
Anaa_00014	-	CDS	-	**S**
Anaa_00087	**bamA**	Outer membrane protein assembly factor BamA precursor	Membrane assembly	**M**
Anaa_00157	**-**	CDS	-	**S**
Anaa_00200	**ankX**	Phosphocholine transferase AnkX	Lipopolysaccharide biosynthesis	**I**
Anaa_00214	-	Cytochrome c	Oxidative phosphorylation	**C**
Anaa_00242	-	CDS	-	**S**
Anaa_00429	**ybaB**	Nucleoid-associated protein YbaB	-	**S**
Anaa_00435	**rplS**	50S ribosomal protein L19	Ribosome	**J**
Anaa_00537	-	CDS	-	**S**
Anaa_00761	**rpmF**	50S ribosomal protein L32	Ribosome	**J**
Anaa_00762	**mlaE**	putative phospholipid ABC transporter permease protein MlaE	ABC transporters	**O**
Anaa_00830	**pepQ**	Xaa-Pro dipeptidase	Amino acid transport and metabolism	**E**
Anaa_00836	**-**	CDS	-	**S**
Anaa_00907	-	putative global regulator	Coenzyme transport and metabolism	**H**
Anaa_00908	**groS**	10 kDa chaperonin	Chaperone	**O**
Anaa_00909	**groL5**	60 kDa chaperonin 5	Chaperone	**O**
Anaa_00986	**addA**	ATP-dependent helicase/nuclease subunit A	Base excision repair	**L**
Anaa_01301	**mgtE**	Magnesium transporter MgtE	Inorganic ion transport and metabolism	**P**
Anaa_01302	**prfB**	Peptide chain release factor 2	Translation, ribosomal structure and biogenesis	**J**
Anaa_01502	-	3-methyladenine DNA glycosylase	General function prediction only	**R**
Anaa_01533	**-**	CDS	-	**S**
Auuu_00015	**tuf**	elongation factor Tu	Translation	**J**
Auuu_00252	-	PD-(D/E)XK nuclease family transposase	Transposition	**L**
Auuu_00261	**ribN**	Riboflavin transporter	Coenzyme transport and metabolism	**H**
Auuu_01094	**-**	Major Facilitator Superfamily protein	General function prediction only	**R**
Bmmm_01072	**-**	CDS	-	**S**
Clee_00011	**-**	CDS	-	**S**
Clee_00276	**-**	putative ABC transporter ATP-binding protein	ABC transporters	**U**
Clee_00705	**iscS**	Cysteine desulfurase	Alanine biosynthesis	**E**
Clee_00770	**infA**	Translation initiation factor IF-1	Translation	**J**
Clee_00771	**yhdE**	Maf-like protein YhdE	General function prediction only	**R**
Clee_00779	-	Recombinase	Recombination	**L**
Cocc_00220	-	CDS	-	**S**
Cocc_00642	**mtaB**	Threonylcarbamoyladenosine tRNA methylthiotransferase MtaB	Translation, ribosomal structure and biogenesis	**J**
Cocc_00653	**lon**	Lon protease	Posttranslational modification, protein turnover, chaperones	**O**
Cocc_00664	**prfA**	Peptide chain release factor 1	Translation, ribosomal structure and biogenesis	**J**

Cocc_00685	**recG**	ATP-dependent DNA helicase RecG	Homologous recombination	**LK**
Cocc_00696	**guaB**	Inosine-5'-monophosphate dehydrogenase	Purine metabolism	**F**
Cocc_00727	**ispA**	Farnesyl diphosphate synthase	Terpenoid backbone biosynthesis	**H**
Cocc_00728	**prsE**	Type I secretion system membrane fusion protein PrsE	Bacterial secretion system	**U**
Cocc_00735	**topA**	DNA topoisomerase 1	Homologous recombination	**L**
Cocc_00781	**glyS**	Glycine--tRNA ligase beta subunit	Aminoacyl-tRNA biosynthesis	**J**
Cocc_00804	**rpoC**	DNA-directed RNA polymerase subunit beta'	Purine metabolism	**K**
Cocc_00809	**fusA**	Elongation factor G	Translation, ribosomal structure and biogenesis	**J**
Cocc_00819	**petB**	Cytochrome b	Oxidative phosphorylation	**C**
Cocc_00833	**ask**	Aspartokinase	Lysine biosynthesis	**E**
Cocc_00844	**infB**	Translation initiation factor IF-2	Translation	**J**
Cocc_00845	**addB**	ATP-dependent helicase/deoxyribonuclease subunit B	Basal transcription factors	**L**
Cocc_00865	**-**	CDS	-	**S**
Cocc_00907	**tig**	Trigger factor	Chaperone	**O**
Cocc_00953	**ftsZ**	Cell division protein FtsZ	Cell division	**D**
Cocc_00968	**virB4**	Type IV secretion system protein virB4	Bacterial secretion system	**U**
Cocc_00970	**atpD**	ATP synthase subunit beta	Oxidative phosphorylation	**C**
Cocc_01265	**valS**	Valine--tRNA ligase	Aminoacyl-tRNA biosynthesis	**J**
Haaa_00317	**ligA**	DNA ligase	Base excision repair	**U**
Haaa_00812	**-**	CDS	-	**S**
Haaa_00814	**-**	CDS	-	**S**
Oooo_00093	**-**	CDS	-	**S**
Ppee_00133	**-**	Putative multidrug export ATP-binding/permease protein	Bacterial secretion system	**U**
Ppee_00152	**murB**	UDP-N-acetylenolpyruvoylglucosamine reductase	Peptidoglycan biosynthesis	**M**
Ppee_00332	**ispDF**	Bifunctional enzyme IspD/IspF	Coenzyme transport and metabolism	**H**
Ppee_00334	**tqsA**	AI-2 transport protein TqsA	Posttranslational modification, protein turnover, chaperones	**O**
Ppee_00905	**-**	OTU-like cysteine protease	General function prediction only	**R**
Ppee_00950	**-**	CDS	-	**S**
Riii_00571	**-**	CDS	-	**S**

Supplementary Table S10. Homologs of *wsp* (*Wolbachia* surface protein) family genes with additional notes.

wMel Position	wMel locus ID	wPpe locus ID	wBm locus ID	Additional notes
471236-470310	PROKKA_00487	PROKKA_00420	none	Major surface antigen 4 precursor
483149-482217	PROKKA_00497	PROKKA_00574	PROKKA_00142	Surface antigen; Bm0432 "secreted into space around *Wolbachia*, interacts with glycolytic pathway
1023047-1023760	PROKKA_01060	none	PROKKA_00142	Major surface antigen 4 precursor. "*wsp* marker" WD_1063; Bm0284
10337-11185	PROKKA_00010	PROKKA_00834	PROKKA_00404	Outer membrane lipoprotein Omp16 precursor; Bm0152 "secreted, interacts with cytoskeleton, localizes in vacuolar space

Supplementary Table S11. Iron metabolism genes.

**Iron Metabolism Components**	
Outer membrane porins	
Outer membrane lipoprotein Omp16 precursor	
Outer membrane protein (OmpH-like)	
Fe ABC-T (iron ATP-binding cassette transporter)	
Heme biosynthesis	
hemA	5-aminolevulinate synthase
hemB	Delta-aminolevulinic acid dehydratase
hemC	Porphobilinogen deaminase
hemD	uroporphyrinogen III synthase
hemE	Uroporphyrinogen decarboxylase
hemF	Coproporphyrinogen-III oxidase, aerobic
hemH	Ferrochelatase
hemJ	protoporphyrinogen oxidase
Iron-sulfer cluster synthesis	
iscS	Cysteine desulfurase
iscA	Iron-binding protein IscA
hscA	chaperone protein HscA
hscB	Co-chaperone protein HscB homolog
fdxA	Ferredoxin 1
nfuA	Fe/S biogenesis protein NfuA
nifU	NifU-like protein
NADH dehydrogenase	
idiA	Iron deficiency-induced protein A precursor
cysW	Sulfate transport system permease protein CysW
fbpC	Fe(3+) ions import ATP-binding protein FbpC
ccmB	Heme exporter protein B
ccmC	Heme exporter protein C (NOTinPpe)
ccmA	Cytochrome c biogenesis ATP-binding export protein CcmA
nuoB	NADH-quinone oxidoreductase subunit B
nuoC	NADH-quinone oxidoreductase subunit C
nuoC1	NADH-quinone oxidoreductase subunit C 1
nuoD	NADH-quinone oxidoreductase subunit D
nuoF	NADH-quinone oxidoreductase subunit F
nuoH	NADH-quinone oxidoreductase subunit H
nuoI	NADH-quinone oxidoreductase subunit I
nuoJ	NADH-quinone oxidoreductase subunit J
nuoK	NADH-quinone oxidoreductase subunit K
nuoL	NADH-quinone oxidoreductase subunit L
nuoM	NADH-quinone oxidoreductase subunit M

nuoN	NADH-quinone oxidoreductase subunit N
ndhD1	NAD(P)H-quinone oxidoreductase chain 4 1
ndhC	NAD(P)H-quinone oxidoreductase subunit 3
nqo2	NADH-quinone oxidoreductase chain 2
nqo3	NADH-quinone oxidoreductase chain 3

Succinate dehydrogenase

sdhA	Succinate dehydrogenase flavoprotein subunit
sdhB	Succinate dehydrogenase iron-sulfur subunit
sdhC	Succinate dehydrogenase cytochrome b556 subunit

Cytochrome c reductase

petA	Ubiquinol-cytochrome c reductase iron-sulfur subunit
petB	Cytochrome b
petC	Cytochrome c1 precursor

Cytochrome c oxidase

ctaA	Heme A synthase
ctaB	Protoheme IX farnesyltransferase
ctaC	Cytochrome c oxidase subunit 2 precursor
ctaD	Cytochrome c oxidase subunit 1
ctaE	Cytochrome c oxidase subunit 3
ctaG	Cytochrome c oxidase assembly protein CtaG

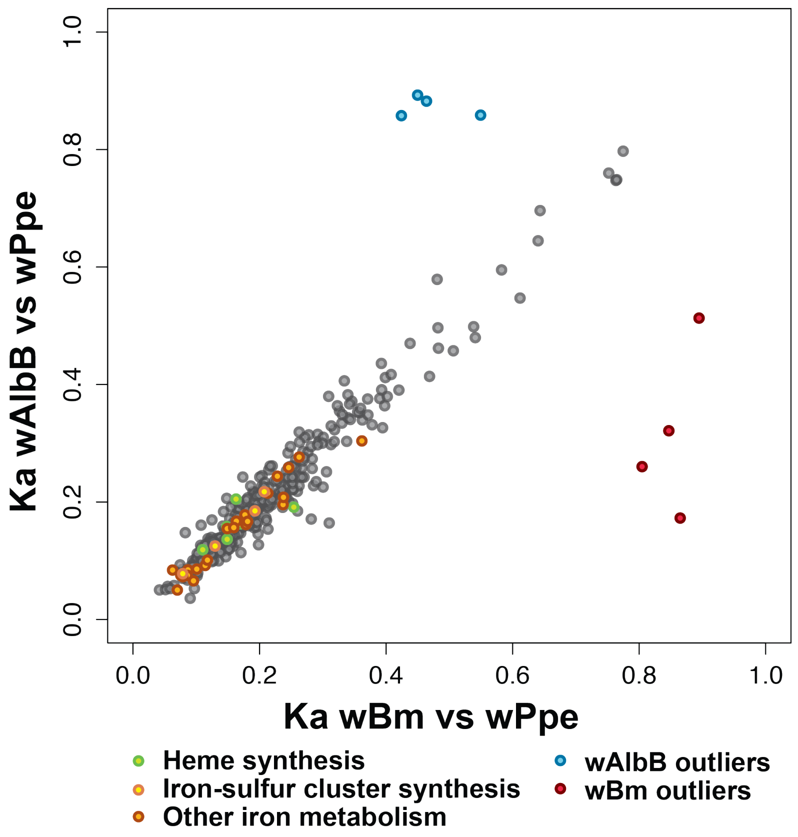

Supplementary Figure S23. Amino acid substitution rates, Ka, among shared orthologs for *Wolbachia* strains wAlbB and wBm compared to wPpe. Green, amber, and orange circles represent iron-related genes. Grey circles represent all other shared orthologs. Blue and red circles represent outliers suggestive of different substitution rates in the different supergroups.

<u>Outliers in Red (low Ka in wAlbB, high Ka in wBm):</u>

Ppee_00060 Uncharacterized
Ppee_00933 Inner membrane protein next to transposase (SNARE associated Golgi protein)
Ppee_00847 Uncharacterized with conserved position between peptidyl-prolyl cic-trans isomerase D (protein folding in outer membrane) and inosine-5'-monophosphase dehydrogenase (de novo purine biosynthesis)
Ppee_00122 Uncharacterized

<u>Outliers in Blue (low Ka in wBm, high Ka in wAlbB):</u>

Ppee_00696 Uncharacterized gene next to fatty acid synthesis gene
Ppee_00855 Uncharacterized protein always next to a ferrochelatase involved in heme synthesis
Ppee_00797 Uncharacterized gene to terpenoid synthesis gene
Ppee_00126 Outer membrane protein always between Rhodocoxin (2Fe-2S ferredoxin) involved in Iron-sulfur assembly, and cytochrome-c ccmH

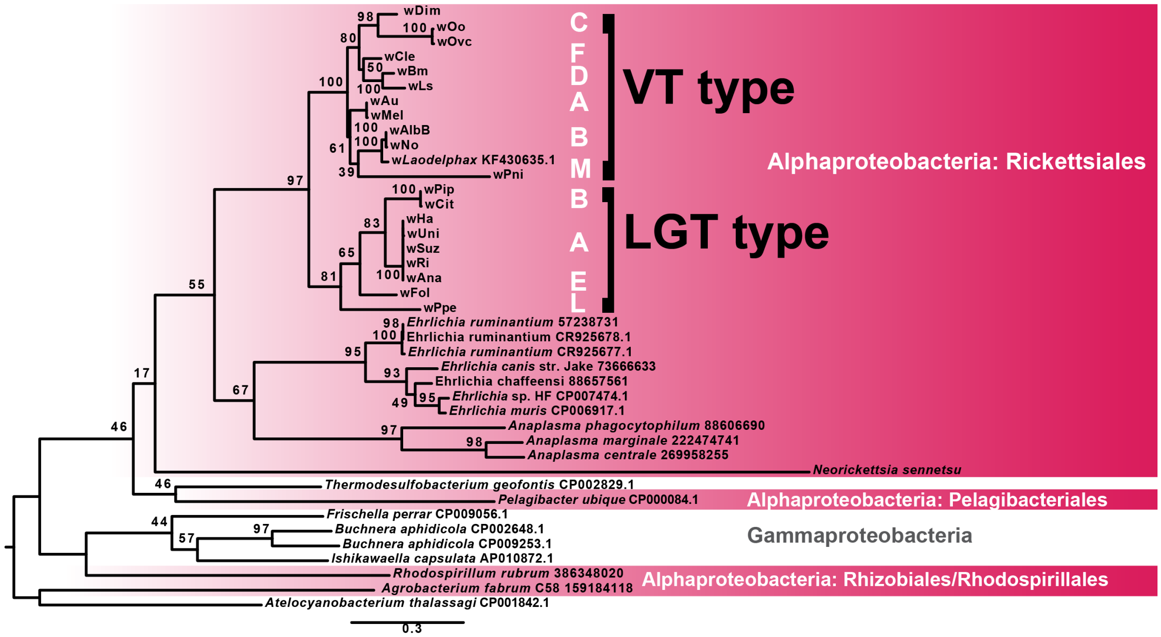

Supplementary Figure S24. Maximum likelihood phylogeny of the 3,4-dihydroxy-2-butanone 4-phosphaste synthase (*ribB*) gene for 21 *Wolbachia* strains and 19 outgroups, comprising 507 nucleotide positions, with bootstrap values from 1,000 replicates shown on nodes. Supergroups (A, B, C, D, F, E, L, M in white font) formed two clades, matching those found previously (Moriyama et al. 2015) with a vertical transfer clade "VT type" mirroring the phylogeny from the rest of the genome, and a possible lateral gene transfer "LGT type". The gene *ribB* appears to not be monophyletic for alphaproteobacteria (pink shading), although there is little to no bootstrap support for deep branches.
